# Synthesis of 3‐Amino‐1‐benzothiophene‐1,1‐diones by Alkyne Directed Hydroarylation and 1/N→3/C‐Sulfonyl Migration

**DOI:** 10.1002/ejoc.201801062

**Published:** 2018-10-10

**Authors:** Ivan Bernar, Daniel Blanco‐Ania, Sophie J. Stok, Lia Sotorríos, Enrique Gómez‐Bengoa, Floris P. J. T. Rutjes

**Affiliations:** ^1^ Radboud University Institute for Molecules and Materials Heyendaalseweg 135 6525 AJ Nijmegen the Netherlands; ^2^ Department of Organic Chemistry I University of the Basque Country (UPV/EHU) P.O. Box 1072 20080 Donostia‐San Sebastián Spain

**Keywords:** Ynamines, Homogeneous catalysis, Palladium, C–H activation, Synthetic methods

## Abstract

A completely regioselective and highly stereoselective palladium‐catalyzed intramolecular hydroarylation of arenesulfonyl ynamines to benzothiazoles was developed. The presence of an electron‐withdrawing group on the triple bond of the sulfonyl ynamine was crucial for the success of the reaction and our mechanistic studies suggest an alkyne‐directed 5‐*exo*‐dig cyclization pathway. The products easily underwent photoinduced rearrangement to 3‐amino‐1‐benzothiophene‐1,1‐diones (up to 35 % yields after two steps).

## Introduction

**Scheme 1:**
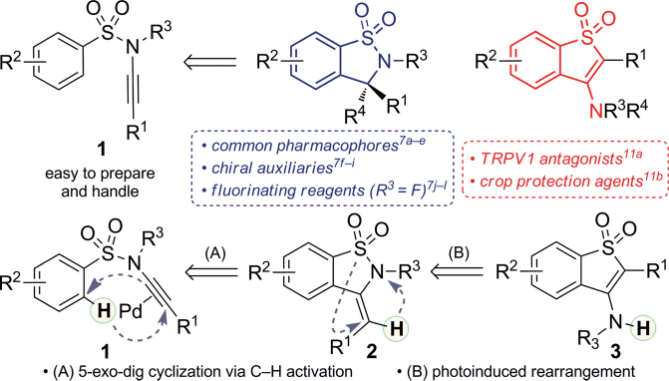
Syntheses and applications of 1,2‐benzothiazole‐1,1‐diones and 3‐amino‐1‐benzothiophene‐1,1‐diones.

Sulfonyl ynamines have recently emerged as a privileged class of ynamides.^1^ They are stable compounds, readily prepared^2^ and easy to handle. In addition, the polarized alkyne system of sulfonyl ynamines shows excellent reactivity in a wide variety of reactions featuring π‐acid catalysis,^3^ metal‐catalyzed cyclizations,^4^ cycloadditions^5^ and rearrangements^6^ for the synthesis of complex nitrogen‐containing heterocycles and natural products. With the idea of extending the chemistry of these ynamines, we envisioned that arenesulfonyl ynamines **1** could be used as simple precursors for a single step, atom‐efficient synthesis of 1,2‐benzothiazole‐1,1‐diones, key elements of diverse biologically active compounds and useful reagents in organic synthesis (Scheme 1).^7^

Considering that compounds **1** react in the presence of alkynophilic transition metals usually through π‐activation of the triple bond, we anticipated that initial alkyne coordination might direct an aromatic *ortho*‐C–H activation followed by a stereocontrolled intramolecular hydroarylation (pathway A, Scheme 1), similar to that of *N*‐alkynyl indoles developed by Park et al.^8^ or alkynyl aryl ethers developed by Hiyama et al.^9^ Moreover, excitation of the obtained benzothiazolediones **2** with light could trigger a [1,3]sigmatropic rearrangement of the sulfonyl group by S–N bond cleavage^10^ followed by colligation to 3‐amino‐1‐benzothiophene‐1,1‐diones **3** (pathway B, Scheme 1).^11^ In particular, our interest in these compounds was awakened because they are potential crop protecting agents, and therefore beneficial for the ECHONET program we are part of.^12^ To the best of our knowledge, alkyne‐directed 5‐*exo*‐dig cyclizations of arenesulfonyl ynamines to 1,2‐benzothiazole‐1,1‐diones and its following rearrangement to 3‐amino‐1‐benzothiophene‐1,1‐diones have not been yet explored.

## Results and Discussion

We initially investigated the behavior of sulfonyl ynamine **1a** in the presence of alkynophilic transition metals and Brønsted acids (Table 1; for detailed information on the reaction optimization, see the Supporting Information). The reaction took place only with palladium‐based catalysts. Thus, sulfonyl ynamine **1a** was converted to products (*E/Z*)‐**2a**, **3**, **4** and **5** in the presence of Pd(OAc)_2_ (5 mol‐%) in toluene at 100 °C for 18 h (Table 1, entry 1). The (*E*)‐exocyclic alkylidene product **2a** was isolated in 21 % yield after purification of the crude mixture by neutral silica‐gel column chromatography.

**Table 1:** Reaction optimization

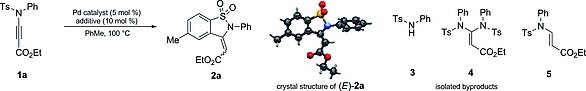									
			Ratio of products [%]^a^						
Entry	Catalyst	Additive	**1a**	**2a**	**3**	**4**	**5**	*E/Z*‐ratio^a^	Yield (*E*)‐**2a** [%]^b^
1	Pd(OAc)_2_		27	35	33	3	2	82:18	21
2	Pd(PPh_3_)_4_		30	10	58	2	0	90:10	7
3	Pd(PPh_3_)_4_	HOAc	43	23	17	3	14	92:8	14
4	Pd(OAc)_2_	Zn	23	37	38	2	0	85:15	20
5	Pd(OAc)_2_	NaOAc^c^	28	14	58	0	0	85:15	8
6	Pd(OAc)_2_/PCy_3_		13	48	36	0	3	90:10	27
7	Pd(OAc)_2_/P(*p*‐tol)_3_		10	51	35	0	4	98:2	37

a Reaction conditions: **1a** (1.0 mmol), Pd catalyst (5 mol‐%), phosphine ligand (10 mol‐%), additive (10 mol‐%), PhMe (10.0 mL), 100 °C for 18 h. [a] Based on the ^1^H NMR spectrum of the crude.

b Isolated yield.

c NaOAc (2.0 equiv.).

The structure of (*E*)‐**2a** was unambiguously determined by NMR spectroscopy and X‐ray crystallography. This reaction also showed degradation of sulfonyl ynamine **1a** under the reaction conditions forming *N*‐phenyl sulfonamide **3**, which was the main byproduct of the ynamide cyclization. Compound **3** reacted further with **1a** to form Michael adduct **4**, which was successfully isolated after purification. Furthermore, sulfonyl ynamine **1a** underwent partial hydrogenation of the triple bond to yield sulfonyl enamine **5** in a trace amount.^13^ The reaction also took place in the presence of palladium(0), albeit in a much lower conversion compared to Pd(OAc)_2_ (entries 2 and 3). The use of Pd(OAc)_2_ and Zn, a catalytic system developed by Hiyama et al. for the cyclization of alkynyl aryl ethers,^9^ had no influence on the yield of **2a** (entry 4). These initial studies showed that formation of benzothiazoledione **2a** took place without any further additives (e.g., NaOAc, entry 5). Finally, addition of phosphine ligands promoted the cyclization. Bulky ligands led in general to higher yields and better ratios of the stereoisomers (entries 6 and 7).

Although the yields are relatively low, this unprecedented intramolecular hydroarylation reaction is completely regioselective and highly stereoselective: (*E*)‐exocyclic 1,2‐benzothiazole‐1,1‐dione **2a** is the major isomer with an *E/Z*‐ratio of up to 98:2, confirmed by NOE studies in all cases.

**Scheme 2:**
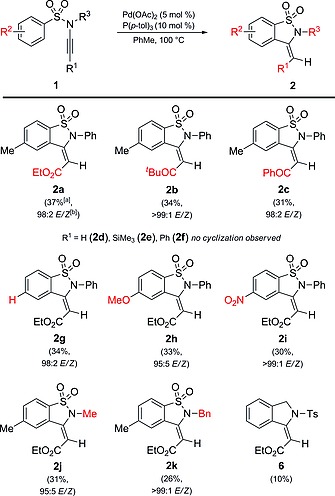
Scope of sulfonyl ynamines for intramolecular hydroarylation. Reaction conditions: **1** (1.0 mmol), Pd(OAc)_2_ (5 mol‐%), P(*p*‐tol)_3_ (10 mol‐%), PhMe (10.0 mL), 100 °C for 18 h. [a] Isolated yield. [b] Based on the ^1^H NMR spectrum of the crude mixture.

With these results in hand, we focused our attention on the scope and limitations of the reaction varying the substituents on the alkyne terminus (R^1^), on the aromatic ring of the sulfonamide group (R^2^) and on the nitrogen (R^3^) for the hydroarylation of sulfonyl ynamines **1a**–**k** (Scheme 2). Notably, the presence of an electron‐withdrawing group on the triple bond, e.g., ester or ketone, was essential for the success of the hydroarylation. Thus, sulfonyl ynamines with a ketone showed similar reactivity to sulfonyl ynamine **1a** proceeding with exclusive regioselectivity and excellent stereoselectivity (**2b**–**2c**). In stark contrast, when the reaction was performed with sulfonyl ynamines **1d**–**1f** (R = H, SiMe_3_, Ph) no cyclization was detected and only decomposition to **3** was observed. Changing the conditions (solvent, temperature, Pd catalyst, addition of base) did not lead to products **2d**–**2f** either.

Next, a series of electronically different sulfonyl ynamines were employed for this transformation. To our delight, we observed no significant difference in the reaction efficiency when changing the substituents at the aryl group of the sulfonamide. 1,2‐Benzothiazole‐1,1‐diones (**2g**–**i**) were obtained in comparable yields and also with complete regioselectivity and with excellent stereoselectivity. Finally, sulfonyl ynamines **1j** and **1k** were subjected to the reaction conditions to compare the reactivity of compounds with different substitution on the nitrogen. Compound **1j** formed product **2j** in 31 % yield and compound **1k** resulted in the formation of a mixture of **2k** (>99:1 *E/Z* ratio) and **6** in 26 and 10 % yield, respectively.^14^

**Figure 1:**
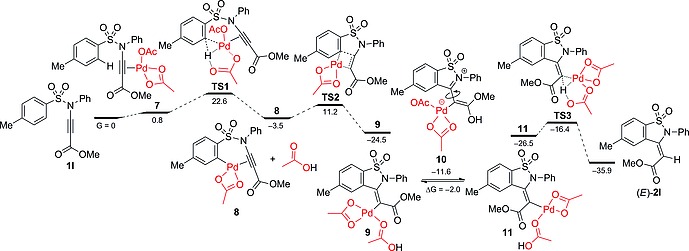
Computed pathway for cyclization of **1l**. Free energies (298 K) with respect to starting materials are shown in kcal/mol.

For a better understanding of the reaction mechanism, we investigated the reaction pathway with DFT calculations (Figure 1). The most logical mechanistic scenario for the reaction of sulfonyl ynamine **1l** and Pd(OAc)_2_ would involve coordination of the Pd^II^ species to the substrate, as in **7**, followed by an alkyne‐directed concerted deprotonation‐metalation sequence (CDM).^15^ Thus, calculations indicate transition state **TS1** in which one of the acetate groups is acting both as a κ^2^ ligand and as a base. The proton abstraction presents the highest activation barrier within the catalytic cycle (22.6 kcal/mol), and therefore should be considered as the rate‐determining step of the reaction. Additionally, *ortho*‐C–H activation in **TS1** proceeds much slower for the alkyne–H and alkyne–SiMe_3_ substructures (24.5 and 24.6 kcal/mol, for **1d** and **1e**, respectively) compared to the corresponding alkyne–CO_2_Me substructure **1l**. The difference between these barrier values is >2.5 kcal/mol corresponding to more than 100 times slower reaction rate, which is in agreement with the experimental results. Consequently, the intermediate **8** performs a carbopalladation to the CC triple bond through **TS2**, leading to intermediate **9**. Also, this step was predicted to be a few kcal/mol higher for sulfonyl ynamines **1d** and **1e** (16.0 and 14.2 kcal/mol respectively) than for sulfonyl ynamine **1l** (11.2 kcal/mol). Further protonation of the “push‐pull” alkenyl–Pd^II^ species **9** with acetic acid forms an enol intermediate **10**, with almost free rotation around the exocyclic C–C bond. We found that the enol structure **10** lies 12.9 kcal/mol higher in energy than palladium species **9**, confirming the feasibility of this or related isomerization processes. The final protodemetalation with acetic acid renders the major product (*E*)‐**2a** and recovers the active Pd^II^‐acetate species.

**Scheme 3:**
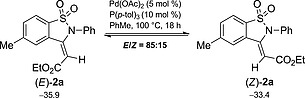
Equilibration between (*E*)‐ and (*Z*)‐isomers of **2a**. Free energies (298 K) are shown in kcal/mol.

Our DFT calculations further reveal that the (*E*)‐isomer is the most stable isomer [2.5 kcal/mol Lower in energy than the (*Z*)‐isomer, Scheme 3]. To validate this result, (*E*)‐**2a** and (*Z*)‐**2a** were subjected independently to the same experimental conditions. We observed the formation of an 85:15 *E/Z* mixture of isomers after 18 h of reaction time. These results suggest that the cyclization of **1a** might proceed in an exceptional stereoselective manner (as proposed in Scheme 2), however the major product further undergoes partial isomerization under the reaction conditions.

**Scheme 4:**
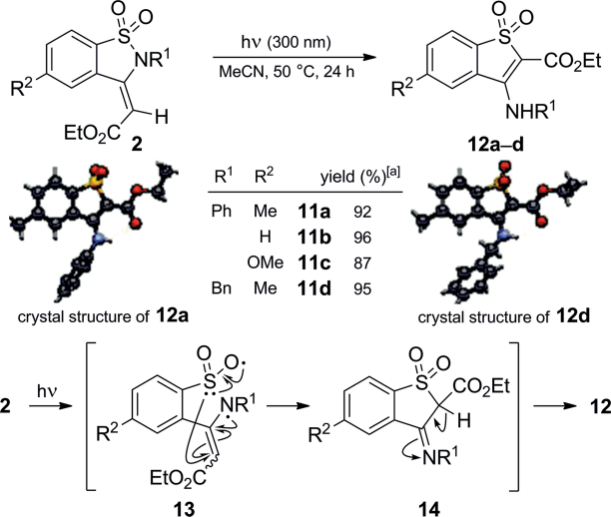
Photochemical rearrangement of 1,2‐benzothiazole‐1,1‐diones **2** to 3‐amino‐1‐benzothiophene‐1,1‐diones **12**. Reaction conditions: **2** (0.1 mmol), CH_3_CN (10.0 mL), hν (300 nm), 24 h. [a] Isolated yield.

Finally, 1,2‐benzothiazole‐1,1‐diones **2** underwent a previously unexplored photoinduced rearrangement to 3‐amino‐1‐benzothiophene‐1,1‐diones **12** while being irradiated with UV light (300 nm; Scheme 4).

Thus, when either stereoisomer of **2a** or the mixture of both was exposed to irradiation at 50 °C for 24 h, a complete photochemical conversion to 3‐amino‐1‐benzothiophene‐1,1‐dione **12a** was observed. Furthermore, to show the scope of this transformation, several 1,2‐benzothiazoledione derivatives were subjected to the same reaction conditions. The corresponding products **12b**–**12d** were obtained in excellent yields in all cases. The structure of the products was confirmed by selNOE spectroscopy and X‐ray crystallography of **12a** and **12d**. We also propose a plausible mechanism on the basis of previously reported photoinduced cleavage of sulfonamides.^7^ This [1,3]sigmatropic rearrangement entails a homolytic cleavage of the sulfonamide S–N bond of 1,2‐benzothiazoledione **2**, followed by recombination of the resulting sulfinate radical with the C‐terminus of the enaminyl radical (**13**). Subsequent tautomerization of the formed imine **14** results in 3‐amino‐1‐benzothiophene‐1,1‐dione **12**.

## Conclusions

In summary, we have demonstrated a completely regioselective and highly stereoselective intramolecular hydroarylation of sulfonyl ynamines. This method opens an easy access to benzothiazole heterocycles, valuable scaffolds in medicinal and organic synthesis. Our mechanistic studies suggest an alkyne‐directed 5‐*exo*‐dig intramolecular cyclization pathway, where the presence of an electron‐withdrawing group at the triple bond was key for the success of the reaction. Moreover, we have discovered the first example of photoinduced rearrangement of 1,2‐benzothiazole‐1,1‐diones to form 3‐amino‐1‐benzothiophene‐1,1‐dione derivatives in excellent yields. Optimization studies that broaden the synthetic scope and applications of the reaction are currently under investigation.

## Experimental Section

**General Information:** Reagents were obtained from commercial suppliers and were used without purification. Standard syringe techniques were applied for the transfer of dry solvents and air‐ or moisture‐sensitive reagents. All inert reactions were carried out under a nitrogen atmosphere using flame‐dried flasks. If stated, reactions were performed in Biotage Initiator+ Microwave Synthesizer under a nitrogen atmosphere. ^1^H and ^13^C NMR spectra were recorded at 298 K on a Varian Inova 400 MHz or Bruker 500 MHz spectrometer in the solvent indicated. Chemical shifts are given in parts per million (ppm) with respect to tetramethylsilane (*δ* =0.00 ppm) as internal standard for ^1^H NMR; and CDCl_3_ (*δ* =77.16 ppm) as internal standard for ^13^C NMR spectroscopy. Coupling constants are reported as *J* values in Hertz (Hz). ^1^H NMR spectroscopic data are reported as follows: chemical shift (ppm), multiplicity (s = singlet, d = doublet, quint = quintet, t = triplet and combination of them), coupling constants (Hz) and integration. All NMR signals were assigned on the basis of ^1^H NMR, ^13^C NMR, gCOSY, gHSQC, gHMBS and NOESY experiments. Mass spectra were recorded on a JEOL AccuTOF CS JMST100CS (ESI) mass spectrometer. Automatic flash column chromatography was performed using Biotage Isolera Spektra One, using SNAP cartridges (Biotage, 30–100 µm, 60 Å), 10–50 g. Analysis by TLC was conducted on Silica gel F254 (Merck KGaA) plates with detection by UV absorption (254 nm) where applicable, and by dipping into a solution of aqueous KMnO_4_/Na_2_CO_3_/NaOH solution followed by charring at ca. 150 °C. IR spectra were recorded on a Bruker Tensor 27 FTIR spectrometer.

**General Procedure for the Synthesis of Sulfonamides:** Sulfonamides **3** and **S1**–**S5** were prepared by the literature procedure reported by Murphy et al.^16^ The sulfonyl chloride (11 mmol, 1.1 equiv.) was added to a solution of the amine (10 mmol, 1 equiv.) and pyridine (950 mg, 12 mmol, 1.2 equiv.) in CH_2_Cl_2_ (30 mL) portion‐wise with stirring. The reaction mixture was then stirred at 23° for 12 h before evaporation of CH_2_Cl_2_ and quenching with an aqueous NaOH solution (2 n, 100 mL). The aqueous solution was rinsed with diethyl ether (2 × 50 mL) then acidified with concentrated HCl and extracted with CH_2_Cl_2_ (3 × 50 mL). The combined organic washings were dried with sodium sulfate and concentrated in vacuo. The obtained crude product was used directly in the next step without further purification.

***N*‐Phenylbenzenesulfonamide (S1):** Sulfonamide **S1** was prepared from aniline (0.9 g, 10 mmol) and benzenesulfonyl chloride (1.9 g, 11 mmol) according to the general procedure and obtained in 90 % yield as a white solid. ^1^H NMR [400 MHz, CDCl_3_]: *δ* = 6.58 (br. s, 1 H): *δ* = 7.02–7.09 (m, 2 H), 7.09–7.18 (m, 1 H), 7.20–7.29 (m, 2 H), 7.40–7.48 (m, 2 H), 7.50–7.58 (m, 1 H), 7.72–7.79 (m, 2 H) ppm. ^13^C NMR [101 MHz, CDCl_3_]: *δ* = 121.6, 125.4, 127.3, 129.3, 129.7, 133.0, 136.5, 139.0 ppm. These data were in accordance to those reported in the literature.^17^

**4‐Methyl‐*N*‐phenylbenzenesulfonamide (3):** Sulfonamide **3** was prepared from aniline (0.9 g, 10 mmol) and 4‐methylbenzenesulfonyl chloride (2.1 g, 11 mmol) according to the general procedure and obtained in 92 % yield as a white solid. ^1^H NMR [400 MHz, CDCl_3_]: *δ* = 2.35 (s, 3 H), 6.61 (br. s, 1 H), 7.02–7.10 (m, 2 H), 7.12–7.25 (m, 5 H), 7.62–7.68 (m, 2 H) ppm. ^13^C NMR [101 MHz, CDCl_3_]: *δ* = 21.6, 121.5, 125.1, 127.5, 129.2, 129.6, 136.0, 136.4, 143.9 ppm. These data were in accordance to those reported in the literature.^17^

**4‐Methoxy‐*N*‐phenylbenzenesulfonamide (S2):** Sulfonamide **S2** was prepared from aniline (0.9 g, 10 mmol) and 4‐methoxybenzenesulfonyl chloride (2.3 g, 11 mmol) according to the general procedure and obtained in 81 % yield as a white solid. ^1^H NMR [400 MHz, CDCl_3_]: *δ* = 3.82 (s, 3 H), 6.70 (br. s, 1 H), 6.81–6.96 (m, 2 H), 7.03–7.15 (m, 3 H), 7.18–7.31 (m, 2 H), 7.59–7.78 (m, 2 H) ppm. ^13^C NMR [101 MHz, CDCl_3_]: *δ* = 55.7, 114.3, 121.6, 125.2, 129.4, 129.5, 130.7, 136.9, 163.2 ppm. These data were in accordance to those reported in the literature.^17^

**4‐Nitro‐*N*‐phenylbenzenesulfonamide (S3):** Sulfonamide **S3** was prepared from aniline (0.9 g, 10 mmol) and 4‐nitrobenzenesulfonyl chloride (2.4 g, 11 mmol) according to the general procedure and obtained in 94 % yield as a light yellow solid. ^1^H NMR [400 MHz, CDCl_3_]: *δ* = 6.02 (br. s, 1 H), 7.07–7.32 (m, 5 H), 7.89–7.93 (m, 2 H), 8.22–8.30 (m, 2 H) ppm. ^13^C NMR [101 MHz, CDCl_3_]: *δ* = 122.4, 124.3, 126.6, 128.7, 129.9, 135.2, 144.5, 150.3 ppm. These data were in accordance to those reported in the literature.^18^

***N*‐Benzyl‐4‐methylbenzenesulfonamide (S4):** Sulfonamide **S4** was prepared from benzylamine (1.07 g, 10 mmol) and 4‐methylbenzenesulfonyl chloride (2.1 g, 11 mmol) according to the general procedure and obtained in 91 % yield as a white solid. ^1^H NMR [400 MHz, CDCl_3_]: *δ* = 2.32 (s, 3 H), 4.02 (d, *J* = 6.5 Hz, 2 H), 6.74 (br. t, *J* = 6.5 Hz, 1 H), 7.05–7.21 (m, 5 H), 7.27–7.31 (m, 2 H), 7.64–7.71 (m, 2 H) ppm. ^13^C NMR [101 MHz, CDCl_3_]: *δ* = 21.7, 48.3, 128.3, 128.4, 129.1, 129.5, 130.9, 139.1, 139.2, 144.3 ppm. These data were in accordance to those reported in the literature.^19^

***N*,4‐Dimethylbenzenesulfonamide (S5):** Sulfonamide **S5** was prepared from methylamine hydrochloride (0.68 g, 10 mmol) and 4‐methylbenzenesulfonyl chloride (2.1 g, 11 mmol) according to the general procedure and obtained in 89 % yield as brown oil. ^1^H NMR [400 MHz, CDCl_3_]: *δ* = 2.42 (s, 3 H), 2.62 (d, *J* = 5.5 Hz, 3 H), 4.45 (br. s, 1 H), 7.29–7.33 (m, 2 H), 7.70–7.76 (m, 2 H) ppm. ^13^C NMR [101 MHz, CDCl_3_]: *δ* = 21.2, 29.5, 127.3, 129.8, 135.7, 143.3 ppm. These data were in accordance to those reported in the literature.^20^

**General Procedure for the Synthesis of 1,2‐Dichlorovinyl Sulfonamides:** 1,2‐Dichlorovinyl sulfonamides **S6**–**S11** were prepared by the literature procedure reported by Anderson et al.^21^ The sulfonamide (8 mmol, 1.0 equiv.) was added dropwise at 0 °C to a suspension of sodium hydride (670 mg, 60 % dispersion in mineral oil, 16.8 mmol, 2.1 equiv.) in DMF (30 mL) and the reaction mixture was warmed to 23° in 2 h. Trichloroethene (800 µL, 8.8 mmol, 1.1 equiv.) was slowly added to this solution, which was after stirred at 50 °C for 16 h. After cooling to 23°, the reaction mixture was quenched with water (300 mL) and extracted with AcOEt (3 × 50 mL). The combined organic washings were dried with sodium sulfate, concentrated in vacuo and the crude residue was purified by column chromatography as indicated. The *E*‐configuration of the obtained products including the new compounds was assigned based on the ^1^H NMR spectra and X‐ray crystallography data known from the literature.^21^

***N*‐[(*E*)‐1,2‐Dichlorovinyl]‐*N*‐phenylbenzenesulfonamide (S6):** Column chromatography (heptane/AcOEt, 40:1 → 10:1) afforded product **S6** as a yellow oil (72 %). *R*_F_ (silica gel, heptane/AcOEt, 10:1): 0.34 (UV, KMnO_4_ solution). ^1^H NMR [400 MHz, CDCl_3_]: *δ* = 6.46 (s, 1 H), 7.30–7.39 (m, 5 H), 7.43–7.50 (m, 2 H), 7.58–7.63 (m, 1 H), 7.75–7.80 (m, 2 H) ppm. ^13^C NMR [101 MHz, CDCl_3_]: *δ* = 120.7, 128.6, 128.78, 128.79, 129.2, 129.4, 130.6, 133.6, 137.6, 138.5 ppm. FTIR**:** ν̃ = 811, 1089, 1169, 1363, 1489, 2934, 3086 cm^–1^. HRMS (ESI‐TOF) *m/z*: [M + H]^+^ calcd. for C_14_H_13_Cl_2_NO_2_S 327.9966, found 327.9954.

***N*‐[(*E*)‐1,2‐Dichlorovinyl]‐4‐methyl‐*N*‐phenylbenzenesulfonamide (S7):** Column chromatography (heptane/AcOEt, 40:1 → 10:1) afforded the product **S7** as a white solid (91 %). *R*_F_ (silica gel, heptane/AcOEt, 1:1): 0.70 (UV, KMnO_4_ solution). ^1^H NMR [400 MHz, CDCl_3_]: *δ* = 2.41 (s, 3 H), 6.46 (s, 1 H), 7.19–7.27 (m, 2 H), 7.30–7.39 (m, 5 H), 7.61–7.69 (m, 2 H) ppm. ^13^C NMR [101 MHz, CDCl_3_]: *δ* = 21.7, 120.5, 128.6, 128.8, 129.1, 129.3, 129.4, 130.7, 135.6, 137.7, 144.6 ppm. These data were in accordance to those reported in the literature.^22^

***N*‐[(*E*)‐1,2‐Dichlorovinyl]‐4‐methoxy‐*N*‐phenylbenzenesulfonamide (S8):** Column chromatography (heptane/AcOEt, 40:1 → 10:1) afforded the product **S8** as a yellow oil (87 %). *R*_F_ (silica gel, heptane/AcOEt, 7:1): 0.23 (UV, KMnO_4_ solution). ^1^H NMR [400 MHz, CDCl_3_]: *δ* = 3.86 (s, 3 H), 6.45 (s, 1 H), 6.86–6.98 (m, 2 H), 7.29–7.41 (m, 5 H), 7.65–7.73 (m, 2 H) ppm. ^13^C NMR [101 MHz, CDCl_3_]: *δ* = 55.6, 113.9, 120.4, 128.7, 129.0, 129.3, 130.0, 130.7, 130.9, 137.8, 163.6 ppm. FTIR: ν̃ = 809, 1089, 1160, 1261, 1361, 1489, 1594, 2945, 3085 cm^–1^. HRMS (ESI‐TOF) *m/z*: [M + H]^+^ calcd. for C_15_H_15_Cl_2_NO_3_S 358.0071, found 358.0092.

***N*‐[(*E*)‐1,2‐Dichlorovinyl]‐4‐nitro‐*N*‐phenylbenzenesulfonamide (S9):** Column chromatography (heptane/AcOEt, 20:1 → 2:1) afforded the product **S9** as a white solid (82 %). *R*_F_ (silica gel, heptane/AcOEt, 10:1): 0.11 (UV, KMnO_4_ solution). ^1^H NMR [400 MHz, CDCl_3_]: *δ* = 6.51 (s, 1 H), 7.31–7.49 (m, 5 H), 7.81–7.99 (m, 2 H), 8.21–8.40 (m, 2 H) ppm. ^13^C NMR [101 MHz, CDCl_3_]: *δ* = 121.3, 124.0, 128.7, 129.72, 129.77, 129.83, 129.84, 137.0, 144.1, 150.6 ppm. FTIR**:** ν̃ = 685, 738, 1094, 1171, 1348, 1530, 3082, 3102 cm^–1^. HRMS (ESI‐TOF) *m/z*: [M + H]^+^ calcd. for C_14_H_12_Cl_2_N_2_O_4_S 372.9817, found 372.9834.

***N*‐Benzyl‐*N*‐[(*E*)‐1,2‐dichlorovinyl]‐4‐methylbenzenesulfonamide (S10):** Column chromatography (heptane/AcOEt, 40:1 → 4:1) afforded the product **S10** as a white solid (73 %). *R*_F_ (silica gel, heptane/AcOEt, 10:1): 0.23 (UV, KMnO_4_ solution). ^1^H NMR [400 MHz, CDCl_3_]: *δ* = 2.47 (s, 3 H), 3.72–4.81 (br. s, 2 H), 6.27 (s, 1 H), 7.28–7.34 (m, 5 H), 7.32–7.37 (m, 2 H), 7.80–7.86 (m, 2 H) ppm. ^13^C NMR [101 MHz, CDCl_3_]: *δ* = 21.7, 52.0, 121.7, 128.23, 128.28, 129.3, 129.4, 129.8, 133.5, 135.3, 144.7 ppm. These data were in accordance to those reported in the literature.^21^

***N*‐[(*E*)‐1,2‐Dichlorovinyl]‐*N*,4‐dimethylbenzenesulfonamide (S11):** Column chromatography (heptane/ AcOEt 40:1 → 10:1) afforded the product **S11** as brown oil (64 %). *R*_F_ (silica gel, heptane/AcOEt, 10:1): 0.13 (UV, KMnO_4_ solution). ^1^H NMR [400 MHz, CDCl_3_]: *δ* = 2.45 (s, 3 H), 2.94 (s, 3 H), 6.39 (s, 1 H), 7.31–7.37 (m, 2 H), 7.79–7.85 (m, 2 H) ppm. ^13^C NMR [101 MHz, CDCl_3_]: *δ* = 21.7, 37.9, 121.4, 127.8, 129.2, 129.5, 132.4, 143.2 ppm. FTIR: ν̃ = 693, 722, 1084, 1160, 1333, 1498, 2986, 3012 cm^–1^. HRMS (ESI‐TOF) *m/z*: [M + H]^+^ calcd. for C_10_H_13_Cl_2_NO_2_S 279.9966, found 279.9983.

**General Procedure A for the Syntheses of Sulfonyl Ynamines:** Sulfonyl Ynamines **1a**, **1d**, **1e**, **1g**, **1h**, **1i**, **1j** and **1k** were prepared by the literature procedure reported by Davies et al.^23^ *n*‐Butyllithium (3.8 mL, 1.6 m in THF, 6 mmol, 2.1 equiv.) was slowly added to a stirred solution of 1,2‐dichlorovinyl sulfonamide (5 mmol, 1.0 equiv.) in THF (30 mL) under an argon atmosphere at –78 °C. After stirring for 1 h, the lithium acetylide was treated with the corresponding electrophile and stirred for 1 h at –78 °C. The mixture was warmed to 23 °C and stirred for 2–3 h. The reaction mixture was quenched with brine (100 mL) and extracted with Et_2_O (2 × 50 mL). The combined organic washings were dried with sodium sulfate, concentrated in vacuo and the crude residue was purified by column chromatography as indicated.

**General Procedure B for the Syntheses of Sulfonyl Ynamines:** Sulfonyl Ynamines **1b** and **1c** were prepared by the literature procedure reported by Wolf et al.^24^ Sulfonyl ynamine **1d** (1.0 g, 3.7 mmol, 1.0 equiv.), CuI (70 mg, 0.37 mmol, 0.1 equiv.) and *N*,*N*‐diisopropylethylamine (1.5 mL, 7.4 mmol, 2.0 equiv.) were dissolved in chloroform (20 mL) under a nitrogen atmosphere. After 30 min, the acyl chloride (5.6 mmol, 1.5 equiv.) was added, and the mixture was stirred until completion as determined by TLC. Solvent was removed in vacuo and the crude residue was purified by column chromatography as indicated.

**General Procedure C for the Syntheses of Sulfonyl Ynamines:** Sulfonyl Ynamine **1f** was prepared by the literature procedure reported by Hsung et al.^25^ Sulfonyl ynamine **1d** (1.0 g, 3.7 mmol, 1.0 equiv.), iodobenzene (830 mg, 4.1 mmol, 1.1 equiv.) and Pd(PPh_3_)_4_ (214 mg, 0.185 mmol, 0.05 equiv.) were dissolved in Et_3_N/toluene mixture (2:1, 36 mL) under a nitrogen atmosphere. The solution was stirred at 23 °C for 10 min, and CuI (11 mg, 0.06 mmol, 0.015 equiv.) was then added. After heating the reaction mixture at 60 °C for 12 h, the mixture was diluted with AcOEt, filtered through a diatomaceous earth pad, and concentrated in vacuo. The resulting crude residue was purified by silica gel flash column chromatography as indicated.

**Ethyl 3‐(4‐Methyl‐*N*‐phenylbenzenesulfonamido)propanoate (1a):** Sulfonyl ynamine **1a** was prepared according to general procedure A using 1,2‐dichlorovinyl sulfonamide **S7** (1.7 g, 5.0 mmol). The lithium acetylide was treated with freshly distilled ethyl chloroformate (714 µL, 7.5 mmol) at –78 °C for 15 min and at 23 °C for 2 h. Column chromatography (heptane/AcOEt, 40:1 → 10:1; silica gel was washed with 1 % Et_3_N in heptane before being used for column chromatography) afforded the product **1a** as a white solid (82 %). *R*_F_ (silica gel, heptane/AcOEt, 10:1): 0.11 (UV, KMnO_4_ solution). ^1^H NMR [400 MHz, CDCl_3_]: *δ* = 1.31 (t, *J* = 7.1 Hz, 3 H), 2.46 (s, 3 H), 4.24 (q, *J* = 7.1 Hz, 2 H), 7.16–7.23 (m, 2 H), 7.29–7.34 (m, 2 H), 7.34–7.38 (m, 3 H), 7.58–7.67 (m, 2 H) ppm. ^13^C NMR [101 MHz, CDCl_3_]: *δ* = 14.2, 21.8, 61.7, 66.6, 82.2, 126.5, 128.4, 129.3, 129.6, 130.0, 133.1, 137.5, 145.9, 154.8 ppm. These data were in accordance to those reported in the literature.^23^

***N*‐(4,4‐Dimethyl‐3‐oxopent‐1‐*yn*‐1‐yl)‐4‐methyl‐*N*‐phenylbenzenesulfon‐amide (1b):** Sulfonyl ynamine **1b** was prepared according to general procedure B. The reaction with pivaloyl chloride (690 µL, 6.6 mmol) was performed at 30 °C for 18 h. Column chromatography (toluene; silica gel was washed with 1 % Et_3_N in heptane before being used for column chromatography) afforded the product **1b** as a light yellow oil (79 %). *R*_F_ (silica gel, toluene): 0.32 (UV, KMnO_4_ solution). ^1^H NMR [400 MHz, CDCl_3_]: *δ* = 1.20 (s, 9 H), 2.40 (s, 3 H), 7.16–7.20 (m, 2 H), 7.24–7.28 (m, 2 H), 7.30–7.34 (m, 3 H), 7.53–7.59 (m, 2 H) ppm. ^13^C NMR [101 MHz, CDCl_3_]: *δ* = 21.6, 26.3, 44.5, 73.6, 89.1, 126.3, 128.3, 129.1, 129.3, 129.7, 133.2, 127.4, 145.6, 193.2 ppm. These data were in accordance to those reported in the literature.^24^

**4‐Methyl‐*N*‐(3‐oxo‐3‐phenylprop‐1‐*yn*‐1‐yl)‐*N*‐phenylbenzenesulfonamide (1c):** Sulfonyl ynamine **1c** was prepared according to general procedure B. The reaction with benzoyl chloride (767 µL, 6.6 mmol) was performed at 30 °C for 24 h. Column chromatography (heptane/AcOEt, 20:1 → 10:1; silica gel was washed with 1 % Et_3_N in heptane before being used for column chromatography) afforded the product **1c** as a light yellow oil (86 %). *R*_F_ (silica gel, heptane/AcOEt, 10:1): 0.33 (UV, KMnO_4_ solution). ^1^H NMR [400 MHz, CDCl_3_]: *δ* = 2.42 (s, 3 H), 7.25–7.29 (m, 4 H), 7.36–7.41 (m, 3 H), 7.47–7.55 (m, 2 H), 7.60–7.65 (m, 3 H), 8.14–8.26 (m, 2 H) ppm. ^13^C NMR [101 MHz, CDCl_3_]: *δ* = 21.7, 74.9, 90.2, 126.4, 128.2, 128.7, 129.2, 129.2, 129.5, 129.9, 132.9, 133.5, 136.9, 137.1, 145.9, 176.8 ppm. These data were in accordance to those reported in the literature.^24^

***N*‐Ethynyl‐4‐methyl‐*N*‐phenylbenzenesulfonamide (1d):** Sulfonyl ynamine **1d** was prepared according to general procedure A using 1,2‐dichlorovinyl sulfonamide **S7** (1.7 g, 5.0 mmol). The lithium acetylide was treated with water (10 mL) and the resulting mixture was stirred at 23 °C for 2 h. Column chromatography (heptane/AcOEt, 20:1 → 10:1; silica gel was washed with 1 % Et_3_N in heptane before being used for column chromatography) afforded the product **1d** as a light yellow oil (81 %). *R*_F_ (silica gel, heptane/AcOEt, 10:1): 0.18 (UV, KMnO_4_ solution). ^1^H NMR [400 MHz, CDCl_3_]: *δ* = 2.44 (s, 3 H), 2.83 (s, 1 H), 7.23–7.34 (m, 7 H), 7.56–7.61 (m, 2 H) ppm. ^13^C NMR [101 MHz, CDCl_3_]: *δ* = 21.7, 59.1, 75.8, 126.2, 128.2, 128.3, 129.0, 129.4, 132.7, 138.4, 145.0 ppm. These data were in accordance to those reported in the literature.^26^

**4‐Methyl‐*N*‐phenyl‐*N*‐[(trimethylsilyl)ethynyl]benzenesulfonamide (1e):** Sulfonyl ynamine **1e** was prepared according to general procedure A using 1,2‐dichlorovinyl sulfonamide **S7** (1.7 g, 5.0 mmol). The lithium acetylide was treated with trimethylsilyl chloride (952 µL, 7.5 mmol) at –78 °C for 15 min and at 23 °C for 2 h. Column chromatography (heptane/AcOEt, 20:1; silica gel was washed with 1 % Et_3_N in heptane before being used for column chromatography) afforded the product **1e** as a white solid (52 %). ***R*_F_** (silica gel, heptane/AcOEt, 10:1): 0.27 (UV, KMnO_4_ solution). ^1^H NMR [400 MHz, CDCl_3_]: *δ* = 0.17 (s, 9 H), 2.45 (s, 3 H), 7.21–7.25 (m, 2 H), 7.26–7.30 (m, 2 H), 7.30–7.36 (m, 3 H), 7.54–7.60 (m, 2 H) ppm. ^13^C NMR [101 MHz, CDCl_3_]: *δ* = 0.16, 21.9, 73.4, 93.2, 126.3, 128.3, 128.6, 129.2, 129.5, 133.0, 138.7, 145.1 ppm. FTIR**:** ν̃ = 752, 834, 902, 1056, 1168, 1372, 1476, 1592, 2137, 2161, 2887, 3034 cm^–1^. HRMS (ESI‐TOF) *m/z*: [M + H]^+^ calcd. for C_18_H_23_NO_2_SSi 344.1141, found 344.1134.

**4‐Methyl‐*N*‐phenyl‐*N*‐(phenylethynyl)benzenesulfonamide (1f):** Sulfonyl ynamine **1f** was prepared according to general procedure C. Column chromatography (heptane/AcOEt, 20:1 → 10:1; silica gel was washed with 1 % Et_3_N in heptane before being used for column chromatography) afforded the product **1f** as a light yellow solid (80 %). *R*_F_ (silica gel, heptane/AcOEt, 10:1): 0.27 (UV, KMnO_4_ solution). ^1^H NMR [400 MHz, CDCl_3_]: *δ* = 2.45 (s, 3 H), 7.27–7.36 (m, 9 H), 7.36–7.42 (m, 3 H), 7.61–7.65 (m, 2 H) ppm. ^13^C NMR [101 MHz, CDCl_3_]: *δ* = 21.7, 70.6, 83.2, 122.7, 126.5, 126.3, 128.1, 128.32, 128.34, 129.0, 129.5, 131.4, 132.9, 139.0, 145.1 ppm. These data were in accordance to those reported in the literature.^27^

**Ethyl 3‐(*N*‐Phenylbenzenesulfonamido)propanoate (1g):** Sulfonyl ynamine **1g** was prepared according to general procedure A using 1,2‐dichlorovinyl sulfonamide **S6** (1.6 g, 5.0 mmol). The lithium acetylide was treated with freshly distilled ethyl chloroformate (714 µL, 7.5 mmol) at –78 °C for 15 min and at 23 °C for 2 h. Column chromatography (toluene; silica gel was washed with 1 % Et_3_N in heptane before being used for column chromatography) afforded the product **1g** as a white solid (74 %). *R*_F_ (silica gel, toluene): 0.35 (UV, KMnO_4_ solution). ^1^H NMR [400 MHz, CDCl_3_]: *δ* = 1.31 (t, *J* = 7.1 Hz, 3 H), 4.24 (q, *J* = 7.1 Hz, 2 H), 7.14–7.22 (m, 2 H), 7.30–7.41 (m, 3 H), 7.49–7.58 (m, 2 H), 7.64–7.73 (m, 1 H), 7.74–7.80 (m, 2 H) ppm. ^13^C NMR [101 MHz, CDCl_3_]: *δ* = 14.2, 61.8, 66.5, 81.9, 126.6, 128.3, 129.27, 129.29, 129.5, 134.6, 135.8, 137.0, 154.0 ppm. FTIR**:** ν̃ = 685, 726, 1088, 1123, 1204, 1372, 1702, 2218, 2982 cm^–1^. HRMS (ESI‐TOF) *m/z*: [M + H]^+^ calcd. for C_17_H_17_NO_4_S 330.0800, found 330.0812.

**Ethyl 3‐(4‐Methoxy‐*N*‐phenylbenzenesulfonamido)propanoate (1h):** Sulfonyl ynamine **1h** was prepared according to general procedure A using 1,2‐dichlorovinyl sulfonamide **S8** (1.8 g, 5.0 mmol). The lithium acetylide was treated with freshly distilled ethyl chloroformate (714 µL, 7.5 mmol) at –78 °C for 15 min and at 23 °C for 2 h. Column chromatography (toluene; silica gel was washed with 1 % Et_3_N in heptane before being used for column chromatography) afforded the product **1h** as colorless oil (84 %). *R*_F_ (silica gel, toluene): 0.25 (UV, KMnO_4_ solution). ^1^H NMR [400 MHz, CDCl_3_]: *δ* = 1.30 (t, *J* = 7.1 Hz, 3 H), 3.88 (s, 3 H), 4.23 (q, *J* = 7.1 Hz, 2 H), 6.91–7.03 (m, 2 H), 7.15–7.24 (m, 2 H), 7.3–7.40 (m, 3 H), 7.64–7.73 (m, 2 H) ppm. ^13^C NMR [101 MHz, CDCl_3_]: *δ* = 14.2, 55.8, 61.7, 66.6, 82.5, 114.4, 126.4, 127.2, 129.2, 129.5, 130.6, 137.2, 154.1, 164.4 ppm. FTIR**:** ν̃ = 689, 1087, 1123, 1371, 1496, 1593, 1702, 2216, 2981 cm^–1^. HRMS (ESI‐TOF) *m/z*: [M + H]^+^ calcd. for C_18_H_19_NO_5_S 360.0906, found 360.0922.

**Ethyl 3‐(4‐Nitro‐*N*‐phenylbenzenesulfonamido)propanoate (1i) and Ethyl 3,3‐Bis(4‐nitro‐*N*‐phenyl‐benzenesulfonamido)acrylate (15):** Sulfonyl ynamine **1i** and compound **15** were prepared according to general procedure A using 1,2‐dichlorovinyl sulfonamide **S9** (1.9 g, 5.0 mmol). The lithium acetylide was treated with freshly distilled ethyl chloroformate (714 µL, 7.5 mmol) at –78 °C for 15 min and at 23 °C for 3 h. Column chromatography (toluene; silica gel was washed with 1 % Et_3_N in heptane before being used for column chromatography) afforded the product **1i** (42 %) and product **15** (41 %) as white solids. Product **15** was a common side product during the course of the reaction.^28^ Compound **1i**: R_F_ (silica gel, toluene): 0.33 (UV, KMnO_4_ solution). ^1^H NMR [400 MHz, CDCl_3_]: *δ* = 1.32 (t, *J* = 7.1 Hz, 3 H), 4.25 (q, *J* = 7.1 Hz, 2 H), 7.17–7.23 (m, 2 H), 7.36–7.46 (m, 3 H), 7.92–8.00 (m, 2 H), 8.33–8.42 (m, 2 H) ppm. ^13^C NMR [101 MHz, CDCl_3_]: *δ* = 14.1, 62.0, 66.7, 80.4, 124.5, 126.5, 129.7, 129.81, 129.84, 136.5, 140.9, 150.7, 153.6 ppm. FTIR**:** ν̃ = 690, 740, 854, 1086, 1126, 1184, 1206, 1348, 1533, 1593, 1706, 2223, 2925, 3107 cm^–1^. HRMS (ESI‐TOF) *m/z*: [M + H]^+^ calcd. for C_17_H_16_N_2_O_6_S 375.0651, found 375.0659. Compound **15**: R_F_ (silica gel, toluene): 0.07 (UV, KMnO_4_ solution). ^1^H NMR [400 MHz, CDCl_3_]: *δ* = 1.41 (t, *J* = 7.1 Hz, 3 H), 4.33 (q, *J* = 7.1 Hz, 2 H), 6.14 (s, 1 H), 7.11–7.16 (m, 2 H), 7.20–7.23 (m, 2 H), 7.24–7.27 (m, 2 H), 7.33–7.38 (m, 2 H), 7.40–7.45 (m, 2 H), 7.45–7.50 (m, 3 H), 7.52–7.57 (m, 1 H), 7.93–8.00 (m, 2 H), 8.09–8.18 (m, 2 H) ppm. ^13^C NMR [101 MHz, CDCl_3_]: *δ* = 14.1, 61.6, 110.0, 117.5, 123.4, 124.1, 129.4, 129.6, 129.7, 129.93, 129.97, 129.99, 130.0, 136.0, 136.3, 139.9, 143.4, 144.2, 149.9, 150.4, 163.9 ppm. FTIR**:** ν̃ = 694, 740, 855, 1086, 1139, 1172, 1202, 1350, 1531, 1720, 3106 cm^–1^. HRMS (ESI‐TOF) *m/z*: [M + H]^+^ calcd. for C_29_H_26_N_4_O_10_S_2_ 653.1012, found 653.1034.

**Ethyl 3‐(*N*,4‐Dimethylbenzenesulfonamido)propanoate (1j):** Sulfonyl ynamine **1j** was prepared according to general procedure A using 1,2‐dichlorovinyl sulfonamide **S11** (1.4 g, 5.0 mmol). The lithium acetylide was treated with freshly distilled ethyl chloroformate (714 µL, 7.5 mmol) at –78 °C for 15 min and at 23 °C for 2 h. Column chromatography (heptane/AcOEt, 20:1 → 10:1; silica gel was washed with 1 % Et_3_N in heptane before being used for column chromatography) afforded the product **1j** as a white solid (71 %). *R*_F_ (silica gel, heptane/AcOEt, 1:1): 0.6 (UV, KMnO_4_ solution). ^1^H NMR [400 MHz, CDCl_3_]: *δ* = 1.31 (t, *J* = 7.1 Hz, 3 H), 2.47 (s, 3 H), 3.17 (s, 3 H), 4.23 (q, *J* = 7.1 Hz, 2 H), 7.35–7.44 (m, 2 H), 7.78–7.92 (m, 2 H) ppm. ^13^C NMR [101 MHz, CDCl_3_]: *δ* = 14.2, 21.8, 38.5, 61.6, 66.0, 83.5, 128.0, 130.2, 133.0, 145.8, 154.1 ppm. These data were in accordance to those reported in the literature.^29^

**Ethyl 3‐(*N*‐Benzyl‐4‐methylbenzenesulfonamido)propanoate (1k):** Sulfonyl ynamine **1k** was prepared according to general procedure A using 1,2‐dichlorovinyl sulfonamide **S10** (1.8 g, 5.0 mmol). The lithium acetylide was treated with freshly distilled ethyl chloroformate (714 µL, 7.5 mmol) at –78 °C for 15 min and at 23 °C for 2 h. Column chromatography (heptane/AcOEt, 40:1 → 10:1; silica gel was washed with 1 % Et_3_N in heptane before being used for column chromatography) afforded the product **1k** as a white solid (87 %). *R*_F_ (silica gel, heptane/AcOEt, 10:1): 0.17 (UV, KMnO_4_ solution). ^1^H NMR [400 MHz, CDCl_3_]: *δ* = 1.28 (t, *J* = 7.1 Hz, 3 H), 2.44 (s, 3 H), 4.18 (q, *J* = 7.1 Hz, 2 H), 4.62 (s, 2 H), 7.24–7.33 (m, 7 H), 7.68–7.75 (m, 2 H) ppm. ^13^C NMR [101 MHz, CDCl_3_]: *δ* = 14.2, 21.6, 55.3, 61.4, 68.1, 82.5, 127.6, 128.5, 128.6, 129.3, 129.7, 133.5, 134.1, 145.4, 153.9 ppm. These data were in accordance to those reported in the literature.^22^

**Products of Palladium‐Catalyzed Intramolecular Hydroarylation of Sulfonyl Ynamine 1a:** Sulfonyl ynamine **1a** (343 mg, 1.0 mmol, 1.0 equiv.) was dissolved in toluene (10 mL) under a nitrogen atmosphere in a Biotage microwave vial (10.0–20.0 mL) equipped with a magnetic stirring bar. Pd(OAc)_2_ (11 mg, 0.05 mmol, 0.05 equiv.) and tri(*p*‐tolyl)phosphine (30 mg, 0.1 mmol, 0.1 equiv.) were added at 23 °C. The vial was covered with a Teflon septum and secured via a crimped aluminum cap. The reaction was irradiated in a Biotage Initiator microwave at 100 °C for 18 h (30 second pre‐stir, Fixed Hold Time On, Low absorbance level). The reaction mixture was quenched with brine (50 mL) and extracted with AcOEt (2 × 20 mL). The resulting organic washings were dried with sodium sulfate, concentrated in vacuo and the crude residue was purified by column chromatography (toluene; silica gel was washed with 1 % Et_3_N in heptane before being used for column chromatography).

**Ethyl (*E*)‐2‐[5‐Methyl‐1,1‐dioxo‐2‐phenyl‐1,2‐benzothiazol‐3(2*H*)‐ylidene]acetate [(*E*)‐2a]:** *R*_F_ (silica gel, toluene): 0.33 (UV, KMnO_4_ solution). ^1^H NMR [400 MHz, CDCl_3_]: *δ* = 1.26 (t, *J* = 7.1 Hz, 3 H), 2.57 (s, 3 H), 4.18 (q, *J* = 7.1 Hz, 2 H), 5.11 (s, 1 H), 7.47–7.51 (m, 2 H), 7.54–7.62 (m, 4 H), 7.82 (d, *J* = 7.9 Hz, 1 H), 9.23 (quint, *J* = 0.8 Hz, 1 H) ppm. ^13^C NMR [101 MHz, CDCl_3_]: *δ* = 14.2, 22.2, 60.5, 97.0, 120.9, 127.4, 129.8, 130.36, 130.39, 130.43, 130.5, 131.0, 133.1, 144.8, 146.2, 166.1 ppm. FTIR**:** ν̃ = 714, 902, 1089, 1174, 1302, 1512, 1635, 1737, 2992 cm^–1^. HRMS (ESI‐TOF) *m/z*: [M + H]^+^ calcd. for C_18_H_19_NO_4_S 344.0957, found 344.0966.

**Ethyl (*Z*)‐2‐[5‐Methyl‐1,1‐dioxo‐2‐phenyl‐1,2‐benzothiazol‐3(2*H*)‐ylidene]acetate [(*Z*)‐2a]:** *R*_F_ (silica gel, toluene): 0.28 (UV, KMnO_4_ solution). ^1^H NMR [400 MHz, CDCl_3_]: *δ* = 1.01 (t, *J* = 7.1 Hz, 3 H), 2.54 (s, 3 H), 3.66 (q, *J* = 7.1 Hz, 2 H), 5.83 (s, 1 H), 7.36–7.49 (m, 5 H), 7.55 (m, *J* = 8.0, 1.2, 0.6 Hz, 1 H), 7.62 (quint, *J* = 0.6 Hz, 1 H), 7.79 (d, *J* = 8.0 Hz, 1 H) ppm. ^13^C NMR [101 MHz, CDCl_3_]: *δ* = 13.9, 22.0, 60.4, 93.0, 121.5, 122.0, 126.9, 128.6, 128.9, 129.5, 130.2, 133.1, 134.9, 140.2, 144.9, 164.0 ppm. FTIR**:** ν̃ = 693, 907, 1082, 1145, 1266, 1327, 1494, 1635, 1708, 3029 cm^–1^. HRMS (ESI‐TOF) *m/z*: [M + H]^+^ calcd. for C_18_H_19_NO_4_S 344.0957, found 344.0943.

**4‐Methyl‐*N*‐Phenylbenzenesulfonamide (3):** The spectroscopic data were identical to those reported above.

**Ethyl 3,3‐Bis(4‐methyl‐*N*‐phenylbenzenesulfonamido)acrylate (4):** *R*_F_ (silica gel, toluene): 0.06 (UV, KMnO_4_ solution). ^1^H NMR [400 MHz, CDCl_3_]: *δ* = 1.39 (t, *J* = 7.1 Hz, 3 H), 2.30 (s, 3 H), 2.38 (s, 3 H), 4.33 (q, *J* = 7.1 Hz, 2 H), 6.09 (s, 1 H), 6.86–6.94 (m, 4 H), 7.06–7.12 (m, 2 H), 7.12–7.19 (m, 6 H), 7.26–7.33 (m, 2 H), 7.33–7.40 (m, 3 H), 7.42–7.48 (m, 1 H) ppm. ^13^C NMR [101 MHz, CDCl_3_]: *δ* = 14.1, 21.5, 21.6, 61.1, 115.5, 127.3, 128.18, 128.23, 128.93, 128.97, 129.0, 129.4, 129.5, 130.0, 130.2, 135.1, 136.0, 136.8, 137.1, 140.6, 143.5, 144.3, 164.7 ppm. FTIR**:** ν̃ = 695, 814, 1087, 1139, 1167, 1362, 1489, 1531, 1719, 2981 cm^–1^. HRMS (ESI‐TOF) *m/z*: [M + H]^+^ calcd. for C_31_H_32_N_2_O_6_S_2_ 591.1624, found 591.1638.

**Ethyl (*E*)‐3‐(4‐Methyl‐*N*‐phenylbenzenesulfonamido)acrylate (5):** *R*_F_ (silica gel, toluene): 0.12 (UV, KMnO_4_ solution). ^1^H NMR [400 MHz, CDCl_3_]: *δ* = 1.24 (t, *J* = 7.1 Hz, 3 H), 2.44 (s, 3 H), 4.14 (q, *J* = 7.1 Hz, 2 H), 4.62 (d, *J* = 13.8 Hz, 1 H), 6.88–6.97 (m, 2 H), 7.27–7.32 (m, 2 H), 7.35–7.45 (m, 3 H), 7.53–7.60 (m, 2 H), 8.37 (d, *J* = 13.7 Hz, 1 H) ppm. ^13^C NMR [101 MHz, CDCl_3_]: *δ* = 14.3, 21.7, 60.1, 100.0, 121.7, 127.8, 129.7, 129.86, 129.89, 134.8, 135.1, 143.9, 144.9, 167.1 ppm. FTIR**:** ν̃ = 727, 906, 1089, 1170, 1368, 1624, 1737, 2245, 2981 cm^–1^. HRMS (ESI‐TOF) *m/z*: [M + H]^+^ calcd. for C_18_H_21_NO_4_S 346.1113, found 346.1131.

**General Procedure for the Intramolecular Hydroarylation of Sulfonyl Ynamines 1a–c and 1g–k:** Sulfonyl ynamine **1** (1.0 mmol, 1.0 equiv.) was dissolved in toluene (10 mL) under a nitrogen atmosphere in a Biotage microwave vial (10.0–20.0 mL) equipped with a magnetic stirring bar. Pd(OAc)_2_ (11 mg, 0.05 mmol, 0.05 equiv.) and tri(*p*‐tolyl)phosphine (30 mg, 0.1 mmol, 0.1 equiv.) were added at 23 °C. The vial was covered with a Teflon septum and secured via a crimped aluminum cap. The reaction was irradiated in a Biotage Initiator microwave at 100 °C for 18 h (30 second pre‐stir, Fixed Hold Time On, Low absorbance level). The reaction mixture was quenched with brine (50 mL) and extracted with AcOEt (2 × 20 mL). The resulting organic washings were dried with sodium sulfate, concentrated in vacuo and the crude residue was purified by column chromatography as indicated.

**Ethyl (*E*)‐2‐[5‐Methyl‐1,1‐dioxo‐2‐phenyl‐1,2‐benzothiazol‐3(2*H*)‐ylidene]acetate [(*E*)‐2a]:** (*E*)‐1,2‐Benzothiazoledione **2a** was prepared according to general procedure using sulfonyl ynamine **1a** (343 mg, 1.0 mmol). Ratio *E/Z* = 98:2 was determined by ^1^H NMR spectroscopy. Column chromatography (toluene; silica gel was washed with 1 % Et_3_N in heptane before being used for column chromatography) afforded product (*E*)‐**2a** as a white solid (127 mg, 37 %). The spectroscopic data were identical to those reported above.

**(*E*)‐3,3‐Dimethyl‐1‐[5‐methyl‐1,1‐dioxo‐2‐phenyl‐1,2‐benzothiazol‐3(2*H*)‐ylidene]butan‐2‐one [(*E*)‐2b]:** (*E*)‐1,2‐Benzothiazoledione **2b** was prepared according to general procedure using sulfonyl ynamine **1b** (355 mg, 1.0 mmol). Ratio *E/Z* >, 99:1 was determined by ^1^H NMR spectroscopy. Column chromatography (toluene; silica gel was washed with 1 % Et_3_N in heptane before being used for column chromatography) afforded the product (*E*)‐**2b** as a white solid (121 mg, 34 %). *R*_F_ (silica gel, toluene): 0.28 (UV, KMnO_4_ solution). ^1^H NMR [400 MHz, CDCl_3_]: *δ* = 1.05 (s, 9 H), 2.57 (s, 3 H), 5.71 (s, 1 H), 7.48–7.53 (m, 2 H), 7.54–7.65 (m, 4 H), 7.82 (d, *J* = 7.9 Hz, 1 H), 9.10 (quint, *J* = 0.6 Hz, 1 H) ppm. ^13^C NMR [101 MHz, CDCl_3_]: *δ* = 22.3, 26.8, 44.7, 101.5, 121.0, 127.8, 129.4, 130.4, 130.5, 130.6, 130.8, 130.9, 133.4, 145.1, 145.6, 204.3 ppm. FTIR**:** ν̃ = 695, 966, 1078, 1183, 1322, 1566, 1673, 2965 cm^–1^. HRMS (ESI‐TOF) *m/z*: [M + H]^+^ calcd. for C_20_H_23_NO_3_S 356.1320, found 356.1343.

**(*E*)‐2‐[5‐methyl‐1,1‐dioxo‐2‐phenyl‐1,2‐benzothiazol‐3(2*H*)‐ylidene]‐1‐phenylethan‐1‐one [(*E*)‐2c]:** (*E*)‐1,2‐Benzothiazoledione **2c** was prepared according to general procedure using sulfonyl ynamine **1c** (375 mg, 1.0 mmol). Ratio *E/Z* = 98:2 was determined by ^1^H NMR spectroscopy. Column chromatography (toluene; silica gel was washed with 1 % Et_3_N in heptane before being used for column chromatography) afforded the product (*E*)‐**2c** as a white solid (116 mg, 31 %). *R*_F_ (silica gel, toluene): 0.31 (UV, KMnO_4_ solution). ^1^H NMR [400 MHz, CDCl_3_]: *δ* = 2.57 (s, 3 H), 6.09 (s, 1 H), 7.37–7.45 (m, 2 H), 7.48–7.55 (m, 1 H), 7.56–7.66 (m, 6 H), 7.72–7.79 (m, 2 H), 7.85 (d, *J* = 8.0 Hz, 1 H), 9.00 (quint, *J* = 0.5 Hz, 1 H) ppm. ^13^C NMR [101 MHz, CDCl_3_]: *δ* = 22.2, 102.4, 121.1, 127.4, 127.7, 128.2, 128.6, 129.1, 130.5, 130.6, 130.7, 130.9, 132.8, 133.6, 139.4, 145.1, 146.4, 189.7 ppm. FTIR: ν̃ = 696, 959, 1185, 1323, 1565, 1653, 1724, 2922, 3064 cm^–1^. HRMS (ESI‐TOF) *m/z*: [M + H]^+^ calcd. for C_22_H_19_NO_3_S 376.1007, found 376.1015.

**Ethyl (*E*)‐2‐[1,1‐Dioxo‐2‐phenyl‐1,2‐benzothiazol‐3(2*H*)‐ylidene]acetate [(*E*)‐2g]:** (*E*)‐1,2‐Benzothiazoledione **2g** was prepared according to general procedure using sulfonyl ynamine **1g** (330 mg, 1.0 mmol). Ratio *E/Z* = 98:2 was determined by ^1^H NMR spectroscopy. Column chromatography (toluene; silica gel was washed with 1 % Et_3_N in heptane before being used for column chromatography) afforded the product (*E*)‐**2g** as a white solid (112 mg, 34 %). *R*_F_ (silica gel, toluene): 0.31 (UV, KMnO_4_ solution). ^1^H NMR [400 MHz, CDCl_3_]: *δ* = 1.28 (t, *J* = 7.1 Hz, 3 H), 4.20 (q, *J* = 7.1 Hz, 2 H), 5.16 (s, 1 H), 7.46–7.56 (m, 2 H), 7.57–7.67 (m, 3 H), 7.76–7.85 (m, 2 H), 7.92–8.02 (m, 1 H), 9.44 (m, 1 H) ppm. ^13^C NMR [101 MHz, CDCl_3_]: *δ* = 14.4, 60.7, 97.6, 121.3, 127.4, 129.9, 130.5, 130.6, 130.7, 131.2, 132.5, 133.3, 133.9, 146.2, 166.3 ppm. FTIR**:** ν̃ = 692, 910, 1045, 1099, 1157, 1186, 1324, 1619, 1708, 2925 cm^–1^. HRMS (ESI‐TOF) *m/z*: [M + H]^+^ calcd. for C_17_H_17_NO_4_S 330.0800, found 330.0827.

**Ethyl (*E*)‐2‐[5‐Methoxy‐1,1‐dioxo‐2‐phenyl‐1,2‐benzothiazol‐3(2*H*)‐ylidene]acetate [(*E*)‐2h]:** (*E*)‐1,2‐Benzothiazoledione **2h** was prepared according to general procedure using sulfonyl ynamine **1h** (360 mg, 1.0 mmol). Ratio *E/Z* = 95:5 was determined by ^1^H NMR spectroscopy. Column chromatography (toluene; silica gel was washed with 1 % Et_3_N in heptane before being used for column chromatography) afforded the product (*E*)‐**2h** as a white solid (119 mg, 33 %). *R*_F_ (silica gel, toluene): 0.17 (UV, KMnO_4_ solution). ^1^H NMR [400 MHz, CDCl_3_]: *δ* = 1.25 (t, *J* = 7.1 Hz, 3 H), 3.98 (s, 3 H), 4.16 (q, *J* = 7.1 Hz, 2 H), 5.12 (s, 1 H), 7.25 (dd, *J* = 8.7, 2.2 Hz, 1 H), 7.47–7.52 (m, 2 H), 7.53–7.64 (m, 3 H), 7.83 (d, *J* = 8.6 Hz, 1 H), 9.14 (d, *J* = 2.3 Hz, 1 H) ppm. ^13^C NMR [101 MHz, CDCl_3_]: *δ* = 14.3, 56.1, 60.5, 97.2, 113.4, 119.7, 122.4, 125.1, 129.6, 130.43, 130.49, 130.53, 131.0, 146.3, 164.0, 166.2 ppm. FTIR**:** ν̃ = 1103, 1183, 1252, 1307, 1473, 1583, 1621, 1707, 2988, 3118 cm^–1^. HRMS (ESI‐TOF) *m/z*: [M + H]^+^ calcd. for C_18_H_19_NO_5_S 360.0906, found 360.0919.

**Ethyl (*E*)‐2‐[5‐Nitro‐1,1‐dioxo‐2‐phenyl‐1,2‐benzothiazol‐3(2*H*)‐ylidene]acetate [(*E*)‐2i]:** (*E*)‐1,2‐Benzothiazoledione **2i** was prepared according to general procedure using sulfonyl ynamine **1i** (375 mg, 1.0 mmol). Ratio *E/Z* >, 99:1 was determined by ^1^H NMR spectroscopy. Column chromatography (toluene; silica gel was washed with 1 % Et_3_N in heptane before being used for column chromatography) afforded the product (*E*)‐**2i** as a white solid (113 mg, 30 %). *R*_F_ (silica gel, toluene): 0.28 (UV, KMnO_4_ solution). ^1^H NMR [400 MHz, CDCl_3_]: *δ* = 1.28 (t, *J* = 7.1 Hz, 3 H), 4.24 (q, *J* = 7.1 Hz, 2 H), 5.27 (s, 1 H), 7.46–7.56 (m, 2 H), 7.58–7.69 (m, 3 H), 8.12 (d, *J* = 8.5 Hz, 1 H), 8.61 (dd, *J* = 8.5, 1.9 Hz, 1 H), 10.43 (d, *J* = 1.9 Hz, 1 H) ppm. ^13^C NMR [101 MHz, CDCl_3_]: *δ* = 14.2, 61.2, 99.9, 122.5, 125.6, 127.3, 129.1, 129.6, 130.7, 130.9, 131.1, 137.6, 143.8, 151.2, 165.6 ppm. FTIR**:** ν̃ = 694, 740, 1095, 1113, 1186, 1341, 1537, 1706, 2926, 3111 cm^–1^. HRMS (ESI‐TOF) *m/z*: [M + H]^+^ calcd. for C_17_H_16_N_2_O_6_S 375.0651, found 375.0667.

**Ethyl (*E*)‐2‐[2,5‐Dimethyl‐1,1‐dioxo‐1,2‐benzothiazol‐3(2*H*)‐ylidene]acetate [(*E*)‐2j]:** (*E*)‐1,2‐Benzothiazoledione **2j** was prepared according to general procedure using sulfonyl ynamine **1j** (281 mg, 1.0 mmol). Ratio *E/Z* = 95:5 was determined by ^1^H NMR spectroscopy. Column chromatography (toluene; silica gel was washed with 1 % Et_3_N in heptane before being used for column chromatography) afforded the product (*E*)‐**2j** as colorless oil (87 mg, 31 %). *R*_F_ (silica gel, toluene): 0.23 (UV, KMnO_4_ solution). ^1^H NMR [400 MHz, CDCl_3_]: *δ* = 1.35 (t, *J* = 7.1 Hz, 3 H), 2.54 (s, 3 H), 3.15 (s, 3 H), 4.25 (q, *J* = 7.1 Hz, 2 H), 5.33 (s, 1 H), 7.50 (d, *J* = 7.9 Hz, 1 H), 7.76 (d, *J* = 7.9 Hz, 1 H), 9.17 (s, 1 H) ppm. ^13^C NMR [101 MHz, CDCl_3_]: *δ* = 14.3, 22.2, 26.5, 60.5, 95.0, 120.8, 127.9, 129.8, 130.5, 132.7, 144.8, 144.9, 166.0 ppm. FTIR**:** ν̃ = 701, 823, 1039, 1137, 1159, 1315, 1619, 1708, 2982 cm^–1^. HRMS (ESI‐TOF) *m/z*: [M + H]^+^ calcd. for C_13_H_17_NO_4_S 282.0800, found 282.0819.

**Ethyl (*E*)‐2‐[2‐Benzyl‐5‐methyl‐1,1‐dioxo‐1,2‐benzothiazol‐3(2*H*)‐ylidene]acetate [(*E*)‐2k] and Ethyl (*E*)‐2‐[2‐(4‐Methylbenzenesulfonyl)isoindolin‐1‐ylidene]acetate (6):** (*E*)‐1,2‐Benzothiazoledione **2k** and compound **6** were prepared according to general procedure using sulfonyl ynamine **1k** (357 mg, 1.0 mmol). Ratio *E/Z* >, 99:1 was determined by ^1^H NMR spectroscopy. Column chromatography (toluene; silica gel was washed with 1 % Et_3_N in heptane before being used for column chromatography) afforded the product (*E*)‐**2k** as a white solid (93 mg, 26 %) and product **6** as a colorless oil (36 mg, 10 %). Compound (*E*)‐**2k**: R_F_ (silica gel, toluene): 0.30 (UV, KMnO_4_ solution). ^1^H NMR [400 MHz, CDCl_3_]: *δ* = 1.26 (t, *J* = 7.1 Hz, 3 H), 2.53 (s, 3 H), 4.15 (q, *J* = 7.1 Hz, 2 H), 4.84 (s, 2 H), 5.28 (s, 1 H), 7.27–7.33 (m, 1 H), 7.34–7.40 (m, 2 H), 7.40–7.44 (m, 2 H), 7.52 (dd, *J* = 7.9, 0.6 Hz, 1 H), 7.81 (d, *J* = 7.9 Hz, 1 H), 9.13 (quint, *J* = 0.6 Hz, 1 H) ppm. ^13^C NMR [101 MHz, CDCl_3_]: *δ* = 14.4, 22.4, 44.5, 60.6, 96.5, 121.0, 127.2, 127.8, 128.1, 129.1, 130.1, 130.4, 132.9, 134.1, 143.7, 145.1, 166.0 ppm. FTIR: ν̃ = 670, 732, 831, 1136, 1168, 1311, 1626, 1706, 2986, 3098 cm^–1^. HRMS (ESI‐TOF) *m/z*: [M + H]^+^ calcd. for C_19_H_21_NO_4_S 358.1113, found 358.1118. Compound **6**: R_F_ (silica gel, toluene): 0.21 (UV, KMnO_4_ solution). ^1^H NMR [400 MHz, CDCl_3_]: *δ* = 1.32 (t, *J* = 7.1 Hz, 3 H), 2.40 (s, 3 H), 4.19 (q, *J* = 7.1 Hz, 2 H), 5.02 (s, 2 H), 6.43 (s, 1 H), 7.28–7.39 (m, 4 H), 7.41–7.47 (m, 1 H), 7.76–7.83 (m, 2 H), 9.13 (d, *J* = 8.1 Hz, 1 H) ppm. ^13^C NMR [101 MHz, CDCl_3_]: *δ* = 14.6, 21.8, 55.4, 60.1, 96.7, 121.9, 127.7, 128.4, 128.9, 130.1, 131.3, 133.0, 134.6, 138.1, 145.1, 152.0, 167.0 ppm. FTIR**:** ν̃ = 667, 800, 1089, 1165, 1344, 1620, 1705, 2922 cm^–1^. HRMS (ESI‐TOF) *m/z*: [M + H]^+^ calcd. for C_19_H_21_NO_4_S 358.1113, found 358.1132.

**Intramolecular Hydroarylation of 1d, 1e and 1f:** Sulfonyl ynamines **1d**, **1e** or **1f** were submitted to the reaction conditions according to general procedure. ^1^H NMR spectroscopy of the crude product indicated partial degradation of the starting material to 4‐methyl‐*N*‐phenylbenzenesulfonamide **3** and additional unidentified products. The formation of products **2d**, **2e** or **2f** was not observed.

**Study of Equilibration Between *E*‐ and *Z*‐Isomers of 2a:** (*E*)‐1,2‐Benzothiazole‐1,1‐dione **2a** or (*Z*)‐1,2‐Benzothiazole‐1,1‐dione **2a** (69 mg, 0.2 mmol, 1.0 equiv.) were independently dissolved in toluene (2 mL) under a nitrogen atmosphere in a Biotage microwave vial (2.0–5.0 mL) equipped with a magnetic stirring bar. Pd(OAc)_2_ (2.2 mg, 0.01 mmol, 0.05 equiv.) and tri(*p*‐tolyl)phosphine (6.1 mg, 0.02 mmol, 0.1 equiv.) were added at 23 °C. The vial was covered with a Teflon septum and secured via a crimped aluminum cap. The reaction was irradiated in a Biotage Initiator microwave at 100 °C for 18 h (30 second pre‐stir, Fixed Hold Time On, Low absorbance level). The reaction mixture was quenched with brine (50 mL) and extracted with AcOEt (2 × 20 mL). The combined organic washings were dried with sodium sulfate, filtered off and concentrated in vacuo. ^1^H NMR spectroscopy of the crude product indicated a mixture of isomers with ratio *E/Z* = 85:15. Additionally, partial decomposition of the starting material was observed.

**General Procedure for Photochemical Rearrangement of 1,2‐Benzothiazole‐1,1‐diones:** To 25 mL quartz flask was added 1,2‐benzothiazole‐1,1‐dione **2a**, **2g**, **2h** or **2k** (0.1–1.0 mmol, 1.0 equiv.) in 10 mL of deoxygenated MeCN with stirring. This flask was irradiated in a Rayonet RMR‐600 photochemical reactor, using eight lamps of 300 nm of wavelength for 24 h with internal temperature of 50 °C. After cooling to 23 °C, the solvent was removed in vacuo and the crude residue was purified by column chromatography as indicated.

**Ethyl 5‐Methyl‐1,1‐dioxo‐3‐(phenylamino)‐1‐benzothiophene‐2‐carboxylate (12a):** 3‐Amino‐1‐benzothiophene‐1,1‐dione **12a** was prepared according to general procedure using 1,2‐benzothiazole‐1,1‐dione **2a** (343 mg, 1.0 mmol). Column chromatography (toluene; silica gel was washed with 1 % Et_3_N in heptane before being used for column chromatography) afforded the product **12a** as a white solid (316 mg, 92 %). *R*_F_ (silica gel, toluene): 0.22 (UV, KMnO_4_ solution). ^1^H NMR [400 MHz, CDCl_3_]: *δ* = 1.45 (t, *J *= 7.1 Hz, 3 H), 2.12 (s, 3 H), 4.43 (q, *J* = 7.1 Hz, 2 H), 6.40 (quint, *J* = 0.5 Hz, 1 H), 7.30–7.34 (m, 2 H), 7.39 (ddd, *J* = 7.8, 1.3, 0.7 Hz, 1 H), 7.44–7.54 (m, 3 H), 7.71 (d, *J* = 7.8 Hz, 1 H), 10.43 (br. s, 1 H) ppm. ^13^C NMR [101 MHz, CDCl_3_]: *δ* = 14.4, 21.7, 61.1, 100.1, 121.2, 125.9, 126.5, 127.0, 128.6, 129.8, 133.6, 137.4, 138.2, 142.8, 155.0, 164.5 ppm. FTIR**:** ν̃ = 704, 730, 1027, 1122, 1160, 1247, 1285, 1569, 1657, 2854, 2925 cm^–1^. HRMS (ESI‐TOF) *m/z*: [M + H]^+^ calcd. for C_18_H_19_NO_4_S 344.0957, found 344.0963.

**Ethyl 1,1‐Dioxo‐3‐(phenylamino)‐1‐benzothiophene‐2‐carboxylate (12b):** 3‐Amino‐1‐benzothiophene‐1,1‐dione **12b** was prepared according to general procedure using 1,2‐benzothiazoledione **2g** (33 mg, 0.1 mmol). Column chromatography (toluene; silica gel was washed with 1 % Et_3_N in heptane before being used for column chromatography) afforded the product **12b** as a white solid (31 mg, 96 %). *R*_F_ (silica gel, toluene): 0.24 (UV, KMnO_4_ solution). ^1^H NMR [400 MHz, CDCl_3_]: *δ* = 1.45 (t, *J *= 7.1 Hz, 3 H), 4.44 (q, *J* = 7.1 Hz, 2 H), 6.66 (d, *J *= 8.0 Hz, 1 H), 7.23–7.28 (m, 1 H), 7.29–7.36 (m, 2 H), 7.43–7.54 (m, 3 H), 7.58–7.62 (m, 1 H), 7.83 (d, *J* = 7.6 Hz, 1 H), 10.44 (br. s, 1 H) ppm. ^13^C NMR [101 MHz, CDCl_3_]: *δ* = 14.4, 61.3, 99.9, 121.6, 125.7, 125.9, 126.9, 128.7, 130.0, 132.1, 133.2, 137.4, 141.0, 154.8, 164.6 ppm. FTIR**:** ν̃ = 702, 763, 1161, 1246, 1284, 1434, 1561, 1610, 1657, 2854, 2925 cm^–1^. HRMS (ESI‐TOF) *m/z*: [M + H]^+^ calcd. for C_17_H_17_NO_4_S 330.0800, found 330.0813.

**Ethyl 5‐Methoxy‐1,1‐dioxo‐3‐(phenylamino)‐1‐benzothiophenecarboxylate (12c):** 3‐Amino‐1‐benzothiophene‐1,1‐dione **12c** was prepared according to general procedure using 1,2‐benzothiazole‐1,1‐dione **2h** (36 mg, 0.1 mmol). Column chromatography (toluene; silica gel was washed with 1 % Et_3_N in heptane before being used for column chromatography) afforded the product **12c** as a white solid (31 mg, 87 %). *R*_F_ (silica gel, toluene): 0.20 (UV, KMnO_4_ solution). ^1^H NMR [400 MHz, CDCl_3_]: *δ* = 1.45 (t, *J *= 7.1 Hz, 3 H), 3.48 (s, 3 H), 4.43 (q, *J* = 7.1 Hz, 2 H), 6.13 (d, *J *= 2.2 Hz, 2 H), 7.04 (dd, *J* = 8.5, 2.2 Hz, 1 H), 7.32–7.38 (m, 2 H), 7.44–7.53 (m, 3 H), 7.72 (d, *J* = 8.5 Hz, 1 H), 10.41 (br. s, 1 H) ppm. ^13^C NMR [101 MHz, CDCl_3_]: *δ* = 14.4, 55.4, 61.2, 100.7, 111.6, 118.3, 122.8, 127.2, 127.8, 128.8, 130.0, 132.7, 137.4, 154.4, 162.3, 164.5 ppm. FTIR: ν̃ = 703, 729, 910, 1021, 1162, 1245, 1567, 1655, 2926 cm^–1^. HRMS (ESI‐TOF) *m/z*: [M + H]^+^ calcd. for C_18_H_19_NO_5_S 360.0906, found 330.0927.

**Ethyl 3‐(Benzylamino)‐5‐methyl‐1,1‐dioxo‐1‐benzothiophene‐2‐carboxylate (12d):** 3‐Amino‐1‐benzothiophene‐1,1‐dione **12d** was prepared according to general procedure using 1,2‐benzothiazole‐1,1‐dione **2k** (36 mg, 0.1 mmol). Column chromatography (toluene; silica gel was washed with 1 % Et_3_N in heptane before being used for column chromatography) afforded the product **12d** as a white solid (34 mg, 95 %). *R*_F_ (silica gel, toluene): 0.25 (UV, KMnO_4_ solution). ^1^H NMR [400 MHz, CDCl_3_]: *δ* = 1.40 (t, *J *= 7.1 Hz, 3 H), 2.40 (s, 3 H), 4.36 (q, *J* = 7.1 Hz, 2 H), 5.02 (d, *J *= 5.9 Hz, 2 H), 7.33–7.40 (m, 3 H), 7.40–7.46 (m, 2 H), 7.49 (d, *J* = 7.8 Hz, 1 H), 7.59 (s, 1 H), 7.77 (d, *J* = 7.8 Hz, 1 H), 9.44 (br. s, 1 H) ppm. ^13^C NMR [101 MHz, CDCl_3_]: *δ* = 14.5, 22.2, 50.0, 61.0, 100.1, 121.2, 125.9, 126.5, 127.0, 128.6, 129.8, 133.6, 137.4, 138.2, 142.8, 154.9, 164.5 ppm. FTIR: ν̃ = 735, 786, 1141, 1216, 1268, 1577, 1659, 1738, 2925, 3254 cm^–1^. HRMS (ESI‐TOF) *m/z*: [M + H]^+^ calcd. for C_19_H_21_NO_4_S 358.1113, found 358.1133.

https://www.ccdc.cam.ac.uk/services/structures?id=doi:10.1002/ejoc.201801062 1828629 (for **2a**), 1828630 (for **12a**), and 1828631 (for **12d**) contain the supplementary crystallographic data for this paper. These data can be obtained free of charge from http://www.ccdc.cam.ac.uk/.

## Supporting information

Supporting Information
