# Supplementary material for: Synthesis of 3‐Amino‐1‐benzothiophene‐1,1‐diones by Alkyne Directed Hydroarylation and 1/N→3/C‐Sulfonyl Migration
Source: European J Org Chem. 2018 Oct 10;2018(39):5435–44. doi: 10.1002/ejoc.201801062 (PMC6220774; doi:10.1002/ejoc.201801062)
Supplement: Supplementary file 1 — Supporting Information [file EJOC-2018-5435-s001.pdf]

EurJOC
European Journal of Organic Chemistry

Supporting Information

Synthesis of 3-Amino-1-benzothiophene-1,1-diones by Alkyne Directed Hydroarylation and 1/N→3/C-Sulfonyl Migration

Ivan Bernar, Daniel Blanco-Ania, Sophie J. Stok, Lia Sotorríos,
Enrique Gómez-Bengoa,* and Floris P. J. T. Rutjes*

ejoc201801062-sup-0001-SupMat.pdf

Content

Experimental Section 2

Synthesis of Sulfonamides **3** and **S1**–**S5** 3

Synthesis of 1,2-Dichlorovinyl Sulfonamides **S6**–**S11** 4

Synthesis of Sulfonyl Ynamines **1a**–**k** 6

Optimization Studies for Intramolecular Hydroarylation of Sulfonyl Ynamine **1a** 11

Intramolecular Hydroarylation of Sulfonyl Ynamine **1a**–**c** and **1g**–**k** 14

Intramolecular Hydroarylation of **1d**, **1e** and **1f** 18

Intramolecular Hydroarylation of Ethyl Acrylate **5** 18

Study of Equilibration between *E*- and *Z*-isomers of **2a** 18

Photochemical Rearrangement of 1,2-Benzothiazole-1,1-diones 20

X-Ray Crystallography of (*E*)-**2a**, **12a** and **12d** 22

References and Notes 24

Experimental Section

Reagents were obtained from commercial suppliers and were used without purification. Standard syringe techniques were applied for the transfer of dry solvents and air- or moisture-sensitive reagents. All inert reactions were carried out under a nitrogen atmosphere using flame-dried flasks. If stated, reactions were performed in Biotage Initiator+ Microwave Synthesizer under a nitrogen atmosphere. ^{1}H and ^{13}C NMR spectra were recorded at 298 K on a Varian Inova 400 MHz or Bruker 500 MHz spectrometer in the solvent indicated. Chemical shifts are given in parts per million (ppm) with respect to tetramethylsilane (0.00 ppm) as internal standard for ^{1}H NMR; and $CDCl_3$ (77.16 ppm) as internal standard for ^{13}C NMR. Coupling constants are reported as *J* values in hertz (Hz). ^{1}H NMR data are reported as follows: chemical shift (ppm), multiplicity (s = singlet, d = doublet, quint = quintet, t = triplet and combination of them), coupling constants (Hz) and integration. All NMR signals were assigned on the basis of ^{1}H NMR, ^{13}C NMR, gCOSY, gHSQC, gHMBS and NOESY experiments. Mass spectra were recorded on a JEOL AccuTOF CS JMST100CS (ESI) mass spectrometer. Automatic flash column chromatography was performed using Biotage Isolera Spektra One, using SNAP cartridges (Biotage, 30–100 µm, 60 Å), 10–50 g. Analysis by TLC was conducted on Silica gel F254 (Merck KGaA) plates with detection by UV absorption (254 nm) where applicable, and by dipping into a solution of aqueous $KMnO_4/Na_2CO_3/NaOH$ solution followed by charring at ca. 150 °C. IR spectra were recorded on a Bruker Tensor 27 FTIR spectrometer.

Synthesis of Sulfonamides 3 and S1–S5

General procedure:[1] The sulfonyl chloride (11 mmol, 1.1 equiv) was added to a solution of the amine (10 mmol, 1 equiv) and pyridine (950 mg, 12 mmol, 1.2 equiv) in CH_2Cl_2 (30 mL) portion-wise with stirring. The reaction mixture was then stirred at 23 °C for 12 h before evaporation of CH_2Cl_2 and quenching with an aqueous NaOH solution (2 N, 100 mL). The aqueous solution was rinsed with diethyl ether (2 × 50 mL) then acidified with concentrated HCl and extracted with CH_2Cl_2 (3 × 50 mL). The combined organic washings were dried over sodium sulfate and concentrated in vacuo. The obtained crude product was used directly in the next step without further purification.

***N*-Phenylbenzenesulfonamide S1.**

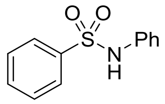
S1

Sulfonamide **S1** was prepared from aniline (0.9 g, 10 mmol) and benzenesulfonyl chloride (1.9 g, 11 mmol) according to the general procedure and obtained in 90% yield as a white solid. **^{1}H NMR** [400 MHz, δ (ppm), $CDCl_3$]: 6.58 (br s, 1 H), 7.02–7.09 (m, 2 H), 7.09–7.18 (m, 1 H), 7.20–7.29 (m, 2 H), 7.40–7.48 (m, 2 H), 7.50–7.58 (m, 1 H), 7.72–7.79 (m, 2 H). **^{13}C NMR** [101 MHz, δ (ppm), $CDCl_3$]: 121.6, 125.4, 127.3, 129.3, 129.7, 133.0, 136.5, 139.0. These data were in accordance to those reported in the literature.[2]

4-Methyl-*N*-phenylbenzenesulfonamide 3.

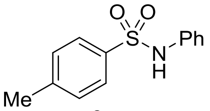
3

Sulfonamide **3** was prepared from aniline (0.9 g, 10 mmol) and 4-methylbenzenesulfonyl chloride (2.1 g, 11 mmol) according to the general procedure and obtained in 92% yield as a white solid. **^{1}H NMR** [400 MHz, δ (ppm), $CDCl_3$]: 2.35 (s, 3 H), 6.61 (br s, 1 H), 7.02–7.10 (m, 2 H), 7.12–7.25 (m, 5 H), 7.62–7.68 (m, 2 H). **^{13}C NMR** [101 MHz, δ (ppm), $CDCl_3$]: 21.6, 121.5, 125.1, 127.5, 129.2, 129.6, 136.0, 136.4, 143.9. These data were in accordance to those reported in the literature.[2]

4-Methoxy-*N*-phenylbenzenesulfonamide S2.

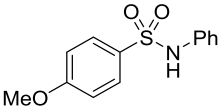
S2

Sulfonamide **S2** was prepared from aniline (0.9 g, 10 mmol) and 4-methoxybenzenesulfonyl chloride (2.3 g, 11 mmol) according to the general procedure and obtained in 81% yield as a white solid. **^{1}H NMR** [400 MHz, δ (ppm), $CDCl_3$]: 3.82 (s, 3 H), 6.70 (br s, 1 H), 6.81–6.96 (m, 2 H), 7.03–7.15 (m, 3 H), 7.18–7.31 (m, 2 H), 7.59–7.78 (m, 2 H). **^{13}C NMR** [101 MHz, δ (ppm), $CDCl_3$]: 55.7, 114.3, 121.6, 125.2, 129.4, 129.5, 130.7, 136.9, 163.2. These data were in accordance to those reported in the literature.[2]

4-Nitro-*N*-phenylbenzenesulfonamide S3.

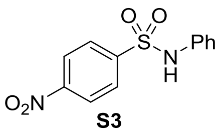
S3

Sulfonamide **S3** was prepared from aniline (0.9 g, 10 mmol) and 4-nitrobenzenesulfonyl chloride (2.4 g, 11 mmol) according to the general procedure and obtained in 94% yield as a light yellow solid. **^{1}H NMR** [400 MHz, δ (ppm),

$CDCl_3$]: 6.02 (br s, 1 H), 7.07–7.32 (m, 5 H), 7.89–7.93 (m, 2 H), 8.22–8.30 (m, 2 H). ^{13}**C NMR** [101 MHz, δ (ppm), $CDCl_3$]: 122.4, 124.3, 126.6, 128.7, 129.9, 135.2, 144.5, 150.3. These data were in accordance to those reported in the literature.[3]

***N*-Benzyl-4-methylbenzenesulfonamide S4.**

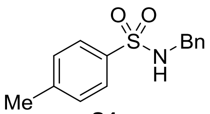

S4

Sulfonamide **S4** was prepared from benzylamine (1.07 g, 10 mmol) and 4-methylbenzenesulfonyl chloride (2.1 g, 11 mmol) according to the general procedure and obtained in 91% yield as a white solid. ^{1}**H NMR** [400 MHz, δ (ppm), $CDCl_3$]: 2.32 (s, 3 H), 4.02 (d, J = 6.5 Hz, 2 H), 6.74 (br t, J = 6.5 Hz, 1 H), 7.05–7.21 (m, 5 H), 7.27–7.31 (m, 2 H), 7.64–7.71 (m, 2 H). ^{13}**C NMR** [101 MHz, δ (ppm), $CDCl_3$]: 21.7, 48.3, 128.3, 128.4, 129.1, 129.5, 130.9, 139.1, 139.2, 144.3. These data were in accordance to those reported in the literature.[4]

***N*,4-Dimethylbenzenesulfonamide S5.**

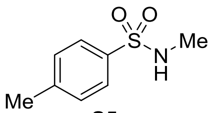

S5

Sulfonamide **S5** was prepared from methylamine hydrochloride (0.68 g, 10 mmol) and 4-methylbenzenesulfonyl chloride (2.1 g, 11 mmol) according to the general procedure and obtained in 89% yield as brown oil. ^{1}**H NMR** [400 MHz, δ (ppm), $CDCl_3$]: 2.42 (s, 3 H), 2.62 (d, J = 5.5 Hz, 3 H), 4.45 (br s, 1 H), 7.29–7.33 (m, 2 H), 7.70–7.76 (m, 2 H). ^{13}**C NMR** [101 MHz, δ (ppm), $CDCl_3$]: 21.2, 29.5, 127.3, 129.8, 135.7, 143.3. These data were in accordance to those reported in the literature.[5]

Synthesis of 1,2-Dichlorovinyl Sulfonamides S6–S11

General procedure: The sulfonamide (8 mmol, 1.0 equiv) was added dropwise at 0 °C to a suspension of sodium hydride (670 mg, 60% dispersion in mineral oil, 16.8 mmol, 2.1 equiv) in DMF (30 mL) and the reaction mixture was allowed to warm to 23 °C in 2 h. Trichloroethene (800 µL, 8.8 mmol, 1.1 equiv) was slowly added to this solution, which was after stirred at 50 °C for 16 h. After cooling to 23 °C, the reaction mixture was quenched with water (300 mL) and extracted with AcOEt (3 × 50 mL). The combined organic washings were dried over sodium sulfate, concentrated in vacuo and the crude residue was purified by column chromatography as indicated. The *E*-configuration of the obtained products including the new compounds was assigned based on the ^{1}H NMR spectra and X-ray crystallography data known from the literature.[6]

***N*-[(*E*)-1,2-Dichlorovinyl]-*N*-phenylbenzenesulfonamide S6**.

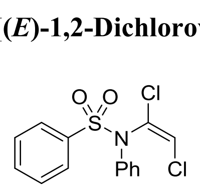

S6

Column chromatography (heptane/ AcOEt 40:1 → 10:1) afforded product **S6** as a yellow oil (72%). $\boldsymbol{R}_\mathbf{F}$ (silica gel, heptane/AcOEt 10:1): 0.34 (UV, $KMnO_4$ solution). ^{1}**H NMR** [400 MHz, δ (ppm), $CDCl_3$]: 6.46 (s, 1 H), 7.30–7.39 (m, 5 H), 7.43–7.50 (m, 2 H), 7.58–7.63 (m, 1 H), 7.75–7.80 (m, 2 H). ^{13}**C NMR** [101 MHz, δ (ppm), $CDCl_3$]:

120.7, 128.6, 128.78, 128.79, 129.2, 129.4, 130.6, 133.6, 137.6, 138.5. **FTIR** [$\bar{\nu}$ (cm^{-1})]: 811, 1089, 1169, 1363, 1489, 2934, 3086. **HRMS** (ESI^+) calcd. for $[C_{14}H_{12}Cl_2NO_2S + H]^+$ 327.9966, found 327.9954.

***N*-[(*E*)-1,2-Dichlorovinyl]-4-methyl-*N*-phenylbenzenesulfonamide S7**.

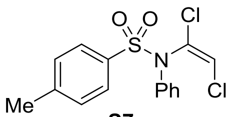

S7

Column chromatography (heptane/AcOEt 40:1 → 10:1) afforded the product **S7** as a white solid (91%). ***R*** R_F (silica gel, heptane/AcOEt 1:1): 0.70 (UV, $KMnO_4$ solution). **^{1}H NMR** [400 MHz, δ (ppm), $CDCl_3$]: 2.41 (s, 3 H), 6.46 (s, 1 H), 7.19–7.27 (m, 2 H), 7.30–7.39 (m, 5 H), 7.61–7.69 (m, 2 H). **^{13}C NMR** [101 MHz, δ (ppm), $CDCl_3$]: 21.7, 120.5, 128.6, 128.8, 129.1, 129.3, 129.4, 130.7, 135.6, 137.7, 144.6. These data were in accordance to those reported in the literature.[7]

***N*-[(*E*)-1,2-Dichlorovinyl]-4-methoxy-*N*-phenylbenzenesulfonamide S8**.

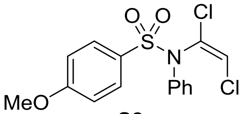

S8

Column chromatography (heptane/AcOEt 40:1 → 10:1) afforded the product **S8** as a yellow oil (87%). **R_F** (silica gel, heptane/AcOEt 7:1): 0.23 (UV, $KMnO_4$ solution). **^{1}H NMR** [400 MHz, δ (ppm), $CDCl_3$]: 3.86 (s, 3 H), 6.45 (s, 1 H), 6.86–6.98 (m, 2 H), 7.29–7.41 (m, 5 H), 7.65–7.73 (m, 2 H). **^{13}C NMR** [101 MHz, δ (ppm), $CDCl_3$]: 55.6, 113.9, 120.4, 128.7, 129.0, 129.3, 130.0, 130.7, 130.9, 137.8, 163.6. **FTIR** [$\bar{\nu}$ (cm^{-1})]: 809, 1089, 1160, 1261, 1361, 1489, 1594, 2945, 3085. **HRMS** (ESI^+) calcd. for $[C_{15}H_{14}Cl_2NO_3S + H]^+$ 358.0071, found 358.0092.

***N*-[(*E*)-1,2-Dichlorovinyl]-4-nitro-*N*-phenylbenzenesulfonamide S9**.

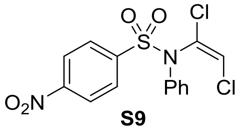

S9

Column chromatography (heptane/AcOEt 20:1 → 2:1) afforded the product **S9** as a white solid (82%). **R_F** (silica gel, heptane/AcOEt 10:1): 0.11 (UV, $KMnO_4$ solution). **^{1}H NMR** [400 MHz, δ (ppm), $CDCl_3$]: 6.51 (s, 1 H), 7.31–7.49 (m, 5 H), 7.81–7.99 (m, 2 H), 8.21–8.40 (m, 2 H). **^{13}C NMR** [101 MHz, δ (ppm), $CDCl_3$]: 121.3, 124.0, 128.7, 129.72, 129.77, 129.83, 129.84, 137.0, 144.1, 150.6. **FTIR** [$\bar{\nu}$ (cm^{-1})]: 685, 738, 1094, 1171, 1348, 1530, 3082, 3102. **HRMS** (ESI^+) calcd. for $[C_{14}H_{11}Cl_2N_2O_4S + H]^+$ 372.9817, found 372.9834.

***N*-Benzyl-*N*-[(*E*)-1,2-dichlorovinyl]-4-methylbenzenesulfonamide S10**.

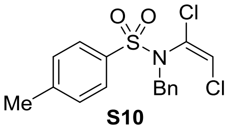

S10

Column chromatography (heptane/AcOEt 40:1 → 4:1) afforded the product **S10** as a white solid (73%). **R_F** (silica gel, heptane/AcOEt 10:1): 0.23 (UV, $KMnO_4$ solution). **^{1}H NMR** [400 MHz, δ (ppm), $CDCl_3$]: 2.47 (s, 3 H), 3.72–4.81 (br s, 2 H), 6.27 (s, 1 H), 7.28–7.34 (m, 5 H), 7.32–7.37 (m, 2 H), 7.80–7.86 (m, 2 H). **^{13}C NMR** [101 MHz, δ (ppm), $CDCl_3$]: 21.7, 52.0, 121.7, 128.23, 128.28, 129.3, 129.4, 129.8, 133.5, 135.3, 144.7. These data were in accordance to those reported in the literature.[6]

***N*-[(*E*)-1,2-Dichlorovinyl]-*N*,4-dimethylbenzenesulfonamide S11**.

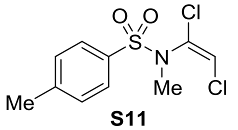

S11

Column chromatography (heptane/ AcOEt 40:1 → 10:1) afforded the product **S11** as brown oil (64%). ***R*$_F$** (silica gel, heptane/AcOEt 10:1): 0.13 (UV, $KMnO_4$ solution). **^{1}H NMR** [400 MHz, δ (ppm), $CDCl_3$]: 2.45 (s, 3 H), 2.94 (s, 3 H), 6.39 (s, 1 H), 7.31–7.37 (m, 2 H), 7.79–7.85 (m, 2 H). **^{13}C NMR** [101 MHz, δ (ppm), $CDCl_3$]: 21.7, 37.9, 121.4, 127.8, 129.2, 129.5, 132.4, 143.2. **FTIR** [$\bar{\nu}$ (cm^{-1})]: 693, 722, 1084, 1160, 1333, 1498, 2986, 3012. **HRMS** (ESI^+) calcd. for $[C_{10}H_{12}Cl_2NO_2S + H]^+$ 279.9966, found 279.9983.

Synthesis of Sulfonyl Ynamines 1a–k

Scheme 1. Sulfonyl Ynamines **1a**–**k** Used in the Study

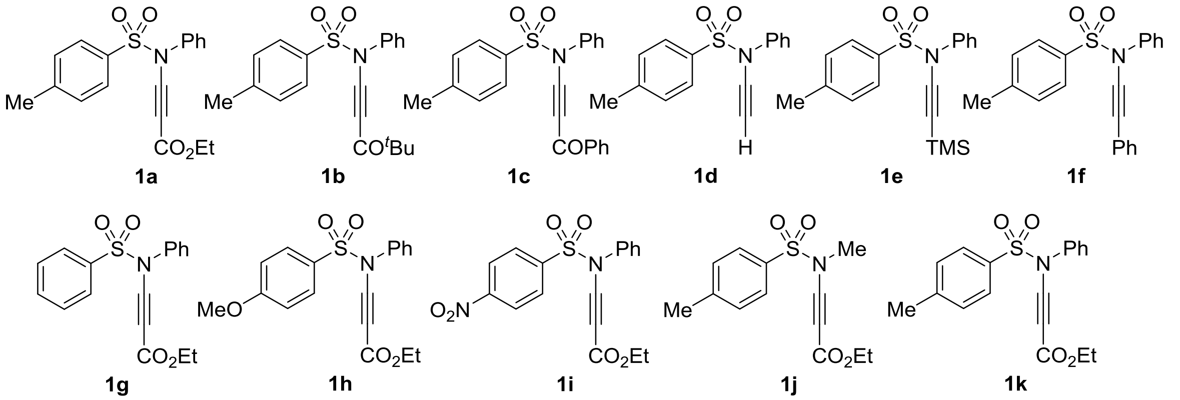

Sulfonyl ynamines **1a**, **1d**, **1e**, **1g**, **1h**, **1i**, **1j** and **1k** were prepared according to the general procedure A, **1b** and **1c** were prepared from **1d** according to the general procedure B, and **1f** was prepared from **1d** according to the general procedure C.

General procedure A:[8] ^{n}Butyllithium (3.8 mL, 1.6 M in THF, 6 mmol, 2.1 equiv) was slowly added to a stirred solution of 1,2-dichlorovinyl sulfonamide (5 mmol, 1.0 equiv) in THF (30 mL) under an argon atmosphere at −78 °C. After stirring for 1 h, the lithium acetylide was treated with the corresponding electrophile and stirred for 1 h at −78 °C. The mixture was allowed to warm to 23 °C and stirred for 2–3 h. The reaction mixture was quenched with brine (100 mL) and extracted with Et_2O (2 × 50 mL). The combined organic washings were dried over sodium sulfate, concentrated in vacuo and the crude residue was purified by column chromatography as indicated.

General procedure B:[9] Sulfonyl ynamine **1d** (1.0 g, 3.7 mmol, 1.0 equiv), CuI (70 mg, 0.37 mmol, 0.1 equiv) and *N*,*N*-diisopropylethylamine (1.5 mL, 7.4 mmol, 2.0 equiv) were dissolved in chloroform (20 mL) under a nitrogen atmosphere. After 30 min, the acyl chloride (5.6 mmol, 1.5 equiv) was added, and the mixture was stirred until completion as determined by TLC. Solvent was removed in vacuo and the crude residue was purified by column chromatography as indicated.

General procedure C:[10] Sulfonyl ynamine **1d** (1.0 g, 3.7 mmol, 1.0 equiv), iodobenzene (830 mg, 4.1 mmol, 1.1 equiv) and $Pd(PPh_3)_4$ (214 mg, 0.185 mmol, 0.05 equiv) were dissolved in Et_3N/toluene mixture (2:1, 36 mL) under a nitrogen atmosphere. The solution was stirred at 23 °C for 10 min, and CuI (11 mg, 0.06 mmol, 0.015 equiv) was then added. After heating the reaction mixture at 60 °C for 12 h, the mixture was diluted with AcOEt, filtered through a diatomaceous earth pad, and concentrated in vacuo. The resulting crude residue was purified by silica gel flash column chromatography as indicated.

Ethyl 3-(4-methyl-*N*-phenylbenzenesulfonamido]propanoate 1a.

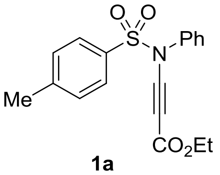

1a

Sulfonyl ynamine **1a** was prepared according to general procedure A using 1,2-dichlorovinyl sulfonamide **S7** (1.7 g, 5.0 mmol). The lithium acetylide was treated with freshly distilled ethyl chloroformate (714 µL, 7.5 mmol) at −78 °C for 15 min and at 23 °C for 2 h. Column chromatography (heptane/AcOEt 40:1 → 10:1; silica gel was washed with 1% Et_3N in heptane before being used for column chromatography) afforded the product **1a** as a white solid (82%). $\boldsymbol{R_F}$ (silica gel, heptane/AcOEt 10:1): 0.11 (UV, $KMnO_4$ solution). **^{1}H NMR** [400 MHz, δ (ppm), $CDCl_3$]: 1.31 (t, *J* = 7.1 Hz, 3 H), 2.46 (s, 3 H), 4.24 (q, *J* = 7.1 Hz, 2 H), 7.16–7.23 (m, 2 H), 7.29–7.34 (m, 2 H), 7.34–7.38 (m, 3 H), 7.58–7.67 (m, 2 H). **^{13}C NMR** [101 MHz, δ (ppm), $CDCl_3$]: 14.2, 21.8, 61.7, 66.6, 82.2, 126.5, 128.4, 129.3, 129.6, 130.0, 133.1, 137.5, 145.9, 154.8. These data were in accordance to those reported in the literature.[8]

***N*-(4,4-Dimethyl-3-oxopent-1-yn-1-yl)-4-methyl-*N*-phenylbenzenesulfonamide 1b.**

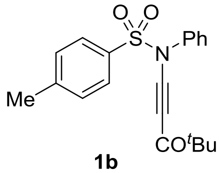

1b

Sulfonyl ynamine **1b** was prepared according to general procedure B. The reaction with pivaloyl chloride (690 µL, 6.6 mmol) was performed at 30 °C for 18 h. Column chromatography (toluene; silica gel was washed with 1% Et_3N in heptane before being used for column chromatography) afforded the product **1b** as a light yellow oil (79%). $\boldsymbol{R_F}$ (silica gel, toluene): 0.32 (UV, $KMnO_4$ solution). **^{1}H NMR** [400 MHz, δ (ppm), $CDCl_3$]: 1.20 (s, 9 H), 2.40 (s, 3 H), 7.16–7.20 (m, 2 H), 7.24–7.28 (m, 2 H), 7.30–7.34 (m, 3 H), 7.53–7.59 (m, 2 H). **^{13}C NMR** [101 MHz, δ (ppm), $CDCl_3$]: 21.6, 26.3, 44.5, 73.6, 89.1, 126.3, 128.3, 129.1, 129.3, 129.7, 133.2, 127.4, 145.6, 193.2. These data were in accordance to those reported in the literature.[9]

4-Methyl-*N*-(3-oxo-3-phenylprop-1-yn-1-yl)-*N*-phenylbenzenesulfonamide 1c.

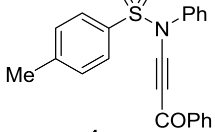

1c

Sulfonyl ynamine **1c** was prepared according to general procedure B. The reaction with benzoyl chloride (767 µL, 6.6 mmol) was performed at 30 °C for 24 h. Column chromatography (heptane/AcOEt 20:1 → 10:1; silica gel was washed with 1% Et_3N in heptane before being used for column chromatography) afforded the product **1c** as a light yellow oil (86%). $\boldsymbol{R_F}$ (silica gel, heptane/AcOEt 10:1): 0.33 (UV, $KMnO_4$ solution). **^{1}H NMR** [400 MHz, δ (ppm), $CDCl_3$]: 2.42 (s, 3 H), 7.25–7.29 (m, 4 H), 7.36–7.41

(m, 3 H), 7.47–7.55 (m, 2 H), 7.60–7.65 (m, 3 H), 8.14–8.26 (m, 2 H). ^{13}**C NMR** [101 MHz, δ (ppm), $CDCl_3$]: 21.7, 74.9, 90.2, 126.4, 128.2, 128.7, 129.2, 129.2, 129.5, 129.9, 132.9, 133.5, 136.9, 137.1, 145.9, 176.8. These data were in accordance to those reported in the literature.[9]

***N*-Ethynyl-4-methyl-*N*-phenylbenzenesulfonamide 1d.**

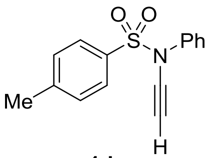

1d

Sulfonyl ynamine **1d** was prepared according to general procedure A using 1,2-dichlorovinyl sulfonamide **S7** (1.7 g, 5.0 mmol). The lithium acetylide was treated with water (10 mL) and the resulting mixture was stirred at 23 °C for 2 h. Column chromatography (heptane/AcOEt 20:1 → 10:1; silica gel was washed with 1% Et_3N in heptane before being used for column chromatography) afforded the product **1d** as a light yellow oil (81%). $\boldsymbol{R}_{\mathbf{F}}$ (silica gel, heptane/AcOEt 10:1): 0.18 (UV, $KMnO_4$ solution). ^{1}**H NMR** [400 MHz, δ (ppm), $CDCl_3$]: 2.44 (s, 3 H), 2.83 (s, 1 H), 7.23–7.34 (m, 7 H), 7.56–7.61 (m, 2 H). ^{13}**C NMR** [101 MHz, δ (ppm), $CDCl_3$]: 21.7, 59.1, 75.8, 126.2, 128.2, 128.3, 129.0, 129.4, 132.7, 138.4, 145.0. These data were in accordance to those reported in the literature.[11]

4-Methyl-*N*-phenyl-*N*-[(trimethylsilyl)ethynyl]benzenesulfonamide 1e.

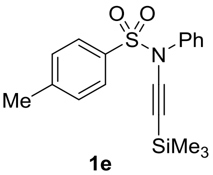

1e

Sulfonyl ynamine **1e** was prepared according to general procedure A using 1,2-dichlorovinyl sulfonamide **S7** (1.7 g, 5.0 mmol). The lithium acetylide was treated with trimethylsilyl chloride (952 µL, 7.5 mmol) at −78 °C for 15 min and at 23 °C for 2 h. Column chromatography (heptane/AcOEt 20:1; silica gel was washed with 1% Et_3N in heptane before being used for column chromatography) afforded the product **1e** as a white solid (52%). $\boldsymbol{R}_{\mathbf{F}}$ (silica gel, heptane/AcOEt 10:1): 0.27 (UV, $KMnO_4$ solution). ^{1}**H NMR** [400 MHz, δ (ppm), $CDCl_3$]: 0.17 (s, 9 H), 2.45 (s, 3 H), 7.21–7.25 (m, 2 H), 7.26–7.30 (m, 2 H), 7.30–7.36 (m, 3 H), 7.54–7.60 (m, 2 H). ^{13}**C NMR** [101 MHz, δ (ppm), $CDCl_3$]: 0.16, 21.9, 73.4, 93.2, 126.3, 128.3, 128.6, 129.2, 129.5, 133.0, 138.7, 145.1. **FTIR** [$\bar{\nu}$ (cm^{-1})]: 752, 834, 902, 1056, 1168, 1372, 1476, 1592, 2137, 2161, 2887, 3034. **HRMS** (ESI^+) calcd. for $[C_{18}H_{22}NO_2SSi + H]^+$ 344.1141, found 344.1134.

4-Methyl-*N*-phenyl-*N*-(phenylethynyl)benzenesulfonamide 1f.

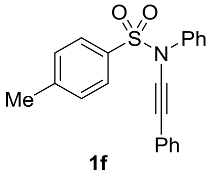

1f

Sulfonyl ynamine **1f** was prepared according to general procedure C. Column chromatography (heptane/AcOEt 20:1 → 10:1; silica gel was washed with 1% Et_3N in heptane before being used for column chromatography) afforded the product **1f** as a light yellow solid (80%). $\boldsymbol{R}_{\mathbf{F}}$ (silica gel, heptane/AcOEt 10:1): 0.27 (UV, $KMnO_4$ solution). ^{1}**H NMR** [400 MHz, δ (ppm), $CDCl_3$]: 2.45 (s, 3 H), 7.27–7.36 (m, 9 H), 7.36–7.42 (m, 3 H), 7.61–7.65 (m, 2 H). ^{13}**C NMR** [101 MHz, δ (ppm), $CDCl_3$]: 21.7, 70.6, 83.2, 122.7, 126.5, 126.3, 128.1, 128.32, 128.34, 129.0, 129.5, 131.4, 132.9, 139.0, 145.1. These data were in accordance to those reported in the literature.[12]

Ethyl 3-(*N*-phenylbenzenesulfonamido)propanoate 1g.

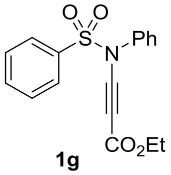

1g

Sulfonyl ynamine **1g** was prepared according to general procedure A using 1,2-dichlorovinyl sulfonamide **S6** (1.6 g, 5.0 mmol). The lithium acetylide was treated with freshly distilled ethyl chloroformate (714 µL, 7.5 mmol) at −78 °C for 15 min and at 23 °C for 2 h. Column chromatography (toluene; silica gel was washed with 1% Et_3N in heptane before being used for column chromatography) afforded the product **1g** as a white solid (74%). $\boldsymbol{R}_\mathbf{F}$ (silica gel, toluene): 0.35 (UV, $KMnO_4$ solution). **^{1}H NMR** [400 MHz, δ (ppm), $CDCl_3$]: 1.31 (t, *J* = 7.1 Hz, 3 H), 4.24 (q, *J* = 7.1 Hz, 2 H), 7.14–7.22 (m, 2 H), 7.30–7.41 (m, 3 H), 7.49–7.58 (m, 2 H), 7.64–7.73 (m, 1 H), 7.74–7.80 (m, 2 H). **^{13}C NMR** [101 MHz, δ (ppm), $CDCl_3$]: 14.2, 61.8, 66.5, 81.9, 126.6, 128.3, 129.27, 129.29, 129.5, 134.6, 135.8, 137.0, 154.0. **FTIR** [$\bar{\nu}$ (cm^{-1})]: 685, 726, 1088, 1123, 1204, 1372, 1702, 2218, 2982. **HRMS** (ESI^+) calcd. for $C_{17}H_{16}NO_4S$ $[M + H]^+$ 330.0800, found 330.0812.

Ethyl 3-(4-methoxy-*N*-phenylbenzenesulfonamido)propanoate 1h.

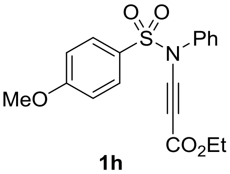

1h

Sulfonyl ynamine **1h** was prepared according to general procedure A using 1,2-dichlorovinyl sulfonamide **S8** (1.8 g, 5.0 mmol). The lithium acetylide was treated with freshly distilled ethyl chloroformate (714 µL, 7.5 mmol) at −78 °C for 15 min and at 23 °C for 2 h. Column chromatography (toluene; silica gel was washed with 1% Et_3N in heptane before being used for column chromatography) afforded the product **1h** as colorless oil (84%). $\boldsymbol{R}_\mathbf{F}$ (silica gel, toluene): 0.25 (UV, $KMnO_4$ solution). **^{1}H NMR** [400 MHz, δ (ppm), $CDCl_3$]: 1.30 (t, *J* = 7.1 Hz, 3 H), 3.88 (s, 3 H), 4.23 (q, *J* = 7.1 Hz, 2 H), 6.91–7.03 (m, 2 H), 7.15–7.24 (m, 2 H), 7.3–7.40 (m, 3 H), 7.64–7.73 (m, 2 H). **^{13}C NMR** [101 MHz, δ (ppm), $CDCl_3$]: 14.2, 55.8, 61.7, 66.6, 82.5, 114.4, 126.4, 127.2, 129.2, 129.5, 130.6, 137.2, 154.1, 164.4. **FTIR** [$\bar{\nu}$ (cm^{-1})]: 689, 1087, 1123, 1371, 1496, 1593, 1702, 2216, 2981. **HRMS** (ESI^+) calcd. for $[C_{18}H_{18}NO_5S + H]^+$ 360.0906, found 360.0922.

Ethyl 3-(4-nitro-*N*-phenylbenzenesulfonamido)propanoate 1i and **ethyl 3,3-bis(4-nitro-*N*-phenyl-benzenesulfonamido)acrylate 15.**

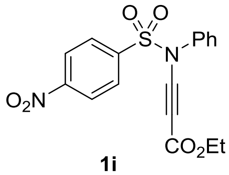

1i

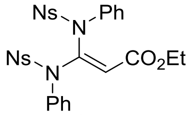

15

Sulfonyl ynamine **1i** and compound **15** were prepared according to general procedure A using 1,2-dichlorovinyl sulfonamide **S9** (1.9 g, 5.0 mmol). The lithium acetylide was treated with freshly distilled ethyl chloroformate (714 µL, 7.5 mmol) at −78 °C for 15 min and at 23 °C for 3 h. Column chromatography (toluene; silica gel was washed with 1% Et_3N in heptane before being used for column chromatography) afforded the product **1i** (42%) and product **15** (41%) as white solids. Product **15** was a common side product during the course of the reaction.[13] Compound **1i**: $\boldsymbol{R}_\mathbf{F}$ (silica gel, toluene): 0.33 (UV, $KMnO_4$ solution). **^{1}H NMR** [400 MHz, δ

(ppm), $CDCl_3$]: 1.32 (t, *J* = 7.1 Hz, 3 H), 4.25 (q, *J* = 7.1 Hz, 2 H), 7.17–7.23 (m, 2 H), 7.36–7.46 (m, 3 H), 7.92–8.00 (m, 2 H), 8.33–8.42 (m, 2 H). **^{13}C NMR** [101 MHz, δ (ppm), $CDCl_3$]: 14.1, 62.0, 66.7, 80.4, 124.5, 126.5, 129.7, 129.81, 129.84, 136.5, 140.9, 150.7, 153.6. **FTIR** [$\bar{\nu}$ (cm^{-1})]: 690, 740, 854, 1086, 1126, 1184, 1206, 1348, 1533, 1593, 1706, 2223, 2925, 3107. **HRMS** (ESI^+) calcd. for $[C_{17}H_{15}N_2O_6S + H]^+$ 375.0651, found 375.0659. Compound **15**: ***R*$_F$** (silica gel, toluene): 0.07 (UV, $KMnO_4$ solution). **^{1}H NMR** [400 MHz, δ (ppm), $CDCl_3$]: 1.41 (t, *J* = 7.1 Hz, 3 H), 4.33 (q, *J* = 7.1 Hz, 2 H), 6.14 (s, 1 H), 7.11–7.16 (m, 2 H), 7.20–7.23 (m, 2 H), 7.24–7.27 (m, 2 H), 7.33–7.38 (m, 2 H), 7.40–7.45 (m, 2 H), 7.45–7.50 (m, 3 H), 7.52–7.57 (m, 1 H), 7.93–8.00 (m, 2 H), 8.09–8.18 (m, 2 H). **^{13}C NMR** [101 MHz, δ (ppm), $CDCl_3$]: 14.1, 61.6, 110.0, 117.5, 123.4, 124.1, 129.4, 129.6, 129.7, 129.93, 129.97, 129.99, 130.0, 136.0, 136.3, 139.9, 143.4, 144.2, 149.9, 150.4, 163.9. **FTIR** [$\bar{\nu}$ (cm^{-1})]: 694, 740, 855, 1086, 1139, 1172, 1202, 1350, 1531, 1720, 3106. **HRMS** (ESI^+) calcd. for $[C_{29}H_{25}N_4O_{10}S_2 + H]^+$ 653.1012, found 653.1034.

Ethyl 3-(*N*,4-dimethylbenzenesulfonamido)propanoate 1j.

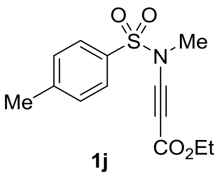

1j

Sulfonyl ynamine **1j** was prepared according to general procedure A using 1,2-dichlorovinyl sulfonamide **S11** (1.4 g, 5.0 mmol). The lithium acetylide was treated with freshly distilled ethyl chloroformate (714 µL, 7.5 mmol) at −78 °C for 15 min and at 23 °C for 2 h. Column chromatography (heptane/AcOEt 20:1 → 10:1; silica gel was washed with 1% Et_3N in heptane before being used for column chromatography) afforded the product **1j** as a white solid (71%). ***R*$_F$** (silica gel, heptane/AcOEt 1:1): 0.6 (UV, $KMnO_4$ solution). **^{1}H NMR** [400 MHz, δ (ppm), $CDCl_3$]: 1.31 (t, *J* = 7.1 Hz, 3 H), 2.47 (s, 3 H), 3.17 (s, 3 H), 4.23 (q, *J* = 7.1 Hz, 2 H), 7.35–7.44 (m, 2 H), 7.78–7.92 (m, 2 H). **^{13}C NMR** [101 MHz, δ (ppm), $CDCl_3$]: 14.2, 21.8, 38.5, 61.6, 66.0, 83.5, 128.0, 130.2, 133.0, 145.8, 154.1. These data were in accordance to those reported in the literature.[14]

Ethyl 3-(*N*-benzyl-4-methylbenzenesulfonamido)propanoate 1k.

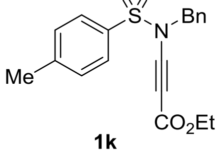

1k

Sulfonyl ynamine **1k** was prepared according to general procedure A using 1,2-dichlorovinyl sulfonamide **S10** (1.8 g, 5.0 mmol). The lithium acetylide was treated with freshly distilled ethyl chloroformate (714 µL, 7.5 mmol) at −78 °C for 15 min and at 23 °C for 2 h. Column chromatography (heptane/AcOEt 40:1 → 10:1; silica gel was washed with 1% Et_3N in heptane before being used for column chromatography) afforded the product **1k** as a white solid (87%). ***R*$_F$** (silica gel, heptane/AcOEt 10:1): 0.17 (UV, $KMnO_4$ solution). **^{1}H NMR** [400 MHz, δ (ppm), $CDCl_3$]: 1.28 (t, *J* = 7.1 Hz, 3 H), 2.44 (s, 3 H), 4.18 (q, *J* = 7.1 Hz, 2 H), 4.62 (s, 2 H), 7.24–7.33 (m, 7 H), 7.68–7.75 (m, 2 H). **^{13}C NMR** [101 MHz, δ (ppm), $CDCl_3$]: 14.2, 21.6, 55.3, 61.4, 68.1, 82.5, 127.6, 128.5, 128.6, 129.3, 129.7, 133.5, 134.1, 145.4, 153.9. These data were in accordance to those reported in the literature.[7]

Optimization Studies for Intramolecular Hydroarylation of Sulfonyl Ynamine 1a

Table 1. Screening of the Reaction Conditions

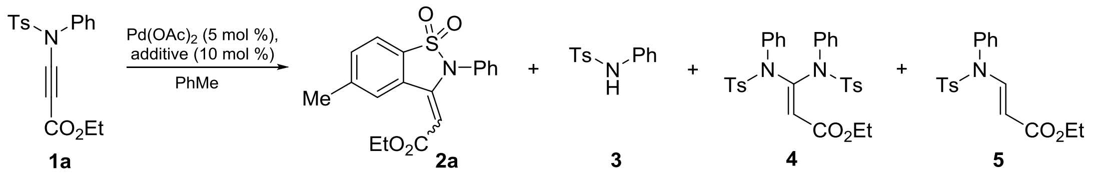

			Ratio of products (%)[a]						
Entry	Temperature (°C)	Additive	**1a**	**2a**	**3**	**4**	**5**	*E*/*Z*-Ratio[a]	Yield (*E*)-**2a** (%)[b]
**1**	100	–	27	35	33	3	2	82:18	21
**2**[c]	100	–	8	42	46	2	2	83:17	23
**3**	80	–	62	12	26	0	0	n.d.	<5
**4**	120	–	6	20	63	11	0	82:18	9
**5**	150	–	6	18	59	17	0	n.d.	<5
**6**	100	NaOAc[d]	28	14	58	0	0	85:15	8
**7**	100	AcOH	21	22	54	0	3	63:37	11
**8**	100	Tf_2NH	3	9	88	0	0	n.d.	<5
**9**	100	Zn	23	37	38	2	0	85:15	20

Conditions: **1a** (1.0 mmol), $Pd(OAc)_2$ (5 mol %), additive (10 mol %), PhMe (10.0 mL), 18 h. [a]Based on the ^1H NMR spectrum of the crude. [b]Isolated yield. [c]10 mol % of $Pd(OAc)_2$ was used. [d]NaOAc (2.0 equiv).

Table 2. Screening of the Ligand

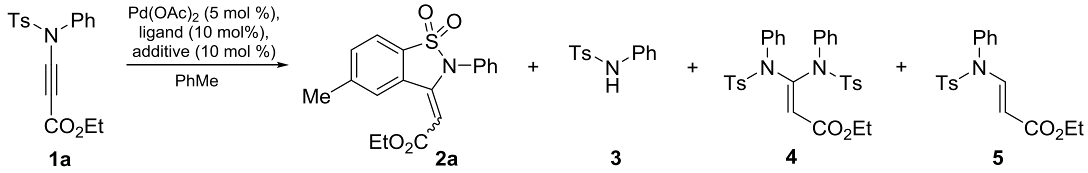

Entry	Ligand	Additive	**1a**	**2a**	**3**	**4**	**5**	*E*/*Z*-Ratio[a]	Yield (*E*)-**2a** (%)[b]
			Ratio of products (%)[a]						
**1**	PPh_3	Zn	15	38	38	9	0	92:8	20
**2**	PPh_3	AcOH	34	23	39	0	4	74:26	13
**3**	dppp	Zn	12	30	53	3	2	86:14	16
**4**	PCy_3	–	13	48	36	0	3	90:10	27
**5**	PCy_3	–	9	34	52	0	5	90:10	22
**6**	PCy_3	Zn	16	48	36	0	0	89:11	23
**7**	P(*p*-tol)$_3$	–	10	51	35	0	4	98:2	37
**8**	P(*p*-tol)$_3$	Zn	15	53	31	0	1	95:5	34
**9**	P(*o*-tol)$_3$	–	12	48	35	0	5	90:10	32
**10**	P(*o*-tol)$_3$	Zn	16	43	41	0	0	90:10	26

Conditions: 1a (1.0 mmol), $Pd(OAc)_2$ (5 mol %), ligand (10 mol %), additive (10 mol %), PhMe (10.0 mL), 18 h. [a]Based on the ^1H NMR spectrum of the crude. [b]Isolated yield.

Table 3. Screening of the Catalyst

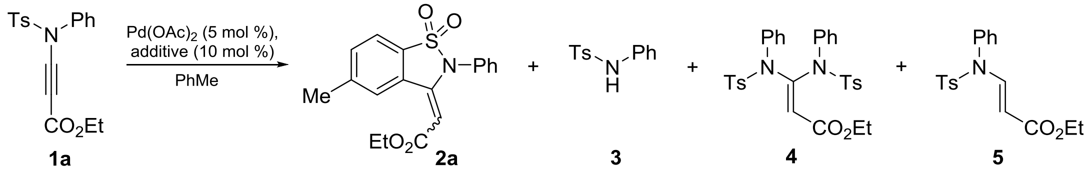

Entry	Catalyst	Temperature (°C)	**1a**	**2a**	**3**	**4**	**5**	*E*/*Z*-Ratio[a]	Yield (*E*)-**2a** (%)[b]
			ratio of products (%)[a]						
**1**	–	100	100	–	–	–	–	n.d.	n.d.
**2**	$Pd(PPh_3)_4$	100	30	10	58	2	0	90:10	7
**3**[c]	$Pd(PPh_3)_4$	100	43	23	17	3	14	92:8	14
**4**	AgOTf	23	10	0	0	48	0	n.d.	n.d.
**5**	Tf_2NH	23	100	0	0	0	0	n.d.	n.d.
**6**	$ClAu(PPh_3)$	100	unidentified products					n.d.	n.d.

Conditions: 1a (1.0 mmol), $Pd(OAc)_2$ (5 mol %), ligand (10 mol %), additive (10 mol %), PhMe (10.0 mL), 18 h. [a]Based on the ^1H NMR spectrum of the crude. [b]Isolated yield. [c]AcOH (10 mol %) was used.

Ethyl (*E*)-2-(5-methyl-1,1-dioxo-2-phenyl-1,2-benzothiazol-3(2*H*)-ylidene)acetate (*E*)-2a.

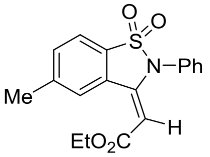
(*E*)-**2a**

***R*F** (silica gel, toluene): 0.33 (UV, $KMnO_4$ solution). **^{1}H NMR** [400 MHz, δ (ppm), $CDCl_3$]: 1.26 (t, *J* = 7.1 Hz, 3 H), 2.57 (s, 3 H), 4.18 (q, *J* = 7.1 Hz, 2 H), 5.11 (s, 1 H), 7.47–7.51 (m, 2 H), 7.54–7.62 (m, 4 H), 7.82 (d, *J* = 7.9 Hz, 1 H), 9.23 (quint, *J* = 0.8 Hz, 1 H). **^{13}C NMR** [101 MHz, δ (ppm), $CDCl_3$]: 14.2, 22.2, 60.5, 97.0, 120.9, 127.4, 129.8, 130.36, 130.39, 130.43, 130.5, 131.0, 133.1, 144.8, 146.2, 166.1. **FTIR** [ṽ (cm^{-1})]: 714, 902, 1089, 1174, 1302, 1512, 1635, 1737, 2992. **HRMS** (ESI^+) calcd. for $[C_{18}H_{18}NO_4S + H]^+$ 344.0957, found 344.0966.

Ethyl (*Z*)-2-(5-methyl-1,1-dioxo-2-phenyl-1,2-benzothiazol-3(2*H*)-ylidene)acetate (*Z*)-2a.

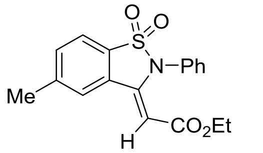
(*Z*)-**2a**

***R*F** (silica gel, toluene): 0.28 (UV, $KMnO_4$ solution). **^{1}H NMR** [400 MHz, δ (ppm), $CDCl_3$]: 1.01 (t, *J* = 7.1 Hz, 3 H), 2.54 (s, 3 H), 3.66 (q, *J* = 7.1 Hz, 2 H), 5.83 (s, 1 H), 7.36–7.49 (m, 5 H), 7.55 (m, *J* = 8.0, 1.2, 0.6 Hz, 1 H), 7.62 (quint, *J* = 0.6 Hz, 1 H), 7.79 (d, *J* = 8.0 Hz, 1 H). **^{13}C NMR** [101 MHz, δ (ppm), $CDCl_3$]: 13.9, 22.0, 60.4, 93.0, 121.5, 122.0, 126.9, 128.6, 128.9, 129.5, 130.2, 133.1, 134.9, 140.2, 144.9, 164.0. **FTIR** [ṽ (cm^{-1})]: 693, 907, 1082, 1145, 1266, 1327, 1494, 1635, 1708, 3029. **HRMS** (ESI^+) calcd. for $[C_{18}H_{18}NO_4S + H]^+$ 344.0957, found 344.0943.

4-Methyl-*N*-phenylbenzenesulfonamide 3. The spectroscopic data were identical to those reported above.

Ethyl 3,3-bis(4-methyl-*N*-phenylbenzenesulfonamido)acrylate 4.

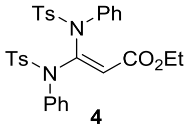
4

***R*F** (silica gel, toluene): 0.06 (UV, $KMnO_4$ solution). **^{1}H NMR** [400 MHz, δ (ppm), $CDCl_3$]: 1.39 (t, *J* = 7.1 Hz, 3 H), 2.30 (s, 3 H), 2.38 (s, 3 H), 4.33 (q, *J* = 7.1 Hz, 2 H), 6.09 (s, 1 H), 6.86–6.94 (m, 4 H), 7.06–7.12 (m, 2 H), 7.12–7.19 (m, 6 H), 7.26–7.33 (m, 2 H), 7.33–7.40 (m, 3 H), 7.42–7.48 (m, 1 H). **^{13}C NMR** [101 MHz, δ (ppm), $CDCl_3$]: 14.1, 21.5, 21.6, 61.1, 115.5, 127.3, 128.18, 128.23, 128.93, 128.97, 129.0, 129.4, 129.5, 130.0, 130.2, 135.1, 136.0, 136.8, 137.1, 140.6, 143.5, 144.3, 164.7. **FTIR** [ṽ (cm^{-1})]: 695, 814, 1087, 1139, 1167, 1362, 1489, 1531, 1719, 2981. **HRMS** (ESI^+) calcd. for $[C_{31}H_{31}N_2O_6S_2 + H]^+$ 591.1624, found 591.1638.

Ethyl (*E*)-3-(4-methyl-*N*-phenylbenzenesulfonamido)acrylate 5.

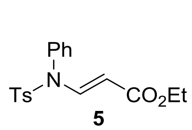
5

***R*F** (silica gel, toluene): 0.12 (UV, $KMnO_4$ solution). **^{1}H NMR** [400 MHz, δ (ppm), $CDCl_3$]: 1.24 (t, *J* = 7.1 Hz, 3 H), 2.44 (s, 3 H), 4.14 (q, *J* = 7.1 Hz, 2 H), 4.62 (d, *J* = 13.8 Hz, 1 H), 6.88–6.97 (m, 2 H), 7.27–7.32 (m, 2 H), 7.35–7.45 (m, 3 H), 7.53–7.60 (m, 2 H), 8.37 (d, *J* = 13.7 Hz, 1 H). **^{13}C NMR** [101 MHz, δ (ppm), $CDCl_3$]: 14.3, 21.7, 60.1, 100.0, 121.7, 127.8, 129.7, 129.86, 129.89, 134.8, 135.1, 143.9, 144.9, 167.1. **FTIR** [ṽ

(cm^{-1})]: 727, 906, 1089, 1170, 1368, 1624, 1737, 2245, 2981. **HRMS** (ESI^{+}) calcd. for [$C_{18}H_{20}NO_4S$ + H]$^{+}$ 346.1113, found 346.1131.

Intramolecular Hydroarylation of Sulfonyl Ynamine 1a–c and 1g–k

Scheme 2. Scope of Sulfonyl Ynamines **1a**–**k** and **1g**–**k** for Intramolecular Hydroarylation

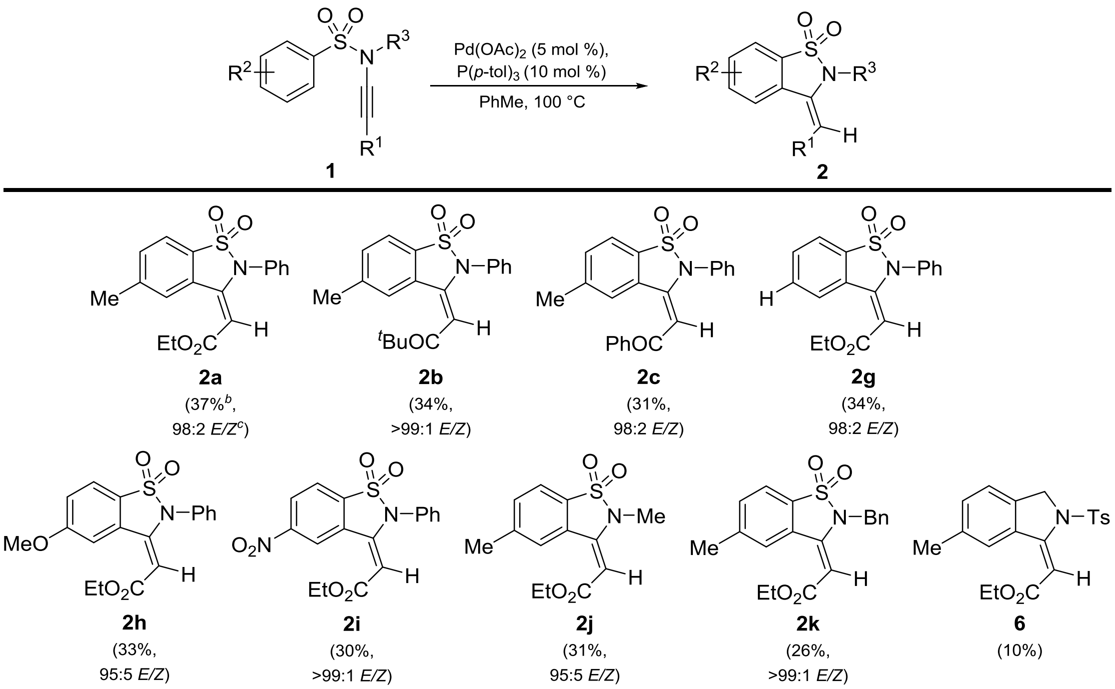

General procedure: Sulfonyl ynamine **1** (1.0 mmol, 1.0 equiv) was dissolved in toluene (10 mL) under a nitrogen atmosphere in a Biotage microwave vial (10.0–20.0 mL) equipped with a magnetic stirring bar. $Pd(OAc)_2$ (11 mg, 0.05 mmol, 0.05 equiv) and tri(*p*-tolyl)phosphine (30 mg, 0.1 mmol, 0.1 equiv) were added at 23 °C. The vial was covered with a Teflon septum and secured via a crimped aluminum cap. The reaction was irradiated in a Biotage Initiator microwave at 100 °C for 18 h (30 second pre-stir, Fixed Hold Time On, Low absorbance level). The reaction mixture was quenched with brine (50 mL) and extracted with AcOEt (2 × 20 mL). The resulting organic washings were dried over sodium sulfate, concentrated in vacuo and the crude residue was purified by column chromatography as indicated.

Ethyl (*E*)-2-(5-methyl-1,1-dioxo-2-phenyl-1,2-benzothiazol-3(2*H*)-ylidene)acetate (*E*)-2a.

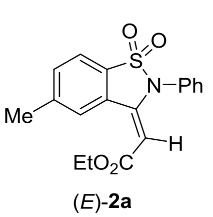

(*E*)-**2a**

(*E*)-1,2-Benzothiazoledione **2a** was prepared according to general procedure using sulfonyl ynamine **1a** (343 mg, 1.0 mmol). Ratio *E*/*Z* = 98:2 was determined by ^{1}H NMR spectroscopy. Column chromatography (toluene; silica gel was washed with 1% Et_3N in heptane before being used for column chromatography) afforded product (*E*)-2a as a white solid (127 mg, 37%). The spectroscopic data

were identical to those reported above (see Section "Optimization Studies for Intramolecular Hydroarylation of Sulfonyl Ynamine **1a**").

(*E*)-3,3-Dimethyl-1-(5-methyl-1,1-dioxo-2-phenyl-1,2-benzothiazol-3(2*H*)-ylidene)butan-2-one (*E*)-2b.

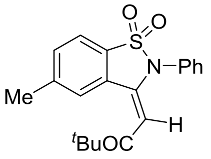

(*E*)-**2b**

(*E*)-1,2-Benzothiazoledione **2b** was prepared according to general procedure using sulfonyl ynamine **1b** (355 mg, 1.0 mmol). Ratio *E*/*Z* > 99:1 was determined by ^{1}H NMR spectroscopy. Column chromatography (toluene; silica gel was washed with 1% Et_3N in heptane before being used for column chromatography) afforded the product (*E*)-**2b** as a white solid (121 mg, 34%). $\boldsymbol{R}_\mathbf{F}$ (silica gel, toluene): 0.28 (UV, $KMnO_4$ solution). **^{1}H NMR** [400 MHz, δ (ppm), $CDCl_3$]: 1.05 (s, 9 H), 2.57 (s, 3 H), 5.71 (s, 1 H), 7.48–7.53 (m, 2 H), 7.54–7.65 (m, 4 H), 7.82 (d, *J* = 7.9 Hz, 1 H), 9.10 (quint, *J* = 0.6 Hz, 1 H). **^{13}C NMR** [101 MHz, δ (ppm), $CDCl_3$]: 22.3, 26.8, 44.7, 101.5, 121.0, 127.8, 129.4, 130.4, 130.5, 130.6, 130.8, 130.9, 133.4, 145.1, 145.6, 204.3. **FTIR** [$\bar{\nu}$ (cm^{-1})]: 695, 966, 1078, 1183, 1322, 1566, 1673, 2965. **HRMS** (ESI^+) calcd. for $[C_{20}H_{22}NO_3S + H]^+$ 356.1320, found 356.1343.

(*E*)-2-(5-methyl-1,1-dioxo-2-phenyl-1,2-benzothiazol-3(2*H*)-ylidene)-1-phenylethan-1-one (*E*)-2c.

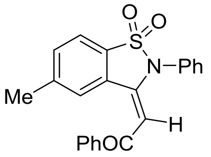

(*E*)-**2c**

(*E*)-1,2-Benzothiazoledione **2c** was prepared according to general procedure using sulfonyl ynamine **1c** (375 mg, 1.0 mmol). Ratio *E*/*Z* = 98:2 was determined by ^{1}H NMR spectroscopy. Column chromatography (toluene; silica gel was washed with 1% Et_3N in heptane before being used for column chromatography) afforded the product (*E*)-**2c** as a white solid (116 mg, 31%). $\boldsymbol{R}_\mathbf{F}$ (silica gel, toluene): 0.31 (UV, $KMnO_4$ solution). **^{1}H NMR** [400 MHz, δ (ppm), $CDCl_3$]: 2.57 (s, 3 H), 6.09 (s, 1 H), 7.37–7.45 (m, 2 H), 7.48–7.55 (m, 1 H), 7.56–7.66 (m, 6 H), 7.72–7.79 (m, 2 H), 7.85 (d, *J* = 8.0 Hz, 1 H), 9.00 (quint, *J* = 0.5 Hz, 1 H). **^{13}C NMR** [101 MHz, δ (ppm), $CDCl_3$]: 22.2, 102.4, 121.1, 127.4, 127.7, 128.2, 128.6, 129.1, 130.5, 130.6, 130.7, 130.9, 132.8, 133.6, 139.4, 145.1, 146.4, 189.7. **FTIR** [$\bar{\nu}$ (cm^{-1})]: 696, 959, 1185, 1323, 1565, 1653, 1724, 2922, 3064. **HRMS** (ESI^+) calcd. for $[C_{22}H_{18}NO_3S + H]^+$ 376.1007, found 376.1015.

Ethyl (*E*)-2-(1,1-dioxo-2-phenyl-1,2-benzothiazol-3(2*H*)-ylidene)acetate (*E*)-2g.

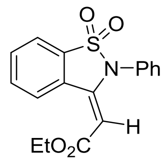

(*E*)-**2g**

(*E*)-1,2-Benzothiazoledione **2g** was prepared according to general procedure using sulfonyl ynamine **1g** (330 mg, 1.0 mmol). Ratio *E*/*Z* = 98:2 was determined by ^{1}H NMR spectroscopy. Column chromatography (toluene; silica gel was washed with 1% Et_3N in heptane before being used for column chromatography) afforded the product (*E*)-**2g** as a white solid (112 mg, 34%). $\boldsymbol{R}_\mathbf{F}$ (silica gel, toluene): 0.31 (UV, $KMnO_4$ solution). **^{1}H NMR** [400 MHz, δ (ppm), $CDCl_3$]: 1.28 (t, *J* = 7.1 Hz, 3 H), 4.20 (q, *J* = 7.1 Hz, 2 H), 5.16 (s, 1 H), 7.46–7.56 (m, 2 H), 7.57–7.67 (m, 3 H), 7.76–7.85 (m, 2 H), 7.92–8.02 (m, 1 H), 9.44 (m, 1 H). **^{13}C NMR** [101 MHz, δ (ppm), $CDCl_3$]: 14.4, 60.7, 97.6, 121.3, 127.4,

129.9, 130.5, 130.6, 130.7, 131.2, 132.5, 133.3, 133.9, 146.2, 166.3. **FTIR** [ν̄ (cm^{-1})]: 692, 910, 1045, 1099, 1157, 1186, 1324, 1619, 1708, 2925. **HRMS** (ESI^+) calcd. for $[C_{17}H_{16}NO_4S + H]^+$ 330.0800, found 330.0827.

Ethyl (*E*)-2-(5-methoxy-1,1-dioxo-2-phenyl-1,2-benzothiazol-3(2*H*)-ylidene)acetate (*E*)-2h.

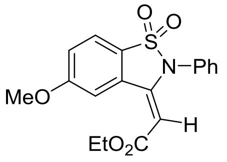

(*E*)-**2h**

(*E*)-1,2-Benzothiazoledione **2h** was prepared according to general procedure using sulfonyl ynamine **1h** (360 mg, 1.0 mmol). Ratio *E*/*Z* = 95:5 was determined by ^{1}H NMR spectroscopy. Column chromatography (toluene; silica gel was washed with 1% Et_3N in heptane before being used for column chromatography) afforded the product (*E*)-**2h** as a white solid (119 mg, 33%). ***R*$_F$** (silica gel, toluene): 0.17 (UV, $KMnO_4$ solution). **^{1}H NMR** [400 MHz, δ (ppm), $CDCl_3$]: 1.25 (t, *J* = 7.1 Hz, 3 H), 3.98 (s, 3 H), 4.16 (q, *J* = 7.1 Hz, 2 H), 5.12 (s, 1 H), 7.25 (dd, *J* = 8.7, 2.2 Hz, 1 H), 7.47–7.52 (m, 2 H), 7.53–7.64 (m, 3 H), 7.83 (d, *J* = 8.6 Hz, 1 H), 9.14 (d, *J* = 2.3 Hz, 1 H). **^{13}C NMR** [101 MHz, δ (ppm), $CDCl_3$]: 14.3, 56.1, 60.5, 97.2, 113.4, 119.7, 122.4, 125.1, 129.6, 130.43, 130.49, 130.53, 131.0, 146.3, 164.0, 166.2. **FTIR** [ν̄ (cm^{-1})]: 1103, 1183, 1252, 1307, 1473, 1583, 1621, 1707, 2988, 3118. **HRMS** (ESI^+) calcd. for $[C_{18}H_{18}NO_5S + H]^+$ 360.0906, found 360.0919.

Ethyl (*E*)-2-(5-nitro-1,1-dioxo-2-phenyl-1,2-benzothiazol-3(2*H*)-ylidene)acetate (*E*)-2i.

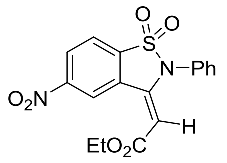

(*E*)-**2i**

(*E*)-1,2-Benzothiazoledione **2i** was prepared according to general procedure using sulfonyl ynamine **1i** (375 mg, 1.0 mmol). Ratio *E*/*Z* > 99:1 was determined by ^{1}H NMR spectroscopy. Column chromato-graphy (toluene; silica gel was washed with 1% Et_3N in heptane before being used for column chromatography) afforded the product (*E*)-**2i** as a white solid (113 mg, 30%). ***R*$_F$** (silica gel, toluene): 0.28 (UV, $KMnO_4$ solution). **^{1}H NMR** [400 MHz, δ (ppm), $CDCl_3$]: 1.28 (t, *J* = 7.1 Hz, 3 H), 4.24 (q, *J* = 7.1 Hz, 2 H), 5.27 (s, 1 H), 7.46–7.56 (m, 2 H), 7.58–7.69 (m, 3 H), 8.12 (d, *J* = 8.5 Hz, 1 H), 8.61 (dd, *J* = 8.5, 1.9 Hz, 1 H), 10.43 (d, *J* = 1.9 Hz, 1 H). **^{13}C NMR** [101 MHz, δ (ppm), $CDCl_3$]: 14.2, 61.2, 99.9, 122.5, 125.6, 127.3, 129.1, 129.6, 130.7, 130.9, 131.1, 137.6, 143.8, 151.2, 165.6. **FTIR** [ν̄ (cm^{-1})]: 694, 740, 1095, 1113, 1186, 1341, 1537, 1706, 2926, 3111. **HRMS** (ESI^+) calcd. for $[C_{17}H_{15}N_2O_6S + H]^+$ 375.0651, found 375.0667.

Ethyl (*E*)-2-(2,5-dimethyl-1,1-dioxo-1,2-benzothiazol-3(2*H*)-ylidene)acetate (*E*)-2j.

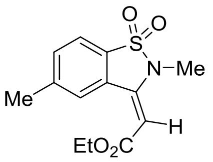

(*E*)-**2j**

(*E*)-1,2-Benzothiazoledione **2j** was prepared according to general procedure using sulfonyl ynamine **1j** (281 mg, 1.0 mmol). Ratio *E*/*Z* = 95:5 was determined by ^{1}H NMR spectroscopy. Column chromato-graphy (toluene; silica gel was washed with 1% Et_3N in heptane before being used for column chromatography) afforded the product (*E*)-**2j** as colorless oil (87 mg, 31%). ***R*$_F$** (silica gel, toluene): 0.23 (UV, $KMnO_4$ solution). **^{1}H NMR** [400 MHz, δ (ppm), $CDCl_3$]: 1.35 (t, *J* = 7.1 Hz, 3 H), 2.54 (s, 3

H), 3.15 (s, 3 H), 4.25 (q, J = 7.1 Hz, 2 H), 5.33 (s, 1 H), 7.50 (d, J = 7.9 Hz, 1 H), 7.76 (d, J = 7.9 Hz, 1 H), 9.17 (s, 1 H). ^{13}**C NMR** [101 MHz, δ (ppm), $CDCl_3$]: 14.3, 22.2, 26.5, 60.5, 95.0, 120.8, 127.9, 129.8, 130.5, 132.7, 144.8, 144.9, 166.0. **FTIR** [ν̄ (cm^{-1})]: 701, 823, 1039, 1137, 1159, 1315, 1619, 1708, 2982. **HRMS** (ESI$^+$) calcd. for $[C_{13}H_{16}NO_4S + H]^+$ 282.0800, found 282.0819.

Ethyl (*E*)-2-(2-benzyl-5-methyl-1,1-dioxo-1,2-benzothiazol-3(2*H*)-ylidene)acetate (*E*)-2k and **ethyl (*E*)-2-[2-(4-methylbenzenesulfonyl)isoindolin-1-ylidene]acetate 6.**

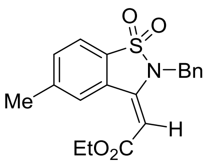

(*E*)-**2k**

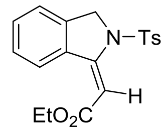

6

(*E*)-1,2-Benzothiazoledione **2k** and compound **6** were prepared according to general procedure using sulfonyl ynamine **1k** (357 mg, 1.0 mmol). Ratio *E*/*Z* > 99:1 was determined by ^{1}H NMR spectroscopy. Column chromatography (toluene; silica gel was washed with 1% Et_3N in heptane before being used for column chromatography) afforded the product (*E*)-**2k** as a white solid (93 mg, 26%) and product **6** as a colorless oil (36 mg, 10%). Compound (*E*)-**2k**: $\boldsymbol{R}_\mathbf{F}$ (silica gel, toluene): 0.30 (UV, $KMnO_4$ solution). ^{1}**H NMR** [400 MHz, δ (ppm), $CDCl_3$]: 1.26 (t, J = 7.1 Hz, 3 H), 2.53 (s, 3 H), 4.15 (q, J = 7.1 Hz, 2 H), 4.84 (s, 2 H), 5.28 (s, 1 H), 7.27–7.33 (m, 1 H), 7.34–7.40 (m, 2 H), 7.40–7.44 (m, 2 H), 7.52 (dd, J = 7.9, 0.6 Hz, 1 H), 7.81 (d, J = 7.9 Hz, 1 H), 9.13 (quint, J = 0.6 Hz, 1 H). ^{13}**C NMR** [101 MHz, δ (ppm), $CDCl_3$]: 14.4, 22.4, 44.5, 60.6, 96.5, 121.0, 127.2, 127.8, 128.1, 129.1, 130.1, 130.4, 132.9, 134.1, 143.7, 145.1, 166.0. **FTIR** [ν̄ (cm^{-1})]: 670, 732, 831, 1136, 1168, 1311, 1626, 1706, 2986, 3098. **HRMS** (ESI$^+$) calcd. for $[C_{19}H_{20}NO_4S + H]^+$ 358.1113, found 358.1118. Compound **6**: $\boldsymbol{R}_\mathbf{F}$ (silica gel, toluene): 0.21 (UV, $KMnO_4$ solution). ^{1}**H NMR** [400 MHz, δ (ppm), $CDCl_3$]: 1.32 (t, J = 7.1 Hz, 3 H), 2.40 (s, 3 H), 4.19 (q, J = 7.1 Hz, 2 H), 5.02 (s, 2 H), 6.43 (s, 1 H), 7.28–7.39 (m, 4 H), 7.41–7.47 (m, 1 H), 7.76–7.83 (m, 2 H), 9.13 (d, J = 8.1 Hz, 1 H). ^{13}**C NMR** [101 MHz, δ (ppm), $CDCl_3$]: 14.6, 21.8, 55.4, 60.1, 96.7, 121.9, 127.7, 128.4, 128.9, 130.1, 131.3, 133.0, 134.6, 138.1, 145.1, 152.0, 167.0. **FTIR** [ν̄ (cm^{-1})]: 667, 800, 1089, 1165, 1344, 1620, 1705, 2922. **HRMS** (ESI$^+$) calcd. for $[C_{19}H_{20}NO_4S + H]^+$ 358.1113, found 358.1132.

Intramolecular Hydroarylation of 1d, 1e and 1f

Scheme 3. Intramolecular Hydroarylation of **1d**, **1e** and **1f**

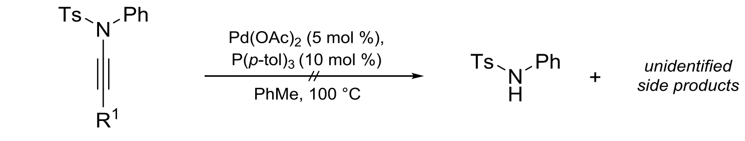

Sulfonyl ynamines **1d**, **1e** or **1f** were submitted to the reaction conditions according to general procedure. ^{1}H NMR spectroscopy of the crude product indicated partial degradation of the starting material to 4-methyl-*N*-phenylbenzenesulfonamide **3** and additional unidentified products. The formation of products **2d**, **2e** or **2f** was not observed.

Intramolecular Hydroarylation of Ethyl Acrylate 5

Scheme 4. Intramolecular Hydroarylation of Ethyl Acrylate **5**

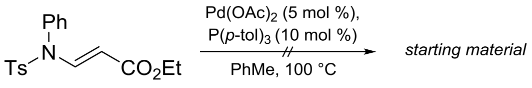

Ethyl Acrylate **5** was submitted to the reaction conditions according to general procedure. ^{1}H NMR spectroscopy of the crude product indicated no reaction of the substrate. Ethyl acrylate **5** was recovered in 94% yield after purification on the column chromatography (toluene; silica gel was washed with 1% Et_3N in heptane before being used for column chromatography).

Study of Equilibration between *E*- and *Z*-isomers of 2a

General procedure: (*E*)-1,2-Benzothiazole-1,1-dione **2a** or (*Z*)-1,2-Benzothiazole-1,1-dione **2a** (69 mg, 0.2 mmol, 1.0 equiv) were independently dissolved in toluene (2 mL) under a nitrogen atmosphere in a Biotage microwave vial (2.0–5.0 mL) equipped with a magnetic stirring bar. Pd(OAc)$_2$ (2.2 mg, 0.01 mmol, 0.05 equiv) and tri(*p*-tolyl)phosphine (6.1 mg, 0.02 mmol, 0.1 equiv) were added at 23 °C. The vial was covered with a Teflon septum and secured via a crimped aluminum cap. The reaction was irradiated in a Biotage Initiator microwave at 100 °C for 18 h (30 second pre-stir, Fixed Hold Time On, Low absorbance level). The reaction mixture was quenched with brine (50 mL) and extracted with AcOEt (2 × 20 mL). The combined organic washings were dried over sodium sulfate, filtered off and concentrated in vacuo. ^{1}H NMR spectroscopy of the crude product indicated a mixture of isomers with ratio E/Z = 85:15 (Scheme 5). Additionally, partial decomposition of the starting material was observed.

These results indicate the possible isomerization of (*E*)- and (*Z*)-isomers of **2a** under the reaction conditions used for the intramolecular hydroarylation of the ynamines.

Scheme 5. Equilibration of (*E*)- to (*Z*)-Isomer of 1,2-Benzothiazole-1,1-dione **2a**

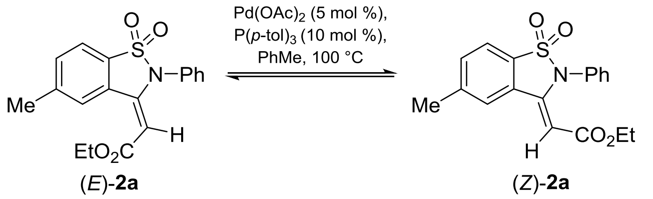

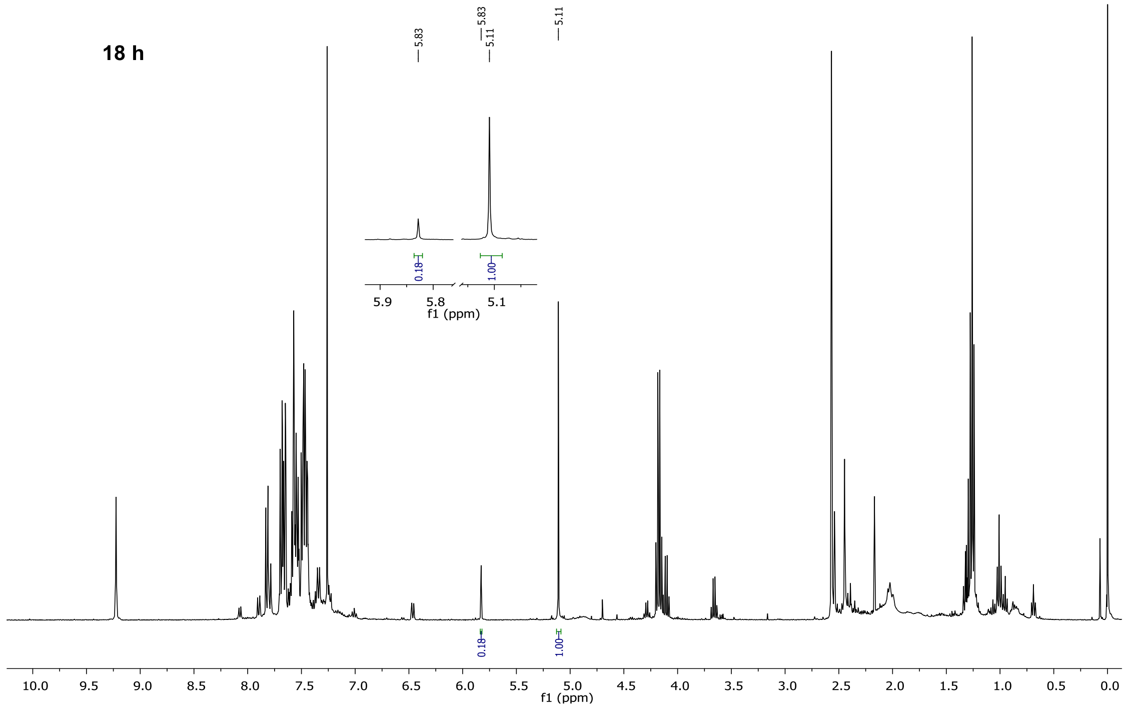

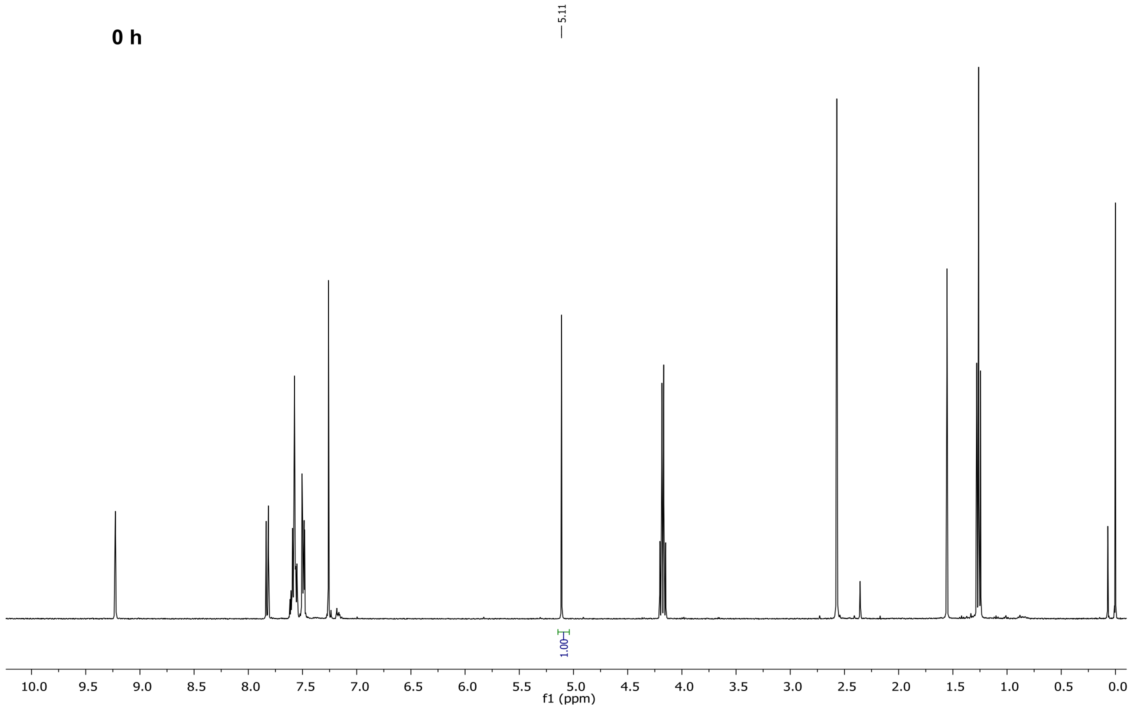

Photochemical Rearrangement of 1,2-Benzothiazole-1,1-diones

Scheme 6. Scope of the Photochemical Rearrangement of 1,2-Benzothiazole-1,1-diones

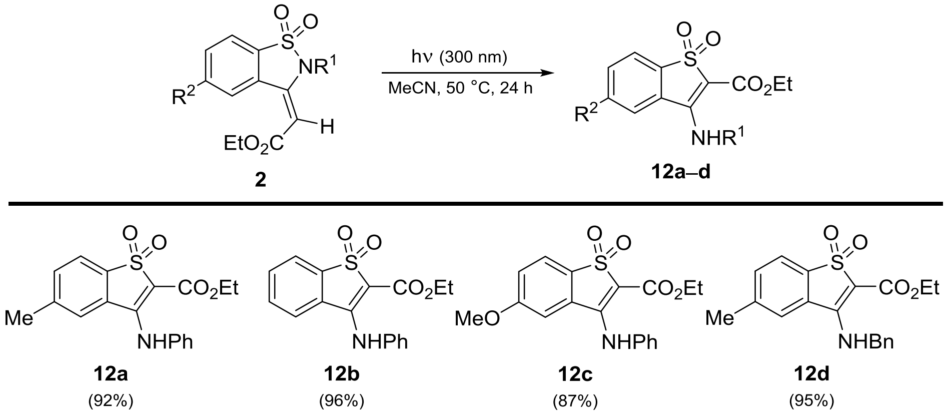

General procedure: To 25 mL quartz flask was added 1,2-benzothiazole-1,1-dione **2a**, **2g**, **2h** or **2k** (0.1–1.0 mmol, 1.0 equiv) in 10 mL of deoxygenated MeCN with stirring. This flask was irradiated in a Rayonet RMR-600 photochemical reactor, using eight lamps of 300 nm of wavelength for 24 h with internal temperature of 50 °C (Figure 1). After cooling to 23 °C, the solvent was removed in vacuo and the crude residue was purified by column chromatography as indicated.

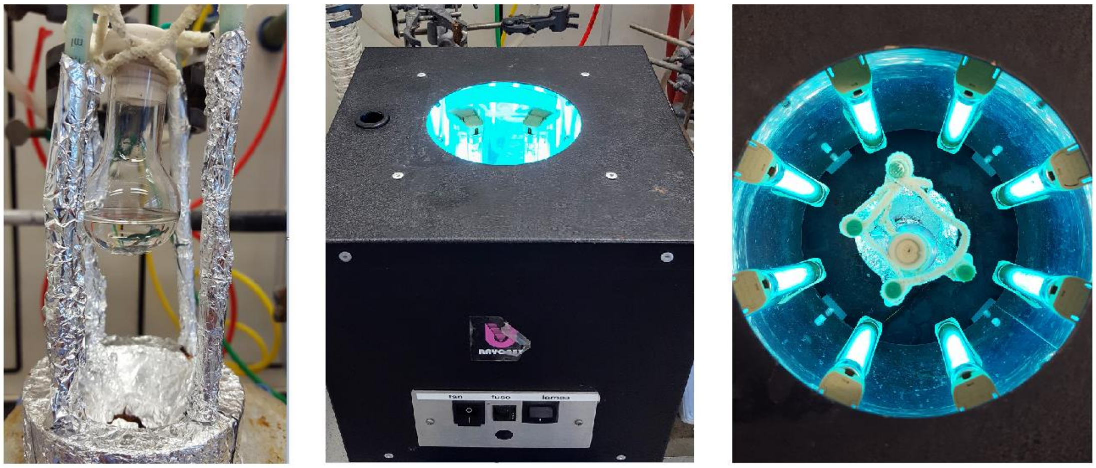

Figure 1. Reaction setup for the Photochemical Rearrangement

Ethyl 5-methyl-1,1-dioxo-3-(phenylamino)-1-benzothiophene-2-carboxylate 12a.

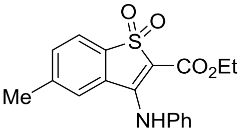

12a

3-Amino-1-benzothiophene-1,1-dione **12a** was prepared according to general procedure using 1,2-benzothiazole-1,1-dione **2a** (343 mg, 1.0 mmol). Column chromatography (toluene; silica gel was washed with 1% Et_3N in heptane before being used for column chromatography) afforded the product **12a** as a white solid (316 mg, 92%). ***R*$_F$** (silica gel, toluene): 0.22 (UV, $KMnO_4$ solution). **^{1}H NMR** [400 MHz, δ (ppm), $CDCl_3$]: 1.45 (t, *J* = 7.1 Hz, 3 H), 2.12 (s, 3 H), 4.43 (q, *J* = 7.1 Hz, 2 H), 6.40 (quint, *J* = 0.5 Hz,

1 H), 7.30–7.34 (m, 2 H), 7.39 (ddd, J = 7.8, 1.3, 0.7 Hz, 1 H), 7.44–7.54 (m, 3 H), 7.71 (d, J = 7.8 Hz, 1 H), 10.43 (bs, 1 H). **^{13}C NMR** [101 MHz, δ (ppm), $CDCl_3$]: 14.4, 21.7, 61.1, 100.1, 121.2, 125.9, 126.5, 127.0, 128.6, 129.8, 133.6, 137.4, 138.2, 142.8, 155.0, 164.5. **FTIR** [$\bar{\nu}$ (cm^{-1})]: 704, 730, 1027, 1122, 1160, 1247, 1285, 1569, 1657, 2854, 2925. **HRMS** (ESI^+) calcd. for $[C_{18}H_{18}NO_4S + H]^+$ 344.0957, found 344.0963.

Ethyl 1,1-dioxo-3-(phenylamino)-1-benzothiophene-2-carboxylate 12b.

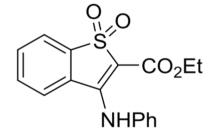

12b

3-Amino-1-benzothiophene-1,1-dione **12b** was prepared according to general procedure using 1,2-benzothiazoledione **2g** (33 mg, 0.1 mmol). Column chromatography (toluene; silica gel was washed with 1% Et_3N in heptane before being used for column chromatography) afforded the product **12b** as a white solid (31 mg, 96%). ***R*$_F$** (silica gel, toluene): 0.24 (UV, $KMnO_4$ solution). **^{1}H NMR** [400 MHz, δ (ppm), $CDCl_3$]: 1.45 (t, J = 7.1 Hz, 3 H), 4.44 (q, J = 7.1 Hz, 2 H), 6.66 (d, J = 8.0 Hz, 1 H), 7.23–7.28 (m, 1 H), 7.29–7.36 (m, 2 H), 7.43–7.54 (m, 3 H), 7.58–7.62 (m, 1 H), 7.83 (d, J = 7.6 Hz, 1 H), 10.44 (bs, 1 H). **^{13}C NMR** [101 MHz, δ (ppm), $CDCl_3$]: 14.4, 61.3, 99.9, 121.6, 125.7, 125.9, 126.9, 128.7, 130.0, 132.1, 133.2, 137.4, 141.0, 154.8, 164.6. **FTIR** [$\bar{\nu}$ (cm^{-1})]: 702, 763, 1161, 1246, 1284, 1434, 1561, 1610, 1657, 2854, 2925. **HRMS** (ESI^+) calcd. for $[C_{17}H_{16}NO_4S + H]^+$ 330.0800, found 330.0813.

Ethyl 5-methoxy-1,1-dioxo-3-(phenylamino)-1-benzothiophenecarboxylate 12c.

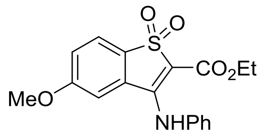

12c

3-Amino-1-benzothiophene-1,1-dione **12c** was prepared according to general procedure using 1,2-benzothiazole-1,1-dione **2h** (36 mg, 0.1 mmol). Column chromatography (toluene; silica gel was washed with 1% Et_3N in heptane before being used for column chromatography) afforded the product **12c** as a white solid (31 mg, 87%). ***R*$_F$** (silica gel, toluene): 0.20 (UV, $KMnO_4$ solution). **^{1}H NMR** [400 MHz, δ (ppm), $CDCl_3$]: 1.45 (t, J = 7.1 Hz, 3 H), 3.48 (s, 3 H), 4.43 (q, J = 7.1 Hz, 2 H), 6.13 (d, J = 2.2 Hz, 2 H), 7.04 (dd, J = 8.5, 2.2 Hz, 1 H), 7.32–7.38 (m, 2 H), 7.44–7.53 (m, 3 H), 7.72 (d, J = 8.5 Hz, 1 H), 10.41 (bs, 1 H). **^{13}C NMR** [101 MHz, δ (ppm), $CDCl_3$]: 14.4, 55.4, 61.2, 100.7, 111.6, 118.3, 122.8, 127.2, 127.8, 128.8, 130.0, 132.7, 137.4, 154.4, 162.3, 164.5. **FTIR** [$\bar{\nu}$ (cm^{-1})]: 703, 729, 910, 1021, 1162, 1245, 1567, 1655, 2926. **HRMS** (ESI^+) calcd. for $[C_{18}H_{18}NO_5S + H]^+$ 360.0906, found 330.0927.

Ethyl 3-(benzylamino)-5-methyl-1,1-dioxo-1-benzothiophene-2-carboxylate 12d.

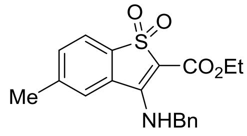

12d

3-Amino-1-benzothiophene-1,1-dione **12d** was prepared according to general procedure using 1,2-benzothiazole-1,1-dione **2k** (36 mg, 0.1 mmol). Column chromatography (toluene; silica gel was washed with 1% Et_3N in heptane before being used for column chromatography) afforded the product **12d** as a white solid (34 mg, 95%). ***R*$_F$** (silica gel, toluene): 0.25 (UV, $KMnO_4$ solution). **^{1}H NMR** [400 MHz, δ (ppm), $CDCl_3$]: 1.40 (t, J = 7.1 Hz, 3 H), 2.40 (s, 3 H), 4.36 (q, J = 7.1 Hz, 2 H), 5.02 (d, J = 5.9 Hz, 2

H), 7.33–7.40 (m, 3 H), 7.40–7.46 (m, 2 H), 7.49 (d, $J = 7.8$ Hz, 1 H), 7.59 (s, 1 H), 7.77 (d, $J = 7.8$ Hz, 1 H), 9.44 (bs, 1 H). **^{13}C NMR** [101 MHz, δ (ppm), $CDCl_3$]: 14.5, 22.2, 50.0, 61.0, 100.1, 121.2, 125.9, 126.5, 127.0, 128.6, 129.8, 133.6, 137.4, 138.2, 142.8, 154.9, 164.5. **FTIR** [$\bar{\nu}$ (cm^{-1})]: 735, 786, 1141, 1216, 1268, 1577, 1659, 1738, 2925, 3254. **HRMS** (ESI^+) calcd. for $[C_{19}H_{20}NO_4S + H]^+$ 358.1113, found 358.1133.

X-Ray Crystallography of (*E*)-2a, 12a and 12d

General procedure on example of (*E*)-2a: High quality single crystal of (*E*)-**2a** was obtained by slow solvent evaporation. A saturated solution of (*E*)-**2a** in toluene was prepared and filtered over an Acrodisc HPLC syringe filter to ensure the absence of crystallites. The resulting filtrate was transferred to a scintillation flask that was sealed and equipped with a hollow needle to allow slow evaporation of the solvent. The acquired single crystal was subjected to single X-ray diffraction. Mercury software (Version 3.9; Cambridge Crystallographic Data Centre) was used to visualize the structure (Schemes 7–9).

Scheme 7. Crystal structure of (*E*)-**2a**

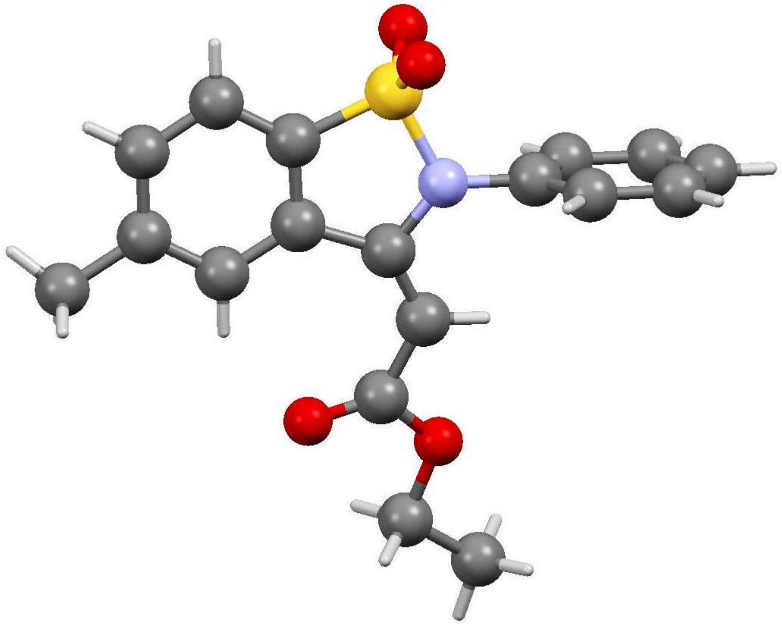

Scheme 8. Crystal structure of **12a**

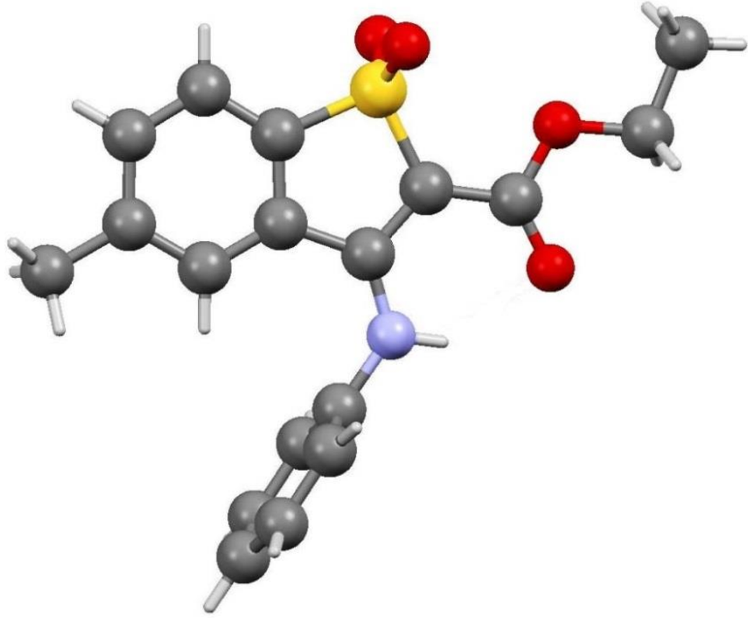

Scheme 9. Crystal structure of **12d**

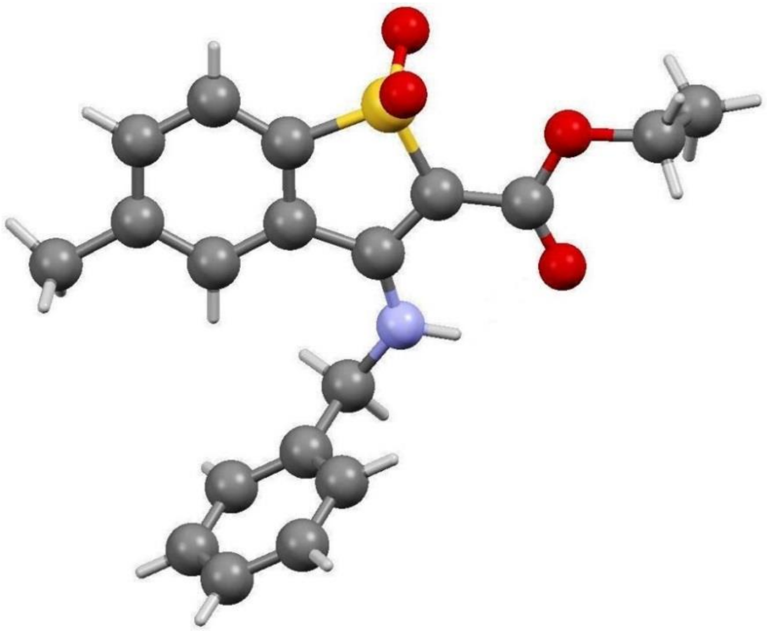

References and Notes

[1] O'Sullivan, S.; Doni, E.; Tuttle, T.; Murphy, J. A. *Angew. Chem. Int. Ed.* **2014**, *53*, 474–478.

[2] Zhang, W.; Xie, J.; Rao, B.; Luo, M. *J. Org. Chem.* **2015**, *80*, 3504–3511.

[3] Laha, J. K.; Jethava, K. P.; Dayal, N. *J. Org. Chem.* **2014**, *79*, 8010–8019.

[4] Heffernan, S. J.; Beddoes, J. M.; Mahon, M. F.; Hennessy, A. J.; Carbery, D. R. *Chem. Commun.* **2013**, *49*, 2314–2316.

[5] Li, F.; Xie, J.; Shan, H.; Sun, C.; Chen, L. *RSC Adv.* **2012**, *2*, 8645–8652.

[6] Mansfield, S. J.; Campbell, C. D.; Jones, M. W.; Anderson, E. A. *Chem. Commun.* **2015**, *51*, 3316–3319.

[7] Geary, L. M.; Hultin, P. G. *Org. Lett.* **2009**, *11*, 5478–5481.

[8] Gillie, A. D.; Jannapu Reddy, R.; Davies, P. W. *Adv. Synth. Catal.* **2016**, *358*, 226–239.

[9] Zhang, P.; Cook, A. M.; Liu, Y.; Wolf, C. *J. Org. Chem.* **2014**, *79*, 4167−4173.

[10] Tracey, M. R.; Zhang, Y.; Frederick, M. O.; Mulder, J. A.; Hsung, R. P. *Org. Lett.* **2004**, *6*, 2209–2212.

[11] Brückner, D. *Tetrahedron* **2006**, *62*, 3809–3814.

[12] Davies, P. W.; Cremonesi, A.; Martin, N. *Chem. Commun.* **2011**, *47*, 379–381.

[13] This side product is a result of addition of 4-nitro-*N*-phenylbenzenesulfonamide **S4** to **1i** formed during the reaction.

[14] Chen, P.; Song, C.-X.; Wang, W.-S.; Yua, X.-L.; Tang, Y. *RSC Adv.* **2016**, *6*, 80055–80058.

Content

Computational Methods 3

DFT Studies of the Reaction Mechanism 4

Cartesian coordinates of the structures involved in the computational study.......................................8

Content

Sulfonyl Ynamines **1e**, **1g**, **1h**, **1i** and Compound **15** 3

Intramolecular Hydroarylation of Sulfonyl Ynamine **1a**. Products (*E*)-**2a**, (*Z*)-**2a**, **4** and **5**......... 13

Intramolecular Hydroarylation of Sulfonyl Ynamines. Products (*E*)-**2b**–**k** and **6**........................ 23

Photochemical Rearrangement of 1,2-Benzothiazole-1,1-diones. Products **12a**–**d**...................... 45

Sulfonyl Ynamines 1e, 1g, 1h, 1i and Compound 15

IB351-2F2

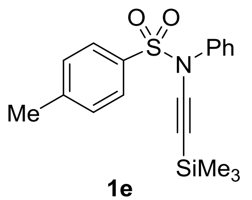

1e

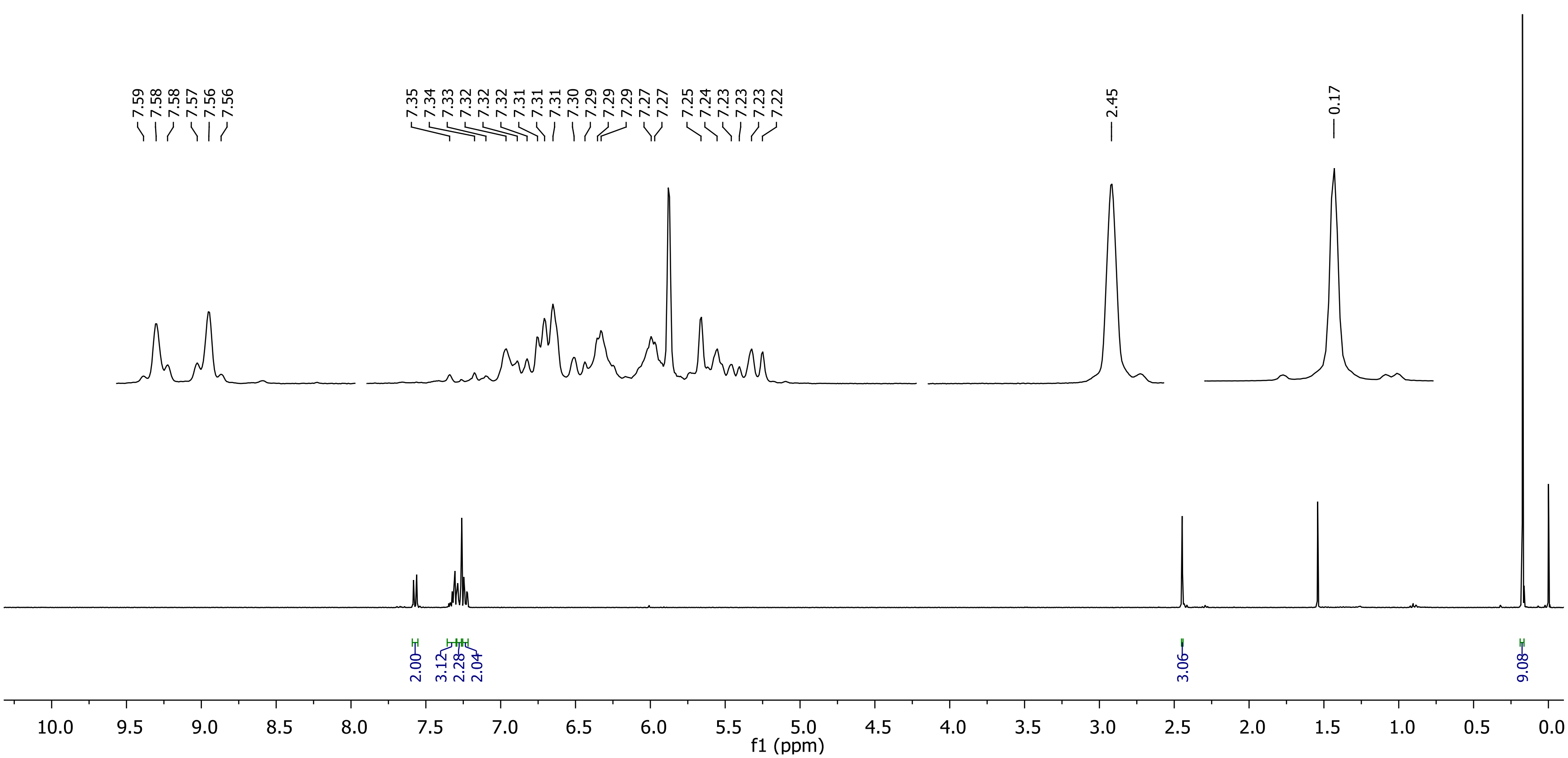

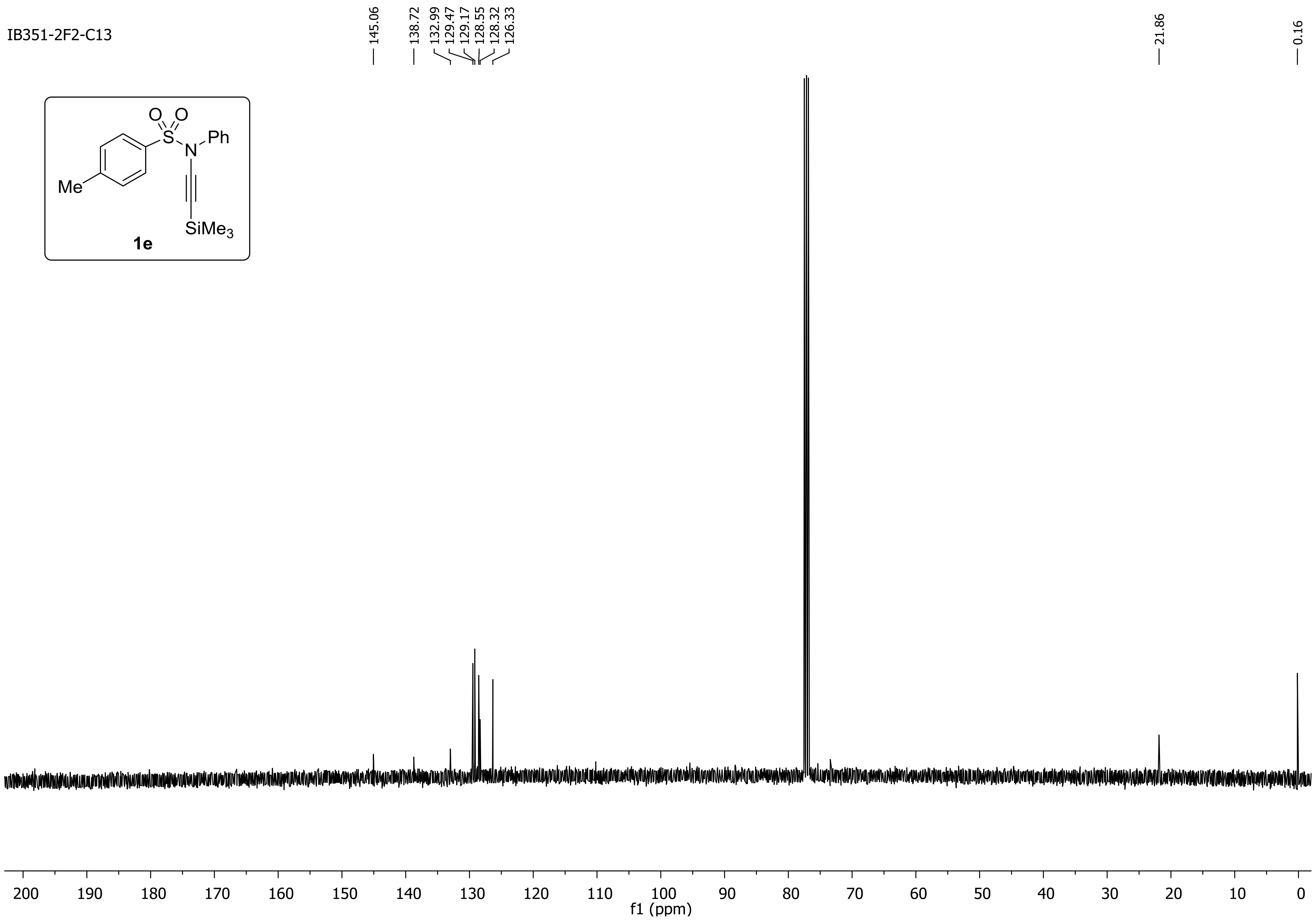
IB351-2F2-C13
145.06
138.72
132.99
129.47
129.17
128.55
128.32
126.33
21.86
0.16
Me
Ph
SiMe3
1e
200
190
180
170
160
150
140
130
120
110
100
90
80
70
60
50
40
30
20
10
0
f1 (ppm)

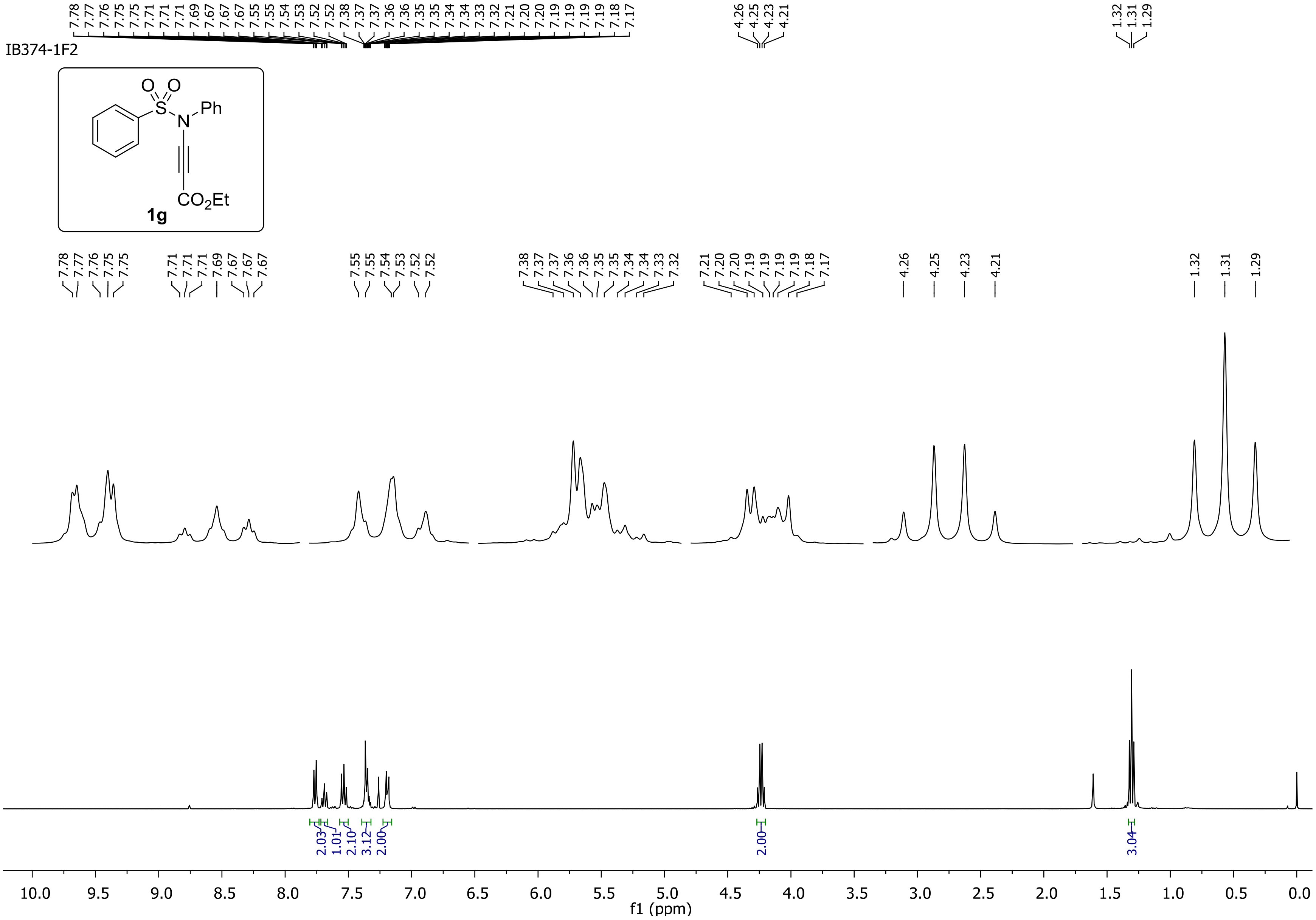
IB374-1F2
Ph
N
S
O O
CO2Et
1g
f1 (ppm)

IB374-1F2

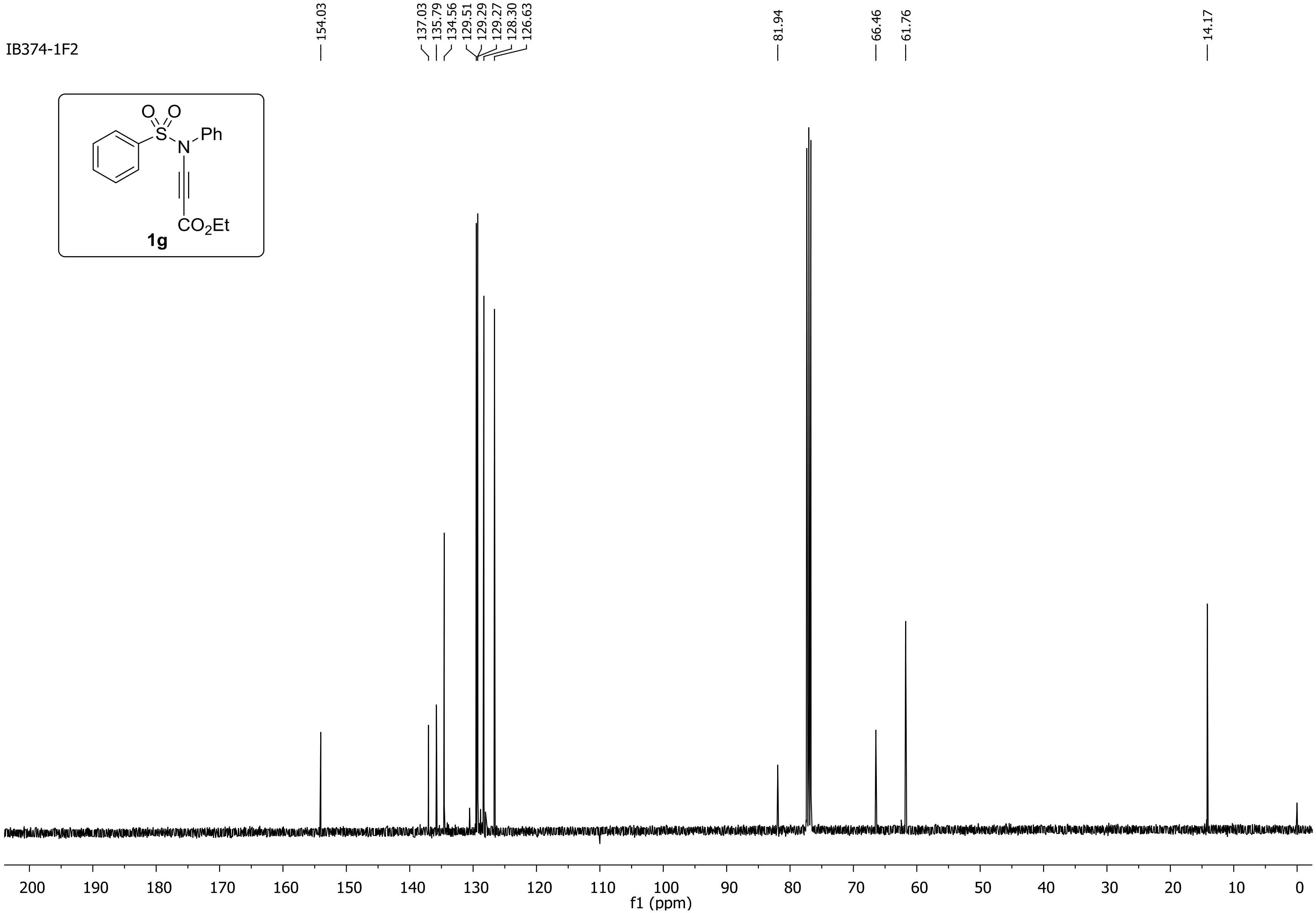
154.03
137.03
135.79
134.56
129.51
129.29
129.27
128.30
126.63
81.94
66.46
61.76
14.17
O
O
S
N
Ph
CO_2Et
1g
200 190 180 170 160 150 140 130 120 110 100 90 80 70 60 50 40 30 20 10 0
f1 (ppm)

IB371-1F1

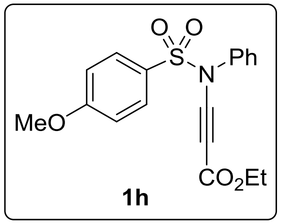
MeO
Ph
N
S
O
O
CO2Et
1h

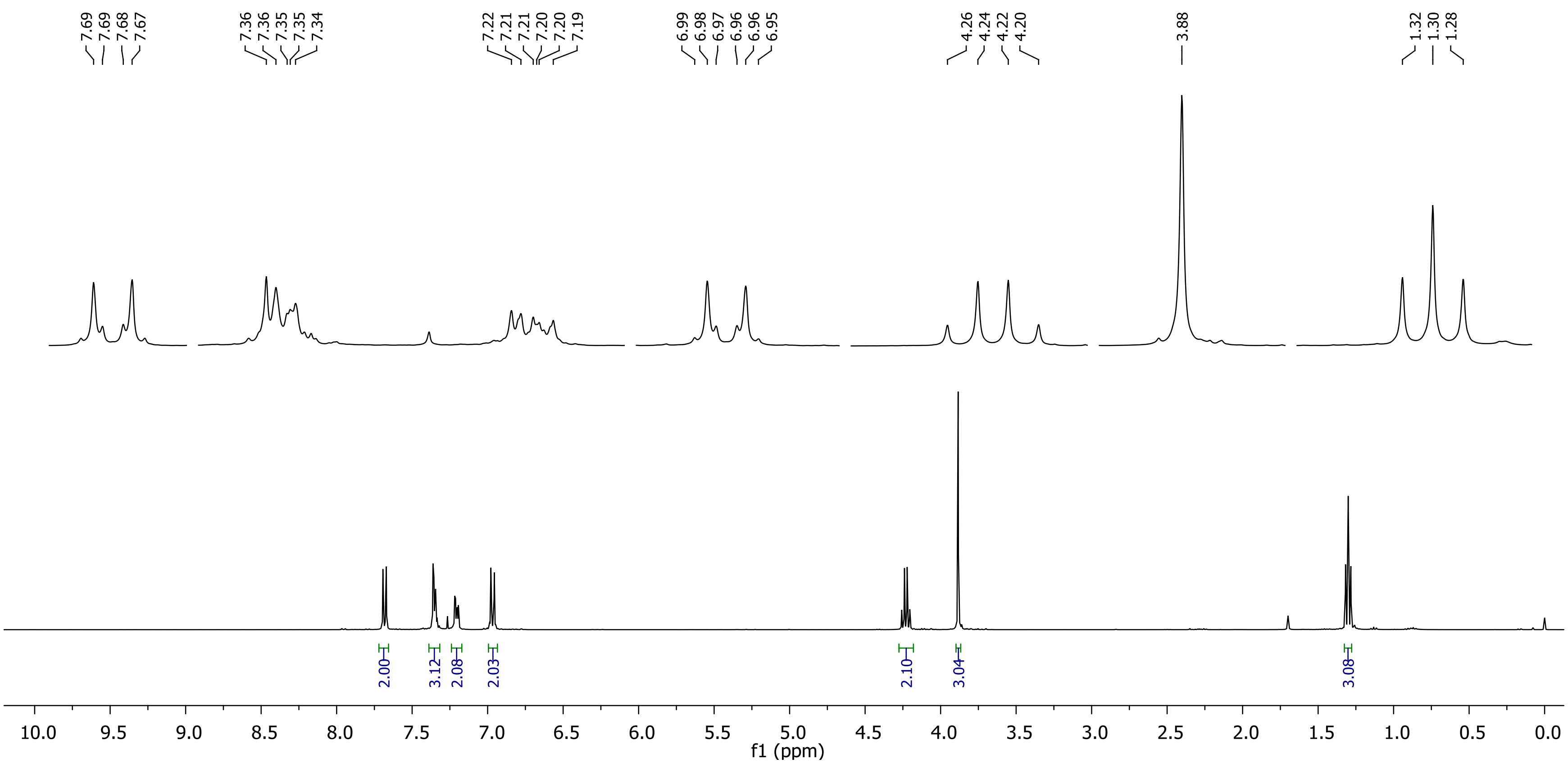
7.69
7.69
7.68
7.67
7.36
7.36
7.35
7.35
7.34
7.22
7.21
7.21
7.20
7.20
7.19
6.99
6.98
6.97
6.96
6.96
6.95
4.26
4.24
4.22
4.20
3.88
1.32
1.30
1.28
2.00
3.12
2.08
2.03
2.10
3.04
3.08
10.0
9.5
9.0
8.5
8.0
7.5
7.0
6.5
6.0
5.5
5.0
4.5
4.0
3.5
3.0
2.5
2.0
1.5
1.0
0.5
0.0
f1 (ppm)

IB371-1F1

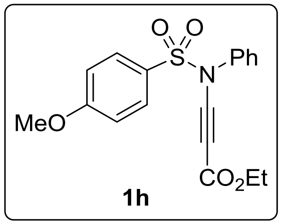
MeO
Ph
CO2Et
1h

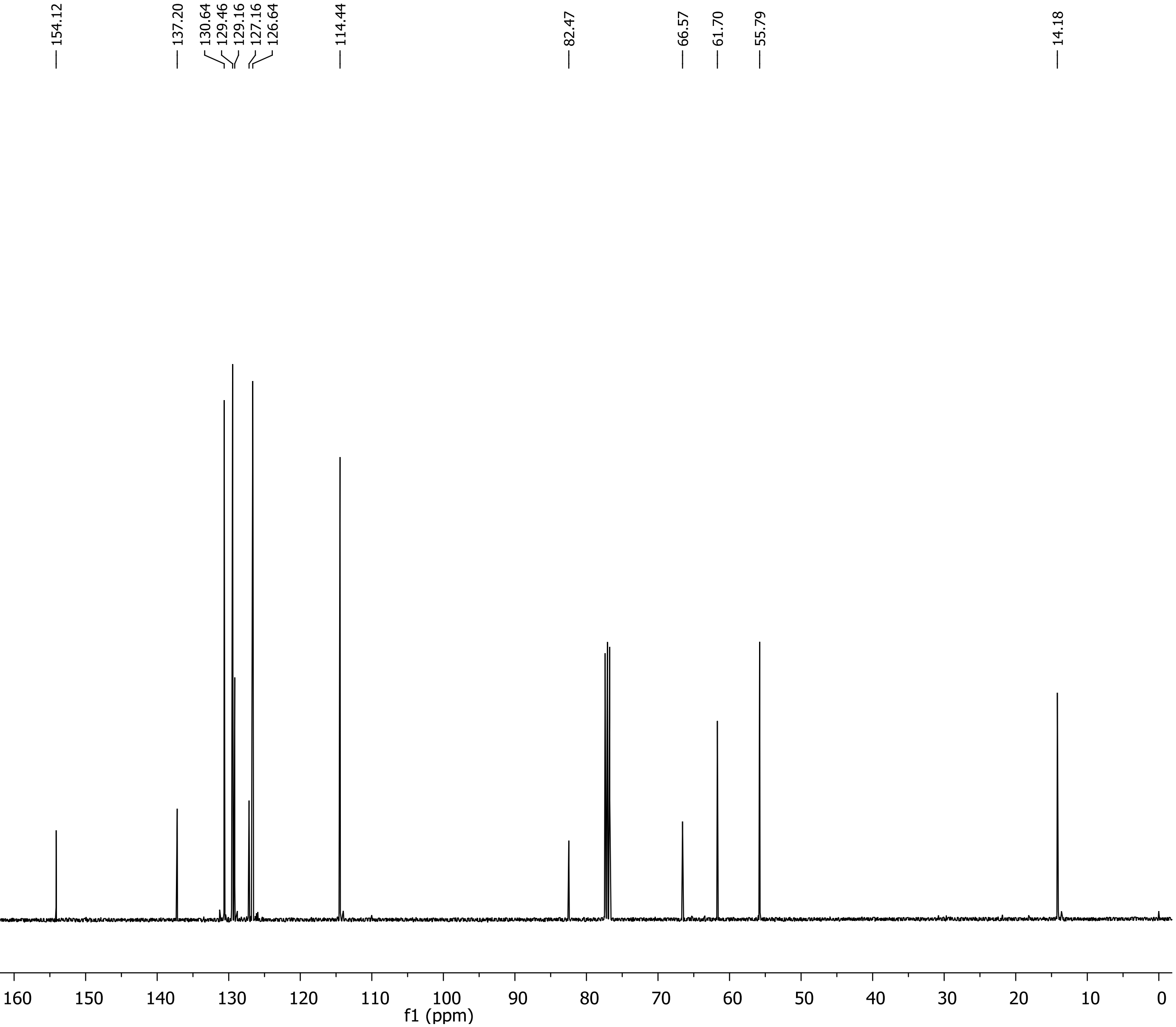
164.44
154.12
137.20
130.64
129.46
129.16
127.16
126.64
114.44
82.47
66.57
61.70
55.79
14.18
200
190
180
170
160
150
140
130
120
110
100
90
80
70
60
50
40
30
20
10
0
f1 (ppm)

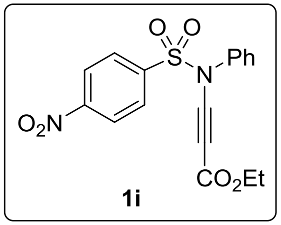
O₂N
Ph
CO₂Et
1i

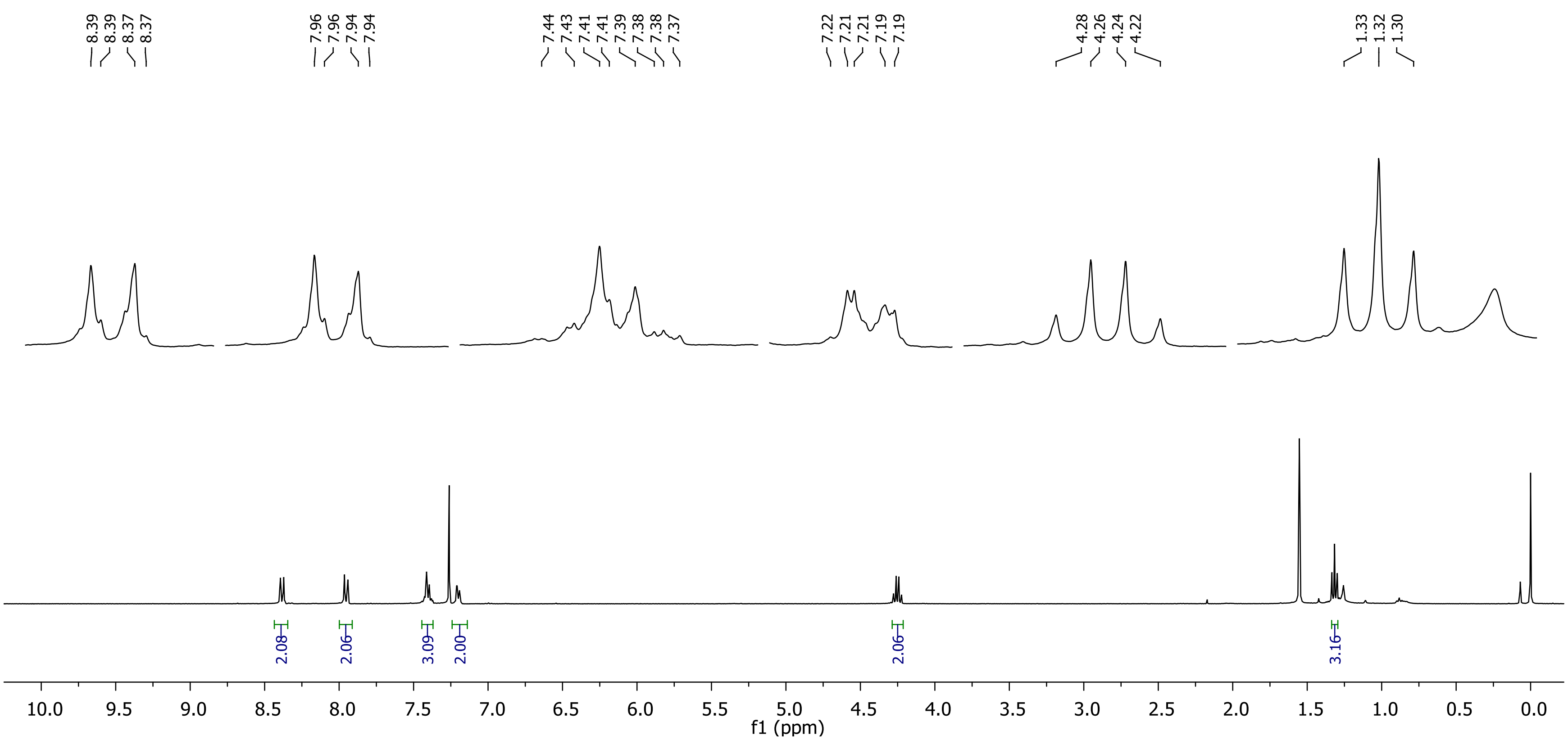
IB377-1F1
8.39 8.39 8.37 8.37
7.96 7.96 7.94 7.94
7.44 7.43 7.41 7.41 7.39 7.38 7.38 7.37
7.22 7.21 7.21 7.19 7.19
4.28 4.26 4.24 4.22
1.33 1.32 1.30
2.08 2.06 3.09 2.00 2.06 3.16
f1 (ppm)

IB377-1F1

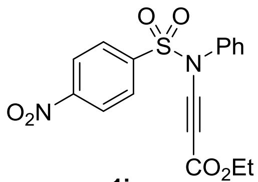
O O
S
N
Ph
O2N
CO2Et
1i

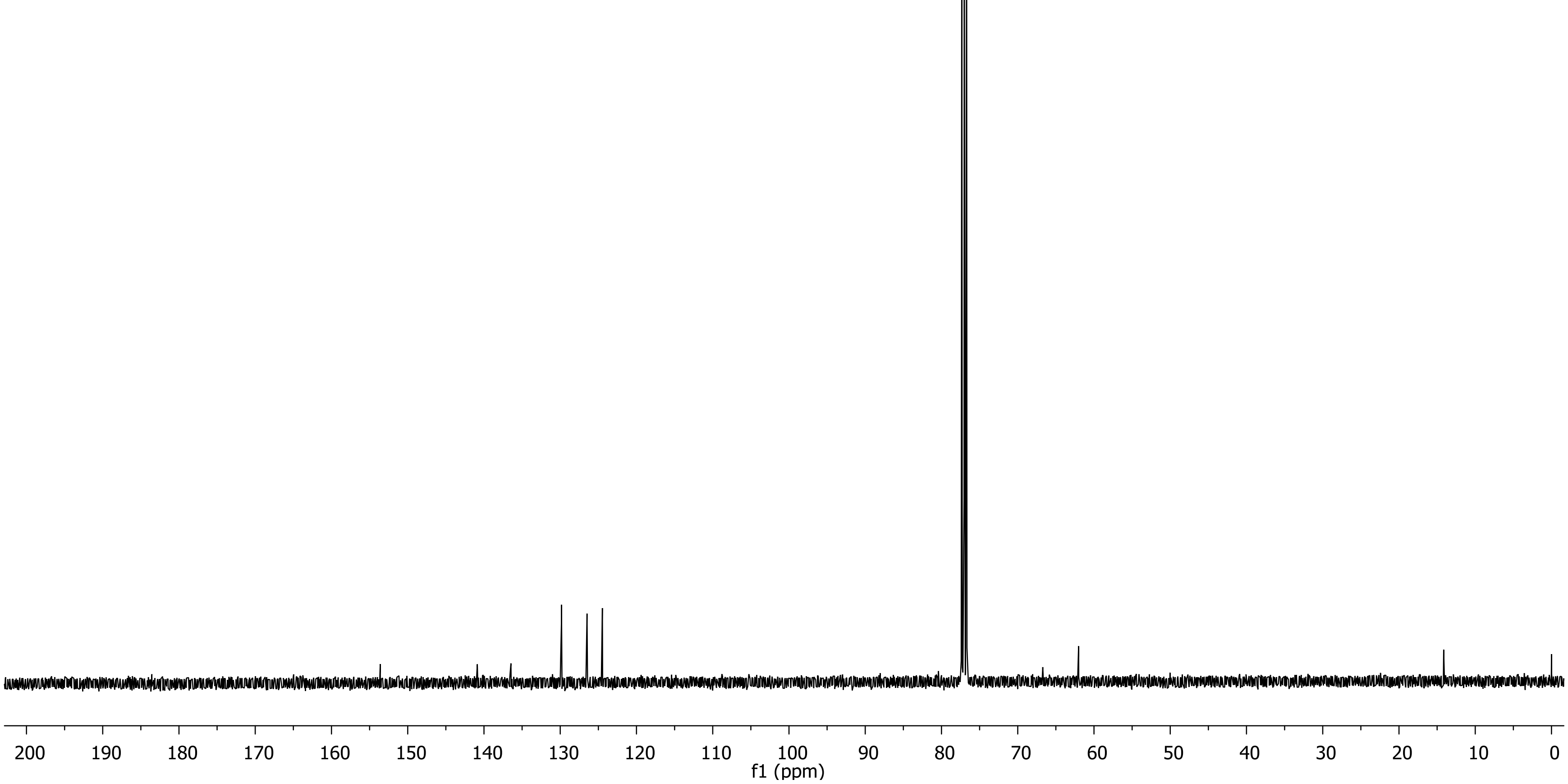
153.60
140.88
136.47
129.84
129.81
129.66
126.49
124.47
80.40
66.72
62.02
14.14
200 190 180 170 160 150 140 130 120 110 100 90 80 70 60 50 40 30 20 10 0
f1 (ppm)

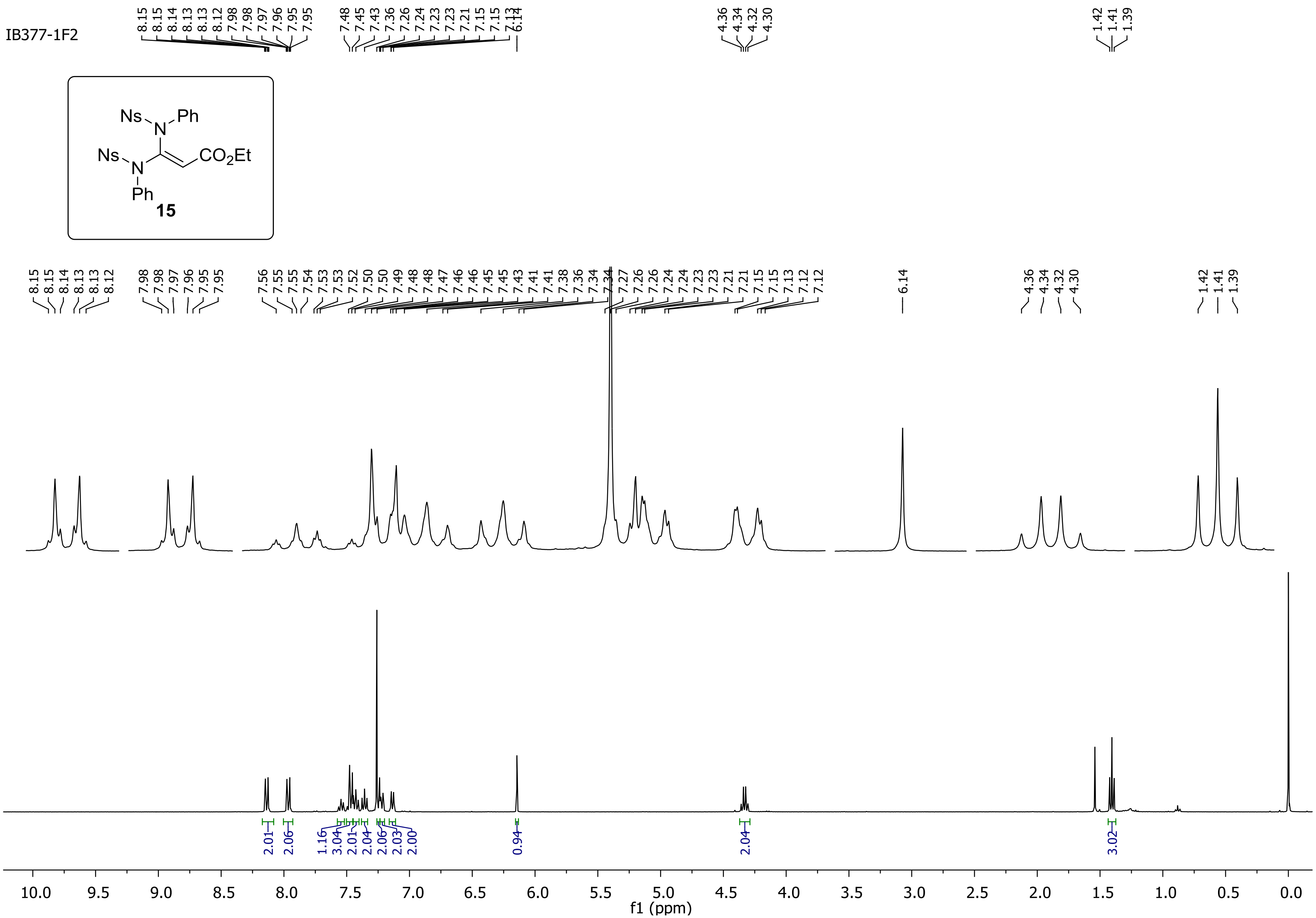
IB377-1F2
8.15 8.15 8.14 8.13 8.13 8.12 7.98 7.98 7.97 7.96 7.95 7.95
7.48 7.45 7.43 7.36 7.26 7.24 7.23 7.23 7.21 7.15 7.15 7.13 6.14
4.36 4.34 4.32 4.30
1.42 1.41 1.39
Ns
N
Ph
CO2Et
15
8.15 8.15 8.14 8.13 8.13 8.12
7.98 7.98 7.97 7.96 7.95 7.95
7.56 7.55 7.55 7.54 7.53 7.53 7.52 7.50 7.50 7.49 7.48 7.48 7.47 7.46 7.46 7.45 7.45 7.43 7.41 7.41 7.38 7.36 7.34 7.34 7.27 7.26 7.26 7.24 7.24 7.23 7.23 7.21 7.21 7.15 7.15 7.13 7.12 7.12
6.14
4.36 4.34 4.32 4.30
1.42 1.41 1.39
2.01 2.06 1.16 3.04 2.01 2.04 2.06 2.03 2.00 0.94 2.04 3.02
10.0 9.5 9.0 8.5 8.0 7.5 7.0 6.5 6.0 5.5 5.0 4.5 4.0 3.5 3.0 2.5 2.0 1.5 1.0 0.5 0.0
f1 (ppm)

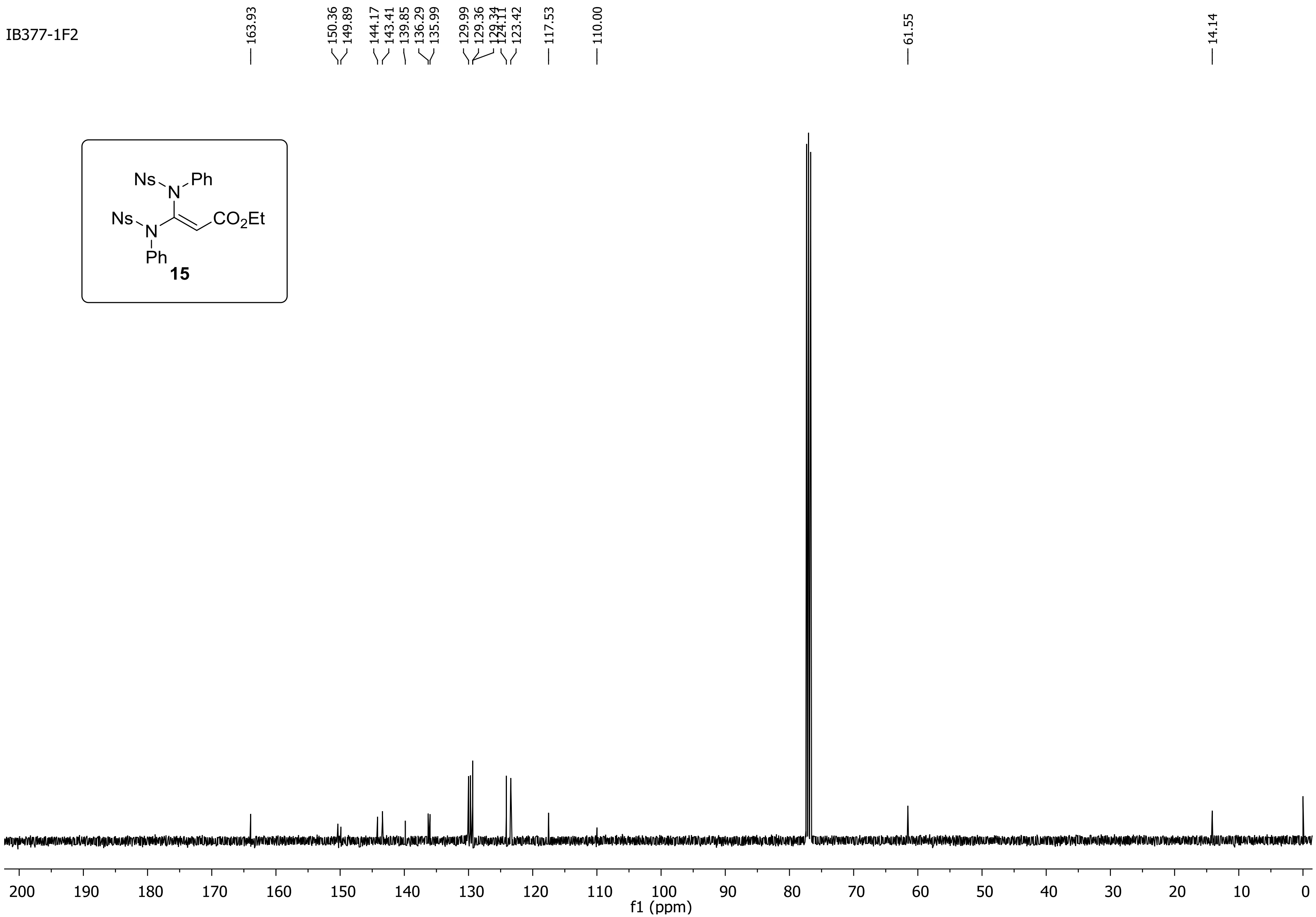
IB377-1F2
163.93
150.36
149.89
144.17
143.41
139.85
136.29
135.99
129.99
129.36
129.34
124.11
123.42
117.53
110.00
61.55
14.14
Ns
N
Ph
Ns
N
Ph
CO_2Et
15
200 190 180 170 160 150 140 130 120 110 100 90 80 70 60 50 40 30 20 10 0
f1 (ppm)

Intramolecular Hydroarylation of Sulfonyl Ynamine 1a. Products (*E*)-2a, (*Z*)-2a, 4 and 5

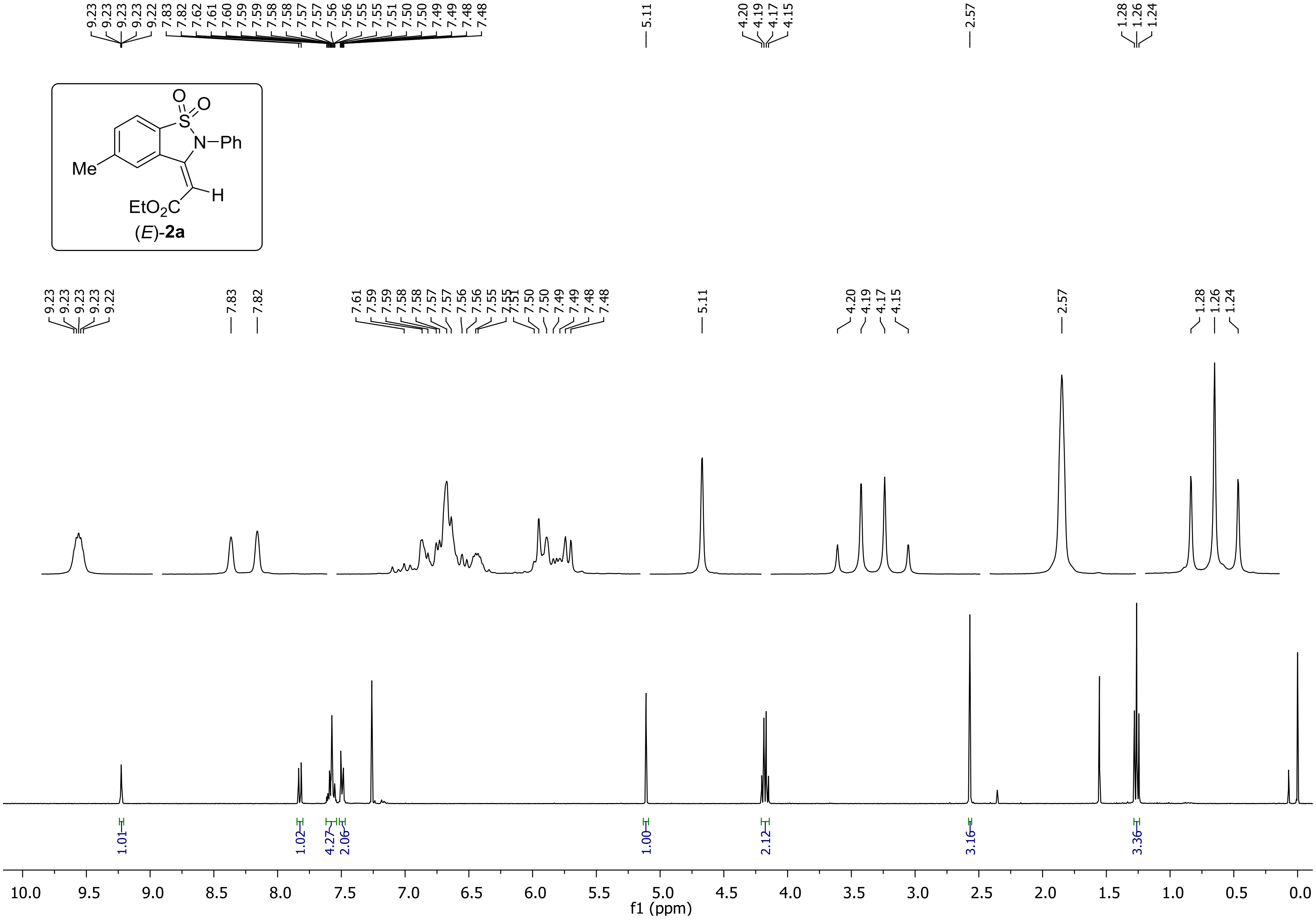

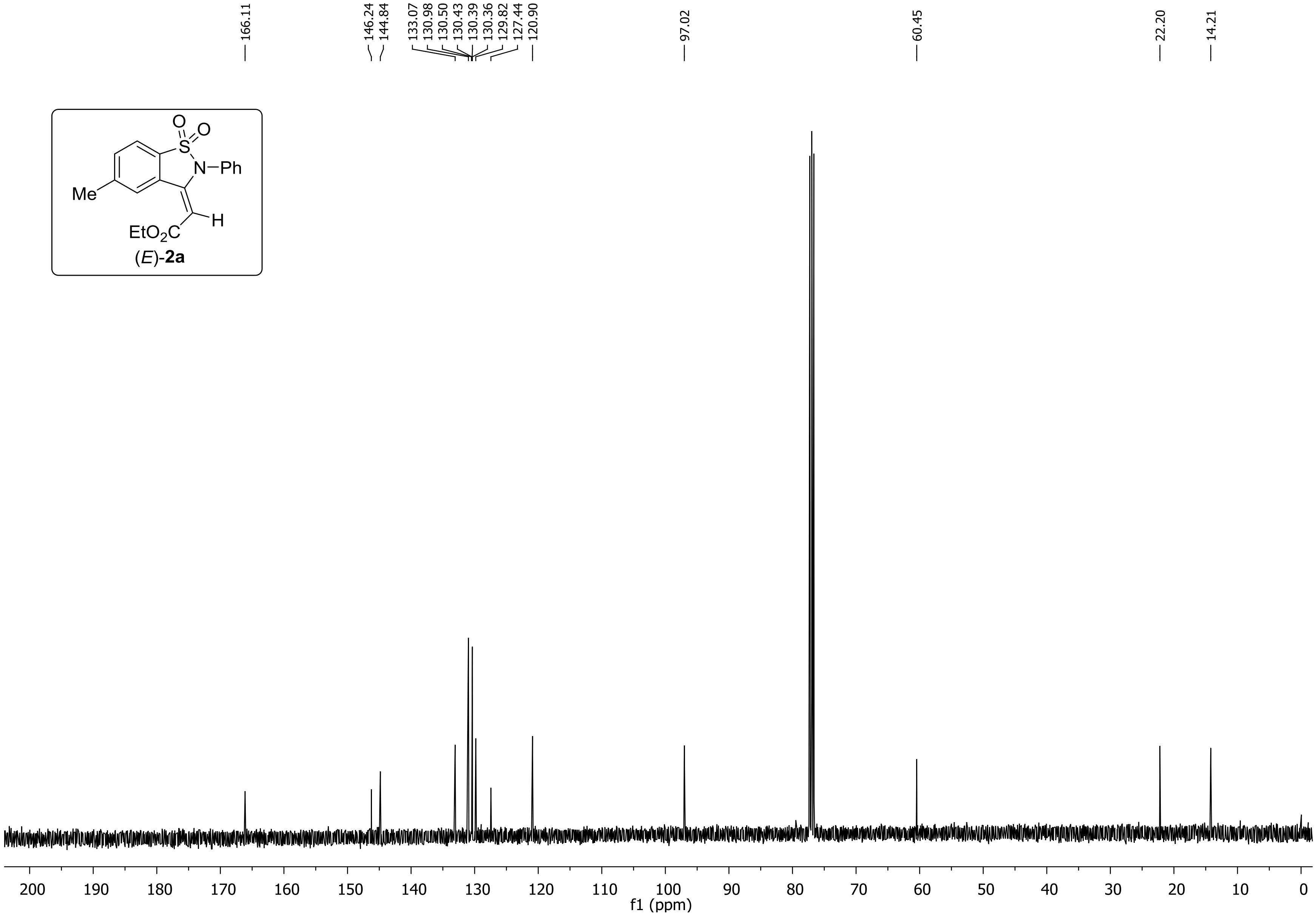
166.11
146.24
144.84
133.07
130.98
130.50
130.43
130.39
130.36
129.82
127.44
120.90
97.02
60.45
22.20
14.21
O
O
S
N−Ph
Me
EtO2C
H
(E)-2a
200
190
180
170
160
150
140
130
120
110
100
90
80
70
60
50
40
30
20
10
0
f1 (ppm)

NOESY/25
for sel1d
1D Selective Gradient NOESY
freq: 5.132ppm 1 1

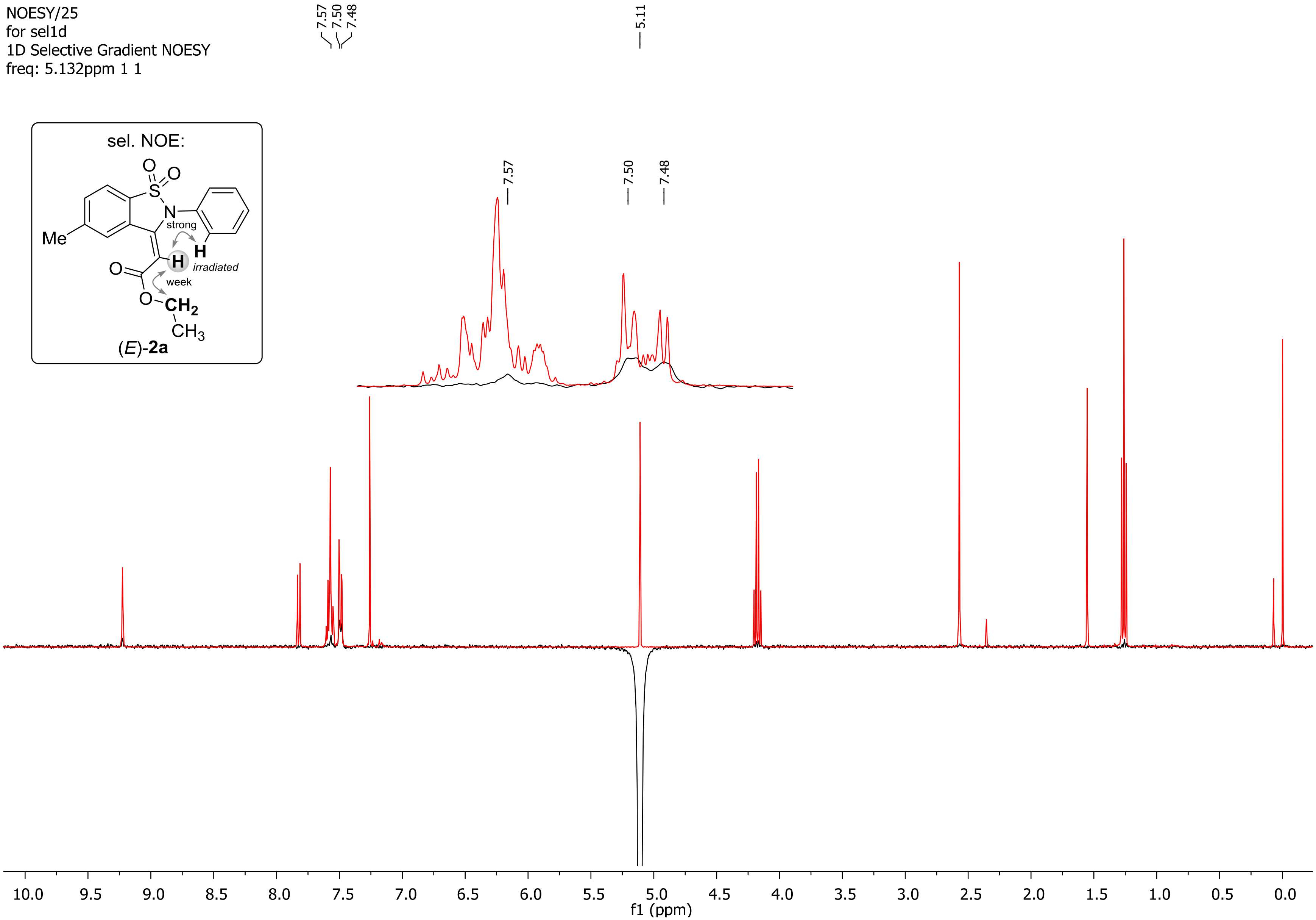

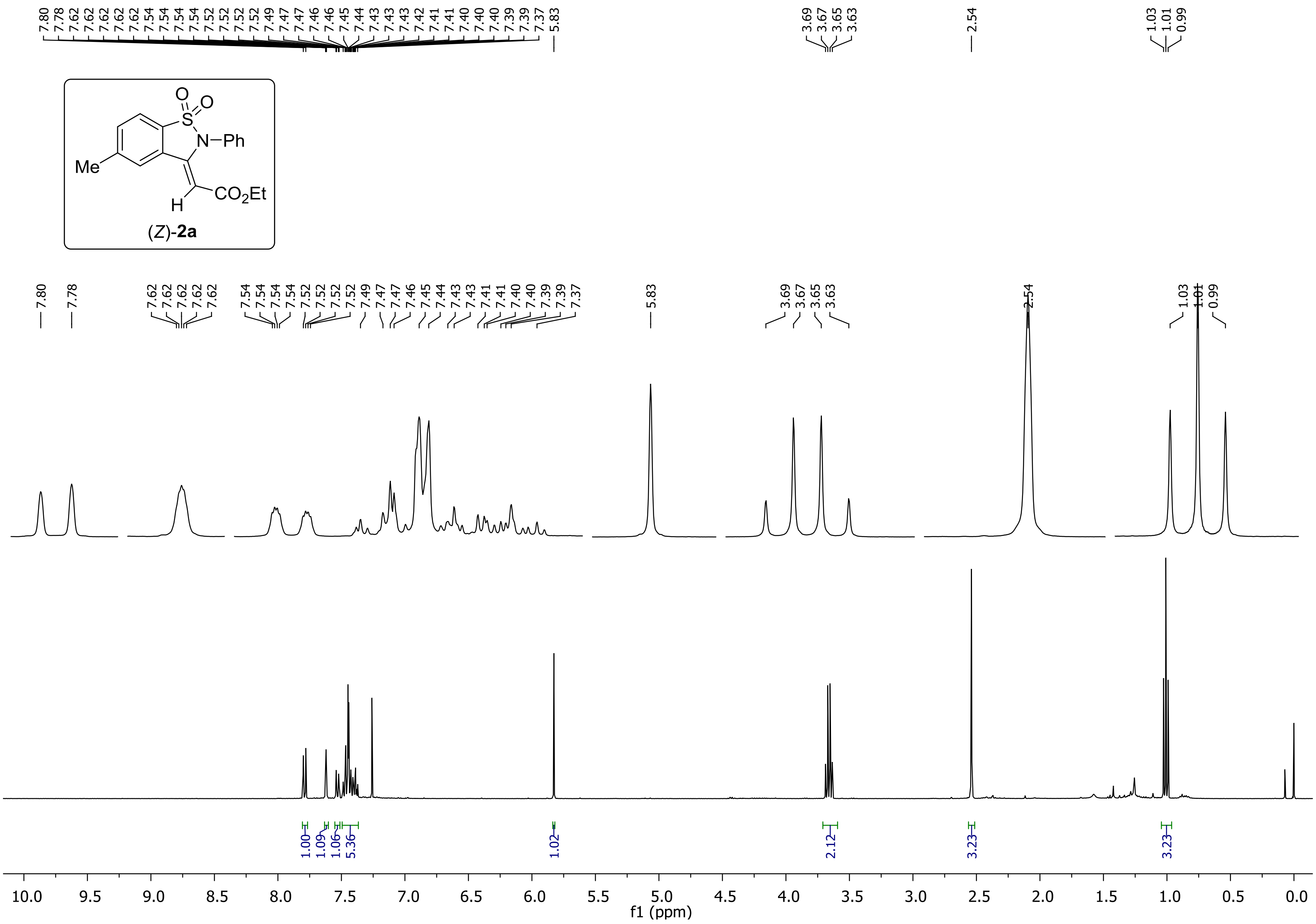
7.80 7.78 7.62 7.62 7.62 7.62 7.62 7.54 7.54 7.54 7.54 7.52 7.52 7.52 7.52 7.49 7.47 7.47 7.46 7.45 7.44 7.43 7.43 7.41 7.41 7.40 7.40 7.39 7.39 7.37 5.83
3.69 3.67 3.65 3.63
2.54
1.03 1.01 0.99
O
O
S
N−Ph
Me
H
CO2Et
(Z)-2a
1.00
1.09
1.06
5.36
1.02
2.12
3.23
3.23
10.0 9.5 9.0 8.5 8.0 7.5 7.0 6.5 6.0 5.5 5.0 4.5 4.0 3.5 3.0 2.5 2.0 1.5 1.0 0.5 0.0
f1 (ppm)

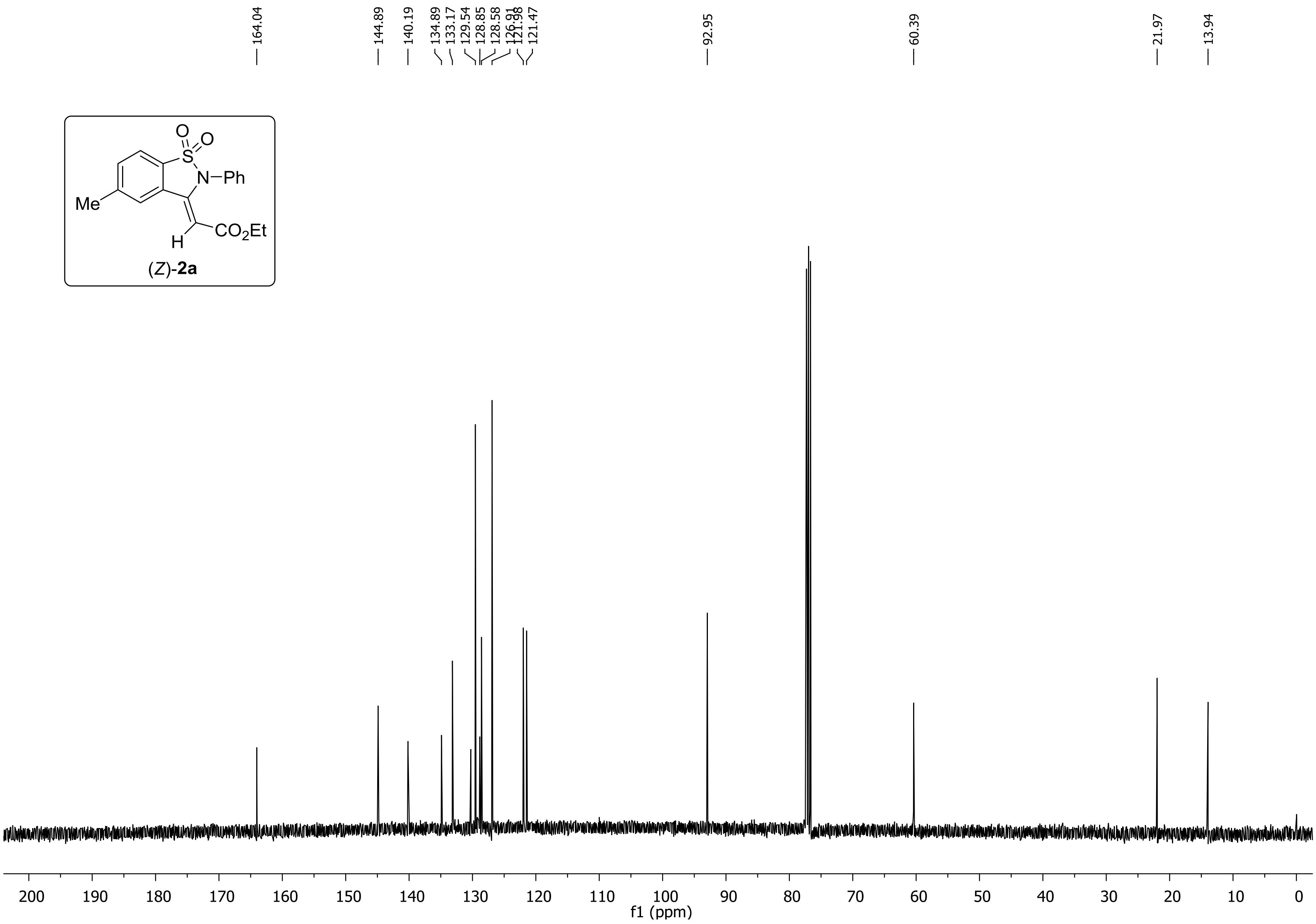
164.04
144.89
140.19
134.89
133.17
129.54
128.85
128.58
126.91
121.98
121.47
92.95
60.39
21.97
13.94
O
O
S
N−Ph
Me
H
CO_2Et
(Z)-2a
200 190 180 170 160 150 140 130 120 110 100 90 80 70 60 50 40 30 20 10 0
f1 (ppm)

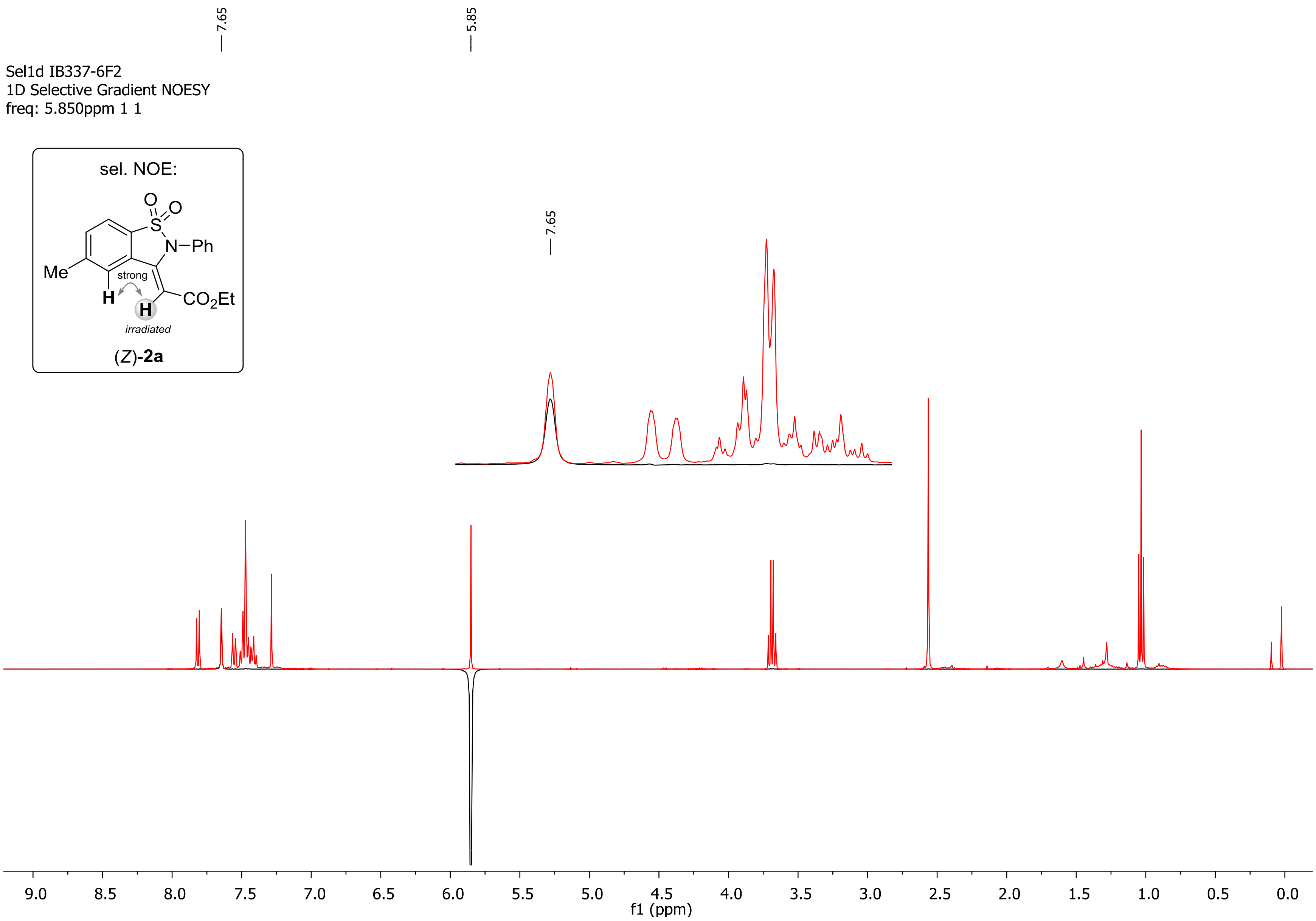

7.65
5.85
Sel1d IB337-6F2
1D Selective Gradient NOESY
freq: 5.850ppm 1 1
sel. NOE:
O
O
S
N−Ph
Me
strong
H
H
CO2Et
irradiated
(Z)-2a
7.65
9.0 8.5 8.0 7.5 7.0 6.5 6.0 5.5 5.0 4.5 4.0 3.5 3.0 2.5 2.0 1.5 1.0 0.5 0.0
f1 (ppm)

IB355-4

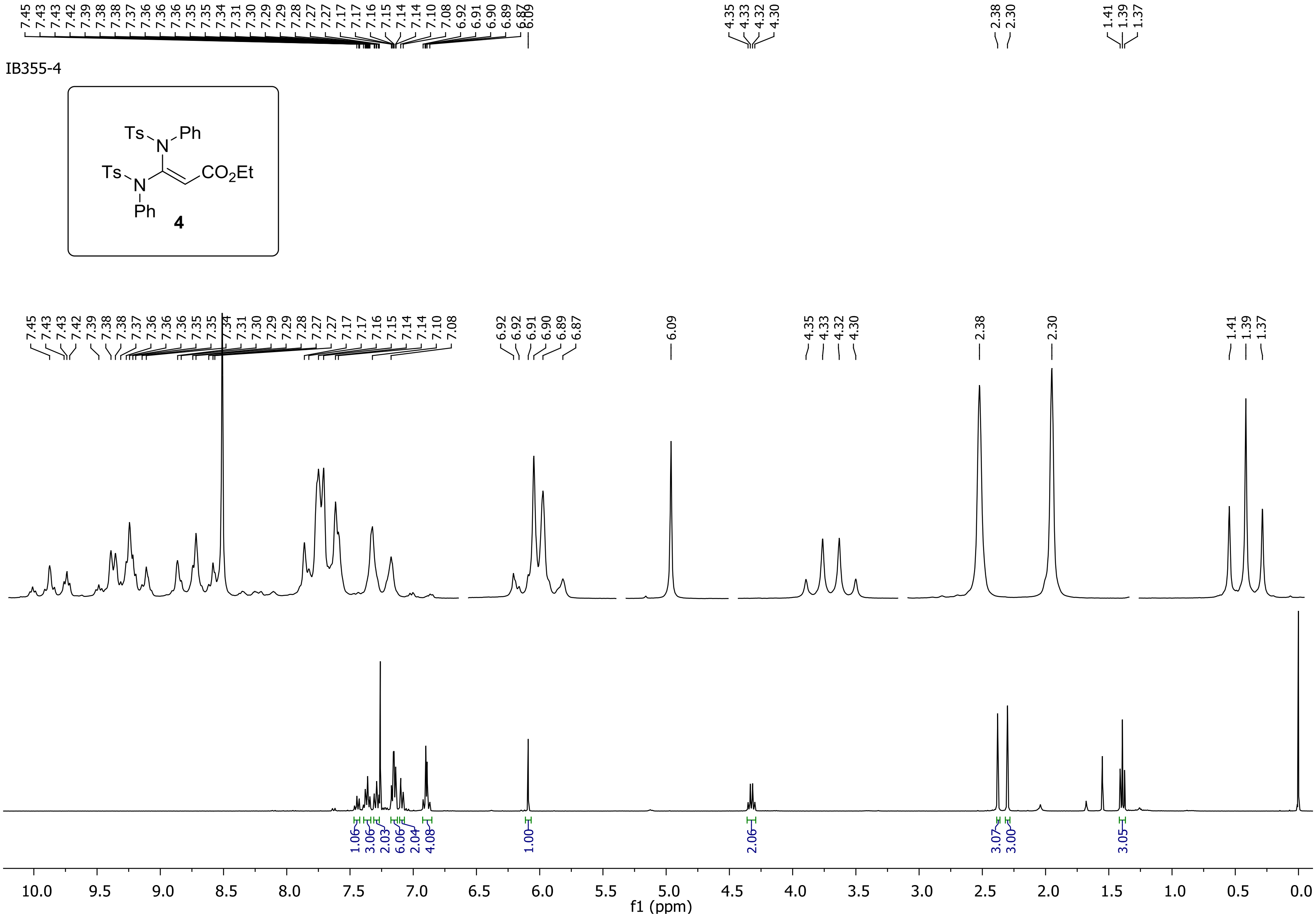

IB355-4

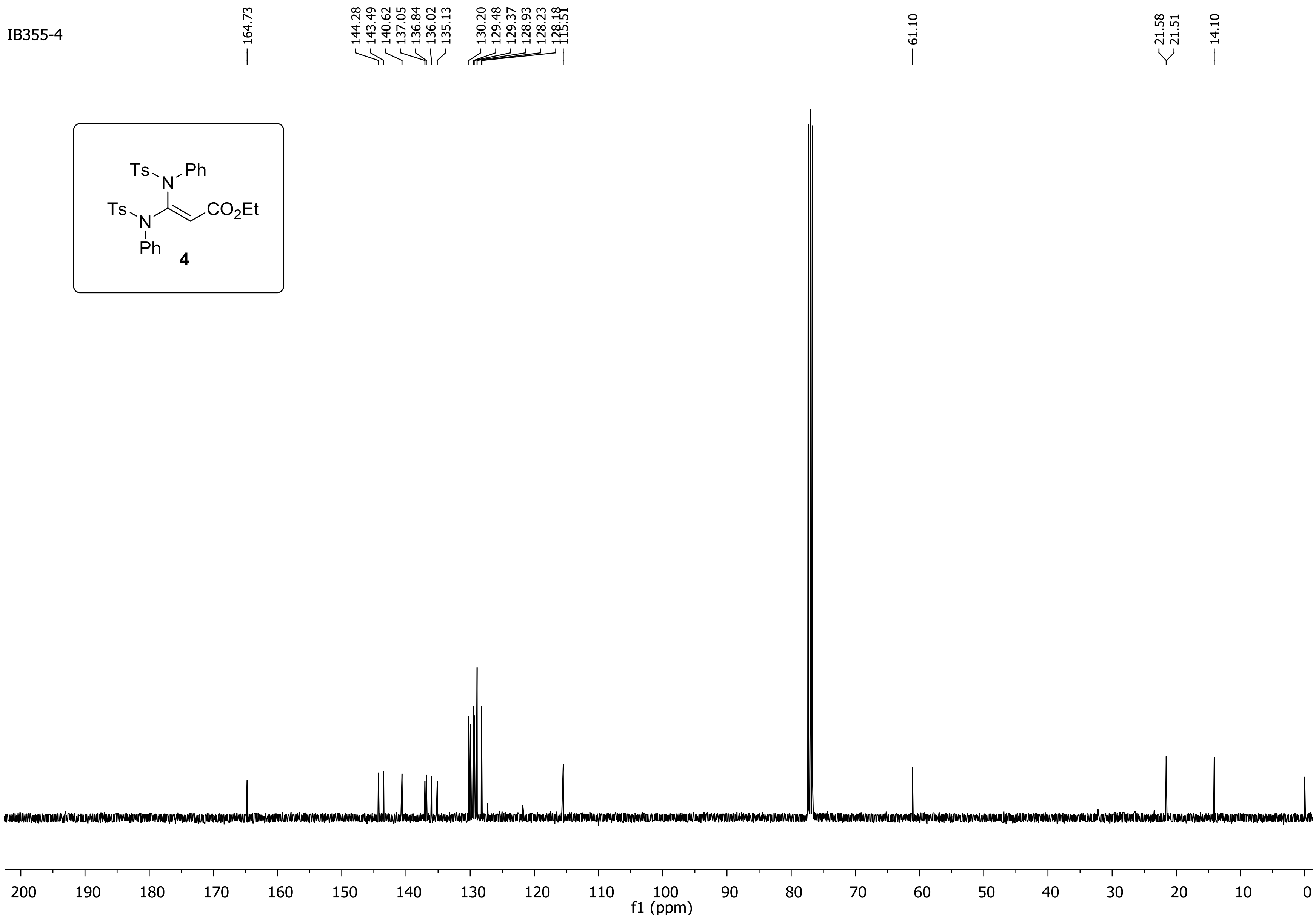

Ts
N
Ph
Ts
N
CO2Et
Ph
4
164.73
144.28
143.49
140.62
137.05
136.84
136.02
135.13
130.20
129.48
129.37
128.93
128.23
128.18
115.51
61.10
21.58
21.51
14.10
200 190 180 170 160 150 140 130 120 110 100 90 80 70 60 50 40 30 20 10 0
f1 (ppm)

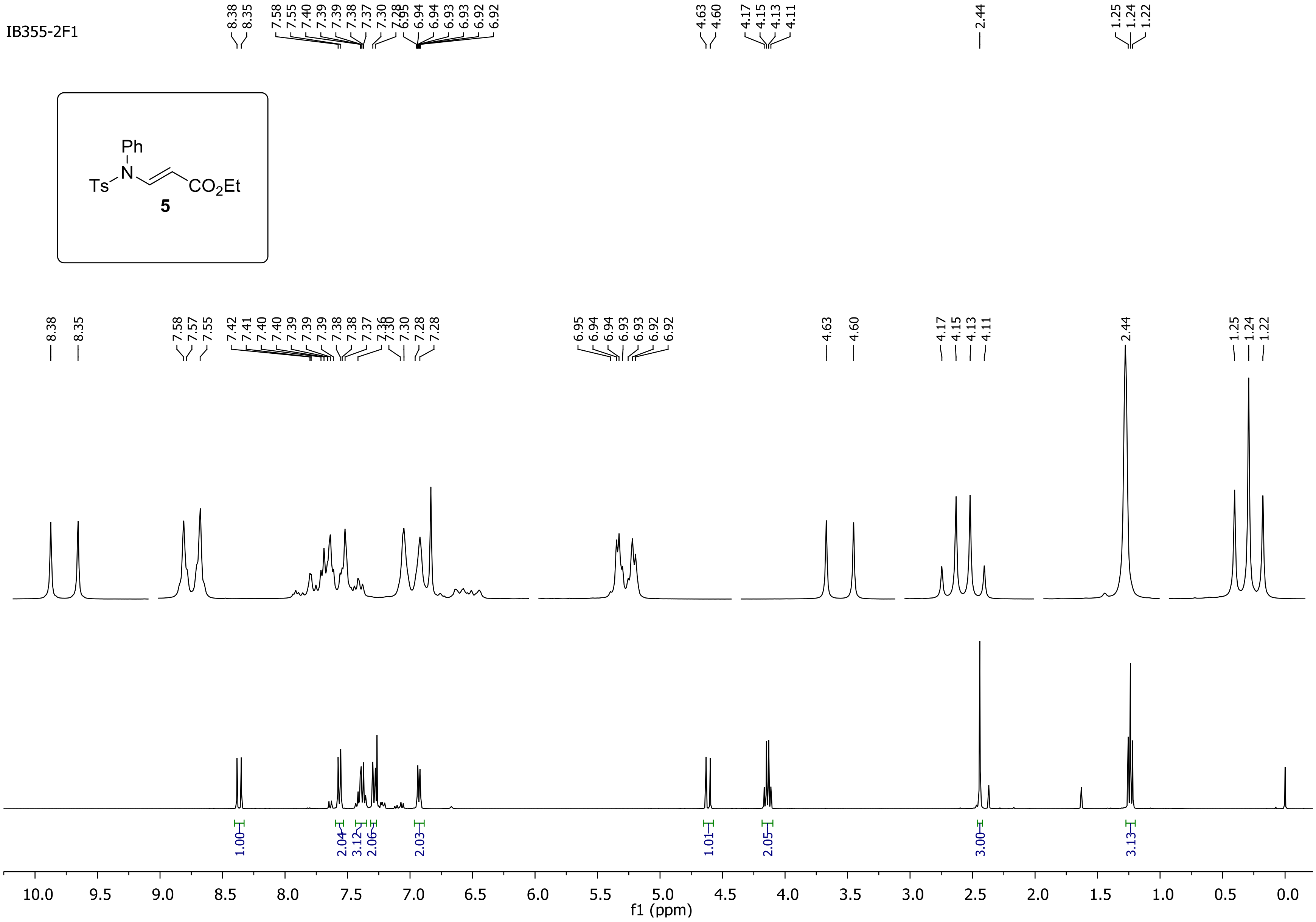

IB355-2F1
Ph
N
Ts
CO2Et
5
f1 (ppm)

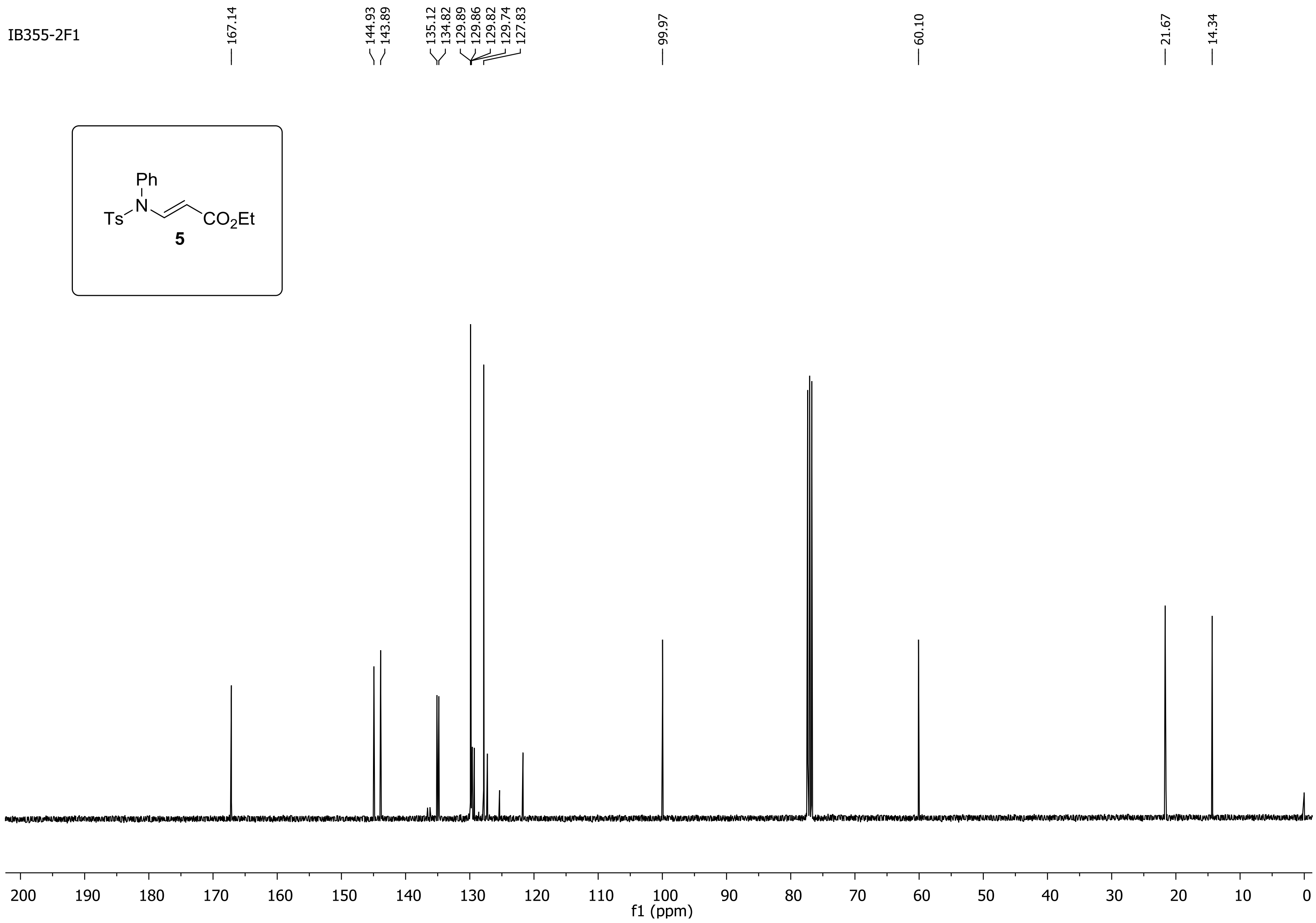
IB355-2F1
167.14
144.93
143.89
135.12
134.82
129.89
129.86
129.82
129.74
127.83
99.97
60.10
21.67
14.34
Ph
N
Ts
CO2Et
5
200 190 180 170 160 150 140 130 120 110 100 90 80 70 60 50 40 30 20 10 0
f1 (ppm)

Intramolecular Hydroarylation of Sulfonyl Ynamines. Products (*E*)-2b–k and 6

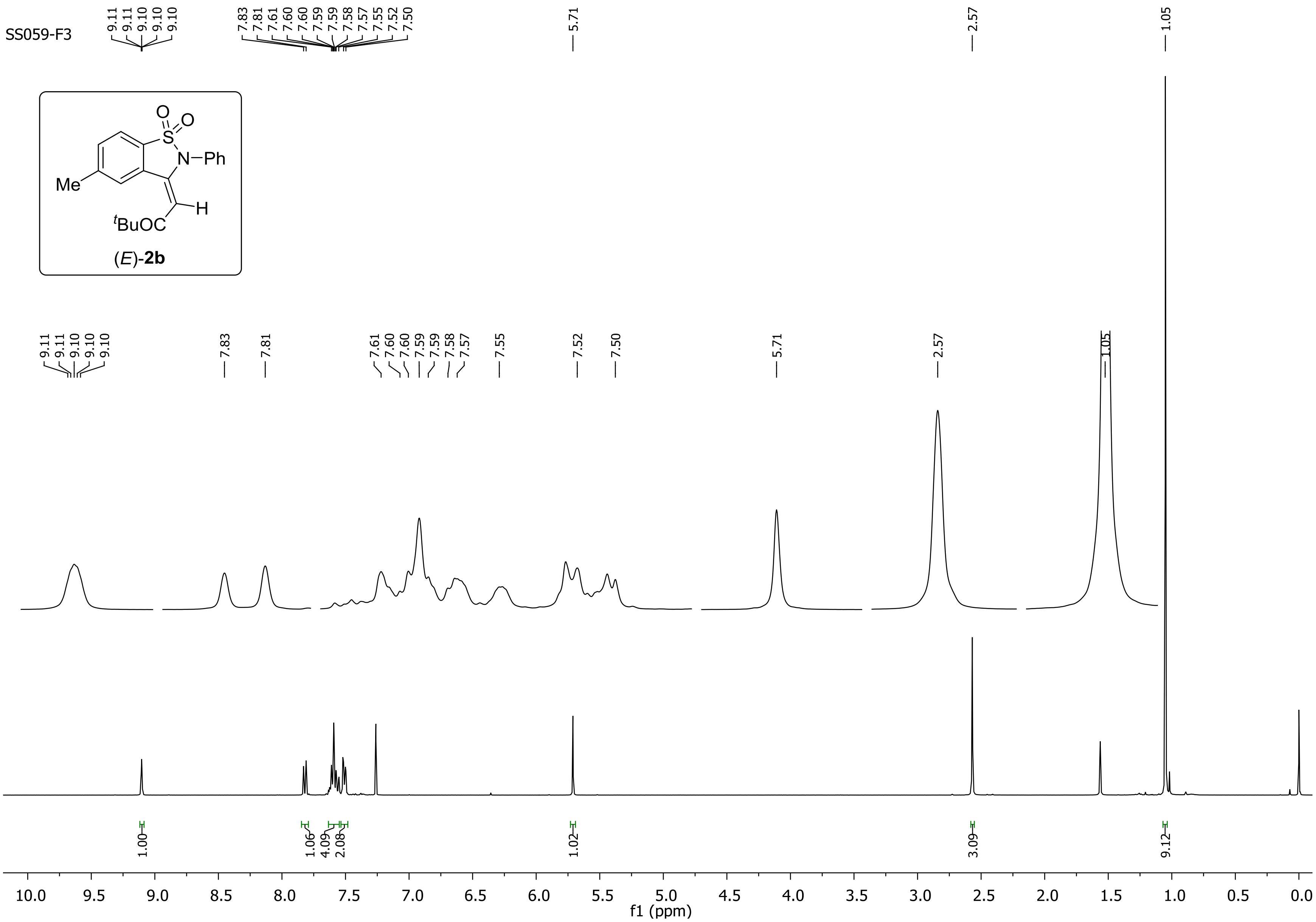

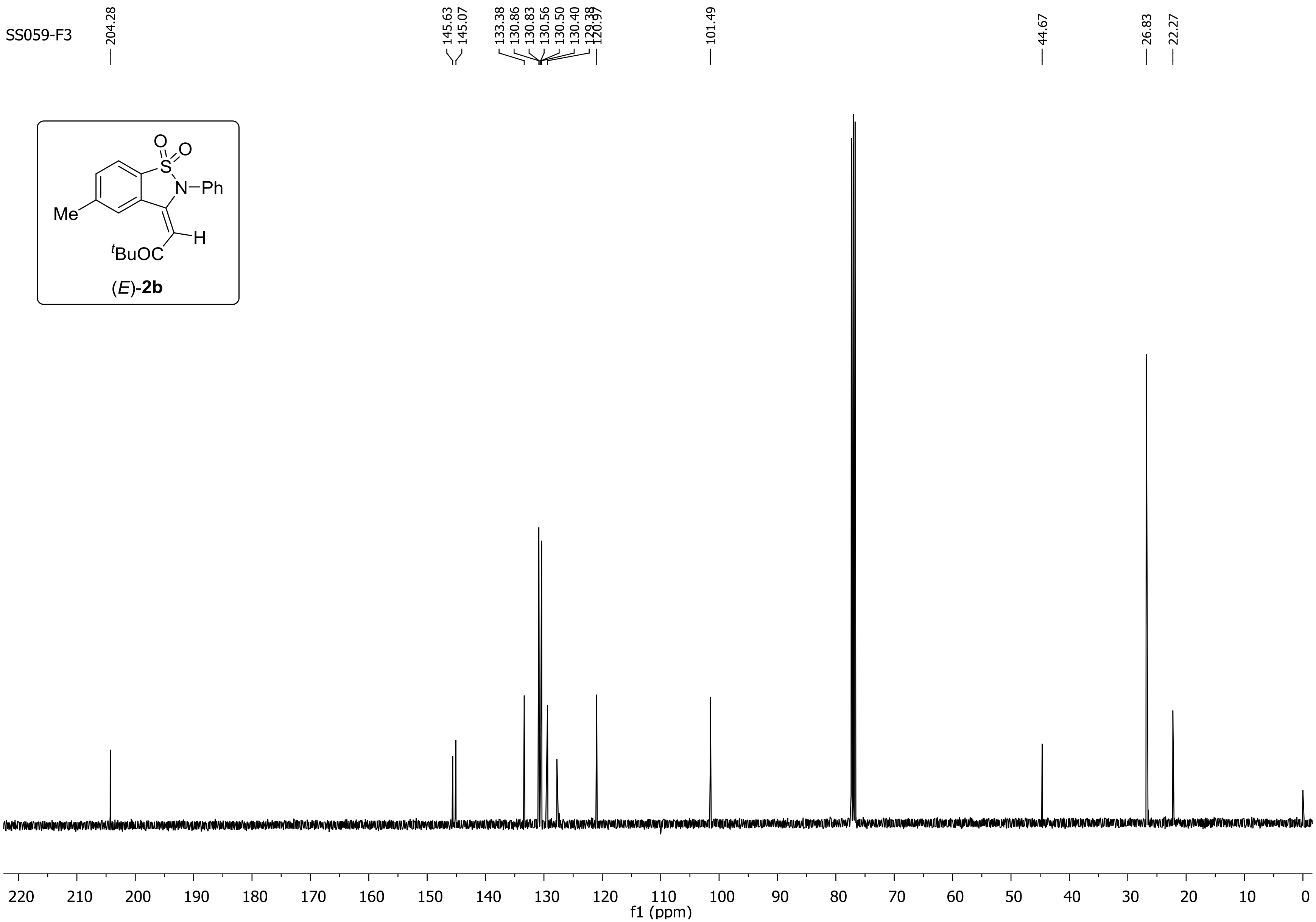
SS059-F3
204.28
145.63
145.07
133.38
130.86
130.83
130.56
130.50
130.40
120.97
101.49
44.67
26.83
22.27
Me
N−Ph
H
tBuOC
(E)-2b
f1 (ppm)

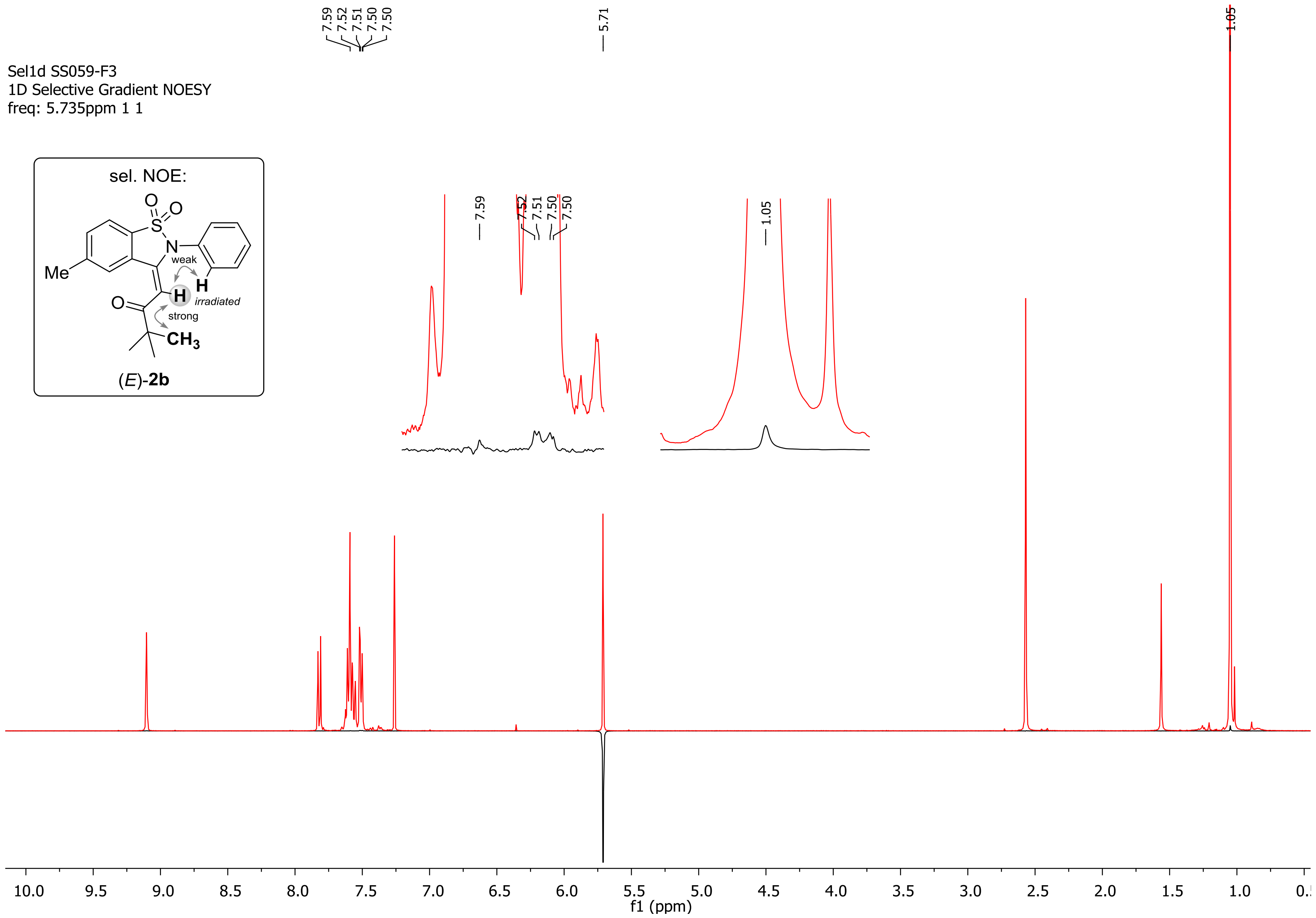
7.59
7.52
7.51
7.50
7.50
5.71
1.05
Sel1d SS059-F3
1D Selective Gradient NOESY
freq: 5.735ppm 1 1
sel. NOE:
O
O
S
N
weak
Me
H
H
irradiated
O
strong
CH3
(E)-2b
7.59
7.52
7.51
7.50
7.50
1.05
10.0
9.5
9.0
8.5
8.0
7.5
7.0
6.5
6.0
5.5
5.0
4.5
4.0
3.5
3.0
2.5
2.0
1.5
1.0
f1 (ppm)

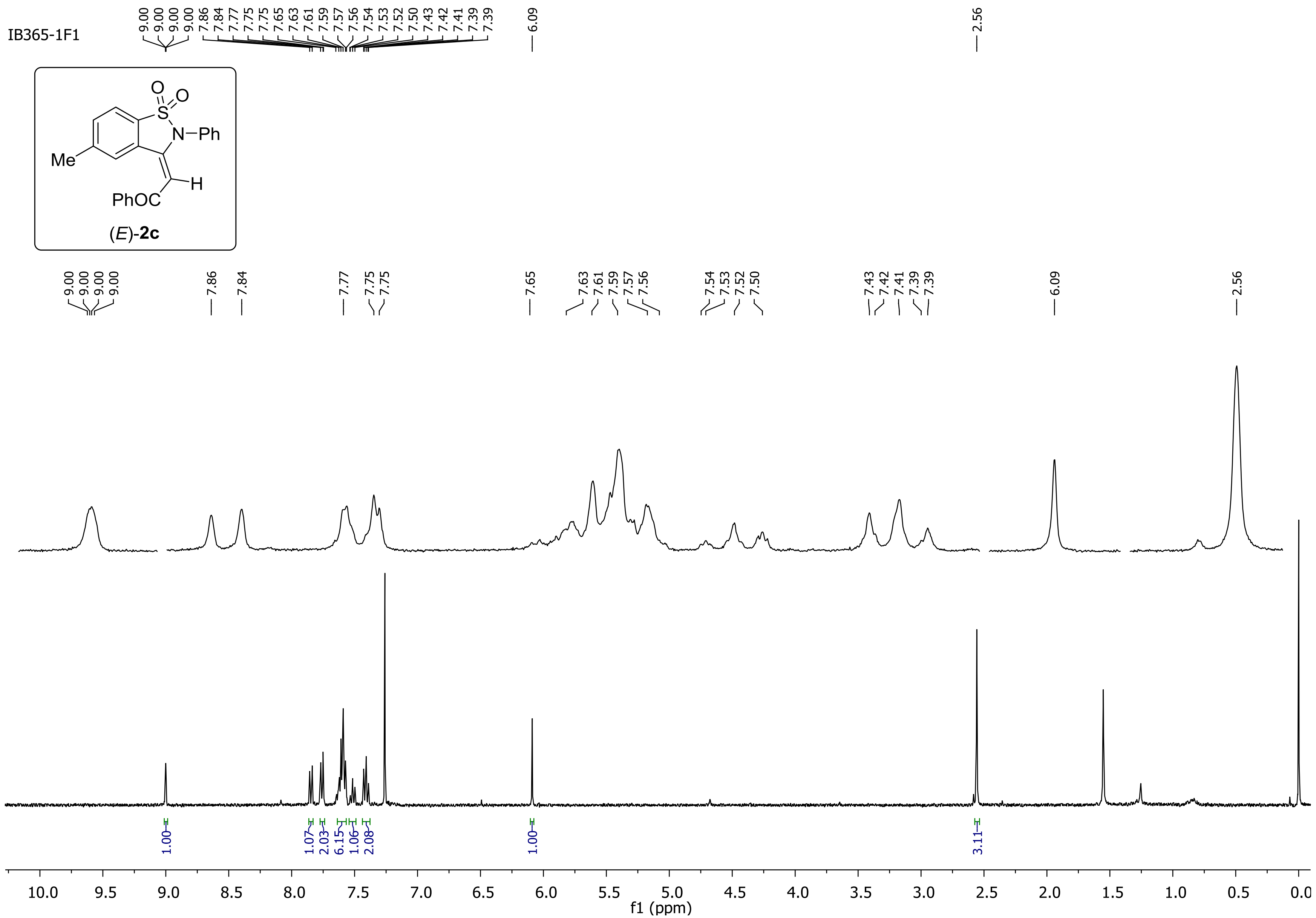

IB365-1F1
9.00
9.00
9.00
9.00
7.86
7.84
7.77
7.75
7.75
7.65
7.63
7.61
7.59
7.57
7.56
7.54
7.53
7.52
7.50
7.43
7.42
7.41
7.39
7.39
6.09
2.56
Me
N−Ph
PhOC
H
(E)-2c
1.00
1.07
2.03
6.15
1.06
2.08
1.00
3.11
10.0
9.5
9.0
8.5
8.0
7.5
7.0
6.5
6.0
5.5
5.0
4.5
4.0
3.5
3.0
2.5
2.0
1.5
1.0
0.5
0.0
f1 (ppm)

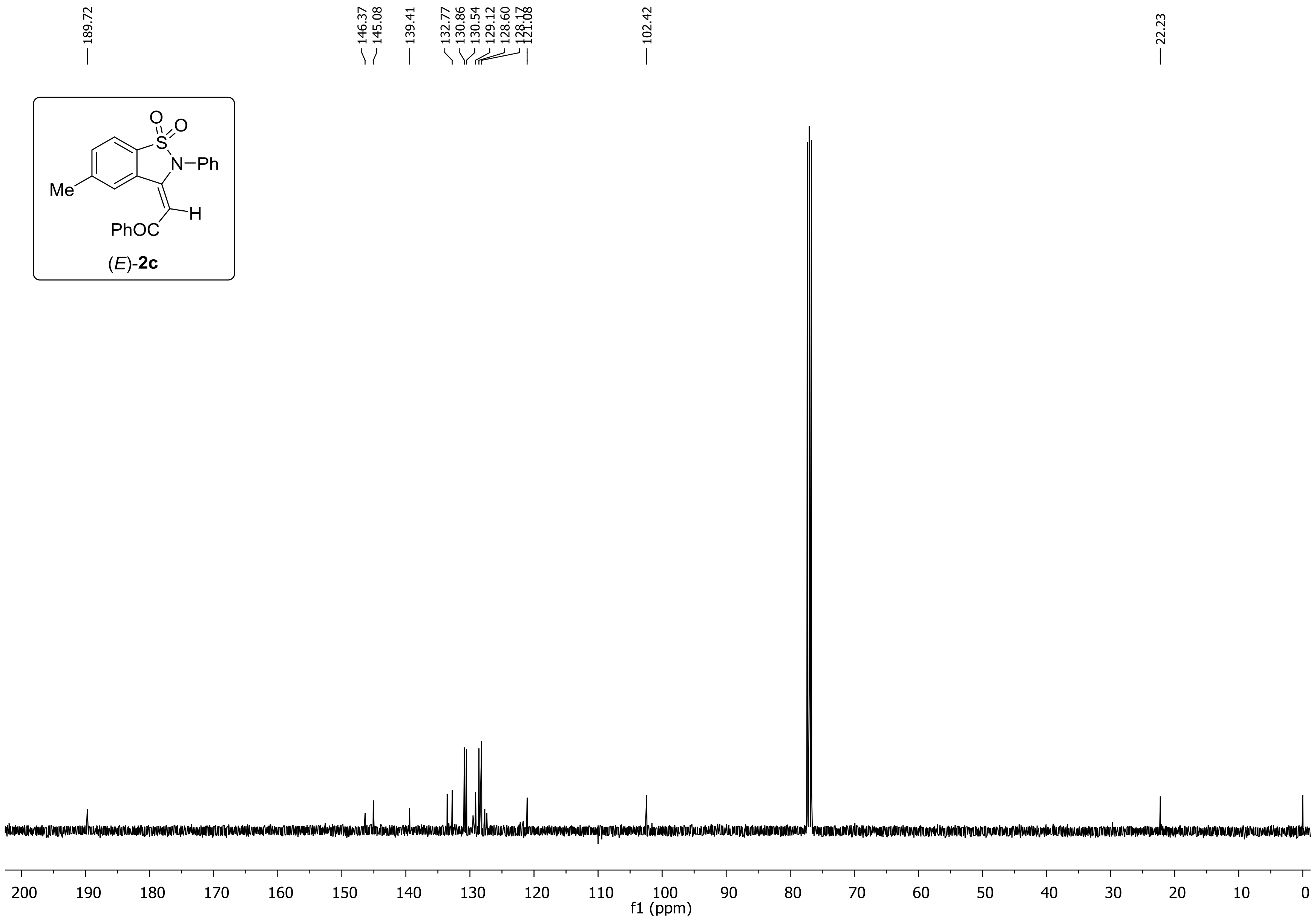
189.72
146.37
145.08
139.41
132.77
130.86
130.54
129.12
128.60
128.17
121.08
102.42
22.23
Me
N−Ph
PhOC
H
(E)-2c
200
190
180
170
160
150
140
130
120
110
100
90
80
70
60
50
40
30
20
10
0
f1 (ppm)

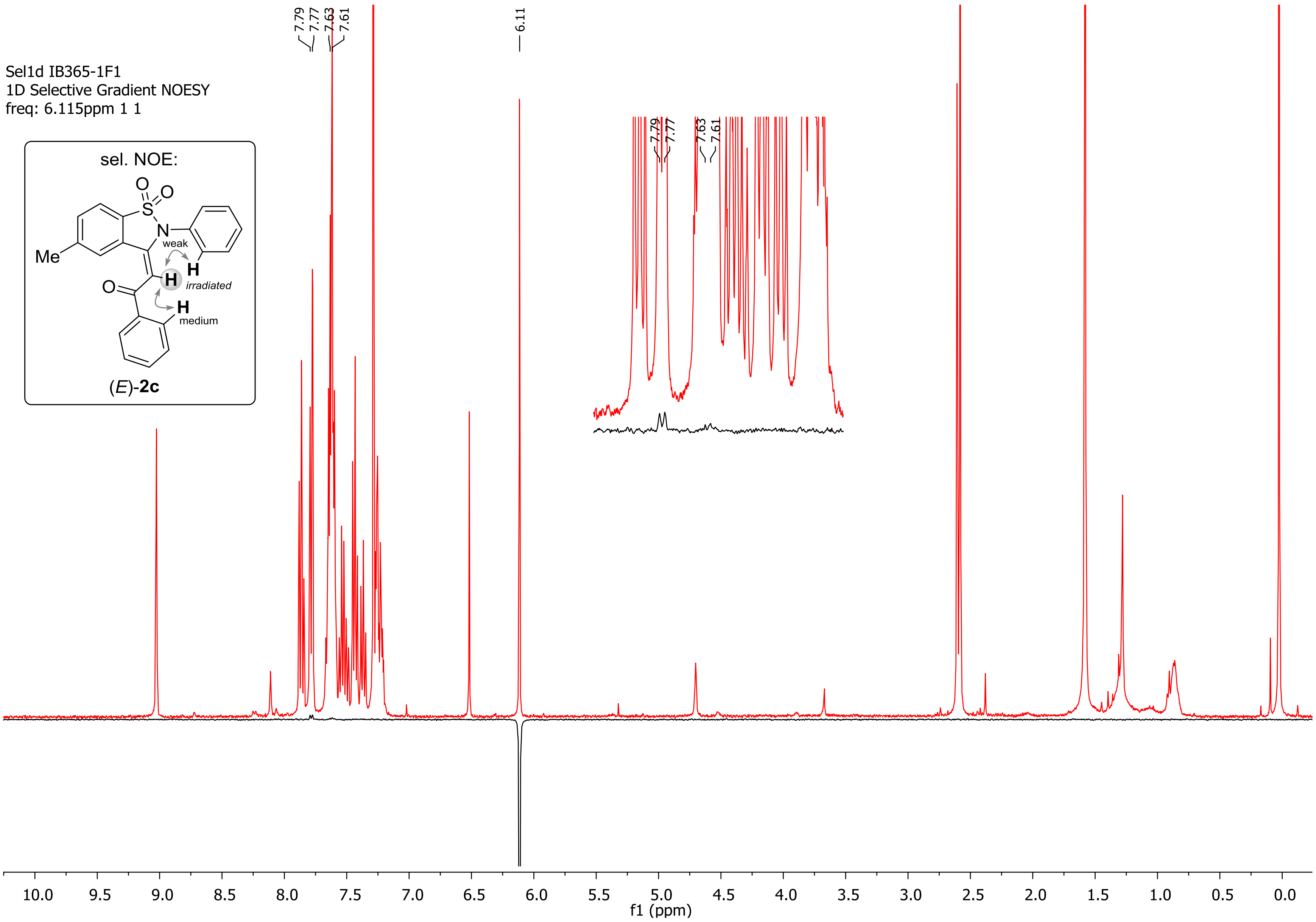

Sel1d IB365-1F1
1D Selective Gradient NOESY
freq: 6.115ppm 1 1
sel. NOE:
weak
irradiated
medium
(E)-2c
7.79
7.77
7.63
7.61
6.11
f1 (ppm)

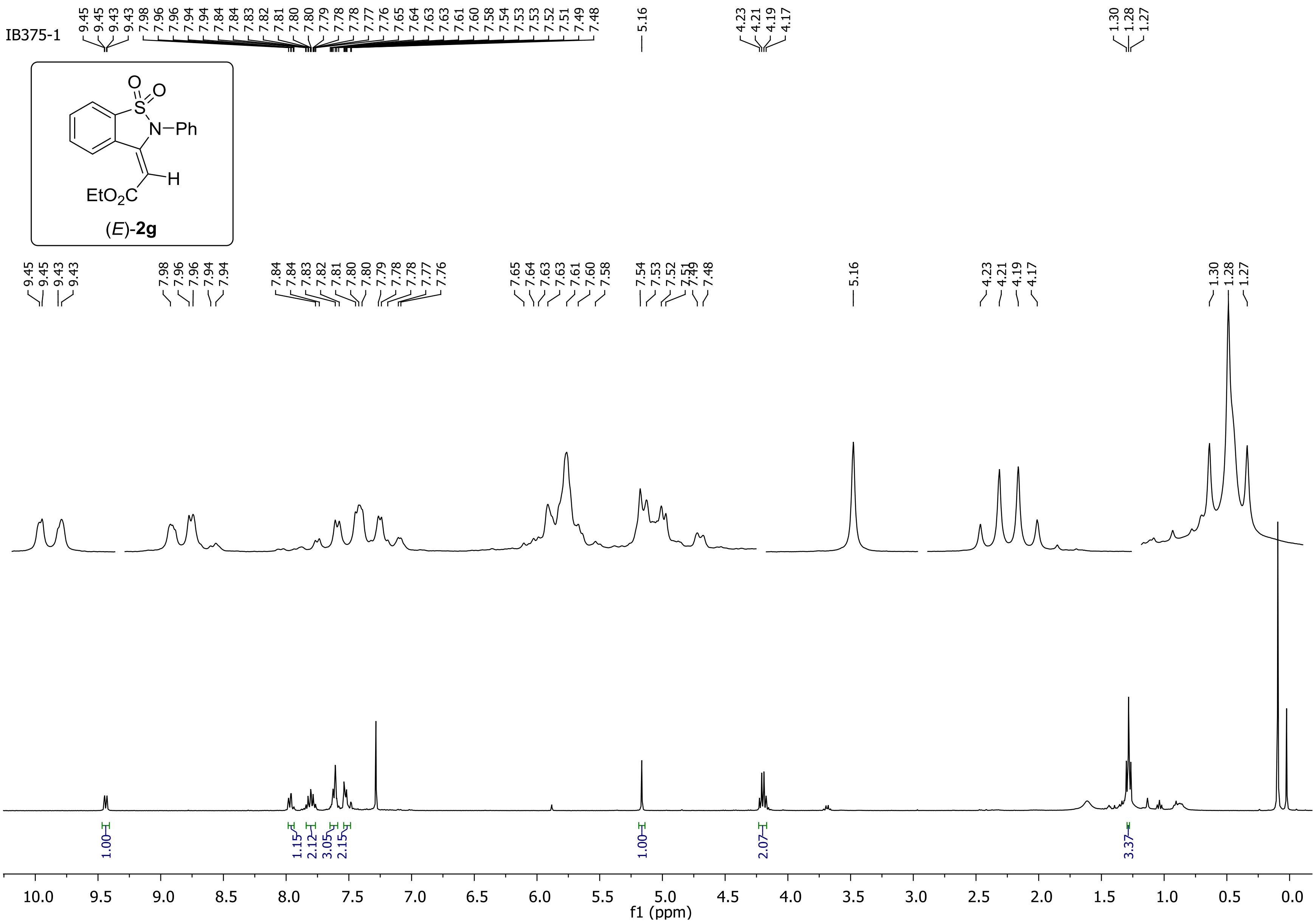
IB375-1
N–Ph
H
EtO2C
(E)-2g
1.00
1.15
2.12
3.05
2.15
1.00
2.07
3.37
f1 (ppm)

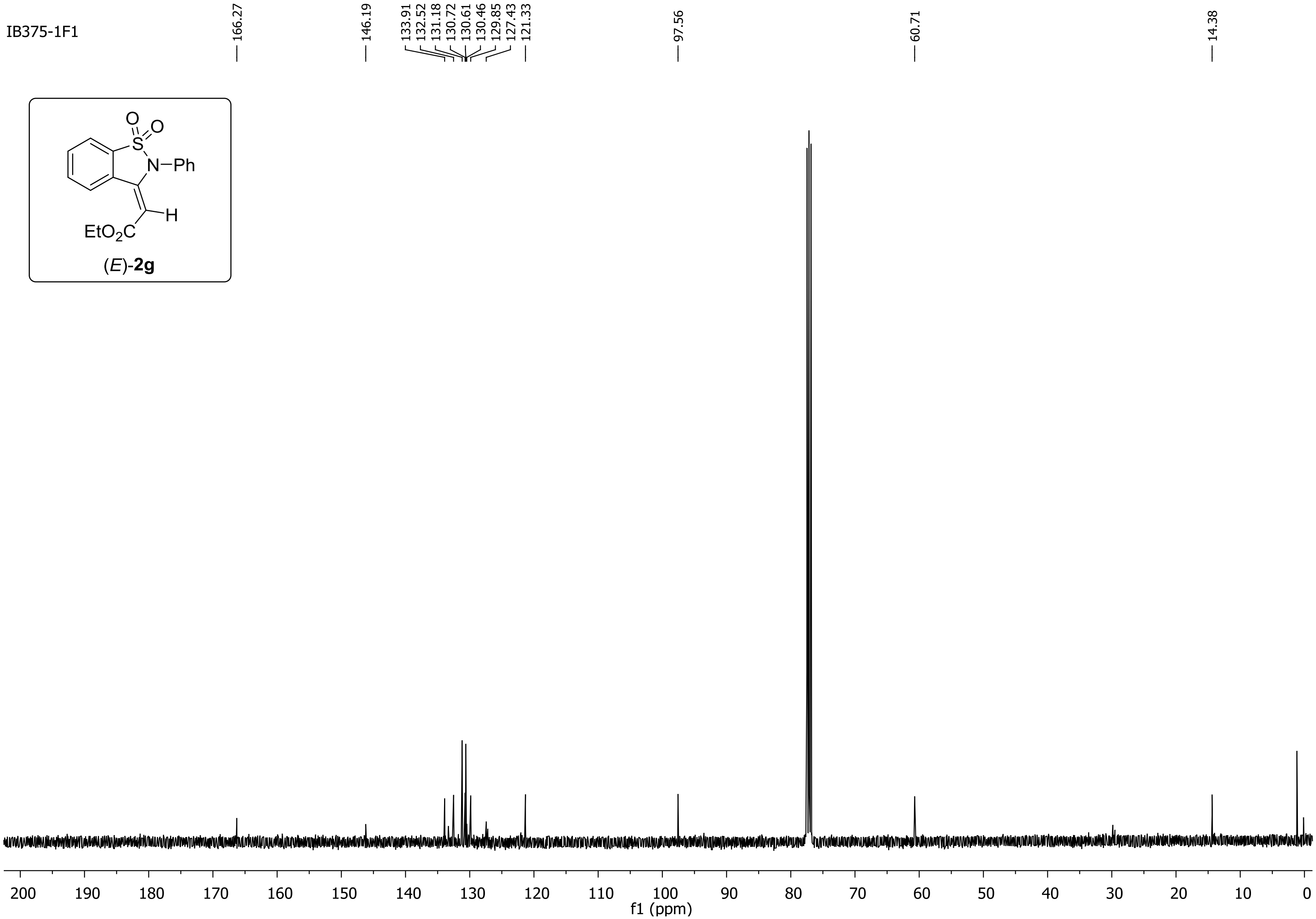

IB375-1F1
166.27
146.19
133.91
132.52
131.18
130.72
130.61
130.46
129.85
127.43
121.33
97.56
60.71
14.38
O
O
S
N−Ph
H
EtO2C
(E)-2g
200 190 180 170 160 150 140 130 120 110 100 90 80 70 60 50 40 30 20 10 0
f1 (ppm)

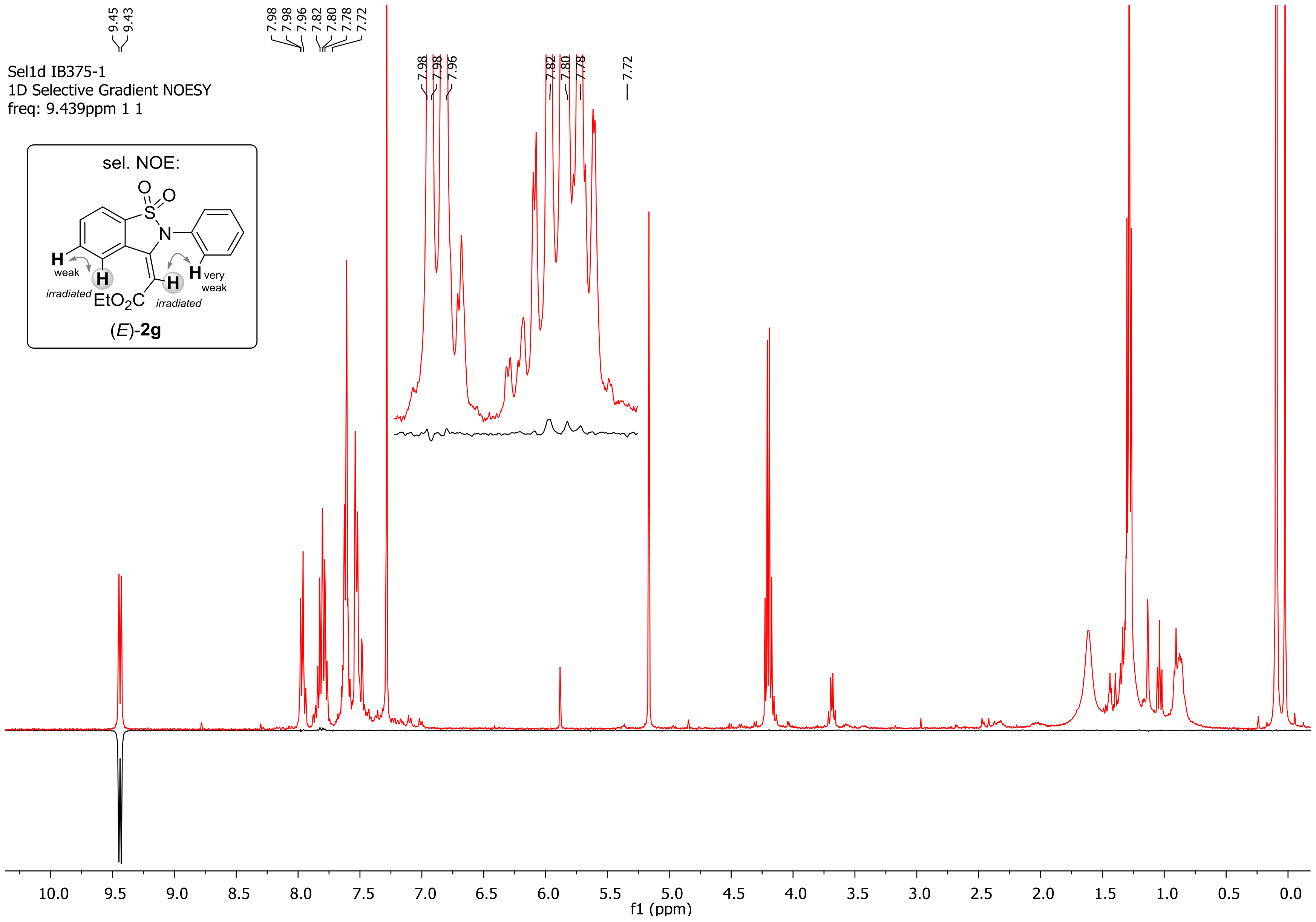
Sel1d IB375-1
1D Selective Gradient NOESY
freq: 9.439ppm 1 1
sel. NOE:
weak
irradiated
irradiated
very weak
(E)-2g
9.45
9.43
7.98
7.98
7.96
7.82
7.80
7.78
7.72
f1 (ppm)

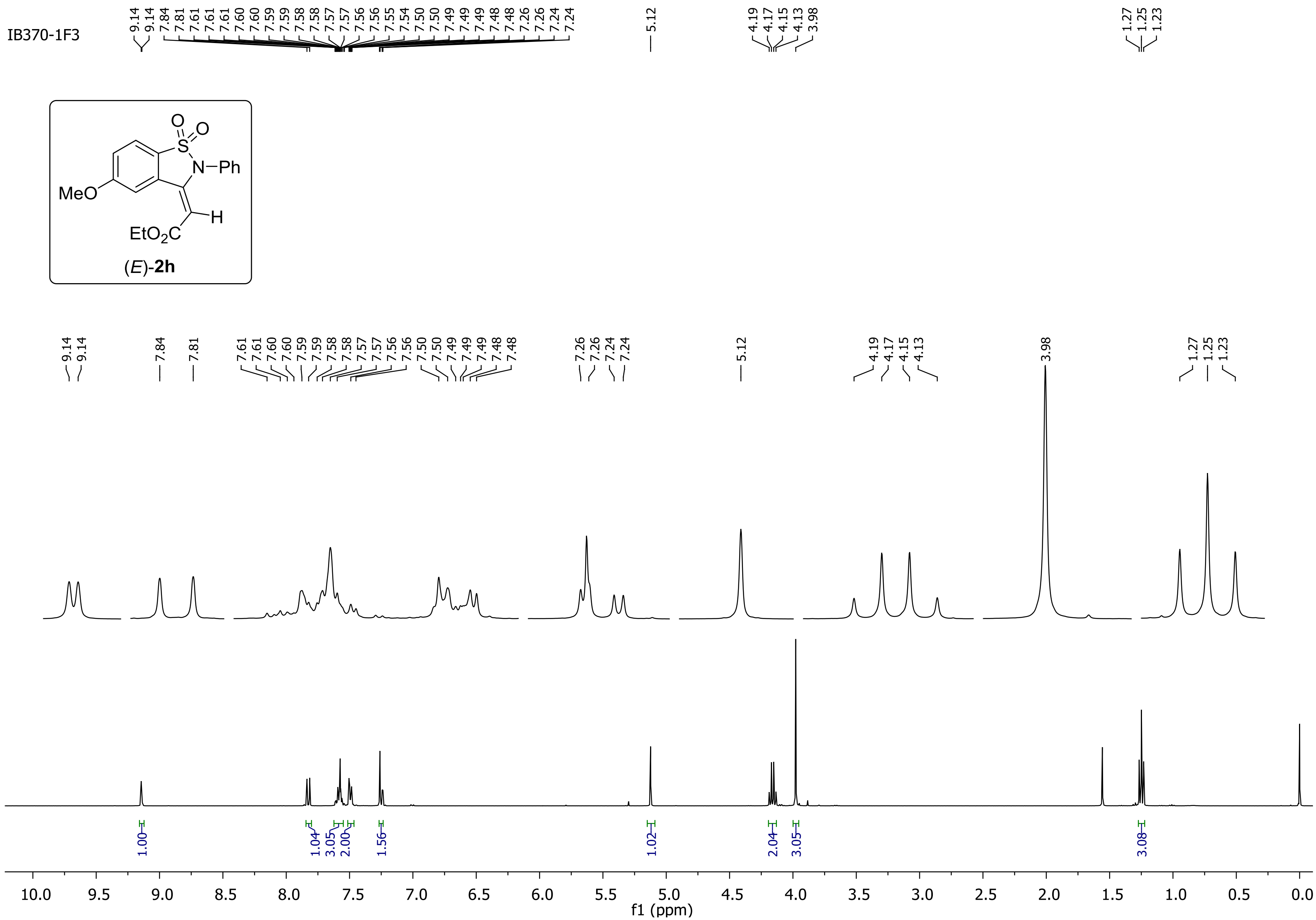
IB370-1F3
MeO
EtO2C
N−Ph
H
(E)-2h
f1 (ppm)

IB370-1F3

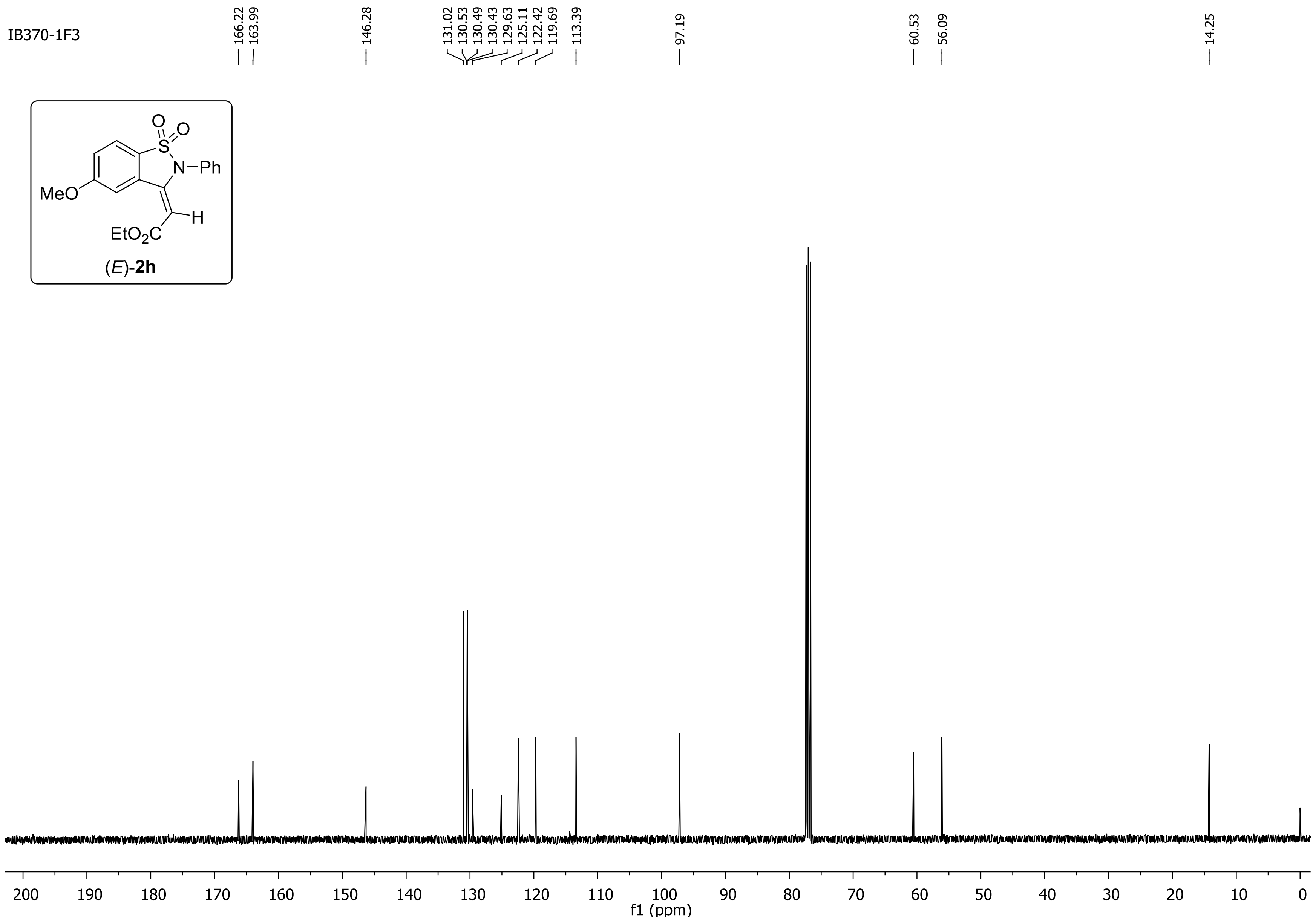

166.22
163.99
146.28
131.02
130.53
130.49
130.43
129.63
125.11
122.42
119.69
113.39
97.19
60.53
56.09
14.25
MeO
N−Ph
H
EtO2C
(E)-2h
200 190 180 170 160 150 140 130 120 110 100 90 80 70 60 50 40 30 20 10 0
f1 (ppm)

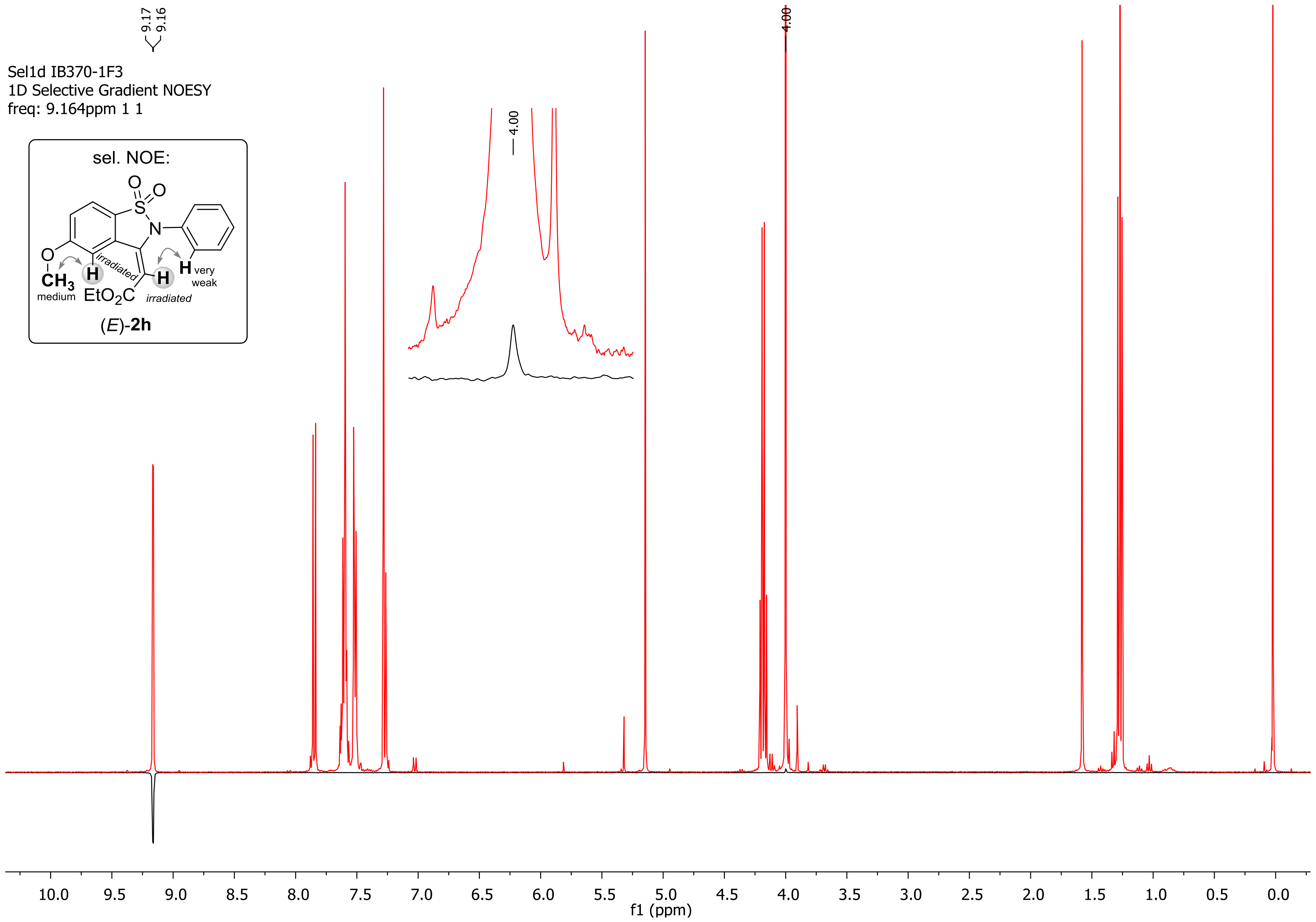

9.17
9.16
4.00
Sel1d IB370-1F3
1D Selective Gradient NOESY
freq: 9.164ppm 1 1
sel. NOE:
irradiated
irradiated
medium
very weak
EtO2C
(E)-2h
4.00
f1 (ppm)
10.0 9.5 9.0 8.5 8.0 7.5 7.0 6.5 6.0 5.5 5.0 4.5 4.0 3.5 3.0 2.5 2.0 1.5 1.0 0.5 0.0

IB379-1F2

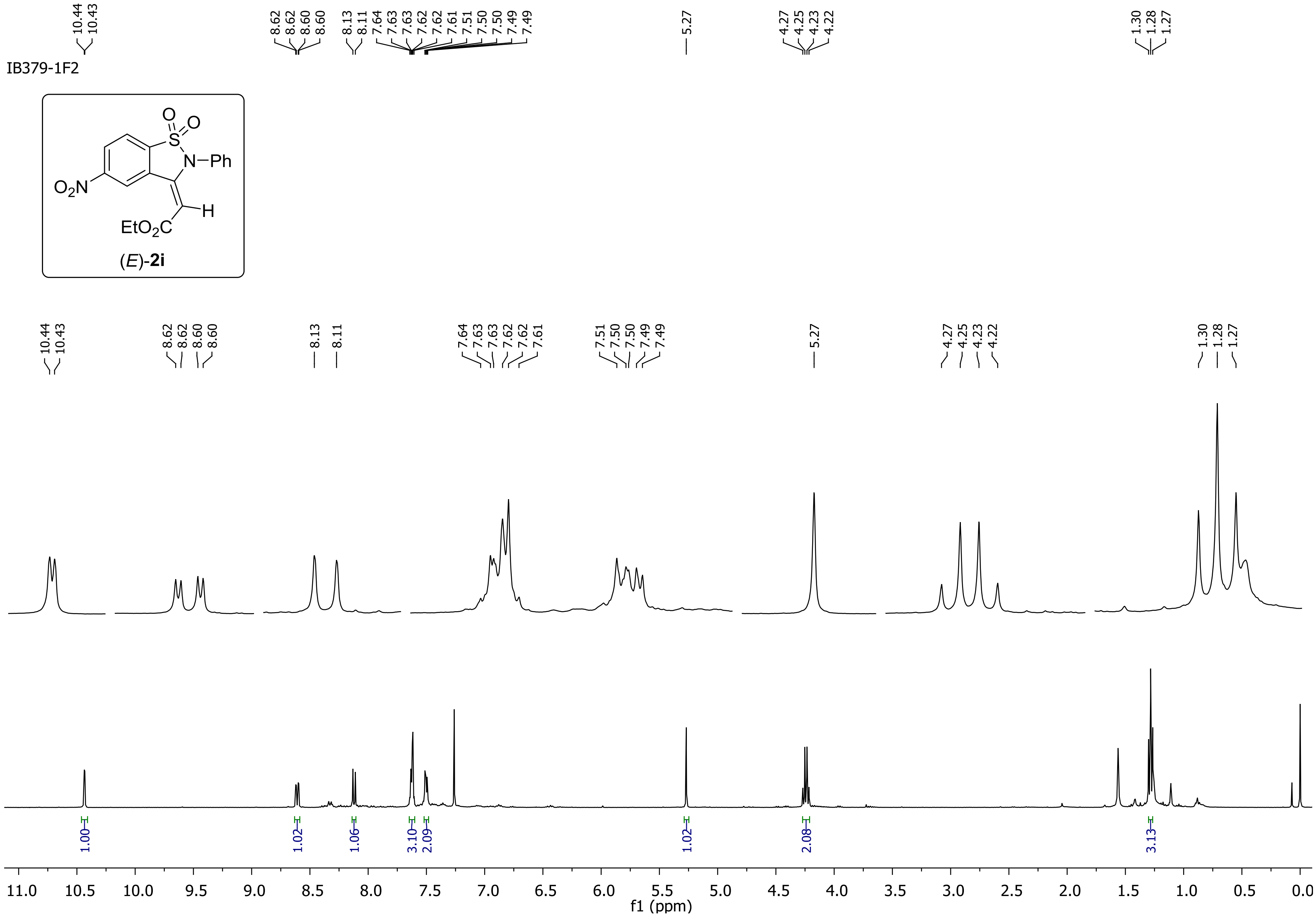

10.44
10.43
8.62
8.62
8.60
8.60
8.13
8.11
7.64
7.63
7.63
7.62
7.62
7.61
7.51
7.50
7.50
7.49
7.49
5.27
4.27
4.25
4.23
4.22
1.30
1.28
1.27
O
O
S
N−Ph
O2N
H
EtO2C
(E)-2i
1.00
1.02
1.06
3.10
2.09
1.02
2.08
3.13
11.0 10.5 10.0 9.5 9.0 8.5 8.0 7.5 7.0 6.5 6.0 5.5 5.0 4.5 4.0 3.5 3.0 2.5 2.0 1.5 1.0 0.5 0.0
f1 (ppm)

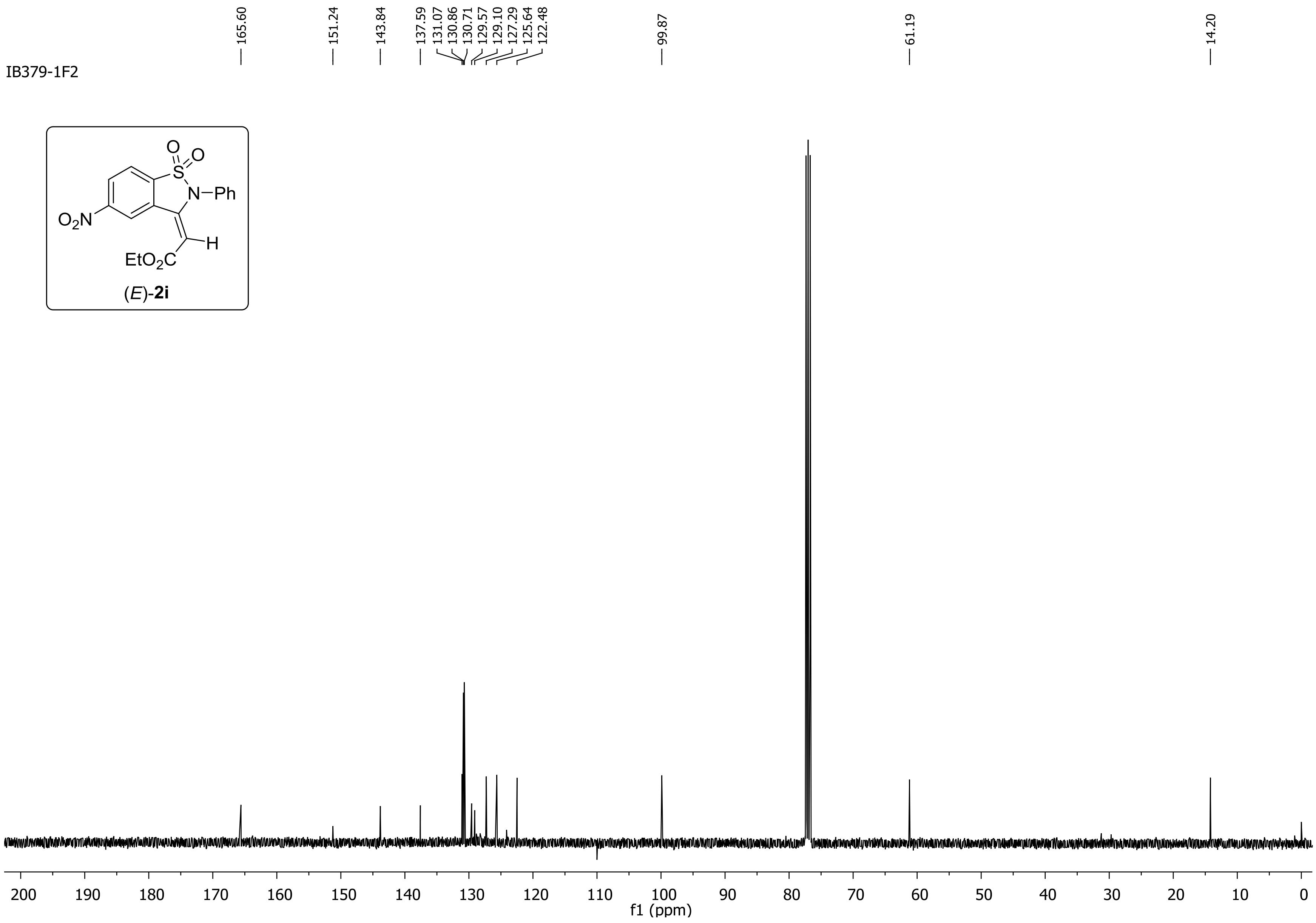
IB379-1F2
165.60
151.24
143.84
137.59
131.07
130.86
130.71
129.57
129.10
127.29
125.64
122.48
99.87
61.19
14.20
O₂N
N−Ph
EtO₂C
H
(E)-2i
f1 (ppm)

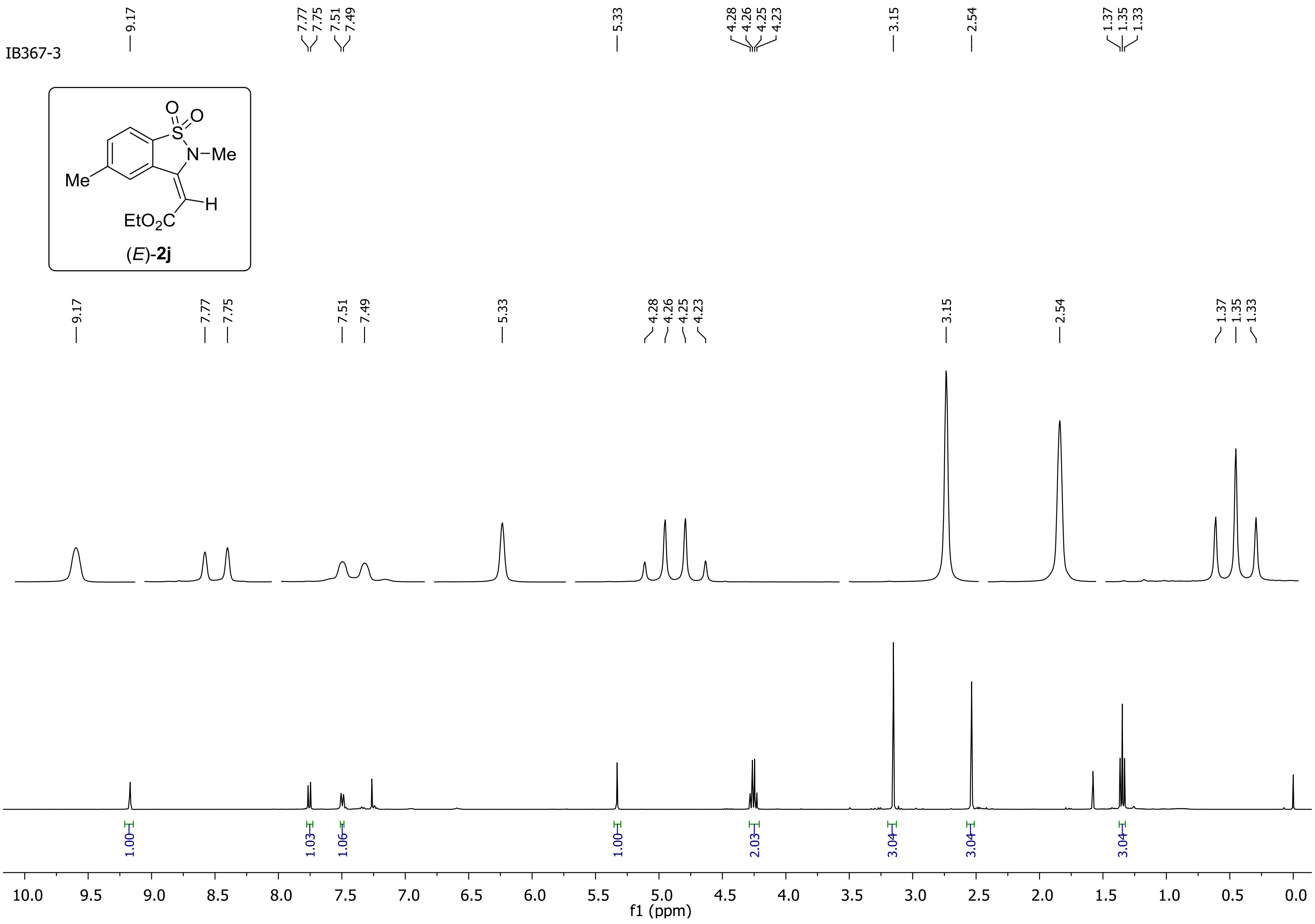
IB367-3
9.17
7.77 7.75 7.51 7.49
5.33
4.28 4.26 4.25 4.23
3.15
2.54
1.37 1.35 1.33
O O
S
N−Me
Me
H
EtO_2C
(*E*)-**2j**
1.00
1.03
1.06
1.00
2.03
3.04
3.04
3.04
10.0 9.5 9.0 8.5 8.0 7.5 7.0 6.5 6.0 5.5 5.0 4.5 4.0 3.5 3.0 2.5 2.0 1.5 1.0 0.5 0.0
f1 (ppm)

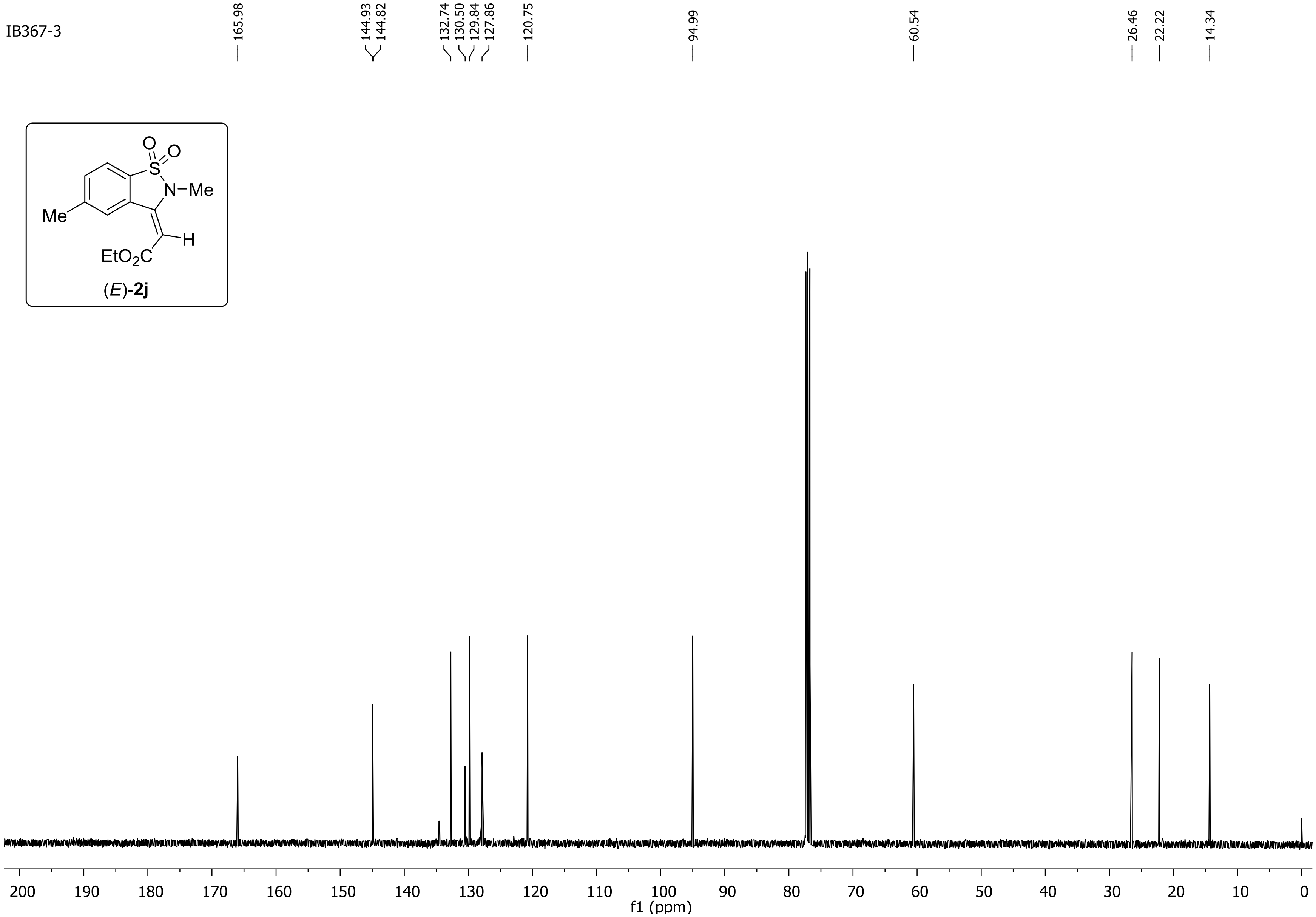
IB367-3
165.98
144.93
144.82
132.74
130.50
129.84
127.86
120.75
94.99
60.54
26.46
22.22
14.34
O
O
S
N−Me
Me
H
EtO_2C
(E)-2j
200 190 180 170 160 150 140 130 120 110 100 90 80 70 60 50 40 30 20 10 0
f1 (ppm)

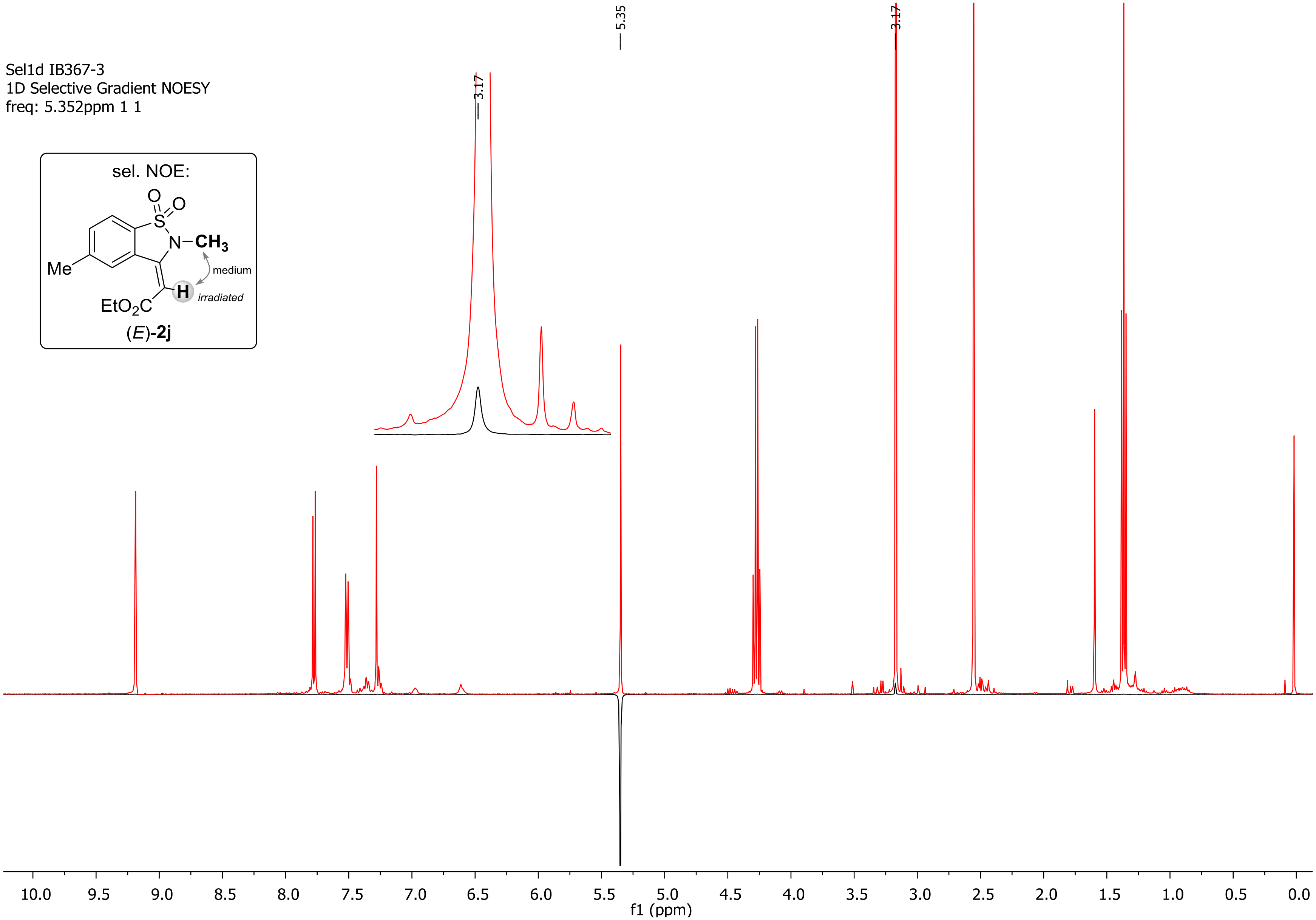

Sel1d IB367-3
1D Selective Gradient NOESY
freq: 5.352ppm 1 1
sel. NOE:
medium
irradiated
(E)-2j
5.35
3.17
3.17
f1 (ppm)
10.0
9.5
9.0
8.5
8.0
7.5
7.0
6.5
6.0
5.5
5.0
4.5
4.0
3.5
3.0
2.5
2.0
1.5
1.0
0.5
0.0

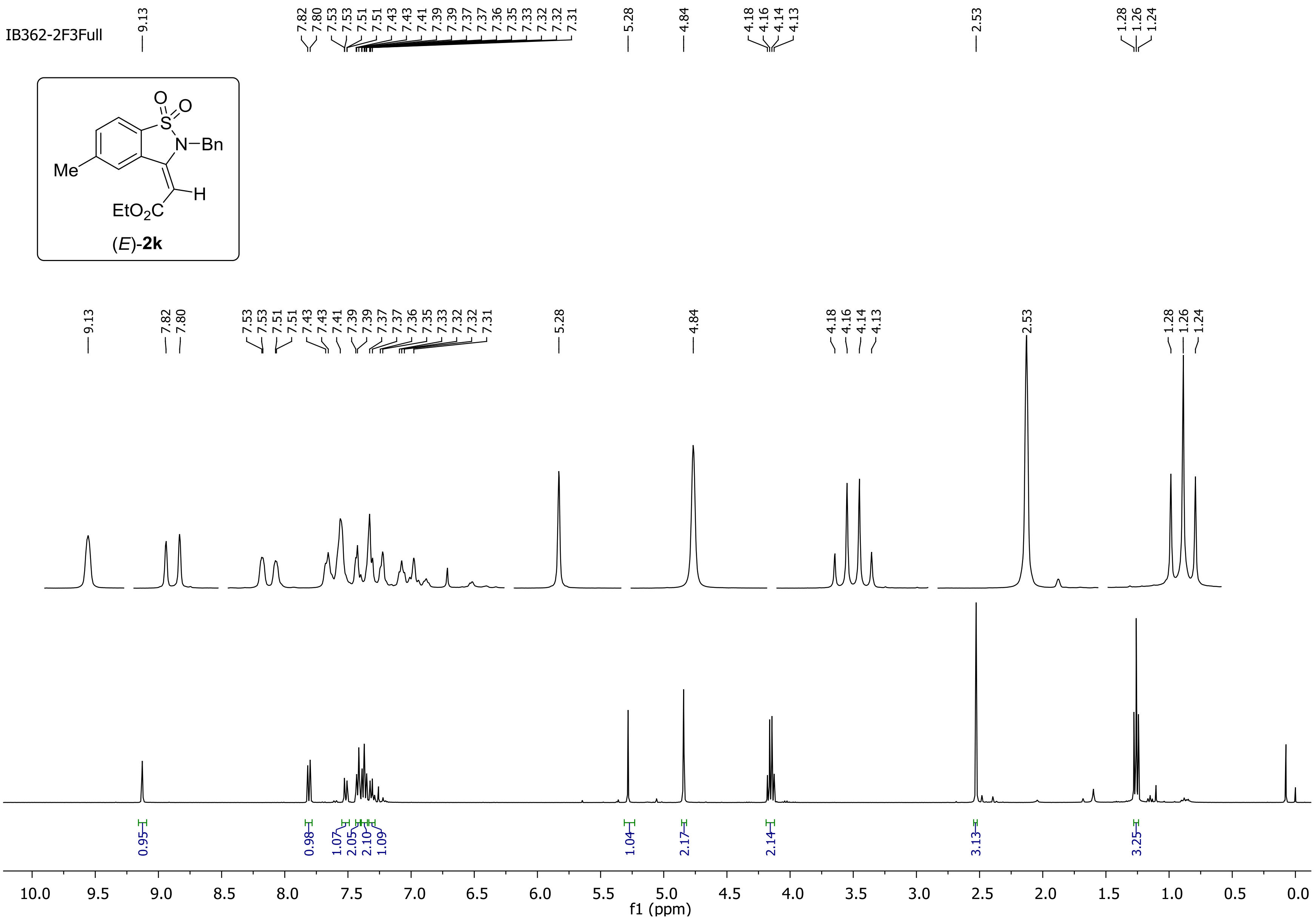
IB362-2F3Full
9.13
7.82 7.80 7.53 7.53 7.51 7.51 7.43 7.43 7.41 7.39 7.39 7.37 7.37 7.36 7.35 7.33 7.32 7.32 7.31
5.28
4.84
4.18 4.16 4.14 4.13
2.53
1.28 1.26 1.24
N−Bn
Me
H
EtO_2C
(E)-2k
0.95
0.98
1.07
2.05
2.10
1.09
1.04
2.17
2.14
3.13
3.25
10.0 9.5 9.0 8.5 8.0 7.5 7.0 6.5 6.0 5.5 5.0 4.5 4.0 3.5 3.0 2.5 2.0 1.5 1.0 0.5 0.0
f1 (ppm)

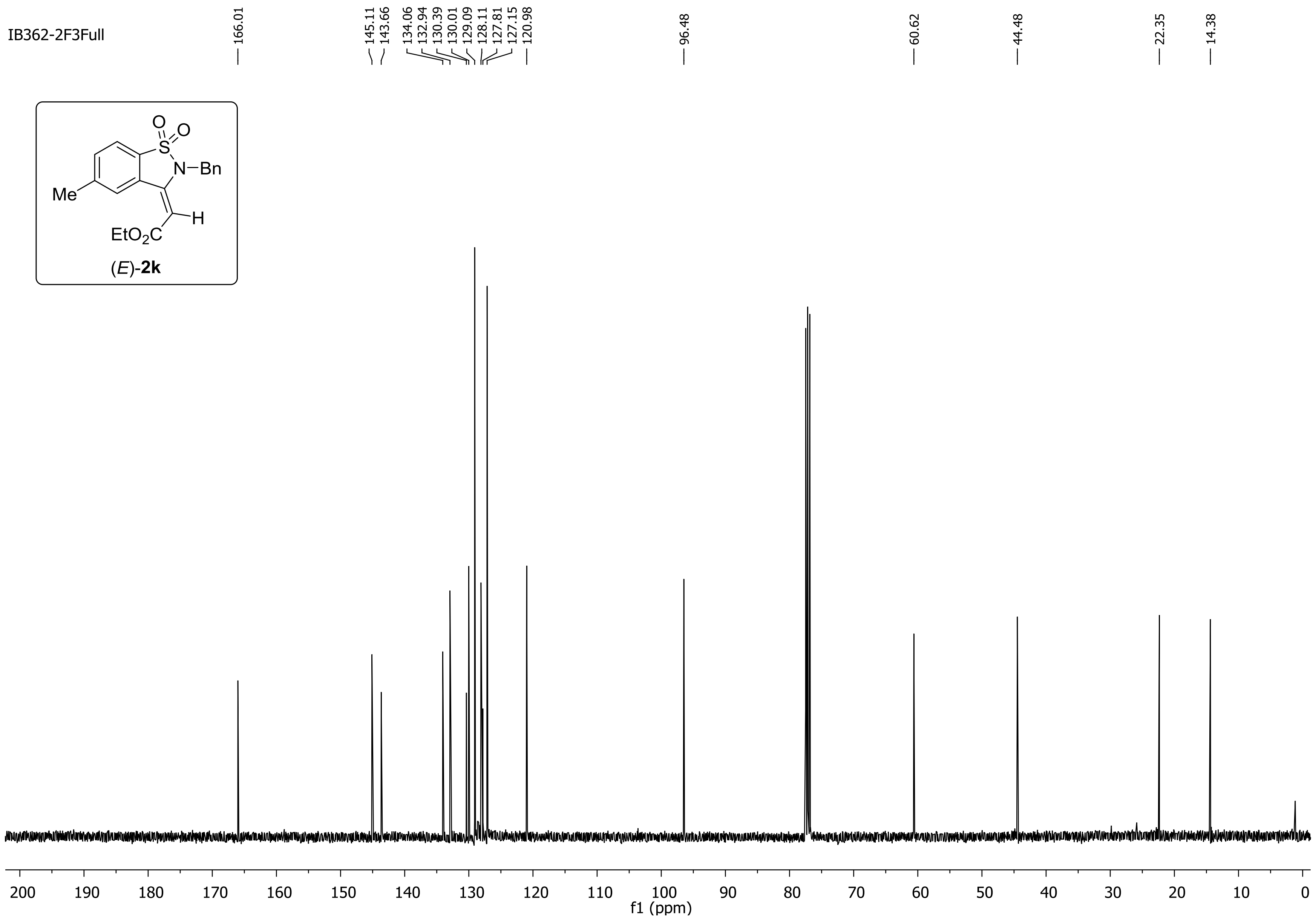
IB362-2F3Full
166.01
145.11
143.66
134.06
132.94
130.39
130.01
129.09
128.11
127.81
127.15
120.98
96.48
60.62
44.48
22.35
14.38
O
O
S
N−Bn
Me
H
EtO_2C
(E)-2k
200 190 180 170 160 150 140 130 120 110 100 90 80 70 60 50 40 30 20 10 0
f1 (ppm)

Sel1d IB362-2F3
1D Selective Gradient NOESY
freq: 5.310ppm 1 1

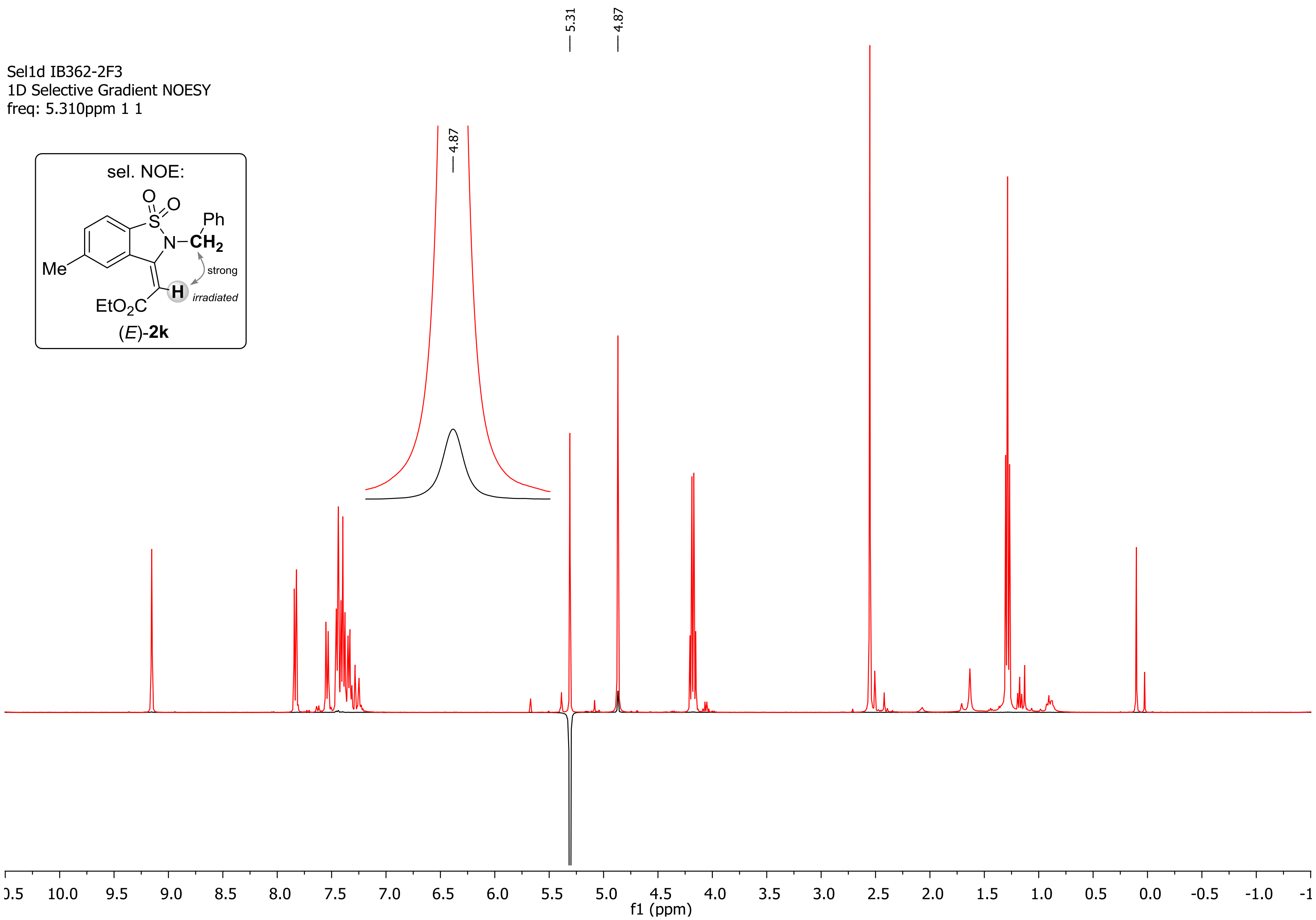

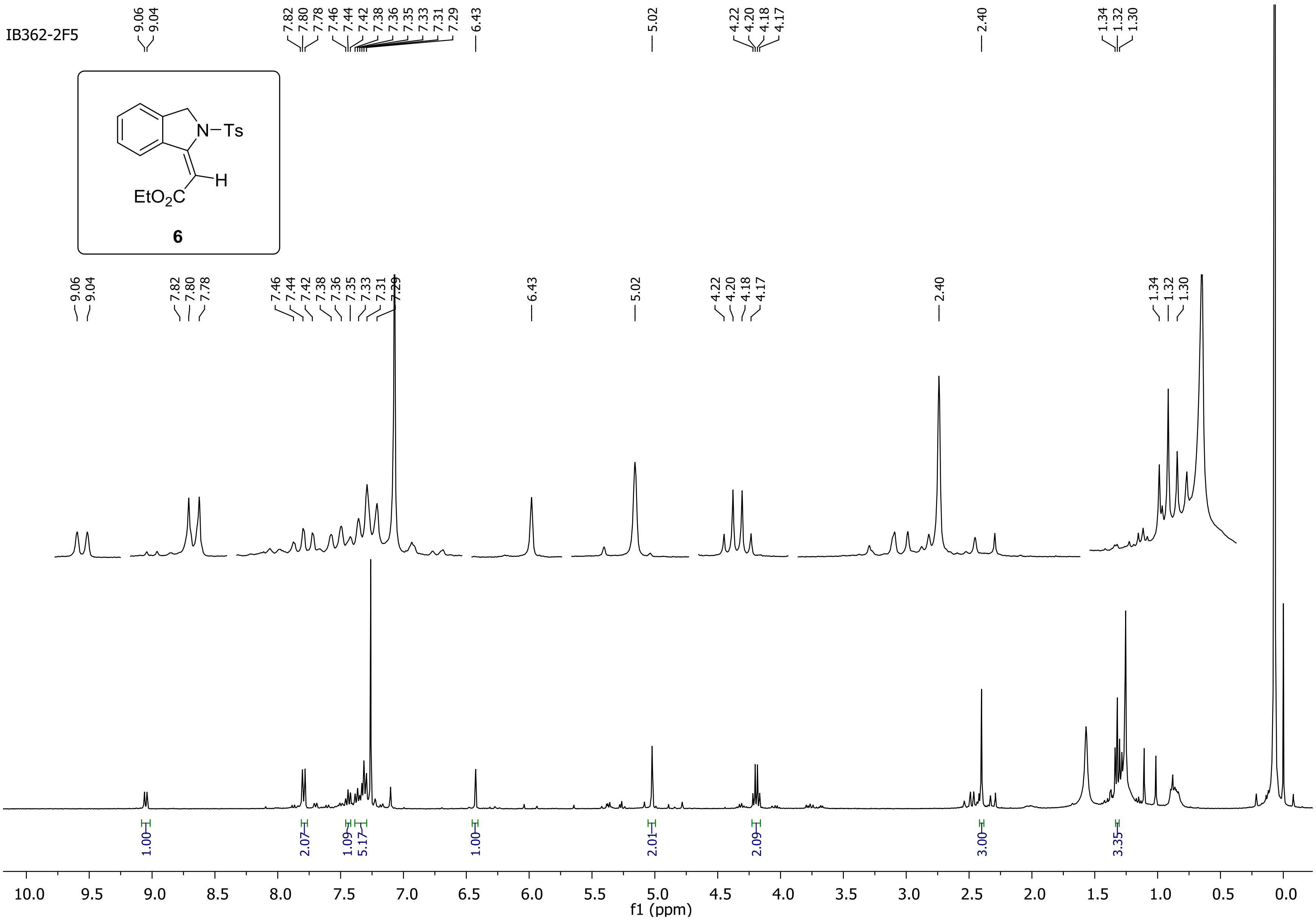
IB362-2F5
N−Ts
H
EtO2C
6
9.06
9.04
7.82
7.80
7.78
7.46
7.44
7.42
7.38
7.36
7.35
7.33
7.31
7.29
6.43
5.02
4.22
4.20
4.18
4.17
2.40
1.34
1.32
1.30
1.00
2.07
1.09
5.17
1.00
2.01
2.09
3.00
3.35
f1 (ppm)

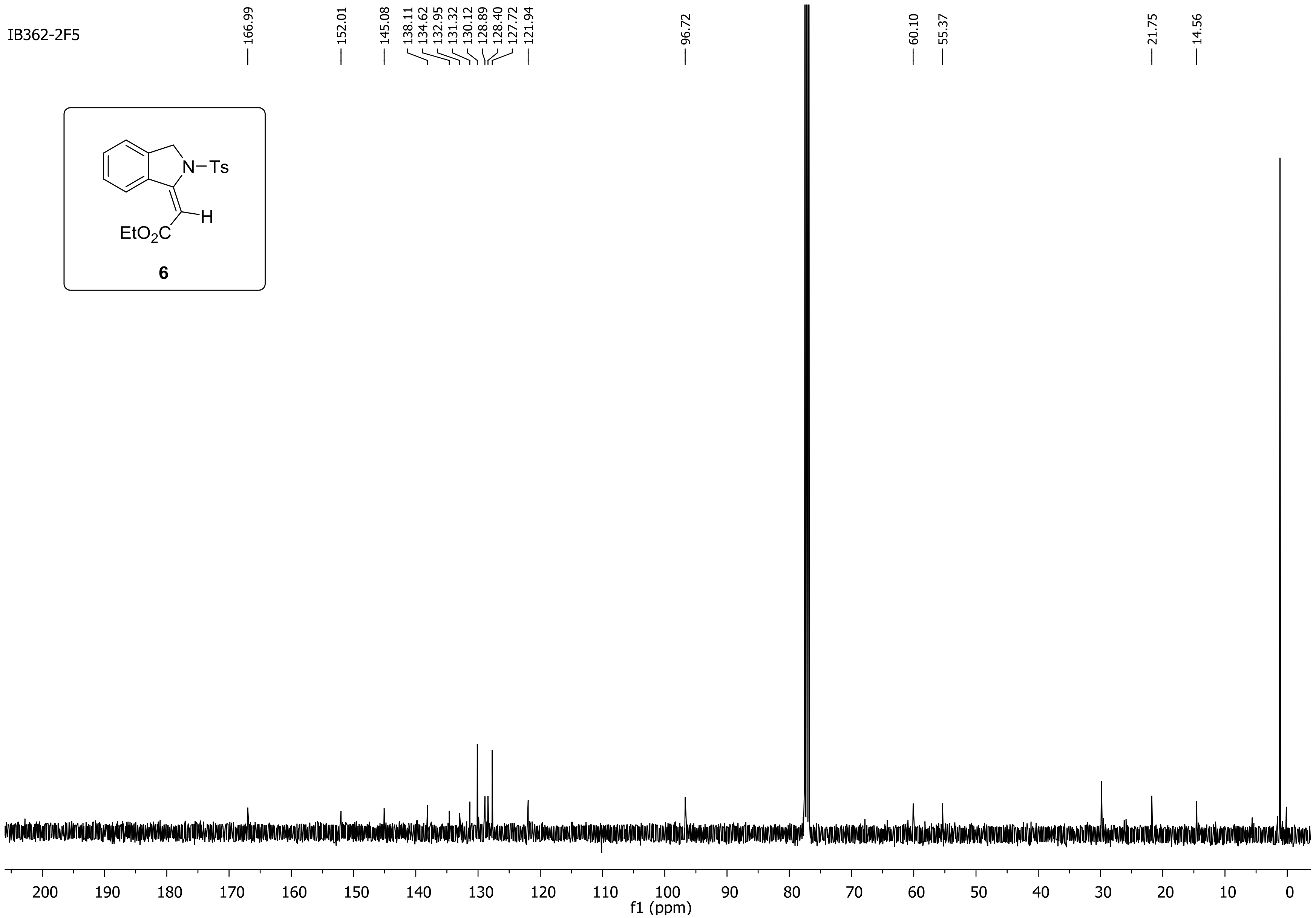
IB362-2F5
166.99
152.01
145.08
138.11
134.62
132.95
131.32
130.12
128.89
128.40
127.72
121.94
96.72
60.10
55.37
21.75
14.56
N−Ts
H
EtO2C
6
200
190
180
170
160
150
140
130
120
110
100
90
80
70
60
50
40
30
20
10
0
f1 (ppm)

Photochemical Rearrangement of 1,2-Benzothiazole-1,1-diones. Products 12a–d

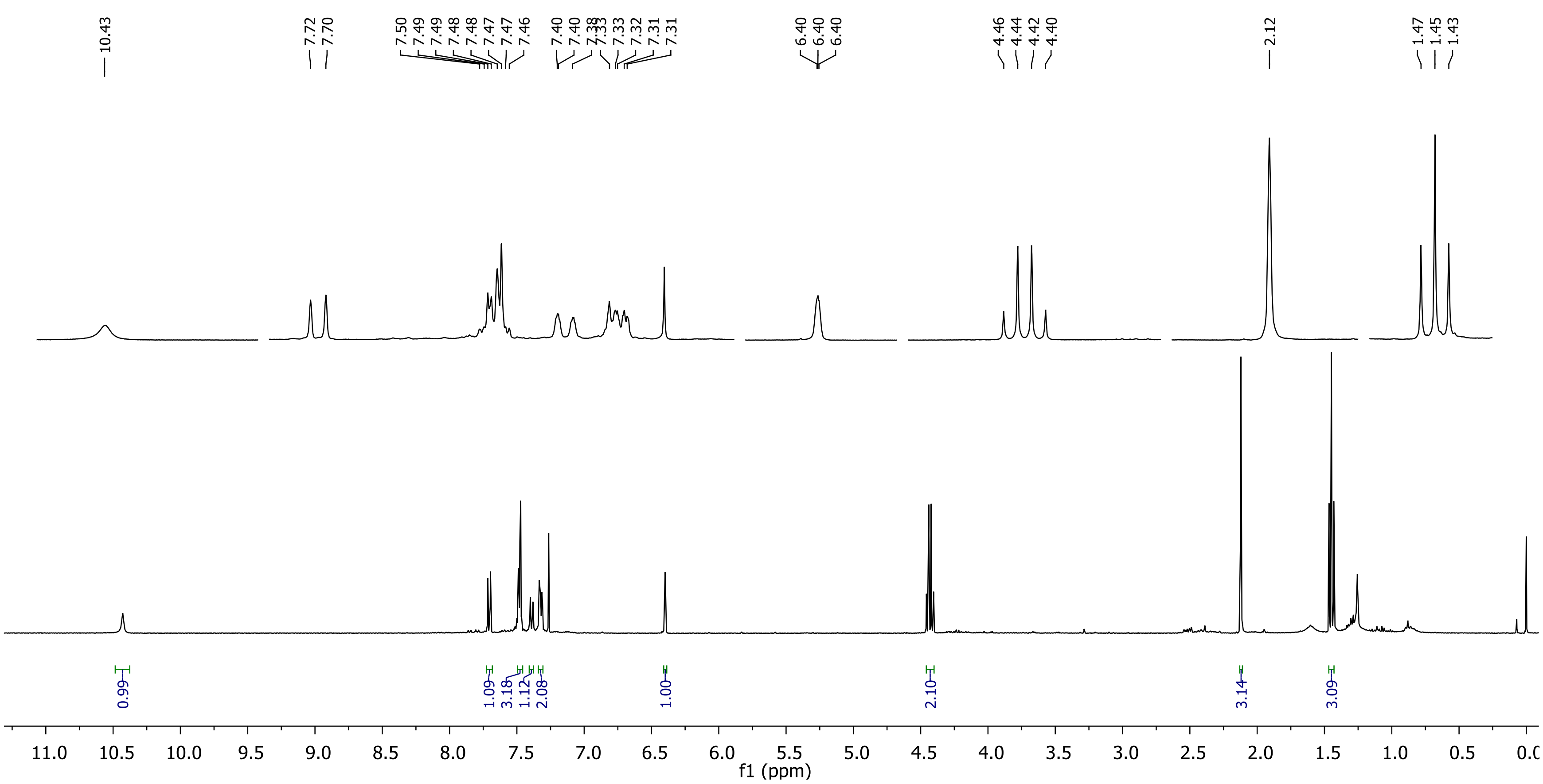

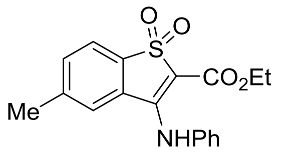

12a

IB337-2F2Full

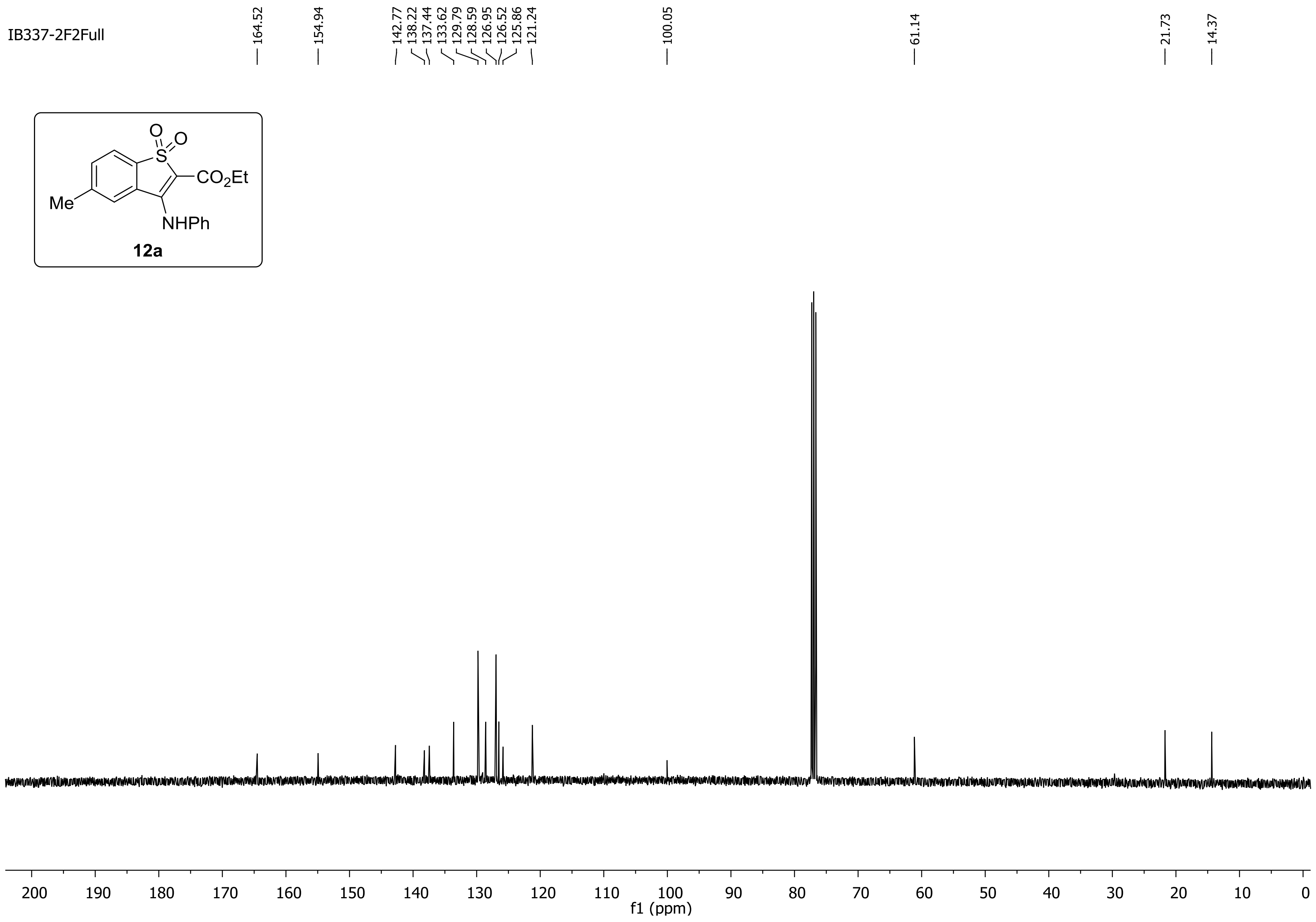

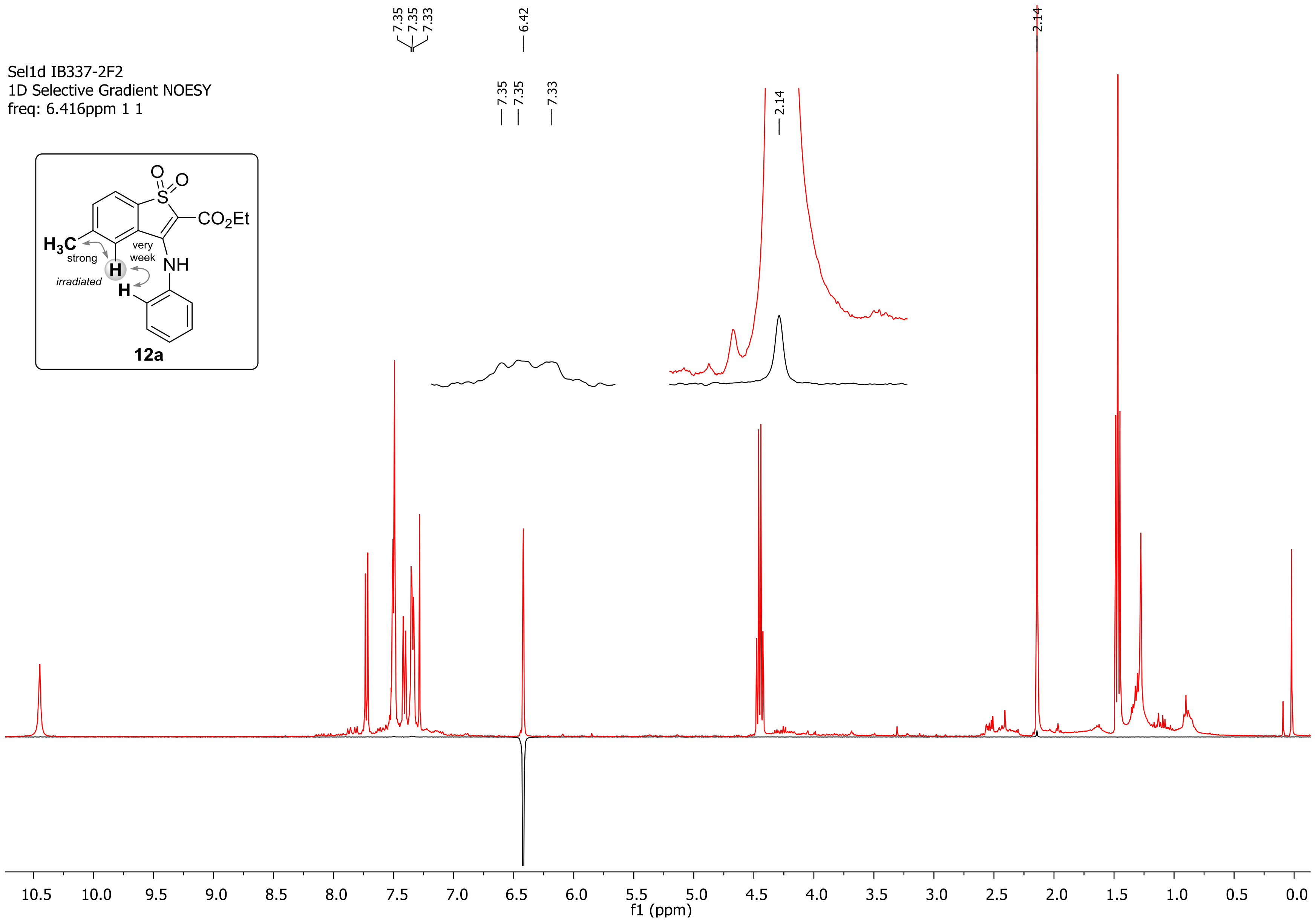

Sel1d IB337-2F2
1D Selective Gradient NOESY
freq: 6.416ppm 1 1
7.35
7.35
7.33
6.42
2.14
H3C
strong
H
irradiated
very
week
NH
H
CO2Et
12a
f1 (ppm)

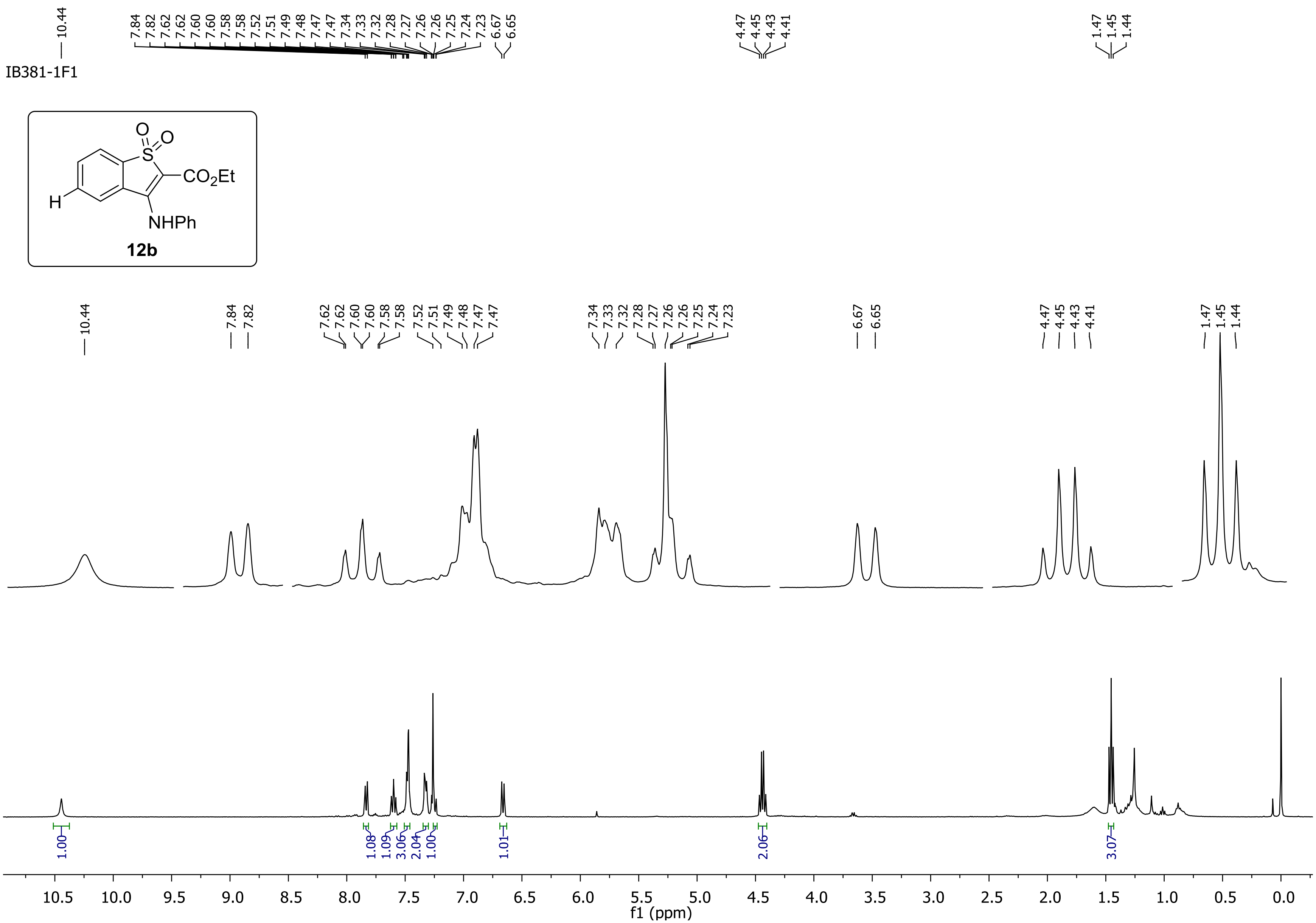
IB381-1F1
10.44
7.84 7.82 7.62 7.62 7.60 7.60 7.58 7.58 7.52 7.51 7.49 7.48 7.47 7.47 7.34 7.33 7.32 7.28 7.27 7.26 7.26 7.25 7.24 7.23 6.67 6.65
4.47 4.45 4.43 4.41
1.47 1.45 1.44
CO2Et
H
NHPh
12b
1.00
1.08
1.09
3.06
2.04
1.00
1.01
2.06
3.07
10.5 10.0 9.5 9.0 8.5 8.0 7.5 7.0 6.5 6.0 5.5 5.0 4.5 4.0 3.5 3.0 2.5 2.0 1.5 1.0 0.5 0.0
f1 (ppm)

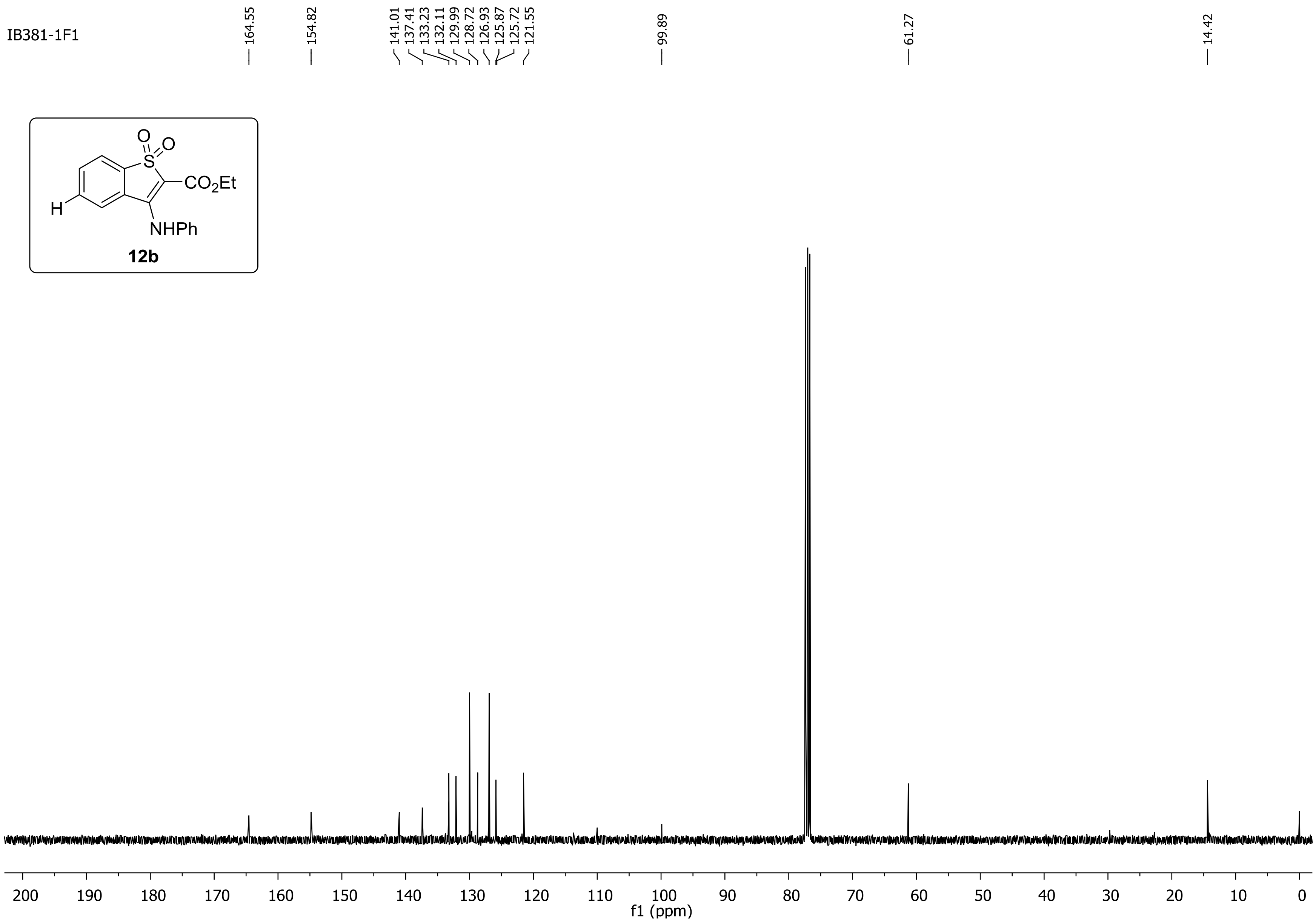
IB381-1F1
164.55
154.82
141.01
137.41
133.23
132.11
129.99
128.72
126.93
125.87
125.72
121.55
99.89
61.27
14.42
O
O
S
CO2Et
H
NHPh
12b
200 190 180 170 160 150 140 130 120 110 100 90 80 70 60 50 40 30 20 10 0
f1 (ppm)

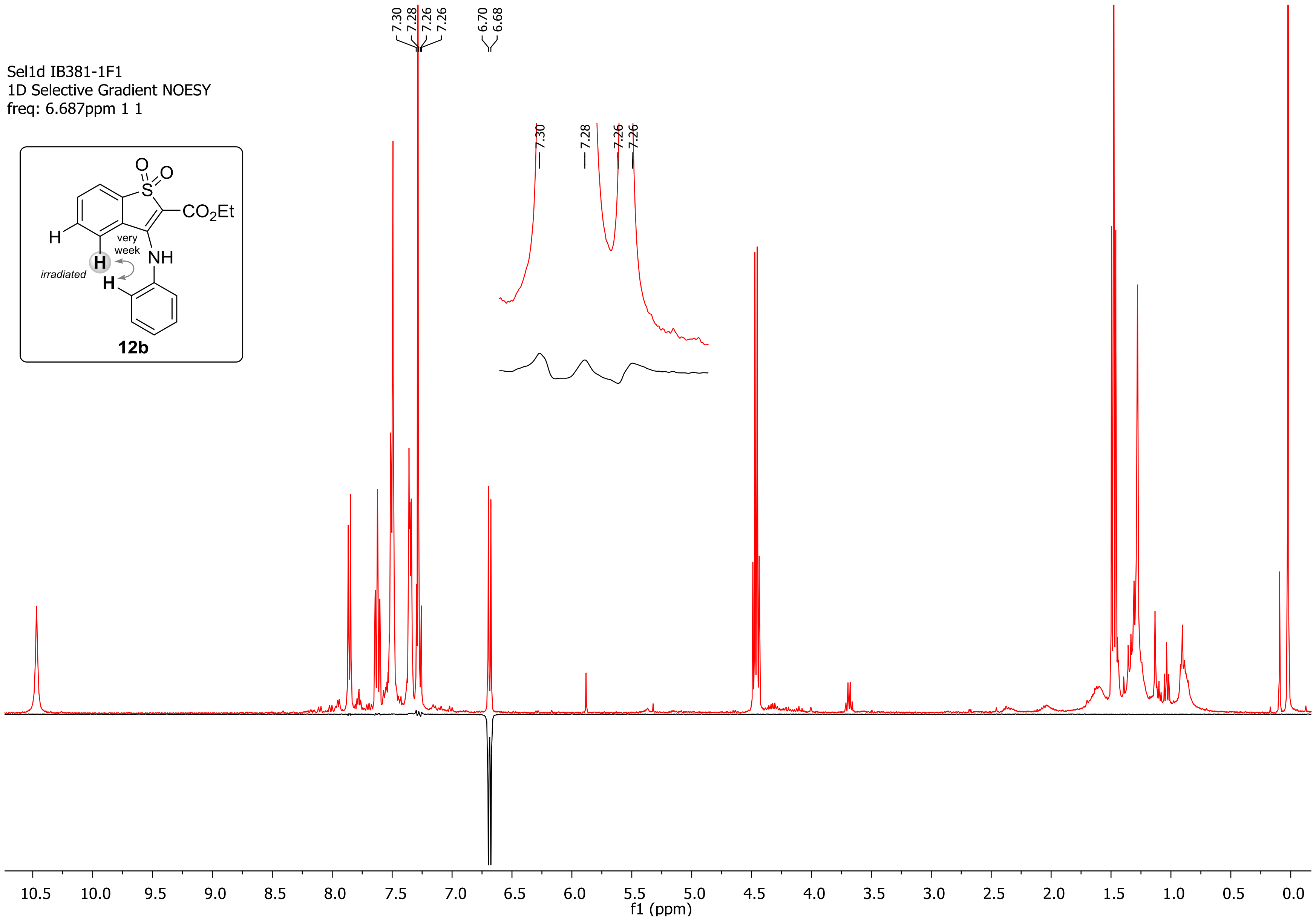
Sel1d IB381-1F1
1D Selective Gradient NOESY
freq: 6.687ppm 1 1
very
week
irradiated
NH
CO_2Et
12b
f1 (ppm)

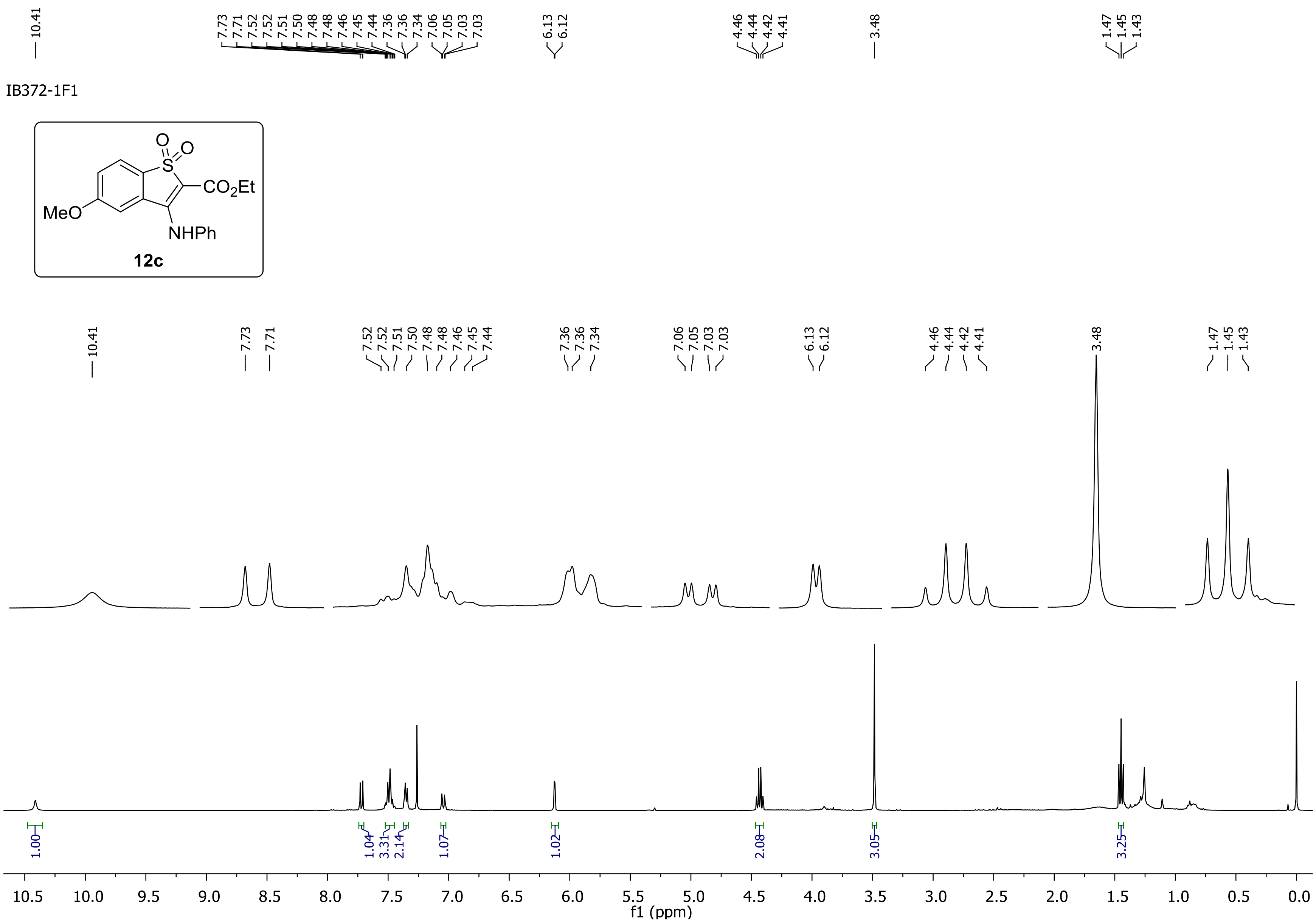

IB372-1F1
MeO
CO2Et
NHPh
12c
f1 (ppm)

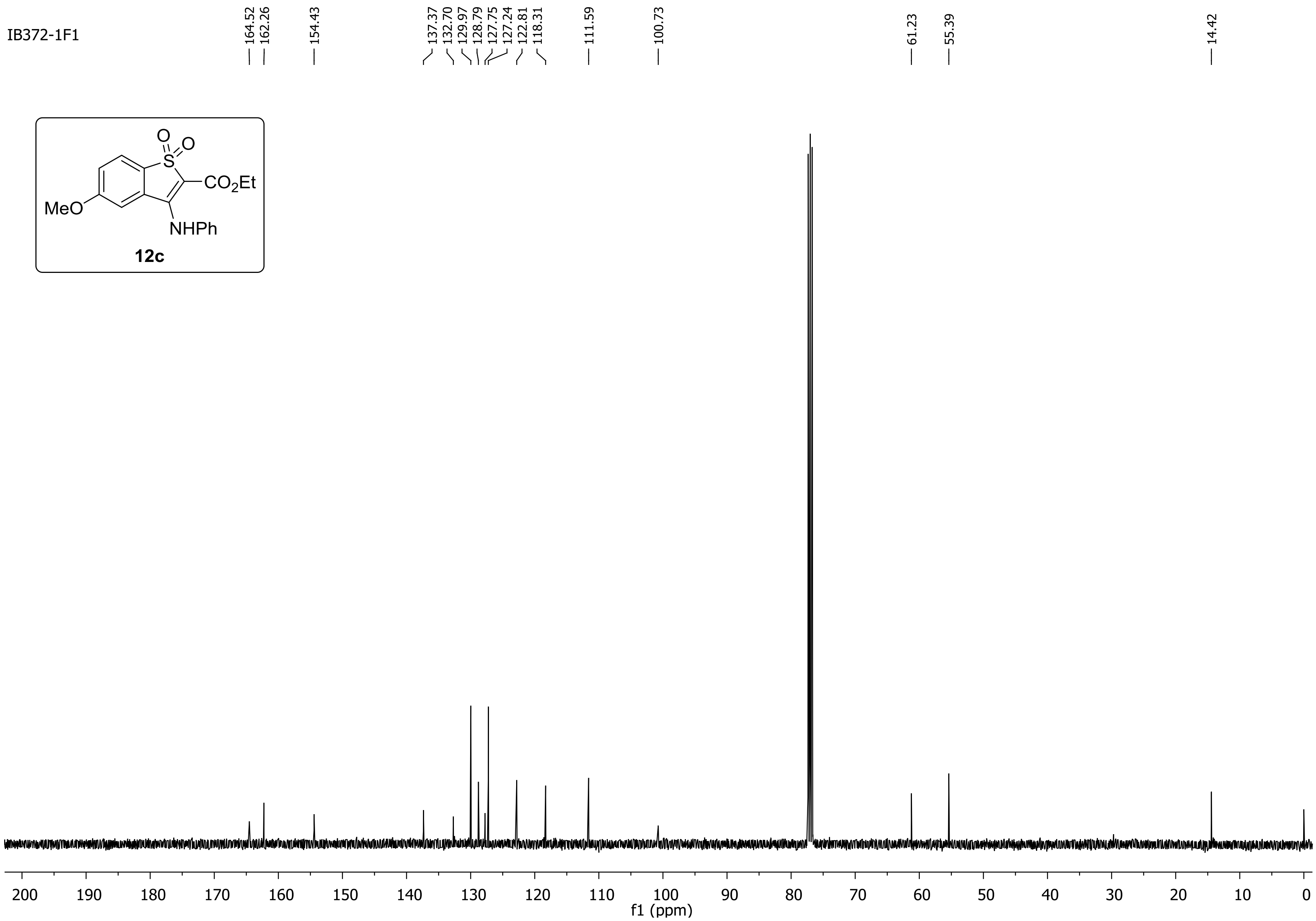
IB372-1F1
164.52
162.26
154.43
137.37
132.70
129.97
128.79
127.75
127.24
122.81
118.31
111.59
100.73
61.23
55.39
14.42
MeO
CO_2Et
NHPh
12c
200 190 180 170 160 150 140 130 120 110 100 90 80 70 60 50 40 30 20 10 0
f1 (ppm)

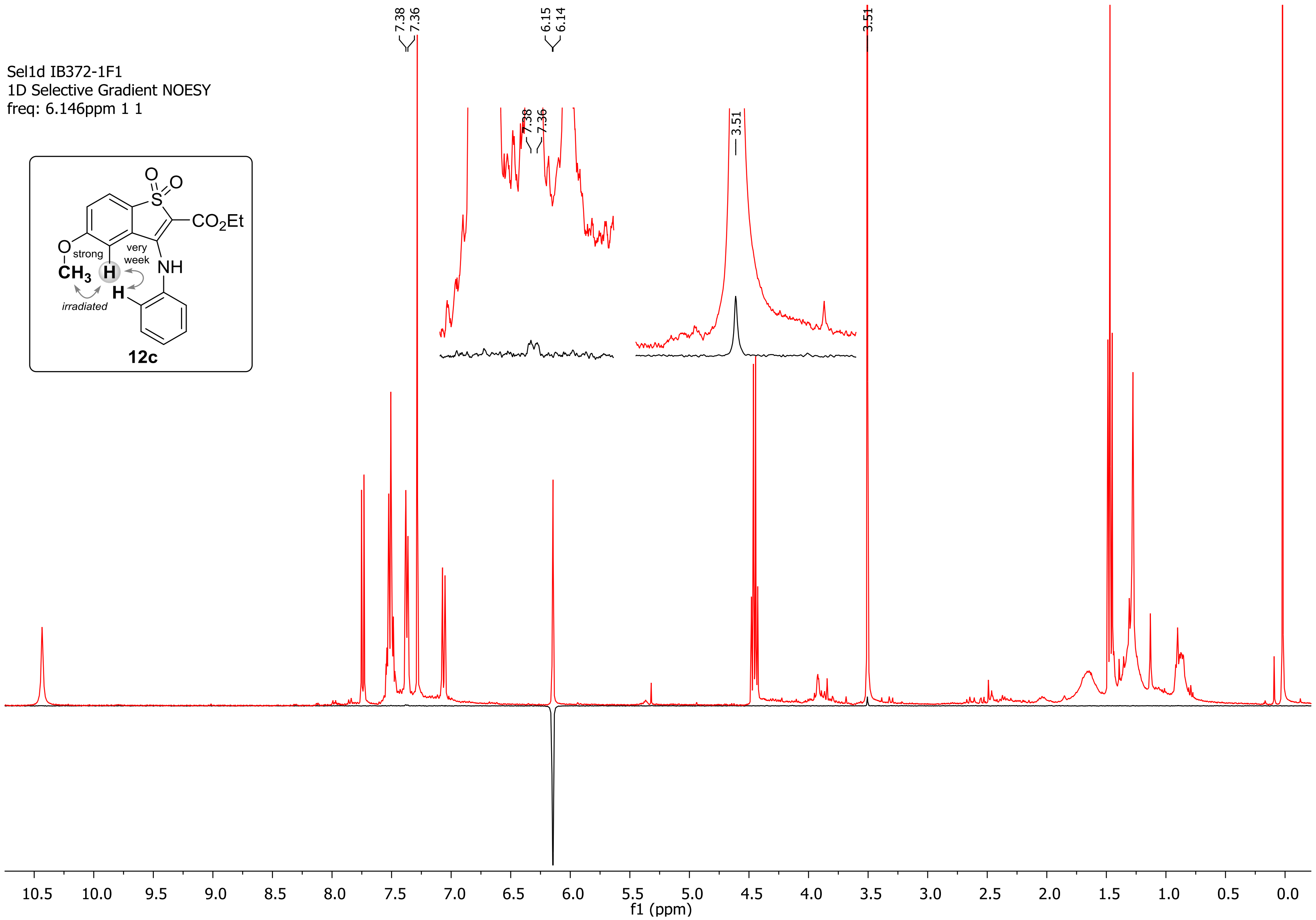
Sel1d IB372-1F1
1D Selective Gradient NOESY
freq: 6.146ppm 1 1
strong
very week
irradiated
12c
7.38
7.36
6.15
6.14
3.51
f1 (ppm)

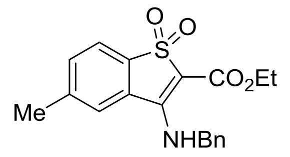

12d

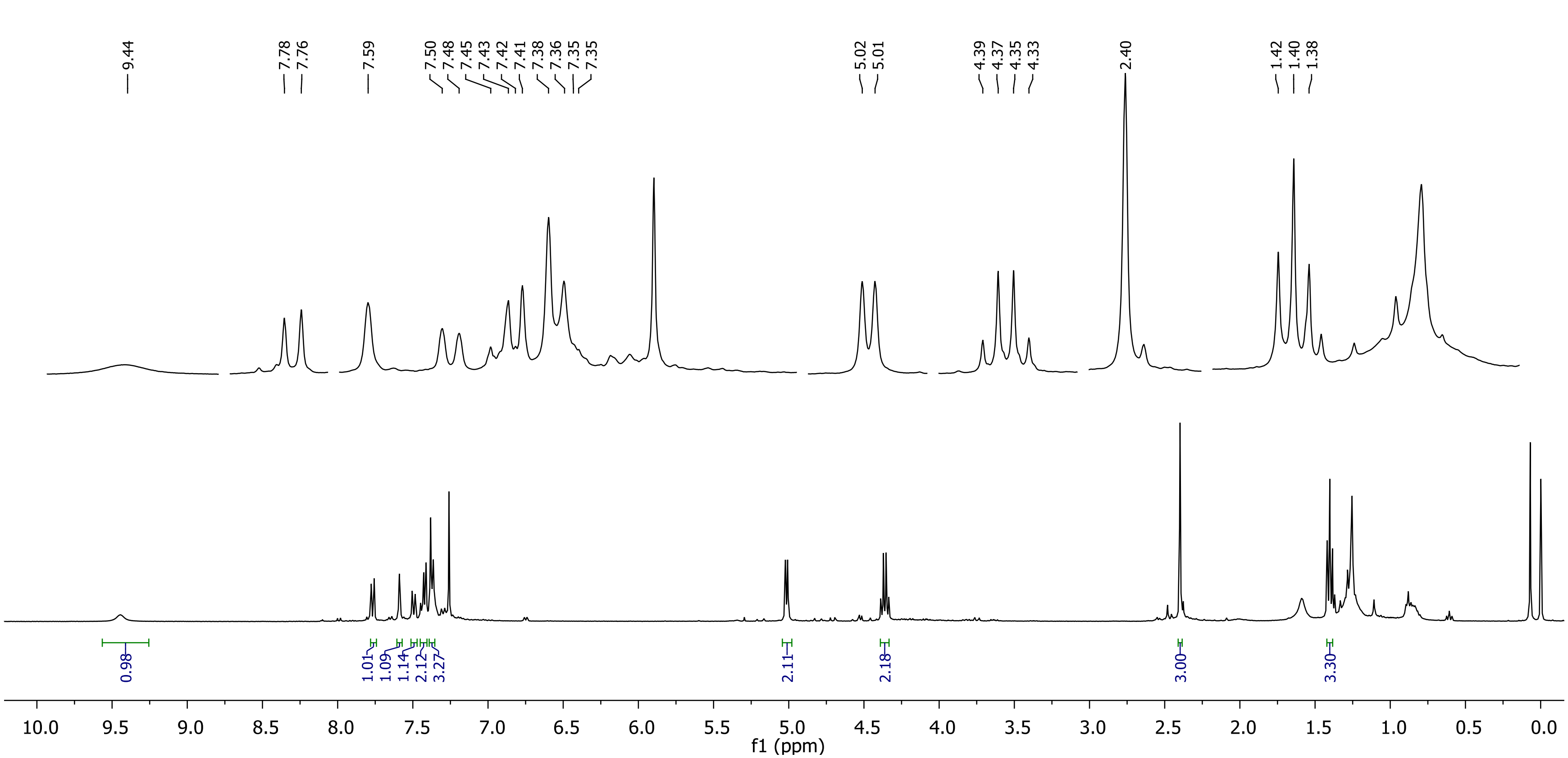
IB364-4F1
9.44
7.78 7.76 7.59 7.50 7.48 7.45 7.43 7.42 7.41 7.38 7.36 7.35 7.35
5.02 5.01
4.39 4.37 4.35 4.33
2.40
1.42 1.40 1.38
0.98
1.01 1.09 1.14 2.12 3.27
2.11
2.18
3.00
3.30
10.0 9.5 9.0 8.5 8.0 7.5 7.0 6.5 6.0 5.5 5.0 4.5 4.0 3.5 3.0 2.5 2.0 1.5 1.0 0.5 0.0
f1 (ppm)

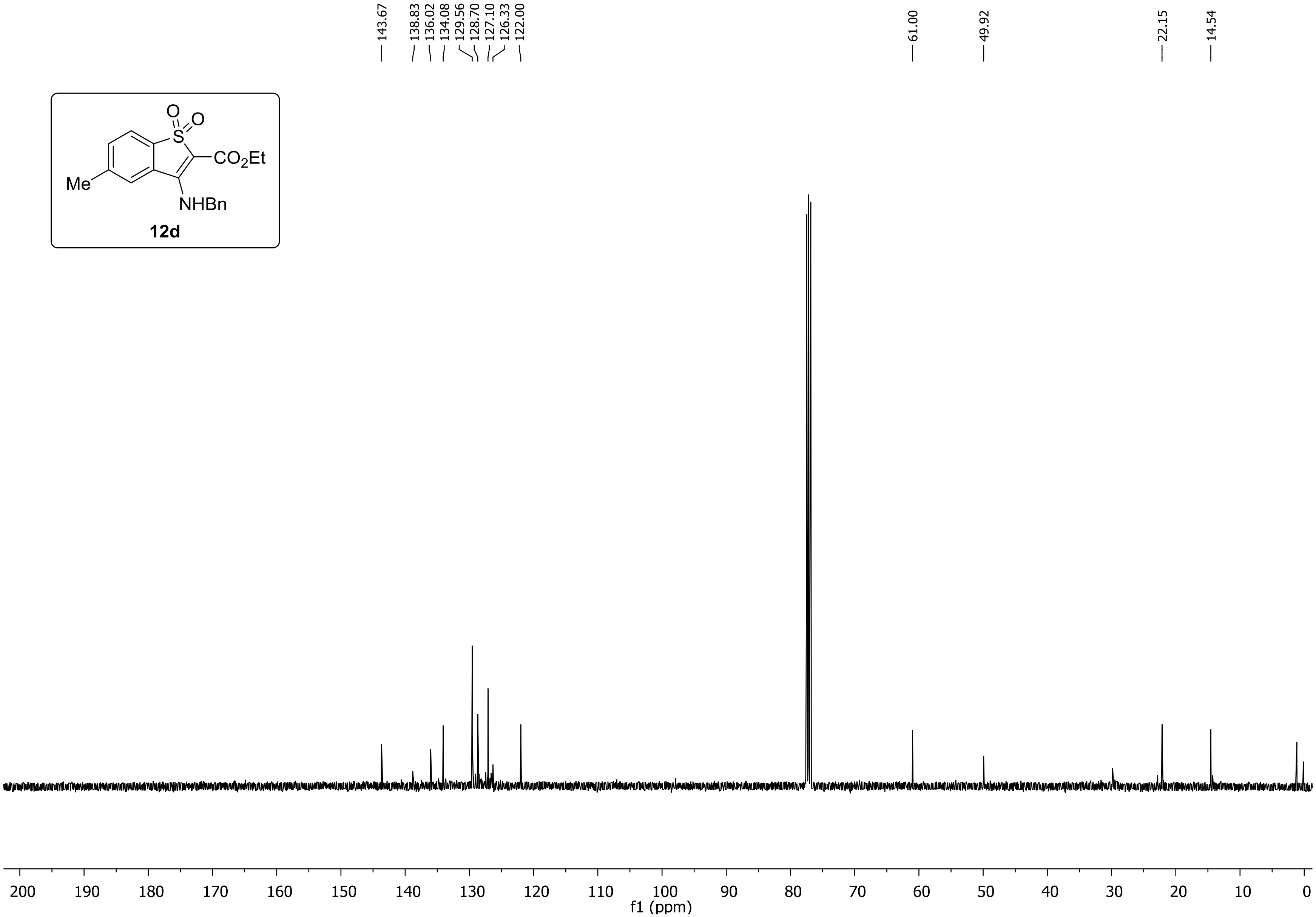

O
O
S
CO2Et
Me
NHBn
12d
143.67
138.83
136.02
134.08
129.56
128.70
127.10
126.33
122.00
61.00
49.92
22.15
14.54
200
190
180
170
160
150
140
130
120
110
100
90
80
70
60
50
40
30
20
10
0
f1 (ppm)

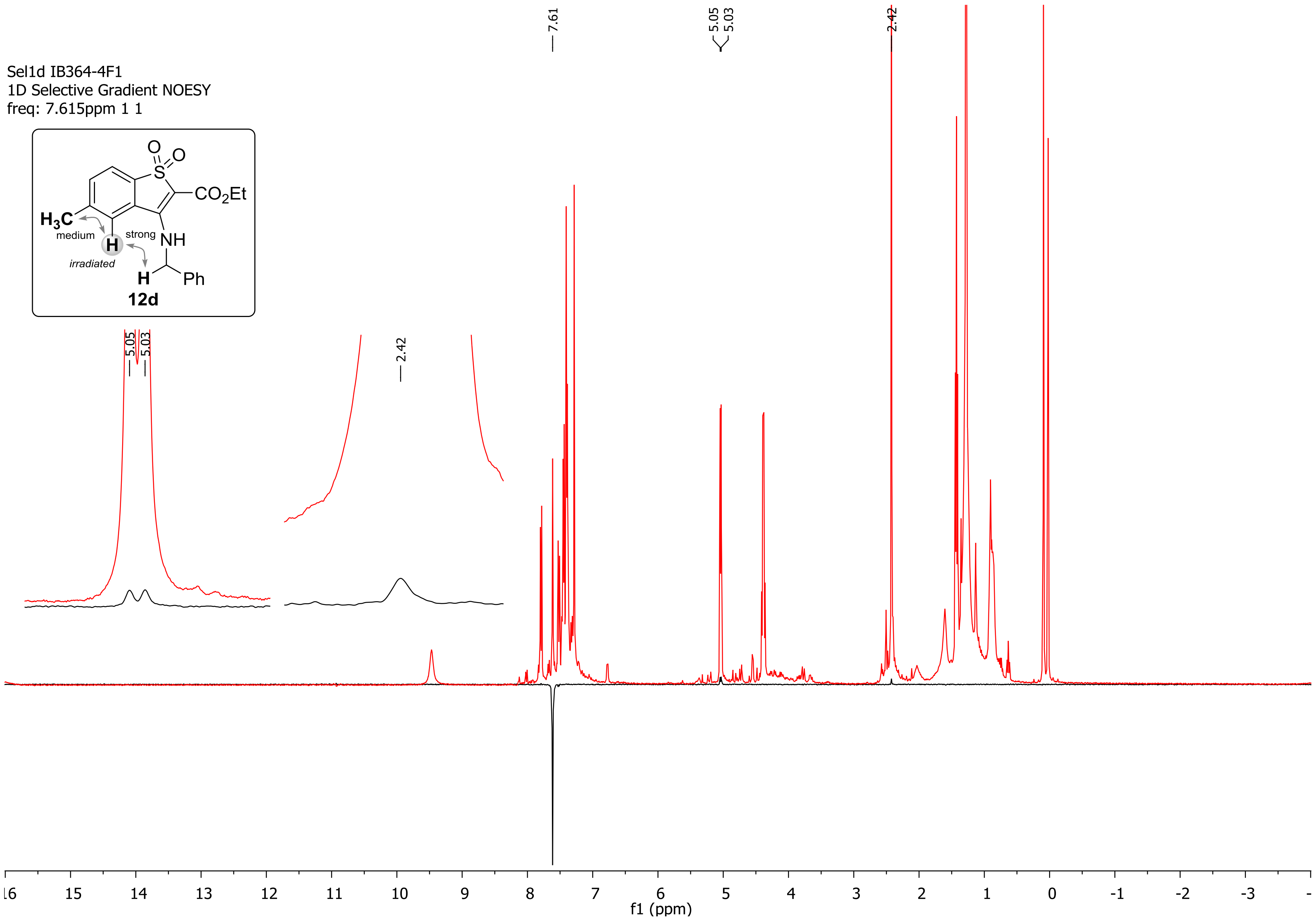
Sel1d IB364-4F1
1D Selective Gradient NOESY
freq: 7.615ppm 1 1
medium
strong
irradiated
12d
7.61
5.05
5.03
2.42
f1 (ppm)

Computational Methods

All structures were initially optimized using density functional theory (DFT) by using the B3LYP[1] functional as implemented in Gaussian 09.[2] Optimizations were carried out in a solvent model (IEFPCM, solvent = toluene)[3] by using the 6-31G** basis set for non-metallic atoms and Stuttgart/Dresden (SDD)[4] effective core potential for palladium. The critical stationary points were characterized by frequency calculations in order to verify that they have the right number of imaginary frequencies, and the intrinsic reaction coordinates (IRC)[5] were followed to verify the energy profiles connecting the key transition structures to the correct associated local minima.

The energies showed in the manuscript have been refined by single-point calculations with the M06[6] functional and def2tzvpp basis set on the previously optimized structures. The values correspond to Free Gibbs energies and are given in kcal/mol. These energies are relative to the initial mixtures of starting material and corresponding palladium complexes, marked as G = 0.0 kcal/mol in each Figure.

---

[1] (a) Lee, C.; Yang, W.; Parr, R. G. *Phys. Rev. B* **1988**, *37*, 785–789; (b) Becke, A. D. *J. Chem. Phys.* **1993**, *98*, 5648–5652; (c) Kohn, W.; Becke, A. D.; Parr, R. G. *J. Phys. Chem*. **1996**, *100*, 12974–12980.

[2] Gaussian 09, Revision D.01; Frisch, M. J.; Trucks, G. W.; Schlegel, H. B.; Scuseria, G. E.; Robb, M. A.; Cheeseman, J. R.; Scalmani, G.; Barone, V.; Mennucci, B.; Petersson, G. A.; Nakatsuji, H.; Caricato, M.; Li, X.; Hratchian, H. P.; Izmaylov, A. F.; Bloino, J.; Zheng, G.; Sonnenberg, J. L.; Hada, M.; Ehara, M.; Toyota, K.; Fukuda, R.; Hasegawa, J.; Ishida, M.; Nakajima, T.; Honda, Y.; Kitao, O.; Nakai, H.; Vreven, T.; Montgomery, J. A.; Peralta, J. E.; Ogliaro, F.; Bearpark, M.; Heyd, J. J.; Brothers, E.; Kudin, K. N.; Staroverov, V. N.; Keith, T.; Kobayashi, R.; Normand, J.; Raghavachari, K.; Rendell, A.; Burant, J. C.; Iyengar, S. S.; Tomasi, J.; Cossi, M.; Rega, N.; Millam, J. M.; Klene, M.; Knox, J. E.; Cross, J. B.; Bakken, V.; Adamo, C.; Jaramillo, J.; Gomperts, R.; Stratmann, R. E.; Yazyev, O.; Austin, A. J.; Cammi, R.; Pomelli, C.; Ochterski, J. W.; Martin, R. L.; Morokuma, K.; Zakrzewski, V. G.; Voth, G. A.; Salvador, P.; Dannenberg, J. J.; Dapprich, S.; Daniels, A. D.; Farkas, O.; Foresman, J. B.; Ortiz, J. V.; Cioslowski, J.; Fox, D. J. Gaussian, Inc., Wallingford CT, **2013**.

[3] (a) Cancès, E.; Mennucci, B.; Tomasi, J. *J. Chem. Phys*. **1997**, *107*, 3032–3047; (b) Cossi, M.; Barone, V.; Mennuci, B.; Tomasi, J. *Chem. Phys. Lett.* **1998**, *286*, 253–260; (c) Tomasi, J.; Mennucci, B.; Cancès, E. *J. Mol. Struct. (Theochem)* **1999**, *464*, 211–226.

[4] (a) Dolg, M.; Wedig, U.; Stoll, H.; Preuss, H. *J. Chem. Phys*. **1987**, *86*, 866–872; (b) Andrae, D.; Haussermann, U.; Dolg, M.; Stoll H.; Preuss, H. *Theor. Chim. Acta* **1990**, *77*, 123–141.

[5] Gonzalez, C.; Schlegel, H. B. *J. Phys. Chem*. **1990**, *94*, 5523–5527.

[6] Zhao, Y.; Truhlar, D. G. *Theor. Chem. Acc.* **2008**, *120*, 215–241.

DFT Studies of the Reaction Mechanism

For a better understanding of the mechanism we investigated the different reaction pathways with DFT calculations. Experimentally, the best results were achieved with palladium acetate [$Pd(OAc)_2$] in the presence of aromatic phosphines. For this system, the most logical mechanistic sequence would involve the coordination of the Pd(II) species to the substrate followed by a concerted metalation-deprotonation (CMD) step, by a transition state in which the reacting acetate group is acting as a κ^2 ligand. The other acetate shows monodentate coordination, and palladium binds also the alkyne, like in **TS1** (Figure 1). Next, the Pd–C bond formed in **8** adds to the triple bond through **TS2**, leading to the alkenyl–Pd(II) intermediate **9**. The final protodemetalation with acetic acid via **TS4** renders the adduct **2l**, recovering the active Pd(II)–acetate species.

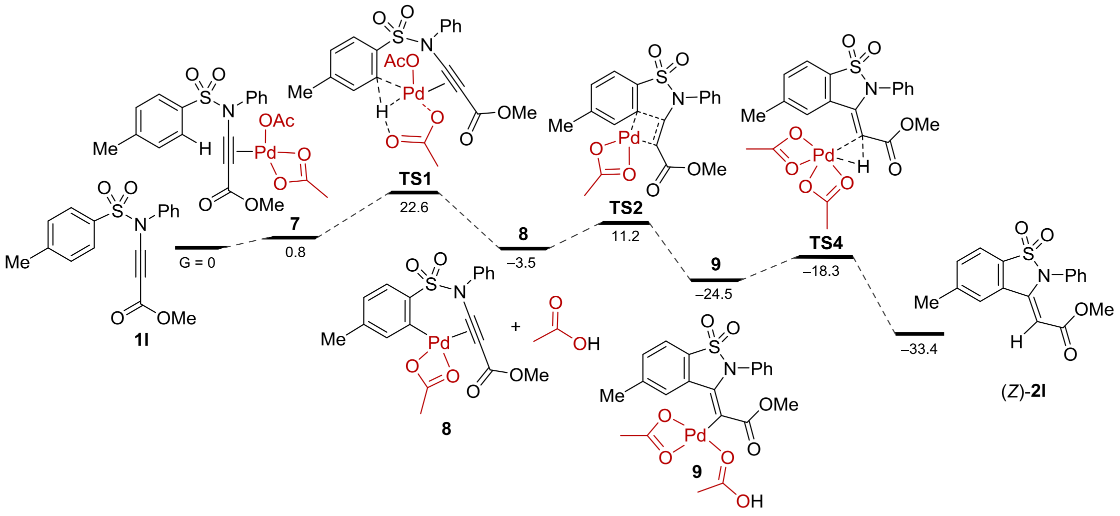

Figure 1. Computed Pathway for Cyclization of **1l** without Isomerization Step. Free Energies (298 K) with Respect to Starting Materials are Shown in kcal/mol.

The computed activation energies showed that the whole cycle is feasible. The initial coordination of Pd to the alkyne in **7** requires a change in denticity of one of the acetate ligands, from κ^2 to κ^1, but it is uphill in only 0.8 kcal/mol. Then, the transition state of the C–H abstraction has an activation barrier of 22.6 kcal/mol (from the separate reactants **1l** and palladium acetate), becoming rate determining step, because the following steps of the catalytic cycle, namely insertion and protonation, are much lower in energy, presenting affordable values (14.7 and 6.2 kcal/mol). Interestingly, this mechanism would only explain the formation of the experimental minor (*Z*)-**2l** isomer, with the ester moiety *cis* to the nitrogen atom. There must be a point during the catalytic cycle, where the (*Z*) and the (*E*)-isomers must interconvert. Intermediate **9** and also the final adduct **2** are good candidates, and the calculations showed that in both cases the *E*-species was thermodynamically more stable than the *Z*-isomer by 2.5 kcal/mol (**2**) and 2.0 kcal/mol (**9**; Figure 2). Thus, we hypothesize that equilibration processes would explain the formation of the experimental major isomers under thermodynamic conditions. To validate this idea, the two isomers of the final product were independently subjected to the experimental reaction conditions, giving rise to the product mixture, consisting of 85:15 *E*/*Z* ratio. Yet another plausible situation is that the equilibration

occurs at the intermediate **9** stage, probably by protonation with acetic acid and formation of an enol intermediate (**10**), allowing the free rotation of the internal CC bond. We found that the enol structure lies 23.1 kcal/mol higher in energy than the previous alkenyl–Pd intermediate (**9**), confirming the feasibility of this or related isomerization processes.

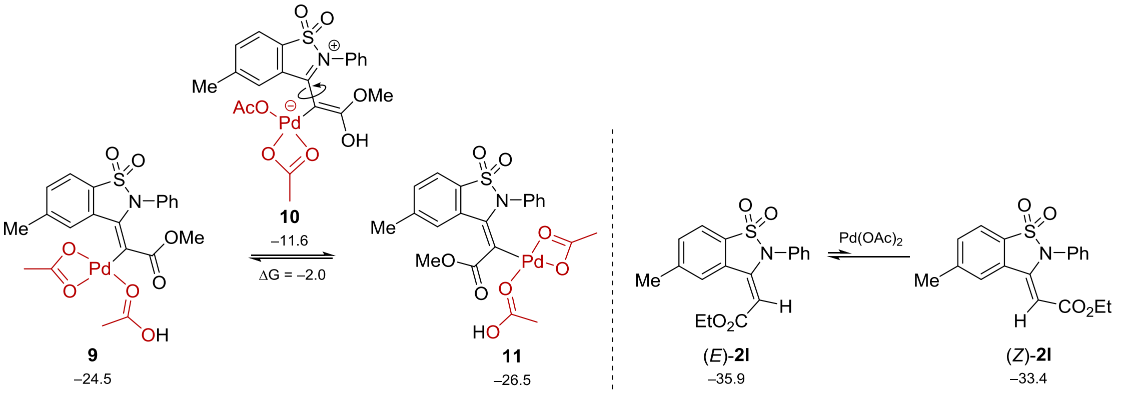

Figure 2. Equilibration Between (*E*)- and (*Z*)-isomers of **2l**. Free Energies (298 K) with Respect to Starting Materials are Shown in kcal/mol.

The experimental case where the palladium atom binds one phosphine molecule (modeled with one PPh_3) was also computationally analyzed, and a similar mechanism was found (Figure 3). For example, the activation energy of the CMD step almost equals the previous one (**TS5**, 23.7 kcal/mol), whilst the addition to the triple bond is slightly more facile, 9.7 kcal/mol (from 7.4 to 17.1 kcal/mol). Surprisingly, the presence of the phosphine disfavors the final protodemetalation to a large extent, increasing its activation energy from 6.2 kcal/mol (Figure 1) to 15.3 kcal/mol (**TS7**). Nonetheless, this effect does not alter the overall mechanism, since C–H activation in **TS5** is still rate limiting.

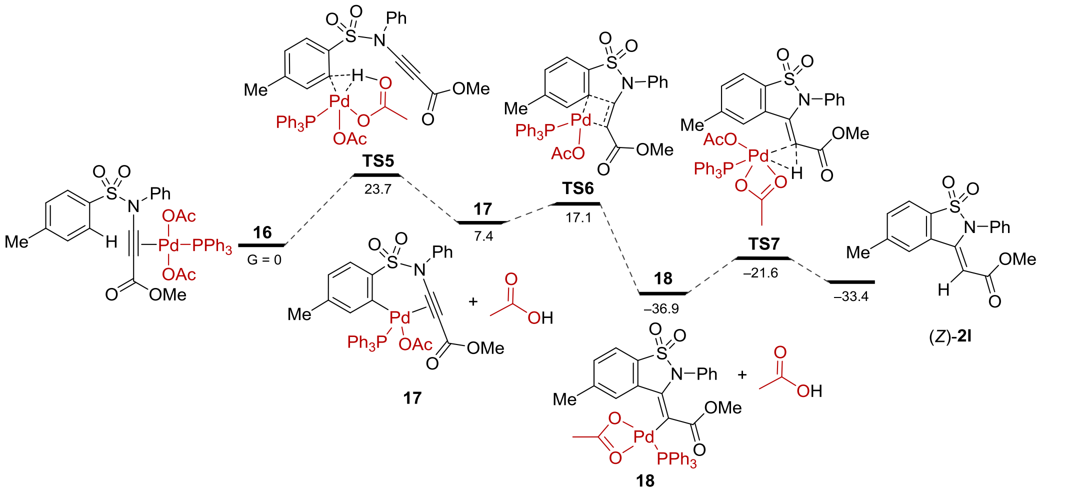

Figure 3. Computed Pathway for Cyclization of **16**. Free Energies (298 K) with Respect to Starting Materials are Shown in kcal/mol.

In the presence of phosphine (Figure 3), the proposed transition state for CMD (**TS5**) involves the decoordination of palladium from the alkyne during C–H abstraction, binding to three oxygens of the two acetate ligands. We checked also the possibility that the palladium center remains bound to the alkyne during the process. In this case, the spectator acetate must leave the coordination sphere of the metal, leaving a cationic species, like in **TS8** (Figure 4). This situation was proven to be highly disfavored, and the activation energy rises to an unaffordable value of 51.0 kcal/mol. Thus, **TS8** cannot compete with the neutral version **TS5** examined in Figure 3.

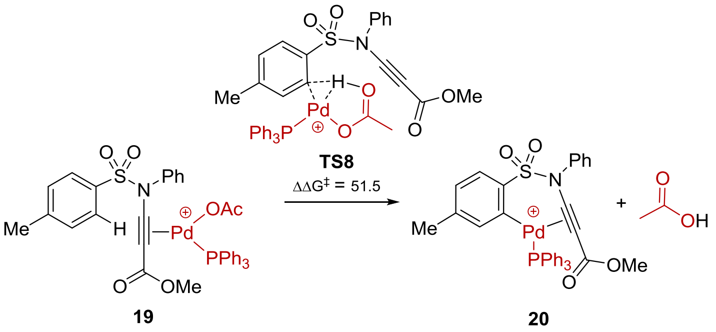

Figure 4. Computed Pathway for Cyclization of **19**. Free Energies (298 K) with Respect to Starting Materials are Shown in kcal/mol.

The most intriguing experimental data is the fact that Pd(0) species are also able to catalyze the process, to a similar extent of that of Pd(II) species, although with the formation of significant amounts of side products **3**–**5**. It is well known that the CMD processes are usually catalyzed by Pd(II) or Pd(IV) species but not Pd(0), and also that they need the presence of an internal or external basic ligand, like acetate in the case of $Pd(OAc)_2$. The fact that in the Pd(0) promoted reaction some of the side products are derived from the cleavage of the C–N bond of the starting material (like **3** and **4**), led us to hypothesize that the first step of this process could be the oxidative addition to the of Pd(0) to the alkyne–sulfonamide bond, like in **TS9** (Figure 5).

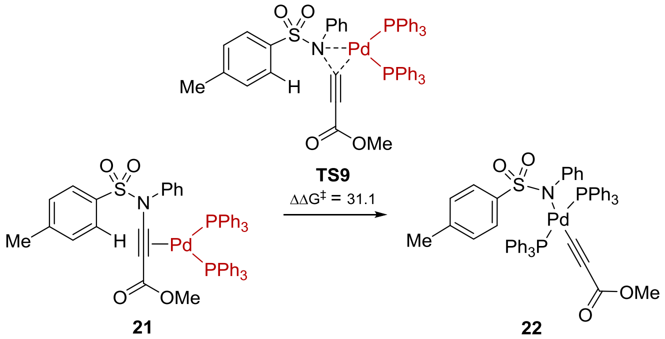

Figure 5. Computed Pathway for Insertion of Palladium(0) into C–N Bond. Free Energies (298 K) with Respect to Starting Materials are Shown in kcal/mol.

The computed activation energy of this step is moderate-high, but affordable at the reflux temperatures required for this process. The formation of palladium species **22** might explain the presence of active Pd(II) species in the reaction medium and the appearance of adducts lacking the N–alkyne bond.

Finally, the type of substitution at the terminal carbon of alkyne seems to exert a large influence on the reaction outcome. We first compared the reactivity of the methyl (computational model) and ethyl esters (experimental substrate), and gratefully found that the energies for the crucial steps do not differ significantly. The computed difference of 0.2 kcal/mol between both esters during the rate determining C–H activation transition state (Figure 6) is meaningless, validating the use of methyl ester as a model in our computational study.

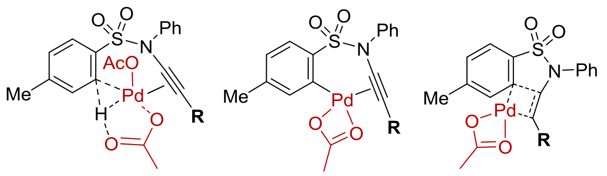

R	TS1	14	TS2
-CO_2Me	21.8	–4.4	10.3
-CO_2Et	**22.0**	**–4.4**	**10.2**
-H	24.5	–1.2	15.2
-$SiMe_3$	24.6	–2.8	13.4

Figure 6. Energy Values for the C–H Activation and Insertion Transition States for Different Substituents at Alkyne Terminus. Free Energies (298 K) with Respect to Starting Materials are Shown in kcal/mol.

A different outcome was obtained in the case of terminal alkyne (R = H) and silylated substrate (R = $SiMe_3$), which were found to show higher energy values in general. For the crucial C–H activation step, the barriers are >2.5 kcal/mol higher than for the ester derivatives (**TS1**, 24.5 and 24.6 kcal/mol), corresponding to more than 100 times slower reaction rate. This data are fully consistent with the absolute lack of reactivity shown by those substrates in the experimental conditions. Although not significant for the reaction rate, the insertion step was also predicted to be a few kcal/mol higher for terminal or silylated alkynes (**TS2**, Figure 6) than for ester based compounds.

Cartesian coordinates of the structures involved in the computational study

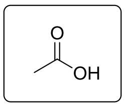

Thermal correction to Gibbs Free Energy = 0.034863 Hartree

Electronic energy = −228.893110039 Hartree

Cartesian Coordinates of the computed structure:

Center Number	Atomic Number	Atomic Type	Coordinates (Angstroms) X	Y	Z
1	8	0	−0.729181	−1.081247	−0.000775
2	6	0	−0.037289	0.014673	−0.008831
3	8	0	−0.705476	1.096956	−0.000861
4	6	0	1.452570	−0.017698	−0.001592
5	1	0	1.843575	0.838942	−0.553990
6	1	0	1.803632	0.056653	1.034166
7	1	0	1.818513	−0.952757	−0.429146
8	1	0	−2.480143	−0.050360	0.024599

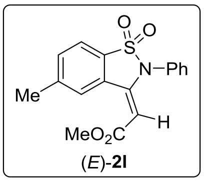

(*E*)-**2l**

Thermal correction to Gibbs Free Energy = 0.235865 Hartree

Electronic energy = −1410.37285199 Hartree

Center Number	Atomic Number	Atomic Type	Coordinates (Angstroms) X	Y	Z
1	6	0	1.329472	−1.735947	0.005756
2	6	0	2.402470	−2.614890	0.024929
3	6	0	3.684707	−2.070282	0.040197
4	6	0	1.475171	−0.343552	0.001619
5	6	0	2.773440	0.178767	0.015801
6	6	0	3.881310	−0.679355	0.033119
7	1	0	2.246386	−3.687988	0.030583
8	1	0	2.907405	1.252893	0.016124
9	8	0	−0.791747	−2.842686	1.242724
10	16	0	−0.370902	−2.219400	−0.012759
11	8	0	−0.755166	−2.855318	−1.275320

12	6	0	−2.266194	−0.282211	0.001079
13	6	0	−2.899770	−0.031506	1.223478
14	6	0	−2.997689	−0.305739	−1.189993
15	6	0	−4.271383	0.217555	1.245900
16	1	0	−2.315388	−0.038543	2.137210
17	6	0	−4.370552	−0.062686	−1.156601
18	1	0	−2.486889	−0.522225	−2.122383
19	6	0	−5.005378	0.201414	0.057859
20	1	0	−4.766741	0.412979	2.191208
21	1	0	−4.943286	−0.083710	−2.078454
22	1	0	−6.074369	0.390108	0.080383
23	7	0	−0.862840	−0.562837	−0.027453
24	6	0	0.174570	0.376895	−0.018607
25	6	0	−0.139000	1.704877	−0.036646
26	6	0	0.715040	2.888293	−0.029869
27	8	0	1.933310	2.982511	0.003157
28	8	0	−0.078545	3.995850	−0.067585
29	6	0	0.618571	5.247575	−0.065683
30	1	0	1.273652	5.328777	−0.937458
31	1	0	−0.155856	6.014174	−0.099009
32	1	0	1.224556	5.354480	0.837623
33	1	0	−1.194384	1.945385	−0.060857
34	6	0	5.279941	−0.111073	0.025650
35	1	0	5.934944	−0.652586	0.714909
36	1	0	5.726324	−0.186441	−0.973147
37	1	0	5.281324	0.944272	0.308715
38	1	0	4.545478	−2.732980	0.059132

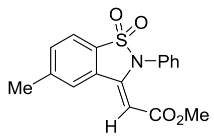

(*Z*)-**2l**

Thermal correction to Gibbs Free Energy = 0.237762 Hartree

Electronic energy = −1410.37083386 Hartree

Center Number	Atomic Number	Atomic Type	Coordinates (Angstroms) X	Y	Z
1	6	0	2.176606	−0.919096	−0.083294
2	6	0	3.500836	−1.340817	−0.046251

3	6	0	4.480887	−0.372895	0.144736
4	6	0	1.800834	0.414617	0.044215
5	6	0	2.805615	1.373600	0.219185
6	6	0	4.147851	0.988909	0.282220
7	1	0	3.758730	−2.388586	−0.161328
8	1	0	2.552138	2.423990	0.308154
9	8	0	0.503569	−2.807438	0.842492
10	16	0	0.759356	−1.954338	−0.317867
11	8	0	0.735532	−2.545275	−1.655394
12	6	0	−1.676991	−0.889268	0.092189
13	6	0	−2.154534	−0.607451	1.374290
14	6	0	−2.473239	−1.564049	−0.834433
15	6	0	−3.454410	−0.975719	1.715425
16	1	0	−1.514249	−0.103819	2.090189
17	6	0	−3.764455	−1.946672	−0.476039
18	1	0	−2.079637	−1.772593	−1.823397
19	6	0	−4.259292	−1.647797	0.793818
20	1	0	−3.831971	−0.751669	2.708889
21	1	0	−4.386961	−2.469878	−1.195643
22	1	0	−5.269302	−1.940098	1.066607
23	7	0	−0.322565	−0.572929	−0.264231
24	6	0	0.336816	0.638986	−0.076051
25	6	0	−0.195559	1.890173	−0.033272
26	6	0	−1.556300	2.308928	−0.375017
27	8	0	−2.415383	1.659512	−0.941560
28	8	0	−1.737629	3.610293	−0.009408
29	6	0	−3.014679	4.160191	−0.354930
30	1	0	−3.821762	3.602541	0.127266
31	1	0	−2.999143	5.190846	0.000196
32	1	0	−3.171312	4.131260	−1.437205
33	1	0	0.475817	2.700937	0.217103
34	6	0	5.234955	2.014260	0.495938
35	1	0	5.722032	1.872911	1.468194
36	1	0	6.013013	1.930728	−0.270254
37	1	0	4.837777	3.031802	0.465903
38	1	0	5.524743	−0.670593	0.185394

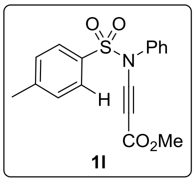

Thermal correction to Gibbs Free Energy = 0.231717 Hartree

Electronic energy = − 1410.31157817 Hartree

Center Number	Atomic Number	Atomic Type	Coordinates (Angstroms) X	Y	Z
1	6	0	1.383075	0.594862	0.801994
2	6	0	2.467014	0.073735	0.691994
3	6	0	−1.496116	−1.042802	0.678994
4	6	0	−2.687096	−0.874663	−0.032006
5	6	0	−3.058211	−1.852620	−0.947006
6	6	0	−0.679246	−2.159897	0.483994
7	6	0	−1.076359	−3.122851	−0.443006
8	6	0	−2.266343	−2.989712	−1.173006
9	1	0	−3.305995	−0.002591	0.145994
10	1	0	−0.449461	−3.995924	−0.596006
11	8	0	−0.202042	−0.410953	2.904994
12	16	0	−0.986970	0.207139	1.844994
13	8	0	−2.110866	1.101270	2.109994
14	6	0	−0.227749	2.102050	−0.092006
15	6	0	0.183229	1.912002	−1.413006
16	6	0	−1.024621	3.194143	0.262994
17	6	0	−0.211664	2.826048	−2.388006
18	1	0	0.826131	1.072927	−1.664006
19	6	0	−1.429517	4.089190	−0.726006
20	1	0	−1.319605	3.330177	1.295994
21	6	0	−1.024538	3.910143	−2.051006
22	1	0	0.114320	2.686010	−3.414006
23	1	0	−2.049418	4.937263	−0.455006
24	1	0	−1.332455	4.617179	−2.815006
25	7	0	0.196142	1.166000	0.921994
26	1	0	0.239739	−2.281005	1.048994
27	6	0	3.547917	−0.757391	0.238994
28	8	0	3.768786	−1.886417	0.624994
29	8	0	4.311995	−0.093480	−0.663006
30	6	0	5.434908	−0.839611	−1.166006

31	1	0	5.105798	−1.779573	−1.615006
32	1	0	5.900983	−0.197666	−1.913006
33	1	0	6.140882	−1.062694	−0.361006
34	6	0	−2.694465	−4.033662	−2.169006
35	1	0	−3.666516	−4.469549	−1.894006
36	1	0	−2.810415	−3.601649	−3.173006
37	1	0	−1.968561	−4.851747	−2.237006
38	1	0	−3.991197	−1.734511	−1.497006

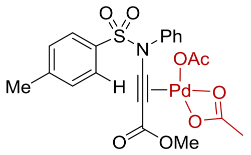

7

Thermal correction to Gibbs Free Energy = 0.317658 Hartree

Electronic energy = − 1995.493381 Hartree

Center Number	Atomic Number	Atomic Type	Coordinates (Angstroms) X	Y	Z
1	6	0	−2.558628	1.030949	−0.984744
2	6	0	−3.803569	0.919544	−0.360132
3	6	0	−4.194558	1.920848	0.525249
4	6	0	−1.701043	2.110750	−0.742913
5	6	0	−2.118748	3.094889	0.147737
6	6	0	−3.361316	3.015891	0.797324
7	46	0	1.603726	0.696712	0.492762
8	1	0	−4.451638	0.078333	−0.577849
9	1	0	−1.465404	3.940048	0.344321
10	8	0	−1.033676	0.244780	−3.025013
11	16	0	−2.038801	−0.248973	−2.095959
12	8	0	−3.184892	−1.053339	−2.505569
13	6	0	−1.749163	−2.225327	−0.026428
14	6	0	−1.512515	−2.062170	1.342596
15	6	0	−2.623442	−3.203097	−0.508458
16	6	0	−2.176338	−2.898439	2.240226
17	1	0	−0.797113	−1.323037	1.692611
18	6	0	−3.284622	−4.022323	0.406333
19	1	0	−2.778444	−3.316141	−1.573954
20	6	0	−3.064818	−3.871463	1.777141
21	1	0	−1.995970	−2.784751	3.304795

22	1	0	−3.963918	−4.786895	0.042508
23	1	0	−3.579256	−4.516859	2.482793
24	7	0	−1.055344	−1.365738	−0.965794
25	1	0	−0.738276	2.182242	−1.237887
26	6	0	0.208513	−1.146398	−0.913271
27	6	0	1.443846	−0.845471	−0.820695
28	6	0	2.622331	−1.476704	−1.490019
29	8	0	2.654660	−2.633499	−1.843232
30	8	0	3.609821	−0.589042	−1.654962
31	6	0	4.811923	−1.119691	−2.241633
32	1	0	5.230393	−1.894554	−1.595461
33	1	0	4.603772	−1.542531	−3.227395
34	1	0	5.492739	−0.273246	−2.320322
35	8	0	1.750214	2.365753	−0.769951
36	6	0	1.983851	3.135193	0.232571
37	8	0	1.950103	2.636796	1.400453
38	6	0	2.324845	4.581523	0.007993
39	1	0	2.070758	5.172680	0.889623
40	1	0	3.403278	4.665638	−0.164706
41	1	0	1.811153	4.962037	−0.877370
42	6	0	−3.777389	4.082930	1.778725
43	1	0	−4.839092	4.009937	2.027099
44	1	0	−3.208507	3.992218	2.711656
45	1	0	−3.586355	5.084594	1.380856
46	1	0	−5.163581	1.851269	1.010870
47	8	0	1.384045	−0.543252	2.053287
48	6	0	2.303188	−1.475533	2.211014
49	8	0	3.220362	−1.705299	1.430224
50	6	0	2.103378	−2.275708	3.491946
51	1	0	1.159626	−2.828218	3.439415
52	1	0	2.927954	−2.977702	3.618673
53	1	0	2.045270	−1.604432	4.353297

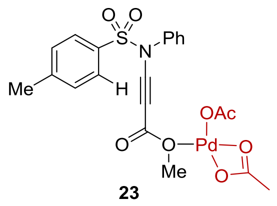

23

Thermal correction to Gibbs Free Energy = 0.318699 Hartree

Electronic energy = − 1995.18673069 Hartree

Center Number	Atomic Number	Atomic Type	Coordinates (Angstroms) X	Y	Z
1	6	0	2.050882	1.489119	0.920125
2	6	0	3.313858	2.087170	0.902125
3	6	0	3.436806	3.362175	0.356125
4	6	0	0.921856	2.130073	0.402125
5	6	0	1.074805	3.401079	−0.144875
6	6	0	2.325779	4.037130	−0.173875
7	46	0	−2.819035	−0.539079	0.191125
8	1	0	4.169879	1.568205	1.319125
9	1	0	0.192785	3.888044	−0.550875
10	8	0	0.528957	−0.341943	2.105125
11	16	0	1.878949	−0.147888	1.589125
12	8	0	3.068963	−0.489840	2.362125
13	6	0	3.254005	−1.523832	−0.424875
14	6	0	3.603983	−0.996818	−1.671875
15	6	0	4.131039	−2.356796	0.275125
16	6	0	4.840996	−1.316768	−2.226875
17	1	0	2.905957	−0.353846	−2.198875
18	6	0	5.373051	−2.656746	−0.283875
19	1	0	3.839055	−2.751808	1.240125
20	6	0	5.729030	−2.142732	−1.532875
21	1	0	5.112980	−0.916757	−3.198875
22	1	0	6.059077	−3.302718	0.255125
23	1	0	6.694040	−2.387692	−1.966875
24	7	0	1.961992	−1.214885	0.147125
25	1	0	−0.061125	1.674033	0.412125
26	6	0	0.865998	−1.356929	−0.585875
27	6	0	−0.123996	−1.508969	−1.281875
28	6	0	−1.166985	−1.774012	−2.203875
29	8	0	−1.067961	−2.363008	−3.254875
30	8	0	−2.398006	−1.253062	−1.792875
31	6	0	−3.508999	−1.435107	−2.717875
32	1	0	−3.159006	−1.249093	−3.732875
33	1	0	−3.884957	−2.456122	−2.631875
34	1	0	−4.252029	−0.703137	−2.408875
35	8	0	−2.082976	−2.006049	1.472125

36	6	0	−2.415005	−1.279062	2.469125
37	8	0	−3.037050	−0.187088	2.203125
38	6	0	−2.060990	−1.651048	3.871125
39	1	0	−1.033003	−1.323006	4.052125
40	1	0	−2.728010	−1.154075	4.578125
41	1	0	−2.104946	−2.735050	3.992125
42	6	0	2.465722	5.429136	−0.739875
43	1	0	3.496714	5.643178	−1.032875
44	1	0	1.822717	5.569110	−1.613875
45	1	0	2.170692	6.181124	0.002125
46	1	0	4.411787	3.841215	0.342125
47	8	0	−3.821097	0.968880	−0.669875
48	6	0	−3.167142	2.093907	−0.849875
49	8	0	−1.973149	2.264956	−0.610875
50	6	0	−4.056188	3.209871	−1.378875
51	1	0	−3.440221	4.041896	−1.722875
52	1	0	−4.693173	2.849845	−2.190875
53	1	0	−4.713202	3.556844	−0.574875

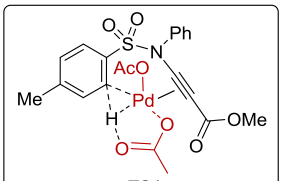

TS1

Thermal correction to Gibbs Free Energy = 0.313919 Hartree

Electronic energy = −1995.166717 Hartree

Frequency = −883.9

Center Number	Atomic Number	Atomic Type	Coordinates (Angstroms) X	Y	Z
1	6	0	0.034310	−2.372193	−0.155039
2	6	0	0.488725	−3.372219	−1.008952
3	6	0	1.864643	−3.601615	−1.103144
4	6	0	0.902312	−1.574091	0.628263
5	6	0	2.271054	−1.865618	0.511622
6	6	0	2.775652	−2.853245	−0.352273
7	1	0	−0.215122	−3.966905	−1.580220
8	1	0	2.971168	−1.295443	1.117756
9	8	0	−2.176571	−1.885772	1.305389
10	16	0	−1.742906	−2.174599	−0.055530

11	8	0	−2.411436	−3.182074	−0.876129
12	6	0	−3.241962	0.132941	−0.679319
13	6	0	−4.432239	−0.507889	−1.028691
14	6	0	−3.237072	1.395291	−0.079622
15	6	0	−5.642783	0.133313	−0.767872
16	1	0	−4.404663	−1.486236	−1.495553
17	6	0	−4.456096	2.028390	0.158403
18	1	0	−2.298278	1.872091	0.186512
19	6	0	−5.656888	1.399755	−0.179810
20	1	0	−6.574496	−0.356067	−1.033941
21	1	0	−4.464087	3.011995	0.617630
22	1	0	−6.602422	1.896755	0.014636
23	7	0	−1.991618	−0.540730	−0.953297
24	1	0	0.555519	−1.217994	1.816717
25	6	0	−0.911501	0.096750	−1.265036
26	6	0	0.251619	0.564839	−1.415060
27	6	0	1.154100	1.071835	−2.470760
28	8	0	0.870560	2.011905	−3.177571
29	8	0	2.297817	0.378456	−2.509206
30	6	0	3.330042	0.966317	−3.328674
31	1	0	2.985757	1.084874	−4.358160
32	1	0	3.604938	1.936465	−2.909700
33	1	0	4.168054	0.272054	−3.279786
34	8	0	1.236437	0.948618	2.612294
35	6	0	0.946460	0.044625	3.465384
36	8	0	0.497606	−1.100835	3.175173
37	6	0	1.135160	0.403374	4.923917
38	1	0	0.219985	0.889109	5.278817
39	1	0	1.302575	−0.495702	5.517990
40	1	0	1.959967	1.108181	5.037827
41	6	0	4.261465	−3.087308	−0.456767
42	1	0	4.698768	−3.286641	0.527474
43	1	0	4.491866	−3.933118	−1.109216
44	1	0	4.764852	−2.200929	−0.858714
45	1	0	2.226018	−4.381938	−1.767003
46	8	0	2.564223	2.698961	−0.398219
47	6	0	1.557592	3.278665	0.009238
48	8	0	0.495386	2.682465	0.498838
49	6	0	1.414580	4.793199	−0.038096

50	1	0	2.385319	5.246642	−0.242110
51	1	0	0.716829	5.062714	−0.837598
52	1	0	1.007636	5.172730	0.902258
53	46	0	0.706096	0.669165	0.625328

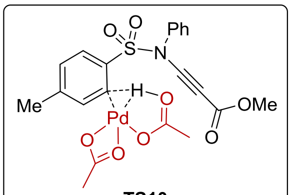

TS10

Thermal correction to Gibbs Free Energy = 0.317471 Hartree

Electronic energy = −1995.16857082 Hartree

Frequency = −648.6428

Center Number	Atomic Number	Atomic Type	Coordinates (Angstroms) X	Y	Z
1	6	0	−1.417717	1.498221	−0.600982
2	6	0	−2.716656	1.815470	−0.207982
3	6	0	−2.910486	2.707507	0.847018
4	6	0	−0.286607	2.076005	0.020018
5	6	0	−0.538429	3.007053	1.051018
6	6	0	−1.829369	3.317300	1.497018
7	1	0	−3.564738	1.389632	−0.732982
8	1	0	0.309668	3.513891	1.504018
9	8	0	0.095084	0.457932	−2.552982
10	16	0	−1.243934	0.364188	−1.985982
11	8	0	−2.453914	0.468419	−2.797982
12	6	0	−2.565326	−1.683559	−0.646982
13	6	0	−2.657346	−1.791542	0.744018
14	6	0	−3.648387	−2.004352	−1.469982
15	6	0	−3.850432	−2.238314	1.313018
16	1	0	−1.804295	−1.525705	1.360018
17	6	0	−4.840469	−2.434124	−0.885982
18	1	0	−3.550369	−1.910371	−2.543982
19	6	0	−4.942492	−2.556105	0.502018
20	1	0	−3.927449	−2.331299	2.392018
21	1	0	−5.686517	−2.684963	−1.518982
22	1	0	−5.869558	−2.900928	0.950018
23	7	0	−1.326242	−1.244796	−1.250982

24	1	0	0.678458	2.412820	−0.716982
25	6	0	−0.180360	−1.863016	−0.964982
26	6	0	0.852526	−2.459213	−0.721982
27	6	0	2.032396	−3.140439	−0.274982
28	8	0	2.042234	−3.985441	0.606018
29	8	0	3.127475	−2.728648	−0.941982
30	6	0	4.368369	−3.283885	−0.473982
31	1	0	4.545424	−2.992919	0.564018
32	1	0	4.354160	−4.374883	−0.545982
33	1	0	5.135449	−2.865032	−1.123982
34	8	0	2.838268	1.421407	−0.508982
35	6	0	2.687448	2.363436	−1.365982
36	8	0	1.630574	3.020638	−1.528982
37	6	0	3.885504	2.653207	−2.242982
38	1	0	3.810383	2.020221	−3.133982
39	1	0	3.873703	3.696209	−2.558982
40	1	0	4.813458	2.411030	−1.723982
41	6	0	−2.049186	4.275342	2.642018
42	1	0	−2.983079	4.833521	2.524018
43	1	0	−2.109290	3.733353	3.594018
44	1	0	−1.229049	4.993185	2.725018
45	1	0	−3.925439	2.950701	1.152018
46	8	0	0.250939	−0.302098	2.095018
47	6	0	1.299809	−0.978299	2.405018
48	8	0	2.388869	−0.668507	1.815018
49	6	0	1.240592	−2.115287	3.374018
50	1	0	2.107598	−2.081453	4.038018
51	1	0	0.314598	−2.082110	3.951018
52	1	0	1.290414	−3.048297	2.800018
53	46	0	1.340143	0.766694	0.671018

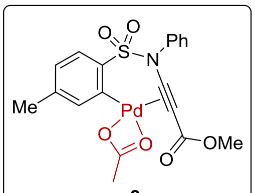

8

Thermal correction to Gibbs Free Energy = 0.264076 Hartree

Electronic energy = −1766.13116627 Hartree

Center Atomic Atomic Coordinates (Angstroms)

Number	Number	Type	X	Y	Z
1	6	0	−0.494751	2.093032	0.009408
2	6	0	−0.991885	3.268610	0.578096
3	6	0	−0.306094	3.827812	1.652230
4	6	0	0.657774	1.459869	0.484274
5	6	0	1.323297	2.040951	1.566120
6	6	0	0.854504	3.225003	2.160223
7	46	0	1.429846	−0.159089	−0.398141
8	1	0	−1.889165	3.727674	0.177614
9	1	0	2.226713	1.577084	1.950973
10	8	0	−0.460523	1.254022	−2.537256
11	16	0	−1.355710	1.402703	−1.393865
12	8	0	−2.674660	2.009660	−1.531700
13	6	0	−2.975920	−0.660454	−0.373926
14	6	0	−4.005829	−0.680719	−1.317930
15	6	0	−3.211028	−1.028748	0.953716
16	6	0	−5.286321	−1.062152	−0.919845
17	1	0	−3.802269	−0.400176	−2.344543
18	6	0	−4.492086	−1.425995	1.334492
19	1	0	−2.398834	−1.023591	1.672596
20	6	0	−5.532005	−1.438120	0.402675
21	1	0	−6.089830	−1.075973	−1.649927
22	1	0	−4.676478	−1.723360	2.362731
23	1	0	−6.528805	−1.744493	0.703600
24	7	0	−1.652076	−0.271947	−0.800286
25	6	0	−0.573879	−0.887729	−0.310616
26	6	0	0.136746	−1.682087	0.363192
27	6	0	0.474715	−2.846516	1.153028
28	8	0	−0.345822	−3.696330	1.439795
29	8	0	1.763734	−2.856554	1.536714
30	6	0	2.162387	−4.009889	2.300390
31	1	0	2.008788	−4.922467	1.720596
32	1	0	3.219899	−3.862602	2.511814
33	1	0	1.587799	−4.073281	3.228286
34	8	0	3.299348	0.636639	−0.935650
35	6	0	3.657560	−0.465020	−1.501750
36	8	0	2.880692	−1.463912	−1.447882
37	6	0	4.968572	−0.523242	−2.232938

38	1	0	4.856498	−0.026302	−3.201730
39	1	0	5.736601	0.012012	−1.670493
40	1	0	5.265523	−1.559907	−2.397810
41	6	0	1.576476	3.819667	3.345669
42	1	0	1.431962	4.902176	3.398846
43	1	0	1.203491	3.392390	4.284433
44	1	0	2.650026	3.618717	3.299878
45	1	0	−0.674491	4.746713	2.100233

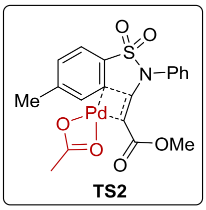

Thermal correction to Gibbs Free Energy = 0.264425 Hartree

Electronic energy = −1766.10806708 Hartree

Frequency = −295.0891

Center Number	Atomic Number	Atomic Type	Coordinates (Angstroms) X	Y	Z
1	6	0	0.440075	2.371187	−0.087505
2	6	0	0.872794	3.650874	0.245060
3	6	0	1.944212	3.778938	1.126685
4	6	0	1.011795	1.220154	0.457281
5	6	0	2.009083	1.381349	1.433114
6	6	0	2.515842	2.652839	1.740863
7	46	0	1.291333	−0.670560	−0.369726
8	1	0	0.358245	4.520123	−0.150800
9	1	0	2.415160	0.512196	1.942529
10	8	0	−0.916332	1.556549	−2.286069
11	16	0	−1.089434	2.118339	−0.946989
12	8	0	−1.996488	3.243327	−0.735501
13	6	0	−2.900709	0.255929	−0.086212
14	6	0	−3.074441	−0.884716	−0.875597
15	6	0	−3.994711	0.929123	0.461576
16	6	0	−4.364324	−1.362252	−1.100442
17	1	0	−2.213604	−1.386940	−1.301728
18	6	0	−5.281561	0.446825	0.219668
19	1	0	−3.828204	1.815684	1.062997
20	6	0	−5.466390	−0.698947	−0.555841

21	1	0	−4.507821	−2.250901	−1.707799
22	1	0	−6.136256	0.965062	0.642071
23	1	0	−6.468651	−1.075404	−0.735905
24	7	0	−1.586317	0.807019	0.155870
25	6	0	−0.558207	−0.003923	0.531802
26	6	0	−0.209129	−1.184966	0.930163
27	6	0	−0.600479	−2.338273	1.720861
28	8	0	−1.695587	−2.455754	2.240477
29	8	0	0.385012	−3.253399	1.804972
30	6	0	0.056609	−4.437506	2.550458
31	1	0	−0.788619	−4.956900	2.090825
32	1	0	0.950895	−5.058074	2.517699
33	1	0	−0.201656	−4.184346	3.581389
34	8	0	2.866228	−0.449032	−1.847829
35	6	0	2.918718	−1.716884	−1.961126
36	8	0	2.171936	−2.437693	−1.213286
37	6	0	3.815898	−2.360239	−2.982349
38	1	0	3.270122	−2.443396	−3.929063
39	1	0	4.699494	−1.742427	−3.152119
40	1	0	4.101687	−3.363581	−2.660532
41	6	0	3.659067	2.803932	2.714159
42	1	0	4.619340	2.810607	2.184266
43	1	0	3.587135	3.741013	3.273342
44	1	0	3.686617	1.977563	3.429654
45	1	0	2.306970	4.770295	1.383508

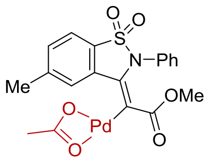

9

Thermal correction to Gibbs Free Energy = 0.268663 Hartree

Electronic energy = −1766.16566003 Hartree

Center	Atomic	Atomic	Coordinates (Angstroms)		
Number	Number	Type	X	Y	Z
1	6	0	1.306060	2.727472	−0.077009
2	6	0	1.147991	4.106791	−0.058142
3	6	0	−0.150294	4.609661	−0.102566
4	6	0	0.231902	1.838145	−0.154981

5	6	0	−1.060956	2.365828	−0.227207
6	6	0	−1.260923	3.751164	−0.189277
7	46	0	−2.118123	−1.160572	−0.036087
8	1	0	2.006757	4.768171	−0.006701
9	1	0	−1.915647	1.707380	−0.295846
10	8	0	3.512006	1.932424	1.244354
11	16	0	2.849021	1.867332	−0.058809
12	8	0	3.630974	2.126752	−1.267979
13	6	0	2.801921	−0.805434	0.246454
14	6	0	2.545492	−1.386014	1.493167
15	6	0	3.854456	−1.263653	−0.550293
16	6	0	3.332038	−2.451254	1.927057
17	1	0	1.741660	−1.000818	2.112208
18	6	0	4.649910	−2.315977	−0.096914
19	1	0	4.030834	−0.799403	−1.514095
20	6	0	4.386892	−2.915004	1.135790
21	1	0	3.135236	−2.904577	2.893928
22	1	0	5.468763	−2.672762	−0.713889
23	1	0	5.002926	−3.739881	1.482265
24	7	0	2.036521	0.321237	−0.201896
25	6	0	0.639730	0.416341	−0.232658
26	6	0	−0.192737	−0.660248	−0.365448
27	6	0	0.214896	−1.958785	−0.873594
28	8	0	1.093130	−2.385104	−1.573123
29	8	0	−0.892413	−2.806568	−0.487882
30	6	0	−1.144199	−3.986682	−1.283953
31	1	0	−0.250464	−4.611842	−1.269684
32	1	0	−1.982575	−4.494745	−0.809090
33	1	0	−1.390585	−3.706122	−2.310836
34	8	0	−4.214683	−1.608067	0.387300
35	6	0	−4.389190	−0.374397	0.605545
36	8	0	−3.379458	0.417356	0.470842
37	6	0	−5.715350	0.186057	1.030184
38	1	0	−5.980205	1.037737	0.397877
39	1	0	−6.486635	−0.582496	0.968941
40	1	0	−5.641692	0.548815	2.060379
41	6	0	−2.659059	4.317515	−0.264049
42	1	0	−2.794796	5.137333	0.447654
43	1	0	−2.865494	4.717620	−1.263992

44	1	0	−3.405445	3.548249	−0.051116
45	1	0	−0.306870	5.684175	−0.074485

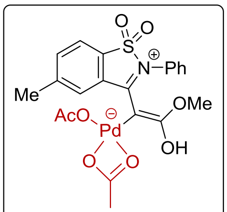

10

Thermal correction to Gibbs Free Energy = 0.325937 Hartree

Electronic energy = −1995.233304 Hartree

Center Number	Atomic Number	Atomic Type	Coordinates (Angstroms) X	Y	Z
1	6	0	−0.380517	−0.540924	−0.642359
2	6	0	0.701341	0.336743	−0.492069
3	8	0	−3.429118	−1.332121	−1.033428
4	6	0	−4.472958	−1.120888	−0.279522
5	8	0	−4.360819	−0.356255	0.705913
6	6	0	−5.766844	−1.792391	−0.644408
7	1	0	−6.547207	−1.520660	0.066564
8	1	0	−6.061634	−1.495537	−1.655419
9	1	0	−5.628669	−2.877949	−0.648602
10	46	0	−2.180280	−0.174070	0.204257
11	6	0	0.572342	1.812787	−0.555742
12	6	0	1.781981	2.496989	−0.423690
13	6	0	−0.577484	2.552786	−0.843025
14	6	0	1.894553	3.874414	−0.552749
15	6	0	−0.513792	3.946655	−0.952125
16	1	0	−1.525097	2.037421	−0.961525
17	6	0	0.726562	4.592895	−0.806037
18	1	0	2.853941	4.372641	−0.462786
19	1	0	0.778288	5.673765	−0.901529
20	16	0	3.115767	1.371575	−0.165682
21	8	0	4.047615	1.366862	−1.293285
22	8	0	3.634101	1.397369	1.199554
23	6	0	2.545064	−1.266901	0.156860
24	6	0	3.666363	−1.794649	−0.492636
25	6	0	1.990462	−1.900277	1.274237
26	6	0	4.236512	−2.976362	−0.019631

27	1	0	4.079506	−1.281315	−1.353796
28	6	0	2.562705	−3.091159	1.721281
29	1	0	1.126082	−1.484342	1.784556
30	6	0	3.682494	−3.630105	1.082470
31	1	0	5.111578	−3.384823	−0.516274
32	1	0	2.135146	−3.587300	2.587262
33	1	0	4.125307	−4.552463	1.446886
34	7	0	2.013928	−0.016780	−0.310433
35	6	0	−0.244764	−1.767520	−1.307013
36	8	0	−1.214940	−2.640354	−1.477802
37	8	0	0.885895	−2.094366	−1.917022
38	6	0	0.955675	−3.329057	−2.656997
39	1	0	0.240338	−3.321438	−3.482124
40	1	0	1.977171	−3.370570	−3.031682
41	1	0	0.755006	−4.179639	−2.002862
42	6	0	−1.770243	4.745625	−1.199484
43	1	0	−2.484678	4.185962	−1.809496
44	1	0	−2.267576	4.981998	−0.251099
45	1	0	−1.552738	5.691305	−1.703707
46	1	0	−2.098390	−2.276489	−1.197614
47	8	0	−0.742714	−0.884630	2.764831
48	6	0	−0.852414	0.338273	2.668123
49	8	0	−1.337319	0.975608	1.628578
50	6	0	−0.372515	1.277297	3.767357
51	1	0	0.609993	1.677534	3.492988
52	1	0	−1.055454	2.121269	3.887845
53	1	0	−0.276552	0.727794	4.704775

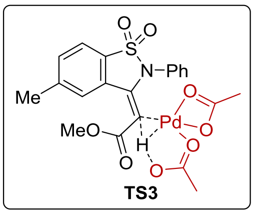

Thermal correction to Gibbs Free Energy = 0.318895 Hartree

Electronic energy = −1955.233926 Hartree

Frequencies: −1129.7

Center Number	Atomic Number	Atomic Type	Coordinates (Angstroms) X	Y	Z

1	6	0	0.009990	−0.803348	−0.778371
2	6	0	−0.988826	0.142942	−0.509585
3	1	0	0.986569	−0.405329	−1.626969
4	8	0	3.097010	−0.768272	−0.574991
5	6	0	3.021261	−0.488643	−1.808094
6	8	0	1.937268	−0.265776	−2.438007
7	6	0	4.315545	−0.381591	−2.580842
8	1	0	4.193908	−0.815326	−3.575369
9	1	0	4.556624	0.679408	−2.704170
10	1	0	5.126707	−0.869673	−2.040788
11	8	0	0.261447	−1.085041	2.352519
12	6	0	1.264097	−1.234068	3.146939
13	8	0	2.434213	−1.176261	2.660487
14	6	0	1.026015	−1.497918	4.606449
15	1	0	0.175738	−0.911662	4.962268
16	1	0	0.785301	−2.557757	4.742769
17	1	0	1.922573	−1.264562	5.182717
18	46	0	1.504676	−0.895960	0.706867
19	6	0	−2.469049	−0.036346	−0.381821
20	6	0	−3.151806	1.147640	−0.074549
21	6	0	−3.222580	−1.214290	−0.439044
22	6	0	−4.518632	1.213936	0.147975
23	6	0	−4.607474	−1.190015	−0.222731
24	1	0	−2.747767	−2.152835	−0.679450
25	6	0	−5.245218	0.027808	0.058044
26	1	0	−5.002366	2.155082	0.387108
27	1	0	−6.319812	0.046320	0.215807
28	16	0	−2.042571	2.508440	0.072739
29	8	0	−2.149842	3.458689	−1.033139
30	8	0	−1.966660	3.011693	1.443260
31	6	0	0.599041	2.107849	−0.268050
32	6	0	1.114095	2.563985	−1.484236
33	6	0	1.253718	2.378790	0.939427
34	6	0	2.317693	3.269291	−1.491572
35	1	0	0.574727	2.367985	−2.403637
36	6	0	2.455299	3.086148	0.919970
37	1	0	0.812956	2.043001	1.870801
38	6	0	2.988293	3.526966	−0.293519
39	1	0	2.721737	3.629154	−2.432909

40	1	0	2.970485	3.294900	1.852158
41	1	0	3.921679	4.082209	−0.304268
42	7	0	−0.677786	1.455336	−0.233368
43	6	0	−0.327603	−2.125367	−1.369111
44	8	0	−1.221108	−2.367671	−2.165517
45	8	0	0.586507	−3.067381	−1.021665
46	6	0	0.440624	−4.339591	−1.672125
47	1	0	0.495869	−4.225469	−2.757659
48	1	0	1.265874	−4.949830	−1.305982
49	1	0	−0.517498	−4.798353	−1.414837
50	6	0	−5.397451	−2.476161	−0.272263
51	1	0	−6.444683	−2.292755	−0.527870
52	1	0	−4.980906	−3.169425	−1.008028
53	1	0	−5.380081	−2.981244	0.701253

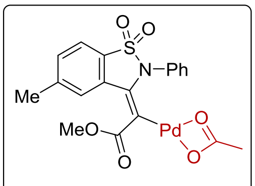

11

Thermal correction to Gibbs Free Energy = 0.268213 Hartree

Electronic energy = −1766.17974491 Hartree

Center Number	Atomic Number	Atomic Type	Coordinates (Angstroms) X	Y	Z
1	6	0	3.214091	−0.900988	−0.196284
2	6	0	4.599902	−1.004364	−0.247890
3	6	0	5.339603	0.171072	−0.162543
4	6	0	2.540535	0.319681	−0.063220
5	6	0	3.309836	1.487235	0.029035
6	6	0	4.706090	1.420204	−0.022522
7	46	0	−1.797844	0.222359	0.016406
8	1	0	5.084453	−1.969603	−0.352905
9	1	0	2.825850	2.444756	0.167548
10	8	0	2.041953	−2.988163	1.045260
11	16	0	2.090584	−2.271694	−0.234948
12	8	0	2.191206	−3.055373	−1.467664
13	6	0	−0.549961	−1.687725	−0.003439
14	6	0	−1.042380	−1.564619	1.331838

15	6	0	−1.199584	−2.565189	−0.913376
16	6	0	−2.180779	−2.305832	1.718502
17	1	0	−0.446392	−1.048410	2.074773
18	6	0	−2.319297	−3.268520	−0.505223
19	1	0	−0.782724	−2.676811	−1.907186
20	6	0	−2.809421	−3.141150	0.809744
21	1	0	−2.539197	−2.230109	2.740131
22	1	0	−2.818078	−3.932988	−1.203121
23	1	0	−3.681600	−3.712136	1.113847
24	7	0	0.746141	−1.188415	−0.351397
25	6	0	1.070728	0.161376	−0.054495
26	6	0	0.038379	1.023165	0.107308
27	6	0	0.189480	2.464215	0.404853
28	8	0	0.899514	2.936608	1.280073
29	8	0	−0.573270	3.222990	−0.404176
30	6	0	−0.570655	4.630790	−0.123697
31	1	0	0.429408	5.051334	−0.259330
32	1	0	−1.270848	5.069594	−0.833234
33	1	0	−0.895916	4.818152	0.903065
34	8	0	−3.943729	−0.170714	−0.196283
35	6	0	−4.116000	1.069867	−0.406998
36	8	0	−3.093849	1.845586	−0.387362
37	6	0	−5.475469	1.640346	−0.698021
38	1	0	−5.530357	1.914171	−1.757445
39	1	0	−6.251160	0.905906	−0.476618
40	1	0	−5.632533	2.549357	−0.112980
41	6	0	5.533731	2.680240	0.059609
42	1	0	6.364092	2.564642	0.764585
43	1	0	5.970451	2.927068	−0.915762
44	1	0	4.931740	3.532970	0.381085
45	1	0	6.423709	0.123279	−0.200039

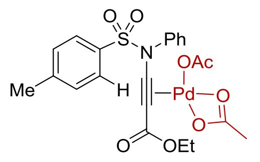

7 (R = CO_2Et)

Thermal correction to Gibbs Free Energy = 0.343787 Hartree

Electronic energy = −2034.505010 Hartree

Standard orientation:

Center Number	Atomic Number	Atomic Type	Coordinates (Angstroms) X	Y	Z
1	6	0	−2.637399	1.051934	−1.126293
2	6	0	−3.929036	0.950132	−0.603149
3	6	0	−4.379696	1.951396	0.253459
4	6	0	−1.791476	2.122038	−0.811438
5	6	0	−2.269299	3.106358	0.048285
6	6	0	−3.560327	3.036896	0.596543
7	46	0	1.396327	0.677409	0.667259
8	1	0	−4.565551	0.116175	−0.875902
9	1	0	−1.625558	3.944012	0.300726
10	8	0	−0.964538	0.259894	−3.044672
11	16	0	−2.043119	−0.228543	−2.198905
12	8	0	−3.160696	−1.020949	−2.700408
13	6	0	−1.932352	−2.212142	−0.119302
14	6	0	−1.797167	−2.056163	1.264270
15	6	0	−2.778064	−3.179006	−0.669490
16	6	0	−2.534880	−2.888424	2.106198
17	1	0	−1.102125	−1.326044	1.669926
18	6	0	−3.514320	−3.994231	0.189869
19	1	0	−2.853841	−3.286968	−1.744046
20	6	0	−3.396201	−3.850400	1.573899
21	1	0	−2.433459	−2.780308	3.181743
22	1	0	−4.172262	−4.750385	−0.226950
23	1	0	−3.968607	−4.492744	2.236380
24	7	0	−1.160574	−1.357173	−1.000248
25	1	0	−0.791665	2.186004	−1.227773
26	6	0	0.097275	−1.148724	−0.848499
27	6	0	1.324537	−0.861023	−0.658499
28	6	0	2.546262	−1.503589	−1.236579
29	8	0	2.589073	−2.661215	−1.587867
30	8	0	3.552152	−0.628169	−1.320134
31	6	0	4.806669	−1.163008	−1.810784
32	1	0	5.100249	−1.992754	−1.161458
33	1	0	4.647752	−1.560847	−2.818065
34	8	0	1.654840	2.347654	−0.576559
35	6	0	1.815884	3.113458	0.442857

36	8	0	1.688344	2.613521	1.603522
37	6	0	2.183495	4.557782	0.248607
38	1	0	1.865581	5.149294	1.109044
39	1	0	3.272656	4.634724	0.160661
40	1	0	1.742884	4.943387	−0.673192
41	6	0	−4.042976	4.103763	1.547180
42	1	0	−5.121753	4.040301	1.709835
43	1	0	−3.551136	4.003353	2.521974
44	1	0	−3.811259	5.105192	1.170902
45	1	0	−5.384878	1.889277	0.660193
46	8	0	1.044390	−0.564422	2.202449
47	6	0	1.950511	−1.493306	2.436882
48	8	0	2.933556	−1.715627	1.738452
49	6	0	1.643592	−2.298692	3.693127
50	1	0	0.707029	−2.849808	3.559911
51	1	0	2.454261	−3.001921	3.885683
52	1	0	1.514409	−1.630700	4.549351
53	6	0	5.817234	−0.033463	−1.792135
54	1	0	6.783096	−0.397481	−2.156470
55	1	0	5.494394	0.791183	−2.434126
56	1	0	5.952095	0.348145	−0.776341

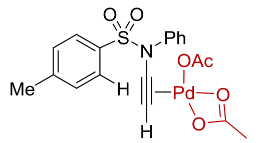

7 (R = H)

Thermal correction to Gibbs Free Energy = 0.281097 Hartree

Electronic energy = −1767.358795 Hartree

Center Number	Atomic Number	Atomic Type	Coordinates (Angstroms) X	Y	Z
1	6	0	−1.472988	2.131217	−0.395385
2	6	0	−2.424403	2.490510	0.562586
3	6	0	−2.013664	3.249316	1.655851
4	6	0	−0.129227	2.507474	−0.282823
5	6	0	0.251566	3.264231	0.821046
6	6	0	−0.675583	3.641958	1.805838
7	46	0	2.016552	−0.667096	−0.223434
8	1	0	−3.460393	2.195292	0.441541

9	1	0	1.290346	3.565714	0.920207
10	8	0	−1.071187	1.321849	−2.902620
11	16	0	−1.980068	1.144858	−1.779796
12	8	0	−3.432907	1.180303	−1.913687
13	6	0	−2.469617	−1.222433	−0.228536
14	6	0	−1.940889	−1.626855	1.002165
15	6	0	−3.810765	−1.440976	−0.555327
16	6	0	−2.782470	−2.256415	1.919149
17	1	0	−0.886072	−1.484614	1.221095
18	6	0	−4.638147	−2.063494	0.379266
19	1	0	−4.193559	−1.128078	−1.518418
20	6	0	−4.128706	−2.469691	1.614309
21	1	0	−2.380317	−2.578227	2.874927
22	1	0	−5.680903	−2.239146	0.133712
23	1	0	−4.777997	−2.958139	2.334703
24	7	0	−1.594022	−0.578860	−1.188338
25	1	0	0.597867	2.221709	−1.035310
26	6	0	−0.418848	−1.020265	−1.469871
27	6	0	0.785400	−1.377444	−1.663912
28	8	0	2.874628	0.997546	−1.158763
29	6	0	3.672602	1.183348	−0.166306
30	8	0	3.567708	0.426624	0.846274
31	6	0	4.724201	2.254733	−0.240511
32	1	0	5.005761	2.579366	0.762759
33	1	0	5.612433	1.842781	−0.731721
34	1	0	4.369759	3.097839	−0.837256
35	6	0	−0.230484	4.440107	3.005786
36	1	0	−1.081827	4.845111	3.558471
37	1	0	0.347378	3.812395	3.694450
38	1	0	0.414727	5.273766	2.711025
39	1	0	−2.745139	3.542895	2.403162
40	8	0	1.411352	−2.073671	1.068627
41	6	0	1.579900	−3.332217	0.712992
42	8	0	1.984645	−3.713451	−0.379872
43	6	0	1.197107	−4.302749	1.824122
44	1	0	0.127543	−4.215640	2.041127
45	1	0	1.423436	−5.323406	1.514320
46	1	0	1.739558	−4.060287	2.742190
47	1	0	1.231418	−1.984365	−2.440242

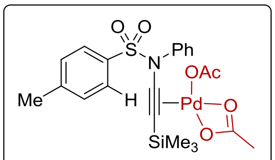

7 (R = $SiMe_3$)

Thermal correction to Gibbs Free Energy = 0.374180 Hartree

Electronic energy = −2176.002677 Hartree

Center Number	Atomic Number	Atomic Type	Coordinates (Angstroms) X	Y	Z
1	6	0	−2.667700	0.869833	−1.036359
2	6	0	−3.907806	0.732615	−0.408243
3	6	0	−4.360387	1.768056	0.406309
4	6	0	−1.875890	2.011398	−0.866654
5	6	0	−2.353027	3.029500	−0.046401
6	6	0	−3.592432	2.924495	0.605057
7	46	0	1.427922	0.892391	0.553640
8	1	0	−4.506355	−0.156557	−0.570169
9	1	0	−1.749197	3.921492	0.093815
10	8	0	−1.091873	0.038502	−3.014981
11	16	0	−2.066467	−0.456300	−2.052519
12	8	0	−3.171957	−1.336175	−2.419737
13	6	0	−1.710072	−2.297635	0.088998
14	6	0	−1.544659	−2.045056	1.455120
15	6	0	−2.481739	−3.371286	−0.364945
16	6	0	−2.169781	−2.887609	2.374814
17	1	0	−0.909765	−1.228688	1.787883
18	6	0	−3.108614	−4.195200	0.569666
19	1	0	−2.589048	−3.549954	−1.427428
20	6	0	−2.955289	−3.956097	1.936946
21	1	0	−2.042294	−2.702297	3.437109
22	1	0	−3.709435	−5.031218	0.225044
23	1	0	−3.441820	−4.604929	2.659165
24	7	0	−1.052426	−1.436424	−0.872954
25	1	0	−0.917062	2.102664	−1.365347
26	6	0	0.199255	−1.103329	−0.777934
27	6	0	1.409946	−0.744732	−0.703225
28	8	0	1.629687	2.452349	−0.828008
29	6	0	1.752414	3.316945	0.117449

30	8	0	1.644790	2.921784	1.318107
31	6	0	2.052588	4.752132	−0.213139
32	1	0	1.711256	5.405512	0.591721
33	1	0	3.136601	4.870795	−0.317407
34	1	0	1.589788	5.030380	−1.162355
35	6	0	−4.073758	4.030383	1.511114
36	1	0	−5.130681	3.913014	1.763213
37	1	0	−3.505038	4.036985	2.448520
38	1	0	−3.939661	5.012105	1.045805
39	1	0	−5.326993	1.676691	0.893348
40	8	0	1.139427	−0.242025	2.183796
41	6	0	2.144886	−0.993884	2.573427
42	8	0	3.213552	−1.115511	1.979017
43	6	0	1.850301	−1.734602	3.871989
44	1	0	1.021306	−2.433659	3.720401
45	1	0	2.735779	−2.284315	4.192503
46	1	0	1.547243	−1.027783	4.649601
47	14	0	3.042114	−1.314476	−1.513709
48	6	0	4.390829	−0.062370	−1.139569
49	1	0	5.331453	−0.356146	−1.620236
50	1	0	4.118254	0.933324	−1.503505
51	1	0	4.550161	−0.010656	−0.058911
52	6	0	2.690712	−1.402995	−3.368469
53	1	0	3.591934	−1.707160	−3.913706
54	1	0	1.900472	−2.125947	−3.595736
55	1	0	2.373412	−0.430450	−3.757904
56	6	0	3.444509	−3.012321	−0.804375
57	1	0	4.376351	−3.394004	−1.237819
58	1	0	3.572252	−2.932898	0.278655
59	1	0	2.652813	−3.738716	−1.015818

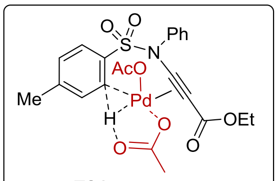

TS1 (R = CO_2Et)

Thermal correction to Gibbs Free Energy = 0.340697 Hartree

Electronic energy = −2034.466795 Hartree

Center Number	Atomic Number	Atomic Type	Coordinates (Angstroms) X	Y	Z

1	6	0	−0.099919	−2.379315	−0.138414
2	6	0	0.445594	−3.372144	−0.946720
3	6	0	1.818809	−3.620857	−0.871415
4	6	0	0.672785	−1.607424	0.761555
5	6	0	2.042289	−1.916167	0.810855
6	6	0	2.638497	−2.897322	0.000337
7	1	0	−0.189028	−3.948335	−1.610519
8	1	0	2.667046	−1.368357	1.512311
9	8	0	−2.469993	−1.902287	1.050723
10	16	0	−1.874555	−2.164187	−0.253295
11	8	0	−2.444540	−3.146458	−1.172899
12	6	0	−3.268153	0.170012	−0.998204
13	6	0	−4.410875	−0.449986	−1.507384
14	6	0	−3.328141	1.417059	−0.370195
15	6	0	−5.639893	0.196483	−1.380300
16	1	0	−4.332859	−1.416596	−1.992617
17	6	0	−4.562873	2.056098	−0.267093
18	1	0	−2.425882	1.878075	0.020808
19	6	0	−5.717400	1.447990	−0.765813
20	1	0	−6.535227	−0.276883	−1.771050
21	1	0	−4.620424	3.028087	0.212874
22	1	0	−6.676249	1.949356	−0.675772
23	7	0	−1.998352	−0.509847	−1.134162
24	1	0	0.185772	−1.274192	1.904889
25	6	0	−0.883681	0.125269	−1.294396
26	6	0	0.289179	0.592593	−1.292138
27	6	0	1.308347	1.132777	−2.219063
28	8	0	1.091480	2.080716	−2.939985
29	8	0	2.462088	0.465677	−2.123865
30	6	0	3.590183	1.091840	−2.793395
31	1	0	3.372363	1.158956	−3.863385
32	1	0	3.688139	2.102994	−2.390242
33	8	0	0.782106	0.863656	2.833604
34	6	0	0.384876	−0.057733	3.622706
35	8	0	−0.036418	−1.190017	3.251601
36	6	0	0.402813	0.262925	5.102121
37	1	0	−0.540871	0.756482	5.357618
38	1	0	0.484877	−0.652701	5.688757

39	1	0	1.218290	0.950297	5.331555
40	6	0	4.123735	−3.147315	0.067227
41	1	0	4.479891	−3.147995	1.101987
42	1	0	4.392527	−4.103278	−0.389571
43	1	0	4.669292	−2.357641	−0.462602
44	1	0	2.250036	−4.398301	−1.495890
45	8	0	2.446840	2.712730	0.058046
46	6	0	1.391829	3.271927	0.356744
47	8	0	0.284363	2.653792	0.695530
48	6	0	1.238913	4.786337	0.339892
49	1	0	2.219936	5.252806	0.242576
50	1	0	0.615489	5.076043	−0.512112
51	1	0	0.742198	5.133233	1.249371
52	46	0	0.489228	0.638303	0.791183
53	6	0	4.813403	0.243691	−2.509930
54	1	0	4.685419	−0.774267	−2.890919
55	1	0	5.687333	0.684519	−2.999589
56	1	0	5.009501	0.198813	−1.434954

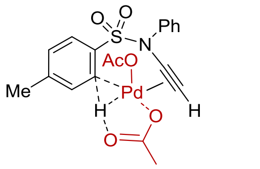

TS1 (R = H)

Thermal correction to Gibbs Free Energy = 0.277740 Hartree

Electronic energy = −1767.316472 Hartree

Center Number	Atomic Number	Atomic Type	Coordinates (Angstroms) X	Y	Z
1	6	0	0.363397	−2.269256	−0.146856
2	6	0	0.763226	−3.383472	−0.878663
3	6	0	2.120470	−3.546781	−1.170938
4	6	0	1.270788	−1.289214	0.322529
5	6	0	2.624394	−1.521908	0.025336
6	6	0	3.070660	−2.621348	−0.729319
7	1	0	0.032403	−4.114573	−1.205368
8	1	0	3.360333	−0.811716	0.395184
9	8	0	−1.617171	−1.707572	1.590795
10	16	0	−1.386920	−2.175580	0.229914
11	8	0	−2.081686	−3.344822	−0.305265

12	6	0	−3.150728	−0.074491	−0.398123
13	6	0	−4.337377	−0.810063	−0.414219
14	6	0	−3.120864	1.258115	0.020822
15	6	0	−5.517809	−0.193162	−0.001246
16	1	0	−4.329565	−1.842908	−0.745028
17	6	0	−4.312756	1.864083	0.415573
18	1	0	−2.183634	1.806503	0.032006
19	6	0	−5.508457	1.141683	0.408800
20	1	0	−6.445979	−0.756049	−0.008546
21	1	0	−4.302167	2.900708	0.737829
22	1	0	−6.431662	1.618675	0.723530
23	7	0	−1.933190	−0.725714	−0.826876
24	1	0	1.086355	−0.712323	1.471612
25	6	0	−0.989672	−0.106238	−1.458515
26	6	0	0.091377	0.390438	−1.879186
27	8	0	1.873619	1.552371	1.686591
28	6	0	1.743922	0.840827	2.736747
29	8	0	1.247195	−0.323653	2.759186
30	6	0	2.195368	1.459147	4.042497
31	1	0	1.357168	2.025626	4.462094
32	1	0	2.477716	0.682529	4.754271
33	1	0	3.020371	2.151655	3.869645
34	6	0	4.535305	−2.783270	−1.049917
35	1	0	5.146337	−2.718786	−0.143447
36	1	0	4.738798	−3.743078	−1.531128
37	1	0	4.874628	−1.988556	−1.724064
38	1	0	2.437904	−4.414791	−1.741926
39	8	0	2.101910	2.624114	−2.222277
40	6	0	1.359923	3.304690	−1.516000
41	8	0	0.631717	2.855522	−0.519916
42	6	0	1.208788	4.808810	−1.713732
43	1	0	1.661377	5.102381	−2.661681
44	1	0	0.155656	5.100174	−1.688419
45	1	0	1.714047	5.331011	−0.894958
46	46	0	0.935413	0.904868	−0.060904
47	1	0	0.619544	0.589633	−2.796953

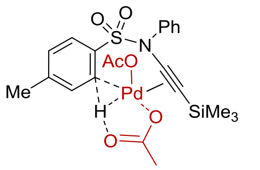

Thermal correction to Gibbs Free Energy = 0.372266 Hartree

Electronic energy = −2175.961643 Hartree

Center Number	Atomic Number	Atomic Type	Coordinates (Angstroms) X	Y	Z
1	6	0	0.353359	−2.400652	0.088495
2	6	0	0.943406	−3.399061	−0.677722
3	6	0	2.339660	−3.482315	−0.716423
4	6	0	1.103973	−1.461894	0.835202
5	6	0	2.499883	−1.603532	0.776778
6	6	0	3.138983	−2.590501	0.005479
7	1	0	0.327545	−4.103360	−1.225702
8	1	0	3.110099	−0.922376	1.365385
9	8	0	−1.940192	−2.089974	1.464049
10	16	0	−1.442769	−2.385563	0.125256
11	8	0	−1.961907	−3.516280	−0.643400
12	6	0	−3.137222	−0.314642	−0.671369
13	6	0	−4.246593	−1.095832	−1.004712
14	6	0	−3.282785	0.980217	−0.163952
15	6	0	−5.524483	−0.569693	−0.819292
16	1	0	−4.104294	−2.095119	−1.401140
17	6	0	−4.568500	1.497058	−0.004642
18	1	0	−2.409541	1.572684	0.093948
19	6	0	−5.688065	0.725913	−0.324797
20	1	0	−6.391749	−1.171760	−1.072414
21	1	0	−4.691514	2.503752	0.383128
22	1	0	−6.685301	1.133670	−0.189418
23	7	0	−1.820192	−0.877387	−0.870674
24	1	0	0.694256	−1.092275	1.984262
25	6	0	−0.781496	−0.133975	−1.167614
26	6	0	0.300500	0.452832	−1.421996
27	8	0	0.874155	1.201725	2.682833
28	6	0	0.762623	0.302233	3.582428
29	8	0	0.587650	−0.926345	3.352107

30	6	0	0.819673	0.775222	5.019953
31	1	0	−0.184486	1.097550	5.315133
32	1	0	1.130715	−0.038262	5.676142
33	1	0	1.490797	1.630591	5.111456
34	6	0	4.644570	−2.684528	−0.021536
35	1	0	5.041178	−2.885927	0.979859
36	1	0	4.986167	−3.481833	−0.686245
37	1	0	5.091411	−1.743660	−0.360777
38	1	0	2.806550	−4.263551	−1.309913
39	8	0	1.945843	3.272974	0.004366
40	6	0	0.769664	3.517797	0.271785
41	8	0	−0.157476	2.620361	0.500126
42	6	0	0.243428	4.944275	0.377082
43	1	0	0.998703	5.644394	0.018065
44	1	0	−0.681106	5.058838	−0.195318
45	1	0	0.011688	5.165271	1.423737
46	46	0	0.529041	0.720603	0.693421
47	14	0	1.509319	0.968383	−2.779874
48	6	0	3.258597	0.917443	−2.097812
49	1	0	3.975212	1.232726	−2.864947
50	1	0	3.330119	1.602851	−1.248393
51	1	0	3.535545	−0.089521	−1.770660
52	6	0	1.040702	2.702272	−3.344503
53	1	0	1.253356	3.422243	−2.550350
54	1	0	1.632332	2.979418	−4.224865
55	1	0	−0.017782	2.773192	−3.613598
56	6	0	1.279562	−0.280228	−4.182627
57	1	0	1.950782	−0.045768	−5.017012
58	1	0	1.503152	−1.299844	−3.851669
59	1	0	0.254241	−0.268445	−4.567096

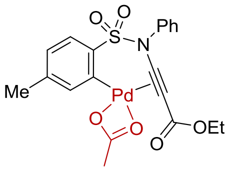

8 (R = CO_2Et)

Thermal correction to Gibbs Free Energy = 0.290265 Hartree

Electronic energy = −1805.431156 Hartree

Center Atomic Atomic Coordinates (Angstroms)

Number	Number	Type	X	Y	Z
1	6	0	1.026871	−2.040499	0.245768
2	6	0	1.692568	−3.021147	0.986285
3	6	0	1.045678	−3.576283	2.086334
4	6	0	−0.257641	−1.597170	0.575230
5	6	0	−0.880712	−2.168134	1.686132
6	6	0	−0.242814	−3.159091	2.451758
7	46	0	−1.252546	−0.277305	−0.548647
8	1	0	2.688772	−3.336936	0.695364
9	1	0	−1.882834	−1.850564	1.960991
10	8	0	1.028327	−1.526426	−2.386006
11	16	0	1.845947	−1.361398	−1.187970
12	8	0	3.262414	−1.709328	−1.177837
13	6	0	2.963465	1.083678	−0.355666
14	6	0	4.044444	1.191621	−1.234798
15	6	0	3.017177	1.649603	0.921225
16	6	0	5.194395	1.861249	−0.820159
17	1	0	3.980977	0.753658	−2.223914
18	6	0	4.166463	2.332640	1.316408
19	1	0	2.164687	1.572793	1.587348
20	6	0	5.257547	2.434945	0.451434
21	1	0	6.037715	1.943204	−1.498894
22	1	0	4.206933	2.782517	2.303629
23	1	0	6.151695	2.965221	0.764885
24	7	0	1.774535	0.395088	−0.799866
25	6	0	0.563748	0.838502	−0.451992
26	6	0	−0.332934	1.550189	0.075301
27	6	0	−0.944656	2.710239	0.690077
28	8	0	−0.316319	3.728142	0.910162
29	8	0	−2.237804	2.514771	0.994578
30	6	0	−2.922022	3.650876	1.580874
31	1	0	−2.833251	4.500766	0.897512
32	1	0	−2.418589	3.918931	2.514968
33	8	0	−2.890213	−1.486191	−1.075593
34	6	0	−3.405909	−0.552726	−1.799576
35	8	0	−2.842279	0.581050	−1.835471
36	6	0	−4.642590	−0.844935	−2.602479
37	1	0	−4.360805	−1.423875	−3.488400

38	1	0	−5.336996	−1.451170	−2.015983
39	1	0	−5.117293	0.083856	−2.921496
40	6	0	−0.927116	−3.742404	3.664546
41	1	0	−0.583068	−4.760825	3.865793
42	1	0	−0.715687	−3.142375	4.558349
43	1	0	−2.013033	−3.766307	3.536726
44	1	0	1.546628	−4.345302	2.667534
45	6	0	−4.365277	3.244131	1.802929
46	1	0	−4.919680	4.079414	2.242025
47	1	0	−4.841574	2.971043	0.857334
48	1	0	−4.431502	2.390878	2.484138

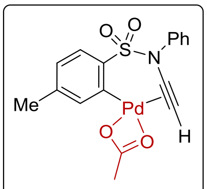

8 (R = H)

Thermal correction to Gibbs Free Energy = 0.228029 Hartree

Electronic energy = −1538.280339 Hartree

Center Number	Atomic Number	Atomic Type	Coordinates (Angstroms) X	Y	Z
1	6	0	0.598270	0.948694	1.067130
2	6	0	1.278462	2.142697	1.361797
3	6	0	0.854163	3.324632	0.775455
4	6	0	−0.495714	0.891824	0.188375
5	6	0	−0.887787	2.118127	−0.379554
6	6	0	−0.234932	3.327131	−0.110648
7	46	0	−1.756875	−0.605520	−0.284150
8	1	0	2.117342	2.128559	2.048313
9	1	0	−1.748041	2.133395	−1.042786
10	8	0	0.266386	−1.334723	2.505457
11	16	0	1.269403	−0.509442	1.847066
12	8	0	2.551722	−0.210089	2.477562
13	6	0	2.798103	−0.992059	−0.505028
14	6	0	4.088496	−1.136717	0.009358
15	6	0	2.579748	−0.438247	−1.769491
16	6	0	5.173703	−0.714813	−0.758220
17	1	0	4.230095	−1.566594	0.993613

18	6	0	3.674918	−0.032030	−2.530342
19	1	0	1.567651	−0.335996	−2.147305
20	6	0	4.970725	−0.166455	−2.026355
21	1	0	6.179814	−0.823576	−0.365274
22	1	0	3.513083	0.394548	−3.515553
23	1	0	5.820406	0.153655	−2.621801
24	7	0	1.682770	−1.446013	0.296354
25	6	0	0.612663	−1.955037	−0.231522
26	6	0	−0.547724	−2.264246	−0.612282
27	8	0	−3.579957	0.473635	−0.061408
28	6	0	−4.251945	−0.571082	−0.378428
29	8	0	−3.629317	−1.640642	−0.673888
30	6	0	−5.755362	−0.527961	−0.363865
31	1	0	−6.105576	−0.728940	0.654466
32	1	0	−6.109259	0.463558	−0.653452
33	1	0	−6.162545	−1.292401	−1.027702
34	6	0	−0.682622	4.608863	−0.770120
35	1	0	−0.739510	5.427737	−0.045483
36	1	0	0.024673	4.916803	−1.549898
37	1	0	−1.664708	4.496948	−1.236372
38	1	0	1.368369	4.253006	1.008351
39	1	0	−1.075036	−3.178978	−0.835079

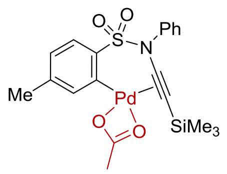

8 (R = SiMe3)

Thermal correction to Gibbs Free Energy = 0.320839 Hartree

Electronic energy = −1946.926469 Hartree

Center Number	Atomic Number	Atomic Type	Coordinates (Angstroms) X	Y	Z
1	6	0	0.978182	−2.052627	−0.253593
2	6	0	1.758167	−3.134535	0.167528
3	6	0	1.344100	−3.863713	1.276782
4	6	0	−0.195469	−1.673894	0.404986
5	6	0	−0.585672	−2.425820	1.517778
6	6	0	0.171792	−3.519186	1.967162

7	46	0	−1.421999	−0.245881	−0.255301
8	1	0	2.662299	−3.390832	−0.374288
9	1	0	−1.503090	−2.170601	2.040835
10	8	0	0.479493	−1.061238	−2.700542
11	16	0	1.519052	−1.124565	−1.677550
12	8	0	2.895851	−1.474225	−2.013770
13	6	0	2.835514	0.951384	−0.382651
14	6	0	3.900201	1.262590	−1.232299
15	6	0	2.969446	1.068367	1.003890
16	6	0	5.109127	1.689329	−0.684108
17	1	0	3.775145	1.162743	−2.304054
18	6	0	4.178727	1.509601	1.540026
19	1	0	2.130911	0.826957	1.648788
20	6	0	5.250451	1.817566	0.699247
21	1	0	5.938741	1.929556	−1.342012
22	1	0	4.283984	1.606032	2.616587
23	1	0	6.191394	2.158548	1.120585
24	7	0	1.590007	0.513230	−0.972073
25	6	0	0.425734	0.949066	−0.454549
26	6	0	−0.406857	1.636121	0.187895
27	8	0	−3.115191	−1.480202	−0.514402
28	6	0	−3.833445	−0.496124	−0.918287
29	8	0	−3.339048	0.673958	−0.914395
30	6	0	−5.227588	−0.753560	−1.420334
31	1	0	−5.175539	−1.021068	−2.481336
32	1	0	−5.676948	−1.590618	−0.882236
33	1	0	−5.839780	0.144178	−1.319095
34	6	0	−0.256718	−4.295754	3.189270
35	1	0	0.002236	−5.354916	3.099078
36	1	0	0.240423	−3.914685	4.090051
37	1	0	−1.335235	−4.219975	3.352671
38	1	0	1.935337	−4.712259	1.609796
39	14	0	−1.361111	3.072923	0.909613
40	6	0	−0.044463	4.218618	1.638061
41	1	0	−0.518535	5.107353	2.070670
42	1	0	0.527847	3.725176	2.429982
43	1	0	0.660926	4.556041	0.872071
44	6	0	−2.325215	3.921379	−0.465537
45	1	0	−2.896527	4.769869	−0.071090

46	1	0	−1.655904	4.297607	−1.245899
47	1	0	−3.023203	3.214867	−0.923579
48	6	0	−2.512327	2.426537	2.253234
49	1	0	−3.053205	3.253472	2.727491
50	1	0	−3.249817	1.741462	1.824207
51	1	0	−1.959790	1.892701	3.033223

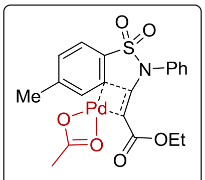

TS2 (R = CO_2Et)

Thermal correction to Gibbs Free Energy = 0.290461 Hartree

Electronic energy = −1805.408101 Hartree

Center Number	Atomic Number	Atomic Type	Coordinates (Angstroms) X	Y	Z
1	6	0	−0.455806	−2.531221	−0.163710
2	6	0	−0.437388	−3.833387	−0.652809
3	6	0	0.588668	−4.200090	−1.521337
4	6	0	0.487851	−1.574809	−0.540322
5	6	0	1.440554	−1.936784	−1.506476
6	6	0	1.529944	−3.258655	−1.968267
7	46	0	1.308086	0.007823	0.536561
8	1	0	−1.227933	−4.526913	−0.385984
9	1	0	2.135179	−1.194452	−1.888270
10	8	0	−1.613060	−1.580949	2.092935
11	16	0	−1.874346	−1.894611	0.688828
12	8	0	−3.079162	−2.628844	0.313538
13	6	0	−2.932279	0.543916	0.041650
14	6	0	−2.777402	1.582897	0.964341
15	6	0	−4.150594	0.330888	−0.607000
16	6	0	−3.856048	2.426062	1.223091
17	1	0	−1.826716	1.726723	1.465930
18	6	0	−5.226296	1.174410	−0.330391
19	1	0	−4.242535	−0.487662	−1.311696
20	6	0	−5.079238	2.222967	0.579140
21	1	0	−3.742068	3.238468	1.934219
22	1	0	−6.176200	1.014157	−0.830970
23	1	0	−5.916583	2.882538	0.786680

24	7	0	−1.852654	−0.374385	−0.243198
25	6	0	−0.595819	0.094219	−0.486732
26	6	0	0.139177	1.134526	−0.715460
27	6	0	0.194108	2.440212	−1.352110
28	8	0	−0.773164	2.973730	−1.864279
29	8	0	1.427165	2.976704	−1.299145
30	6	0	1.563530	4.295854	−1.878325
31	1	0	0.877267	4.976525	−1.364425
32	1	0	1.260779	4.252460	−2.929527
33	8	0	2.631939	−0.888807	2.007120
34	6	0	3.079166	0.269537	2.289964
35	8	0	2.653182	1.278226	1.628738
36	6	0	4.066601	0.459369	3.409120
37	1	0	3.516061	0.603265	4.345186
38	1	0	4.691861	−0.429080	3.515286
39	1	0	4.679846	1.344530	3.231202
40	6	0	2.623194	−3.658950	−2.928612
41	1	0	3.493760	−4.043510	−2.383593
42	1	0	2.288512	−4.447114	−3.609043
43	1	0	2.961069	−2.808437	−3.527038
44	1	0	0.629427	−5.217773	−1.898787
45	6	0	3.011491	4.714938	−1.714702
46	1	0	3.679868	4.023048	−2.235153
47	1	0	3.156158	5.715618	−2.133911
48	1	0	3.292733	4.736351	−0.658333

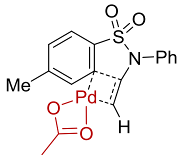

TS2 (R = H)

Thermal correction to Gibbs Free Energy = 0.228440 Hartree

Electronic energy = −1538.254519 Hartree

Center Number	Atomic Number	Atomic Type	Coordinates (Angstroms) X	Y	Z
1	6	0	−0.267308	1.828331	0.689663
2	6	0	−0.651829	3.132453	0.985477
3	6	0	−1.745628	3.670179	0.311245
4	6	0	−0.910660	1.055532	−0.277892

5	6	0	−1.932785	1.663515	−1.025916
6	6	0	−2.390319	2.951004	−0.708248
7	46	0	−1.283626	−0.994927	−0.376327
8	1	0	−0.082351	3.718275	1.699443
9	1	0	−2.395624	1.127346	−1.849078
10	8	0	1.113913	0.069402	2.207995
11	16	0	1.276862	1.177311	1.265368
12	8	0	2.224023	2.249466	1.558496
13	6	0	2.960033	−0.162928	−0.389056
14	6	0	3.085431	−1.509950	−0.032658
15	6	0	4.074486	0.585507	−0.772961
16	6	0	4.341515	−2.111926	−0.083954
17	1	0	2.211462	−2.070373	0.280616
18	6	0	5.328535	−0.025160	−0.812748
19	1	0	3.946614	1.631189	−1.030042
20	6	0	5.461851	−1.372535	−0.473371
21	1	0	4.446613	−3.157262	0.190305
22	1	0	6.198529	0.552259	−1.110189
23	1	0	6.438159	−1.846866	−0.508840
24	7	0	1.684885	0.520826	−0.331864
25	6	0	0.602606	−0.047642	−0.971365
26	6	0	0.192693	−0.956542	−1.779559
27	8	0	−2.861792	−1.413125	1.064369
28	6	0	−2.981655	−2.577173	0.565677
29	8	0	−2.266538	−2.897505	−0.447353
30	6	0	−3.920438	−3.583561	1.174067
31	1	0	−3.379777	−4.155891	1.935760
32	1	0	−4.756806	−3.075830	1.657762
33	1	0	−4.278564	−4.279597	0.413258
34	6	0	−3.558995	3.550254	−1.452081
35	1	0	−4.497389	3.354283	−0.919300
36	1	0	−3.457832	4.635122	−1.548240
37	1	0	−3.656563	3.123869	−2.454303
38	1	0	−2.071030	4.680597	0.541581
39	1	0	0.355329	−1.555314	−2.656533

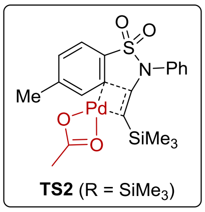

Thermal correction to Gibbs Free Energy = 0.320403 Hartree

Electronic energy = −1946.900134 Hartree

Center Number	Atomic Number	Atomic Type	Coordinates (Angstroms) X	Y	Z
1	6	0	0.599955	2.474035	−0.086555
2	6	0	1.130774	3.711935	0.260452
3	6	0	2.218830	3.746300	1.129626
4	6	0	1.085511	1.270351	0.431345
5	6	0	2.104678	1.345364	1.398627
6	6	0	2.710469	2.568943	1.717555
7	46	0	1.284312	−0.607131	−0.444788
8	1	0	0.677230	4.623171	−0.115360
9	1	0	2.447239	0.442151	1.894661
10	8	0	−0.827750	1.798584	−2.283131
11	16	0	−0.952164	2.342771	−0.929778
12	8	0	−1.778646	3.525066	−0.696934
13	6	0	−2.843739	0.567430	−0.140464
14	6	0	−3.048724	−0.497383	−1.025352
15	6	0	−3.924464	1.221028	0.455479
16	6	0	−4.349944	−0.920817	−1.291273
17	1	0	−2.198977	−0.981299	−1.493916
18	6	0	−5.223594	0.795911	0.173501
19	1	0	−3.735648	2.052501	1.125313
20	6	0	−5.437122	−0.276585	−0.693598
21	1	0	−4.515343	−1.747097	−1.976195
22	1	0	−6.066261	1.301862	0.634761
23	1	0	−6.448610	−0.609070	−0.907504
24	7	0	−1.518143	1.062153	0.156671
25	6	0	−0.523487	0.166701	0.502126
26	6	0	−0.294015	−1.057098	0.841510
27	8	0	2.923646	−0.538724	−1.892088
28	6	0	2.861898	−1.803364	−1.997328
29	8	0	2.033891	−2.454343	−1.267852

30	6	0	3.721719	−2.534535	−2.994237
31	1	0	3.178279	−2.608135	−3.942596
32	1	0	4.646500	−1.982975	−3.172548
33	1	0	3.937032	−3.546028	−2.644534
34	6	0	3.874089	2.618182	2.677869
35	1	0	4.825812	2.597785	2.133224
36	1	0	3.859857	3.533600	3.276606
37	1	0	3.867960	1.762326	3.358480
38	1	0	2.659041	4.702620	1.397226
39	14	0	−0.598624	−2.701311	1.662060
40	6	0	−0.843304	−3.992260	0.311218
41	1	0	−0.964838	−4.990938	0.746552
42	1	0	−1.733878	−3.776848	−0.288141
43	1	0	0.022886	−4.009158	−0.357647
44	6	0	−2.138785	−2.528376	2.744427
45	1	0	−3.011195	−2.237159	2.150942
46	1	0	−2.369327	−3.481121	3.235239
47	1	0	−1.995789	−1.775043	3.525942
48	6	0	0.916579	−3.118736	2.708392
49	1	0	1.085829	−2.368022	3.487430
50	1	0	0.789626	−4.089876	3.200394
51	1	0	1.813502	−3.173103	2.082963
